# [^177^Lu]Lu-DOTA-TATE plus long-acting octreotide in patients with newly diagnosed, advanced, grade 2–3, gastroenteropancreatic neuroendocrine tumours: preplanned and post-hoc efficacy analyses from the randomised, phase 3 NETTER-2 trial

**DOI:** 10.1016/j.eclinm.2026.104109

**Published:** 2026-08-07

**Authors:** Diego Ferone, Marianne Pavel, Ken Herrmann, Pamela L. Kunz, Sten Myrehaug, Daniel Halperin, Beth Chasen, Jaume Capdevila, Amparo García-Burillo, Salvatore Tafuto, Secondo Lastoria, Do-Youn Oh, Changhoon Yoo, Stephen Falk, Thorvardur R. Halfdanarson, Maddalena Sansovini, Laura Gerard, Emmanuel Deshayes, Angelina Filice, Ilya Folitar, Yufen Zhang, Wouter W. de Herder, Simron Singh

**Affiliations:** a Endocrinology, Department of Internal Medicine and Medical Specialties (DiMI), University of Genova, IRCCS Ospedale Policlinico San Martino, Genova, Italy; b Department of Medicine 1, Uniklinikum Erlangen, and Comprehensive Cancer Center Erlangen-EMN, Friedrich Alexander-Universität Erlangen-Nürnberg, Erlangen, Germany; c Department of Nuclear Medicine, University of Duisburg-Essen, and German Cancer Consortium (DKTK)-University Hospital Essen, Essen, Germany; d National Center for Tumor Diseases (NCT), NCT West, Heidelberg, Germany; e Yale School of Medicine and Yale Cancer Center, Yale University, New Haven, CT, USA; f Sunnybrook Odette Cancer Centre, University of Toronto, Toronto, ON, Canada; g MD Anderson Cancer Center, Houston, TX, USA; h Department of Medical Oncology, Gastrointestinal and Endocrine Tumor Unit, Vall d'Hebron Hospital Universitari, Vall d’Hebron Barcelona Hospital Campus, Barcelona, Spain; i Neuroendocrine and Endocrine Tumor Translational Research Program (NET-VHIO), Vall d'Hebron Institute of Oncology (VHIO), Vall d’Hebron Barcelona Hospital Campus, Barcelona, Spain; j Nuclear Medicine Department, Hospital Universitari Vall d’Hebron, Barcelona, Spain; k Sarcoma and Rare Tumors Unit, Istituto Nazionale Tumori IRCCS, Fondazione G. Pascale, Naples, Italy; l Division of Nuclear Medicine, Istituto Nazionale Tumori IRCCS, Fondazione G. Pascale, Naples, Italy; m Seoul National University Hospital, Cancer Research Institute, Seoul National University College of Medicine, Integrated Major in Innovative Medical Science, Seoul National University Graduate School, Seoul, South Korea; n Asan Medical Center, University of Ulsan College of Medicine, Seoul, South Korea; o Bristol Haematology and Oncology Centre, University Hospitals Bristol NHS Foundation Trust, Bristol, UK; p Mayo Clinic, Rochester, MN, USA; q IRCCS Istituto Romagnolo per lo Studio dei Tumori Dino Amadori, Meldola, Italy; r Edouard Herriot University Hospital, Hospices Civils de Lyon, Lyon, France; s Unicancer, Montpellier Cancer Institute (ICM), Montpellier University, Montpellier, France; t AUSL-IRCCS of Reggio Emilia, Reggio Emilia, Italy; u Novartis Pharma AG, Basel, Switzerland; v Novartis Pharmaceuticals Corp, East Hanover, NJ, USA; w Erasmus MC and Erasmus MC Cancer Institute, Rotterdam, Netherlands

**Keywords:** [^177^Lu]Lu-DOTA-TATE, ^177^Lu-DOTATATE, Gastroenteropancreatic neuroendocrine tumours, Efficacy analyses, Subgroup analyses

## Abstract

**Background:**

In the phase 3 NETTER-2 study, first-line [^177^Lu]Lu-DOTA-TATE (hereafter ^177^Lu-DOTATATE) significantly improved progression-free survival in patients with advanced, well-differentiated, higher grade 2–3 (Ki67 ≥10% and ≤55%), somatostatin receptor-positive gastroenteropancreatic neuroendocrine tumours (GEP-NETs). Median progression-free survival was 22·8 months (95% CI 19·4–not estimable [NE]) with ^177^Lu-DOTATATE *vs* 8·5 months (7·7–13·8) in the control arm. Here, we report subgroup analyses, including preplanned assessments of efficacy by NET grade (G) and site of origin; further post-hoc analyses are reported in the main text.

**Methods:**

In this open-label, parallel-group study, patients from nine countries were randomised 2:1 to receive four cycles of ^177^Lu-DOTATATE plus octreotide long-acting repeatable (LAR) 30 mg every 8 weeks then octreotide LAR 30 mg every 4 weeks, or high-dose octreotide LAR 60 mg every 4 weeks. The primary endpoint (progression-free survival) was previously reported. Tumours were assessed at baseline, week 16, week 24, then every 12 weeks until disease progression or death. Preplanned subgroup analyses used blinded independent centrally reviewed data. The study is registered with ClinicalTrials.gov, NCT03972488, and is completed.

**Findings:**

Between Jan 22, 2020, and Oct 13, 2022, 261 patients were screened; 35 were excluded due to screen failure, and 226 were randomised. ^177^Lu-DOTATATE (n = 151) reduced risk of disease progression/death *vs* control (n = 75) regardless of NET grade/origin (hazard ratio [95% CI]: G2 NET 0·31 [0·18–0·53]; G3 NET 0·27 [0·14–0·49]; pancreatic NET 0·34 [0·20–0·56]; gastrointestinal NET 0·23 [0·12–0·46]). Median progression-free survival in months (95% CI) with ^177^Lu-DOTATATE was: G2 NET 29·0 (21·8–NE); G3 NET 22·2 (13·9–27·8); pancreatic NET 19·4 (16·6–24·9); gastrointestinal NET NE (22·6–NE). ^177^Lu-DOTATATE improved objective response rate *vs* control regardless of NET grade/origin.

**Interpretation:**

These findings support the use of first-line ^177^Lu-DOTATATE for patients with advanced, well-differentiated, higher grade 2–3 (Ki67 ≥10% and ≤55%), somatostatin receptor-positive GEP-NETs, and for whom chemotherapy is not considered the most appropriate treatment option, regardless of NET grade (2/3) or origin (pancreas/gastrointestinal).

**Funding:**

Advanced Accelerator Applications, a Novartis Company.

**Research in context**

**Evidence before this study**

Somatostatin analogues were previously established as the standard of care for patients with advanced grade 1 and grade 2 gastroenteropancreatic neuroendocrine tumours (GEP-NETs) based on the phase 3 PROMID and CLARINET studies. Subsequently, the phase 3 NETTER-1 trial established the efficacy and safety of the radioligand therapy [^177^Lu]Lu-DOTA-TATE (hereafter ^177^Lu-DOTATATE) in combination with octreotide long-acting repeatable (LAR) 30 mg in patients with advanced somatostatin receptor (SSTR)-positive grade 1 and grade 2 midgut NETs who had progressed on somatostatin analogues. Based on these results ^177^Lu-DOTATATE became the standard of care in this patient population.

Despite these advances, there were a lack of data to support first-line treatment options for patients with advanced higher grade 2 and grade 3 well-differentiated GEP-NETs. These patients have a poor prognosis compared with those who have lower-grade disease and represent a significant clinical unmet need. In this context, NETTER-2 was the first randomised study to investigate a radioligand therapy as a first-line treatment for any malignancy. The primary analysis found that ^177^Lu-DOTATATE plus octreotide LAR 30 mg prolonged progression-free survival in patients with newly diagnosed, advanced, well-differentiated, higher grade 2 and grade 3, SSTR-positive GEP-NETs compared with control (high-dose octreotide LAR 60 mg). Given the heterogeneity of GEP-NETs, subgroup analysis by tumour grade and primary tumour site are particularly important to better understand treatment effects across different patient populations.

**Added value of this study**

While the primary results of NETTER-2 indicate that ^177^Lu-DOTATATE is efficacious in patients with advanced, well-differentiated, higher grade 2 or grade 3, somatostatin receptor-positive GEP-NETs, a more comprehensive understanding of treatment effects in specific patient populations is required. In our preplanned analyses of patient subgroups from NETTER-2, a progression-free survival and objective response rate benefit in favour of ^177^Lu-DOTATATE was observed across all evaluable subgroups regardless of NET grade (2 *vs* 3), site of origin (pancreas *vs* gastrointestinal), or somatostatin receptor uptake score (3 *vs* 4). Additional post-hoc subgroup analyses suggest that the progression-free survival and objective response rate benefit of ^177^Lu-DOTATATE *vs* control is consistent regardless of NET grade plus site of origin. Furthermore, in post-hoc analyses, treatment effects on progression-free survival and objective response rate appeared to be minimally affected when adjusted for baseline covariates using multivariate regression models. Our results provide a more comprehensive understanding of the effect of ^177^Lu-DOTATATE in different populations. This will help healthcare professionals to make informed treatment decisions and improve outcomes for patients with higher grade 2–3 GEP-NETs.

**Implications of all the available evidence**

These efficacy analyses provide additional insights into the clinical benefit of ^177^Lu-DOTATATE across different patient subgroups. The findings supplement the primary outcomes of NETTER-2 and support the use of first-line ^177^Lu-DOTATATE in patients with advanced, well-differentiated, higher grade 2–3, somatostatin receptor-positive GEP-NETs, regardless of NET grade (2 *vs* 3) or site of origin (pancreas *vs* gastrointestinal) and regardless of the somatostatin receptor uptake score (3 *vs* 4).

## Introduction

[^177^Lu]Lu-DOTA-TATE (hereafter ^177^Lu-DOTATATE) is a β-emitting radioligand therapy with a high affinity for somatostatin receptor subtype 2, which is the subtype most commonly found in gastroenteropancreatic neuroendocrine tumours (GEP-NETs), being expressed in approximately 80–90% of GEP-NETs.^1^^,^^2^ The results of the phase 3 NETTER-1 trial provided evidence to support the use of ^177^Lu-DOTATATE as a standard of care in patients with advanced, somatostatin receptor-positive midgut NETs who have experienced disease progression following treatment with non-radiolabelled first-generation somatostatin analogues.^3^

NETTER-2 was the first randomised study to evaluate the first-line efficacy of ^177^Lu-DOTATATE in patients with advanced, well-differentiated, higher grade 2 or grade 3 (Ki67 ≥10 and ≤55%), somatostatin receptor-positive GEP-NETs. NETTER-2 was also the first randomised study of a radioligand therapy in the first-line metastatic setting for any solid tumour.^4^ When NETTER-2 was designed, there was no recommended standard of care for patients with newly diagnosed, well-differentiated, higher grade 2 or grade 3 GEP-NETs, representing a significant unmet need for this patient population. The study met its primary objective of progression-free survival, with a 72% reduction (p < 0·0001) in the risk of disease progression or death with ^177^Lu-DOTATATE *vs* control after 101 progression-free survival events.^4^ Median progression-free survival was 22·8 months (95% CI 19·4–not estimable) with ^177^Lu-DOTATATE *vs* 8·5 months (7·7–13·8) with control.^4^ Objective response rate was also significantly higher with ^177^Lu-DOTATATE (43% [95% CI 35–51]) *vs* control (9% [4–18]).^4^ Overall survival data were immature at the time of primary analysis and at the second overall survival interim analysis (data cutoff May 24, 2024). With deaths recorded for ∼31% of randomised patients at the second overall survival interim analysis, the median overall survival was 43·3 months (95% CI 37·3–not estimable) with ^177^Lu-DOTATATE and not estimable with the control.^5^ Overall survival monitoring is ongoing.

The primary results of NETTER-2 suggest that ^177^Lu-DOTATATE should be considered a new standard of care for the first-line treatment of patients with advanced, well-differentiated, higher grade 2 or 3, somatostatin receptor-positive GEP-NETs. Nevertheless, NETs are extremely heterogenous, with outcomes based on stage, grade, primary location, and somatostatin receptor expression.^6^^,^^7^ As such, in this Article we report efficacy subgroup analyses from NETTER-2, including both preplanned analyses (progression-free survival and objective response rate of ^177^Lu-DOTATATE *vs* control by NET grade, site of origin, and somatostatin receptor uptake score) and post-hoc analyses. In addition, multivariate analyses were performed to assess the treatment effect of ^177^Lu-DOTATATE when adjusted for baseline covariates (including disease spread) and identify potential prognostic factors for progression-free survival and objective response rate. The results of these analyses provide a more comprehensive understanding of treatment effects in different populations and thus may help healthcare professionals to make more informed treatment decisions and improve patient outcomes.

## Methods

### Study design and participants

NETTER-2 was a prospective, open-label, parallel-group, randomised phase 3 study conducted in 45 centres across 9 countries.^4^ The full eligibility criteria for NETTER-2 have been published in the primary manuscript.^4^ In brief, eligible patients were aged ≥15 years with advanced, well-differentiated, higher grade 2 or 3 (Ki67 ≥10% and ≤55%), somatostatin receptor-positive GEP-NETs that were diagnosed within 6 months of enrolment. Somatostatin receptor expression was determined from images obtained from any somatostatin receptor imaging modality within the 3 months preceding randomisation, with scoring based on a visual semiquantitative scale. Written informed consent was provided by all patients.

### Ethics

The trial protocol was approved by the institutional review board or independent ethics committee at each participating centre, the details of which, together with corresponding reference numbers, are included in the appendix (Table S1). The trial was carried out in accordance with the principles of the Declaration of Helsinki, the International Conference on Harmonisation Good Clinical Practice guidelines, and all applicable regulations.^4^ Regulatory approval for the trial was obtained in the EU (by the EMA) and the US (by the FDA) (Regulatory Agency Identifier numbers: 2019-001562-15 [EudraCT], 2023-507443-10-00 [EU CT], 77219 [IND]). The protocol, with amendments, and statistical analysis plan have been published.^4^ NETTER-2 was registered with ClinicalTrials.gov, NCT03972488, in May 2019.

### Randomisation and masking

Interactive response technologies (web and voice; Calyx, Nottingham, UK) were used to randomly assign patients in a 2:1 ratio to the ^177^Lu-DOTATATE arm or the control arm, with stratification by NET grade (2 *vs* 3) and site of origin (pancreas *vs* other).^4^ The 2:1 randomisation design was chosen to increase patients’ chances of receiving ^177^Lu-DOTATATE. Patients were offered to cross over to ^177^Lu-DOTATATE after centrally confirmed radiological progression to minimise dropout from the control arm. The randomisation list included 240 pre-allocated records for each of the four study strata (960 records in total). Within each stratum, the first enrolled patient was assigned the first randomisation schedule in the pre-allocated sequence, and subsequent patients were assigned sequentially to the next available entry within that stratum-specific list. Randomisation was performed using blocks of six within each stratum and forced randomisation was not permitted in this study. As the trial was open label, treatment masking was not applicable.

### Procedures

Patients were randomised 2:1 to receive ^177^Lu-DOTATATE plus octreotide long-acting repeatable (LAR) 30 mg (^177^Lu-DOTATATE arm) or high-dose octreotide LAR 60 mg (control arm).^4^ In the ^177^Lu-DOTATATE arm, ^177^Lu-DOTATATE (7·4 GBq [200 mCi]) was administered every 8 weeks for a total of four cycles (cumulative dose 29·6 GBq [800 mCi]), while intramuscular octreotide LAR 30 mg was administered after each ^177^Lu-DOTATATE infusion every 8 weeks for a total of four ^177^Lu-DOTATATE cycles, and then every 4 weeks. Patients in the ^177^Lu-DOTATATE arm received a 2·5% lysine–arginine solution for renal protection. In the control arm, patients received intramuscular high-dose octreotide LAR (60 mg) every 4 weeks (per the control arm in the NETTER-1 study).^3^ As previously reported, tumours were assessed in both arms at baseline, week 16, week 24, and then every 12 weeks until centrally confirmed disease progression or death.^4^ Randomised treatment continued until centrally confirmed disease progression or treatment discontinuation.^4^ Subject to meeting protocol criteria, patients in the control arm who experienced disease progression could enrol for post-progression crossover, while patients in the ^177^Lu-DOTATATE arm who experienced disease progression could enrol for re-treatment.^4^

### Outcomes

The primary endpoint (progression-free survival) and key secondary endpoint (objective response rate) for NETTER-2, alongside other secondary endpoints (including safety), have already been reported.^4^ As reported previously, progression-free survival was defined as the time from randomisation to first-line progression or death from any cause and objective response rate was defined as the rate of best overall response of complete response or partial response; both were based on independent blinded central assessments per Response Evaluation Criteria in Solid Tumours, version 1·1.^4^^,^^8^

Preplanned subgroup analyses assessed the progression-free survival and objective response rate of ^177^Lu-DOTATATE *vs* control by NET grade (2 *vs* 3), site of origin (pancreas *vs* gastrointestinal [ie, all GEP-NET sites of origin except the pancreas]), and central baseline somatostatin receptor uptake score (3 [uptake greater than liver but lower than spleen] *vs* 4 [uptake greater than spleen]).

Post-hoc subgroup analyses further assessed the progression-free survival and objective response rate of ^177^Lu-DOTATATE *vs* control by NET grade plus site of origin, ie, in grade 2 pancreatic NET, grade 2 gastrointestinal NET, grade 3 pancreatic NET, and grade 3 gastrointestinal NET.

Post-hoc multivariate analyses assessed the treatment effect of ^177^Lu-DOTATATE after adjustment for various baseline covariates and evaluated baseline characteristics that may impact progression-free survival and objective response rate.

### Statistics

The sample size calculation (which was based on the primary endpoint of progression-free survival and did not take into account the key secondary endpoint of objective response rate) and statistical analyses for NETTER-2 have already been reported in the primary manuscript.^4^ For the present efficacy analyses, the full analysis set (which comprised all randomly assigned patients) was used, and analyses were performed using SAS 9·4 software (SAS Institute Inc., Cary, NC, USA). Subgroup and multivariable analyses were performed using standard SAS procedures (PROC) and did not involve bespoke programming, simulations, or non-standard code.

For the efficacy subgroup analyses, NET grade (2 *vs* 3) and site of origin (pancreas *vs* gastrointestinal) were based on data from electronic case report forms, and baseline somatostatin receptor uptake score (3 *vs* 4) was based on central review. These data were obtained at the time of the primary analysis (ie, at 101 progression-free survival events).^4^ There was no target sample size for subgroups, and no inferential statistics were planned or produced for subgroup analyses. The median progression-free survival and corresponding 95% CIs for each treatment arm were estimated using the Kaplan–Meier method. A stratified Cox model was used to obtain the hazard ratio (HR) for progression-free survival between treatment arms for the overall population; within each subgroup, an unstratified Cox model was used. Each covariate included in the Cox models was evaluated for the proportional hazard assumption using a Kolmogorov-type supremum test, with no evidence of violation of proportionality. Objective response rate was summarised using descriptive statistics by treatment arm, with two-sided exact 95% CIs. A stratified Cochran-Mantel-Haenszel method was used to obtain the odds ratio (OR) for objective response rate (response *vs* no response) between treatment arms for the overall population; within each subgroup, an unstratified Cochran-Mantel-Haenszel method was used. For stratified analyses, the randomisation stratification factors (NET grade 2 *vs* 3; tumour origin pancreas *vs* other) were used.

Post-hoc analyses using a multivariate Cox regression model (for progression-free survival) and a logistic regression model (for objective response rate: response *vs* no response) were performed, where baseline covariates included: age (<65 *vs* ≥65 years); sex (male *vs* female); primary NET site (pancreas *vs* small intestine *vs* other); NET grade (grade 2 *vs* grade 3 [Ki67 ≤30%] *vs* grade 3 high [Ki67 >30%]); Ki67 as a continuous variable; metastatic spread (liver metastases only [± lymph nodes] *vs* other metastases, ie, either no liver metastases or metastases in other organs in addition to the liver [± lymph nodes]); chromogranin A (≤2 × upper limit of normal *vs* > 2 × upper limit of normal); and central somatostatin receptor uptake score (3 *vs* 4). All clinically relevant covariates were included in the logistic regression model, without stepwise selection. Model assumptions (linearity, multicollinearity, and influential outliers) showed no material violations, apart from the expected moderate correlation between tumour grade and Ki67; both were retained for clinical relevance. For progression-free survival, HRs (ie, relative risk for one baseline covariate subgroup compared with another, or a unit change in a continuous variable), 95% CIs, and associated nominal p values were calculated from the multivariate Cox regression model. For objective response rate, ORs (ie, response *vs* no response for one baseline covariate subgroup compared with another, or a unit change in a continuous variable), 95% CIs, and associated nominal p values were calculated from the logistic regression model. In both the Cox and the logistic regression models, missing data were handled using the default SAS listwise deletion procedure, in which any observations with missing outcome or covariate values were excluded from the relevant analyses. A total of 24 patients had at least one missing covariate and were therefore excluded; as a result, 202 randomised patients were included in the final multivariable analyses.

### Role of the funding source

Advanced Accelerator Applications, a Novartis Company, designed and sponsored the trial. The funder was involved in the study design, data collection, data analysis, data interpretation, and writing of this manuscript. All authors had full access to the data in the study for interpretation.

## Results

Between Jan 22, 2020, and Oct 13, 2022, 261 patients were screened, with 35 (13%) being excluded due to screen failure. Further information on patient flow is provided in Figure S1. Overall, 226/261 (87%) patients were randomised: 151/226 (67%) to the ^177^Lu-DOTATATE arm and 75/226 (33%) to the control arm.^4^

Baseline demographics and disease characteristics were generally well balanced between treatment arms and have been previously reported for the full NETTER-2 study population (Table 1).^4^ Overall, of the 226 randomised patients, 121 (54%) patients were male, and 165 (73%) were White.^4^ The primary tumour site was the pancreas in 123 (54%) patients, while 103 (46%) patients had non-pancreatic (ie, gastrointestinal) NETs, including 66 (29%) patients with small intestine NETs. Looking at NET grade, 147 (65%) patients and 79 (35%) patients had grade 2 and 3 NETs, respectively. With regards to NET grade and primary tumour site, 76 (34%) patients had grade 2 pancreatic NETs, 47 (21%) had grade 3 pancreatic NETs, 71 (31%) had grade 2 gastrointestinal NETs, and 32 (14%) had grade 3 gastrointestinal NETs. In terms of somatostatin receptor uptake score, 34 (15%) patients had a score of 3 (ie, uptake greater than liver but lower than spleen) and 185 (82%) had a score of 4 (ie, uptake greater than spleen).

**Table 1:** Baseline disease characteristics.

	^177^Lu-DOTATATE (n = 151)	Control (n = 75)	All patients (N = 226)
Age (years)	61 (51–72)	60 (51–69)	61 (51–70)
Sex			
Male	81 (53·6)	40 (53·3)	121 (53·5)
Female	70 (46·4)	35 (46·7)	105 (46·5)
Primary tumour site of origin (GEP-NET)			
Pancreas	82 (54·3)	41 (54·7)	123 (54·4)
Other^a^	69 (45·7)	34 (45·3)	103 (45·6)
Small intestine	45 (29·8)	21 (28·0)	66 (29·2)
NET grade			
Grade 2	99 (65·6)	48 (64·0)	147 (65·0)
Ki67 index (%)	13·0 (10–17)	13·5 (10–15)	13·0 (10–16)
Grade 3, n (%)	52 (34·4)	27 (36·0)	79 (35·0)
Ki67 index (%)	30·0 (24–37)	30·0 (25–40)	30·0 (25–38)
Ki67 index (%)	17 (12–25)	16 (12–25)	16 (12–25)
Primary tumour site of origin by grade			
Pancreas, grade 2	50 (33·1)	26 (34·7)	76 (33·6)
Other, grade 2	49 (32·5)	22 (29·3)	71 (31·4)
Pancreas, grade 3	32 (21·2)	15 (20·0)	47 (20·8)
Other, grade 3	20 (13·2)	12 (16·0)	32 (14·2)
Metastases in liver only^b^			
Yes	65 (43·0)	38 (50·7)	103 (45·6)
No	85 (56·3)	36 (48·0)	121 (53·5)
Chromogranin A			
≤2 × ULN	43 (28·5)	24 (32·0)	67 (29·6)
>2 × ULN	100 (66·2)	44 (58·7)	144 (63·7)
Not reported	8 (5·3)	7 (9·3)	15 (6·6)
Central baseline somatostatin receptor tumour uptake score			
3	24 (15·9)	10 (13·3)	34 (15·0)
4	123 (81·5)	62 (82·7)	185 (81·9)

Data are median (IQR) or n (%). Data for the following characteristics are also reported in Singh S et al. (2024): age; sex; primary tumour site of origin (GEP-NET), pancreas; primary tumour site of origin (GEP-NET), small intestine; NET grade, Grade 2; NET grade, Grade 3; Ki67 index.^4^ GEP = gastroenteropancreatic. IQR = interquartile range. NET = neuroendocrine tumour. ULN = upper limit of normal.

a For primary tumour site of origin (GEP-NET), the ‘Other’ category for the overall population included small intestine (as reported in the table) plus the following additional sites (not shown in the table): rectum, stomach, gastrointestinal, ascending colon, midgut, gall bladder, hindgut, likely midgut, papilla of Vater, probable intestinal origin, and sigmoid colon.

b Two patients had no metastatic sites.

**Fig. 1:**
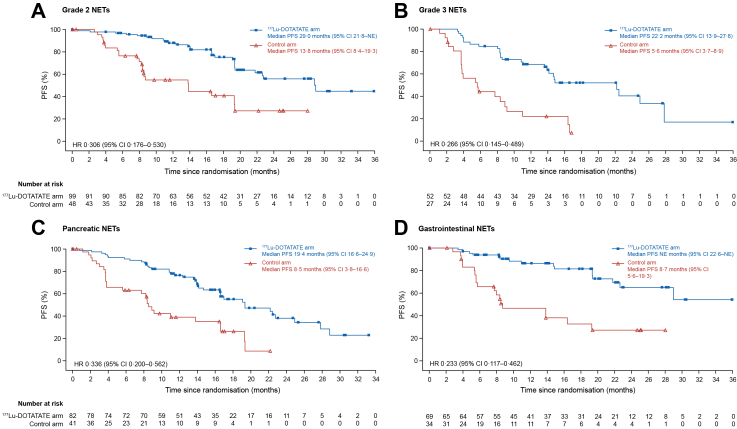
**Progression-free survival (preplanned subgroup analysis) among patients with grade 2 NETs (A), grade 3 NETs (B), pancreatic NETs (C), and gastrointestinal NETs (D)**. Figure shows progression-free survival in the full analysis set (based on central review using Response Evaluation Criteria in Solid Tumours, version 1·1 criteria) by subgroups of interest. Median progression-free survival and corresponding 95% CI was estimated using the Kaplan–Meier method. HRs and corresponding 95% CIs are for the ^177^Lu-DOTATATE arm *vs* the control arm and were based on an unstratified Cox model. HR = hazard ratio. NE = not estimable. NET = neuroendocrine tumour. PFS = progression-free survival.

For the preplanned subgroup analyses of progression-free survival, a clinical benefit in favour of ^177^Lu-DOTATATE *vs* control was evident regardless of NET grade or site of origin. Among patients receiving ^177^Lu-DOTATATE *vs* control, the HR (95% CI) for progression-free survival was 0·306 (0·176–0·530) for those with grade 2 NETs, 0·266 (0·145–0·489) for grade 3 NETs, 0·336 (0·200–0·562) for pancreatic NETs, and 0·233 (0·117–0·462) for gastrointestinal NETs (Fig. 1). In the ^177^Lu-DOTATATE arm, the median progression-free survival was shorter for patients with grade 3 (22·2 months) *vs* grade 2 (29·0 months) NETs, and it was 19·4 months for patients with pancreatic NETs *vs* not estimable for patients with gastrointestinal NETs (Fig. 1). In further post-hoc analyses, we tested progression-free survival by NET grade plus site of origin and confirmed a consistent benefit of ^177^Lu-DOTATATE *vs* control in all four subgroups (ie, grade 2 pancreatic NET, grade 2 gastrointestinal NET, grade 3 pancreatic NET, and grade 3 gastrointestinal NET; appendix, Table S2).

Similarly, the preplanned subgroup analysis of objective response rate indicated that the treatment benefit of ^177^Lu-DOTATATE *vs* control was maintained regardless of NET grade or site of origin (Table 2). Treatment with ^177^Lu-DOTATATE was associated with a notable benefit in response rate compared with control among patients with grade 2 NETs, grade 3 NETs, pancreatic NETs, and gastrointestinal NETs (Table 2). In the ^177^Lu-DOTATATE arm, the objective response rate was high for patients with grade 3 (48% [25/52]) and grade 2 (40% [40/99]) NETs; the objective response rate was higher for patients with pancreatic NETs (51% [42/82]) *vs* those with gastrointestinal NETs (33% [23/69]). In subsequent post-hoc analyses, objective response rate results did not substantially change when analysed by NET grade plus site of origin (Appendix, Table S3).

**Table 2:** Objective response rate (preplanned subgroup analysis).

	Complete response, n (%)	Partial response, n (%)	Total responders/N	Objective response rate (95% CI)	OR (95% CI)
NET grade: grade 2					
^177^Lu-DOTATATE	6 (6·1)	34 (34·3)	40/99	40·4 (30·7–50·7)	5·83 (2·12–16·00)
Control	0	5 (10·4)	5/48	10·4 (3·5–22·7)	
NET grade: grade 3					
^177^Lu-DOTATATE	2 (3·8)	23 (44·2)	25/52	48·1 (34·0–62·4)	11·57 (2·48–53·97)
Control	0	2 (7·4)	2/27	7·4 (0·9–24·3)	
NET site of origin: pancreas					
^177^Lu-DOTATATE	5 (6·1)	37 (45·1)	42/82	51·2 (39·9–62·4)	7·56 (2·70–21·19)
Control	0	5 (12·2)	5/41	12·2 (4·1–26·2)	
NET site of origin: gastrointestinal					
^177^Lu-DOTATATE	3 (4·3)	20 (29·0)	23/69	33·3 (22·4–45·7)	8·00 (1·76–36·35)
Control	0	2 (5·9)	2/34	5·9 (0·7–19·7)	

The table shows best overall response for patients in the full analysis set (based on central review using Response Evaluation Criteria in Solid Tumours, version 1·1 criteria) by subgroups of interest. For objective response rate, two-sided exact 95% CIs are presented. ORs are for the ^177^Lu-DOTATATE arm *vs* the control arm and were based on unstratified Cochran-Mantel-Haenszel method. CI = confidence interval. NET = neuroendocrine tumour. OR = odds ratio.

In the preplanned subgroup analyses of progression-free survival and objective response rate by central baseline somatostatin receptor uptake scores (Table 3), the findings were consistent with those from the overall primary analysis. The HR for progression-free survival with ^177^Lu-DOTATATE *vs* control was 0·306 (95% CI 0·105–0·895) among patients with an uptake score of 3 and 0·300 (95% CI 0·192–0·468) among patients with a score of 4. In the ^177^Lu-DOTATATE arm, the median progression-free survival was not estimable in patients with an uptake score of 3 and 22·8 months in those with a score of 4. Objective response rate in the ^177^Lu-DOTATATE arm was 46% (11/24) among those with an uptake score of 3 and 42% (52/123) for those with a score of 4. There was an increased likelihood of response with ^177^Lu-DOTATATE *vs* control among patients with an uptake score of 4 (OR 5·75 [95% CI 2·43–13·65]). While the same trend was observed among patients with an uptake score of 3, the OR was not estimable as no response was observed in the control arm.

**Table 3:** Progression-free survival and objective response rate by central baseline somatostatin receptor uptake score (preplanned subgroup analysis).

	Somatostatin receptor uptake score 3		Somatostatin receptor uptake score 4	
	^177^Lu-DOTATATE	Control	^177^Lu-DOTATATE	Control
Progression-free survival				
Event/N (%)	9/24 (37·5)	6/10 (60·0)	46/123 (37·4)	38/62 (61·3)
Median progression-free survival (95% CI), months	NE (13·6–NE)	8·0 (3·7–NE)	22·8 (19·4–29·0)	8·7 (7·7–16·4)
HR (95% CI)	0·31 (0·10–0·89)		0·30 (0·19–0·47)	
Objective response rate				
Complete response, n (%)	2 (8·3)	0	6 (4·9)	0
Partial response, n (%)	9 (37·5)	0	46 (37·4)	7 (11·3)
Total responders/N (%)	11/24 (45·8)	0/10 (0·0)	52/123 (42·3)	7/62 (11·3)
Objective response rate (95% CI)	45·8 (25·6–67·2)	0·0 (0·0–30·8)	42·3 (33·4–51·5)	11·3 (4·7–21·9)
OR (95% CI)	NE (NE–NE)		5·75 (2·43–13·65)	

The table shows progression-free survival and best overall response for patients in the full analysis set (based on central review using Response Evaluation Criteria in Solid Tumours, version 1·1 criteria) by subgroups based on central baseline somatostatin receptor uptake score. For progression-free survival, the median progression-free survival and corresponding 95% CIs were estimated using the Kaplan–Meier method. HRs and corresponding 95% CIs are for the ^177^Lu-DOTATATE arm *vs* the control arm and were based on an unstratified Cox model. For objective response rate, two-sided exact 95% CIs are presented. ORs are for the ^177^Lu-DOTATATE arm *vs* the control arm and were based on unstratified Cochran-Mantel-Haenszel method. CI = confidence interval. HR = hazard ratio. NE = not estimable. OR = odds ratio.

**Fig. 2:**
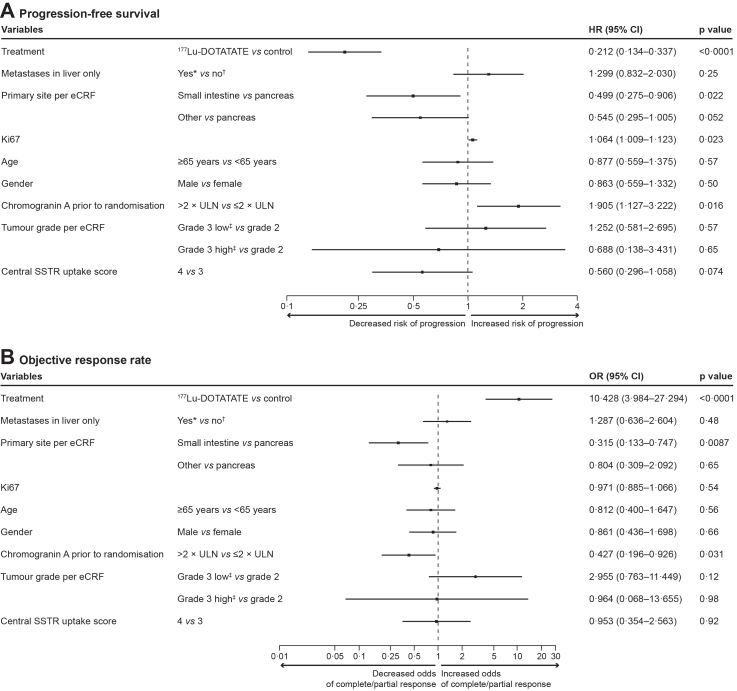
**Post-hoc multivariate analysis of baseline characteristics that may impact progression-free survival (A) and objective response rate (B)**. Figure shows results from a multivariate Cox regression model of progression-free survival (months) and a logistic regression model of best overall response as complete/partial response (yes/no), with treatment and other key baseline variables as covariates, based on the full analysis set. ∗Patients who had liver metastases only ± lymph nodes involved. ^†^Patients who had no liver metastases or had both liver and other organ metastases involved. ^‡^Grade 3 low: Ki67 ≤30%; grade 3 high: Ki67 >30%. eCRF = electronic case report form. HR = hazard ratio. NET = neuroendocrine tumour. OR = odds ratio. SSTR = somatostatin receptor. ULN = upper limit of normal.

Using post-hoc multivariate regression models, treatment effects (^177^Lu-DOTATATE *vs* control) on progression-free survival and objective response rate were minimally affected when adjusted for baseline covariates based on age, sex, primary NET site of origin, NET grade, Ki67 (continuous), metastatic spread, chromogranin A, and central somatostatin receptor uptake score (Fig. 2). The HR for progression-free survival was 0·212 (95% CI 0·134–0·337; p < 0·0001) after adjusting for baseline covariates, compared with 0·276 (0·182–0·418; p < 0·0001) in the unadjusted primary analysis. The OR for objective response rate was 10·43 (95% CI 3·98–27·29; p < 0·0001) after adjusting for baseline covariates, compared with 7·81 (3·32–18·40; p < 0·0001) in the unadjusted primary analysis. Post-hoc multivariate analyses with various baseline covariates showed that ‘treatment’ had the greatest influence on progression-free survival and objective response rate among all other parameters, and few parameters had a notable influence on progression-free survival and objective response rate. Of note, disease spread had a limited impact on progression-free survival and objective response rate (Fig. 2).

## Discussion

Preplanned subgroup analyses from the randomised, phase 3 NETTER-2 trial found that the treatment benefit of ^177^Lu-DOTATATE plus octreotide LAR 30 mg *vs* octreotide LAR 60 mg (high-dose) in terms of progression-free survival and objective response rate was consistent regardless of NET grade (2 *vs* 3) and origin (pancreas *vs* gastrointestinal). This benefit was also seen in patients with somatostatin receptor uptake scores of both 3 and 4. Additionally, when the original progression-free survival and objective response rate analyses were adjusted for baseline covariates, consistent benefits were observed with first-line ^177^Lu-DOTATATE *vs* high-dose octreotide LAR. These findings are important given that there is a paucity of robust data and evidence to support first-line treatment options for patients with higher grade 2 and grade 3, well-differentiated GEP-NETs.^9^^,^^10^ Potential treatment options that may be considered for subsets of this population include somatostatin analogues, chemotherapy, and targeted treatments such as everolimus and sunitinib, although ^177^Lu-DOTATATE has also been investigated for progressive higher grade 2–3 NETs.^9^, ^10^, ^11^ However, there is currently no defined standard of care in patients with newly diagnosed higher grade 2 and grade 3 advanced GEP-NETs.^9^^,^^10^ NETTER-2 assessed ^177^Lu-DOTATATE plus octreotide LAR 30 mg *vs* high-dose octreotide LAR 60 mg in patients with newly diagnosed, advanced, well-differentiated, higher grade 2 and grade 3, somatostatin receptor-positive GEP-NETs, excluding those for whom chemotherapy was considered more appropriate than the study treatments.^4^ The findings of our additional NETTER-2 efficacy analyses build upon the primary results, providing additional evidence to support use of ^177^Lu-DOTATATE as a first-line treatment for this heterogeneous patient population, regardless of NET grade (higher grade 2 *vs* grade 3), site of origin (pancreas *vs* gastrointestinal), or somatostatin receptor uptake score (3 *vs* 4), thus helping to fill the evidence gap in clinical treatment guidelines for grade 2–3 GEP-NETs.

The clinical benefit observed with ^177^Lu-DOTATATE *vs* high-dose octreotide LAR in the preplanned subgroup analyses of NET grade (2 *vs* 3) and origin (pancreas *vs* gastrointestinal) was also maintained in the NET grade plus site of origin subgroup analysis. Of note, in the ^177^Lu-DOTATATE arm, median progression-free survival was 29·0 and 22·2 months, and objective response rate was 40% (40/99) and 48% (25/52), for grade 2 and 3 NETs, respectively. This is an important finding, given that there are limited first-line treatment options for patients with higher grade 2–3 GEP-NETs. Response rates with radioligand therapy appear to be higher compared with other systemic therapies,^4^^,^^11^ suggesting radioligand therapy is an option for those with a high disease burden and may provide symptomatic relief while being generally well tolerated.^12^ Further, high tumour burden is associated with worse prognosis^13^; therefore, it is expected that radioligand therapy may improve the overall prognosis, given that high response rates are associated with durable response.^4^ In terms of NET site of origin, in the ^177^Lu-DOTATATE arm, median progression-free survival was 19·4 months and not estimable, and objective response rate was 51% (42/82) and 33% (23/69), for pancreatic and gastrointestinal NETs, respectively. Our progression-free survival and objective response rate findings for the subgroup of patients with pancreatic NETs were consistent with those from the OCLURANDOM study, in which patients with progressive pancreatic NETs who received ^177^Lu-DOTATATE had a median progression-free survival of 20·7 months and an objective response rate of 63%.^14^ The difference in objective response rate that we observed between pancreatic NETs and other gastrointestinal NETs may be due to differences in tumour-absorbed dose by primary tumour site or the natural history of the disease. There have been several studies investigating the relationship between tumour-absorbed dose and response to radioligand therapy.^15^^,^^16^ For example, a retrospective study by Jahn U et al. identified a significant correlation between absorbed dose and tumour shrinkage in patients with pancreatic NETs or small intestinal NETs treated with ^177^Lu-DOTATATE, particularly in those with pancreatic NETs.^16^ Interestingly, among patients receiving ^177^Lu-DOTATATE, those with more aggressive NETs had a higher objective response rate (eg, grade 3 NETs and pancreatic NETs) and in some cases had a shorter median progression-free survival (eg, grade 3 NETs). The highest objective response rate and the lowest estimable median progression-free survival were observed among the subgroup of patients with grade 3 pancreatic NETs (Appendix, Tables S2 and S3). This is an important consideration when selecting a first-line treatment option for patients with aggressive NETs, for whom treatment goals may focus on achieving an initial response and reducing tumour burden. The findings from NETTER-2 suggest that, for some patient subgroups, ^177^Lu-DOTATATE can achieve an objective response rate that is similar to chemotherapy^17^, ^18^, ^19^; this further supports the adoption of ^177^Lu-DOTATATE as a first-line treatment option. The high objective response rate may be clinically meaningful for functioning pancreatic NETs, since symptom relief may be achieved with pronounced downstaging of tumour burden.^20^ Furthermore, in bulky disease it is crucial to achieve objective response rate to prevent complications and long-term sequelae. In the NEOLUPANET study, patients with non-functioning pancreatic NETs who were candidates for surgery received neoadjuvant ^177^Lu-DOTATATE treatment.^21^ Most patients achieved a partial response (58% [18/31]), and severe complications following surgery were limited.^21^ These findings suggest that in clinical scenarios in which achieving a rapid and meaningful tumour response is crucial—for example, due to high tumour burden, symptoms, or impending organ dysfunction—^177^Lu-DOTATATE could be considered a legitimate alternative to chemotherapy, with a comparable objective response rate and manageable safety profile.

It is difficult to directly compare our findings with those of chemotherapy trials. While NETTER-2 enrolled patients with newly diagnosed, higher grade 2 or grade 3 GEP-NETs with high somatostatin receptor expression (mostly with the highest somatostatin receptor uptake score) and no risk of rapid deterioration,^4^ trials investigating chemotherapy agents often have less-selective inclusion criteria (ie, they do not require patients to be somatostatin receptor-positive with no risk of rapid deterioration).^19^^,^^22^ Our subgroup analyses of efficacy by somatostatin receptor uptake score confirm that somatostatin receptor imaging is not only diagnostic but may also be predictive, helping stratify patients likely to benefit from radioligand therapy. Thus, it is crucial to select patients appropriately for radioligand therapy, by carefully considering somatostatin receptor imaging to identify those with lower or highly heterogeneous somatostatin receptor uptake who would likely not benefit from such treatment. The strength of NETTER-2 is that patients had been carefully selected based on homogenous and strong somatostatin receptor expression, and patients who were instead considered candidates for chemotherapy based on physicians’ overall assessment were excluded. In addition, NETTER-2 included patients with a range of tumour origin sites, including pancreatic NETs in approximately 54% (123/226) of patients, which expands the applicability of the findings to a broad GEP-NET population. However, our findings should be interpreted within the context of the NETTER-2 study population and should not be extrapolated to patient populations that differ substantially from those enrolled, such as those with somatostatin receptor-heterogeneous or somatostatin receptor-negative grade 3 NETs, those with high-grade tumours displaying rapid clinical decline, or those who may be considered as more appropriate candidates for first-line chemotherapy. Importantly, treatment decisions for patients with GEP-NETs should be individualised and managed according to their overall clinical profile. Taken together, the NETTER-2 findings indicate that ideal candidates for first-line ^177^Lu-DOTATATE plus octreotide LAR 30 mg therapy are patients with newly diagnosed, advanced, well-differentiated, higher grade 2–3 (Ki67 ≥10–≤55%), somatostatin receptor-positive GEP-NETs for whom chemotherapy is not considered the most appropriate option. Treatment benefit was observed regardless of NET grade (2 *vs* 3), tumour origin (pancreas *vs* gastrointestinal), and baseline somatostatin receptor uptake (score 3 *vs* 4), although the magnitude of benefit differed between subgroups. Accordingly, although all assessed subgroups appear suitable for ^177^Lu-DOTATATE, treatment goals should guide clinical decision-making. For example, response-oriented goals (tumour shrinkage/objective response rate) may be particularly relevant for patients with more aggressive disease, such as those with pancreatic NETs. We expect that, should healthcare professionals identify patients with disease characteristics that match those of the NETTER-2 patient population/subgroups during routine practice, these patients would benefit from treatment with ^177^Lu-DOTATATE to a similar extent as was observed in NETTER-2 and its relevant subgroup analyses. Overall, our findings suggest that molecular imaging should be a gatekeeper for daily therapeutic decision-making. If appropriate based on molecular imaging and biological behaviour, clinicians, including nuclear medicine physicians, should consider earlier integration of radioligand therapy in the treatment pathway.

Before NETTER-2, there was little evidence available to aid treatment decisions in patients with higher grade 2 and grade 3 GEP-NETs; because of this, outcomes were generally poor in this patient population.^4^ For advanced grade 1–2 (ie, lower-grade with Ki67 <10%) GEP-NETs, somatostatin analogues are the first-line treatment,^9^^,^^10^^,^^23^ based on the phase 3 PROMID and CLARINET studies.^24^^,^^25^ These trials recruited patients with low-grade (primarily grade 1) tumours, which are less aggressive and are associated with a better prognosis. Another treatment option in the lower-grade setting is everolimus, which is recommended for progressive grade 1–2 pancreatic NETs,^9^^,^^23^ although data from the COMPETE study suggest that radioligand therapy (^177^Lu-edotreotide) improves progression-free survival to a greater extent than everolimus in patients with progressive grade 1–2 GEP-NETs and has a favourable safety profile.^26^ More recently, cabozantinib has emerged as an option for pancreatic or extra-pancreatic NETs regardless of grade based on the phase 3 CABINET trial^27^; however, cabozantinib is only indicated for previously treated NETs and has not been assessed as a first-line treatment option. While chemotherapy may be considered as a treatment option for patients with higher grade 2 and grade 3 GEP-NETs, it lacks prospective data in this patient population. Multicentre, retrospective studies have shown response rates between 27% and 51% with temozolomide regimens in patients with grade 3 NETs.^17^^,^^18^ Prospective chemotherapy data are available in patients with lower-grade NETs. For example, capecitabine plus temozolomide (CAPTEM) showed a response rate of 40% in patients with advanced, low- or intermediate-grade pancreatic NETs (who may have received prior treatment with somatostatin analogues, everolimus, and/or sunitinib) in the prospective, randomised, phase 2 ECOG-ACRIN E2211 study.^19^ Additionally, the phase 3 SEQTOR trial in patients with low- or intermediate-grade advanced pancreatic NETs showed that the approved chemotherapy streptozocin^28^ plus 5-fluorouracil was more effective than everolimus at obtaining a treatment response, regardless of sequence order.^22^^,^^29^ However, ECOG-ACRIN E2211 and SEQTOR do not inform on the wider high-grade GEP-NET population. Indeed, patients enrolled in NETTER-2 had newly diagnosed, advanced, grade 2 or 3 GEP-NETs, and thus would not have been eligible for ECOG-ACRIN E2211 or SEQTOR.

To date, there has been no prospective, randomised study providing evidence of chemotherapy efficacy in the first-line treatment of advanced grade 2 and 3 GEP-NETs.^10^ The decision to use chemotherapy as a first-line treatment is based on multiple factors, including tumour growth, Ki67 index, lack of somatostatin receptor expression, and others.^9^ However, it should be noted that the NETTER-2 population excluded patients for whom chemotherapy was considered more appropriate than the study treatments. This decision was made by the individual study investigators based on patient and disease characteristics. Nevertheless, for patients for whom ^177^Lu-DOTATATE is deemed the most appropriate treatment, the present efficacy subgroup analyses provide important data on the clinical benefit of ^177^Lu-DOTATATE across various subpopulations of patients; this will help healthcare professionals to make more informed treatment decisions and improve patient outcomes.

Limitations of NETTER-2 have been previously reported.^4^ Focusing specifically on the analyses reported in this Article, it should be noted that subgroup analyses were based on a limited sample size, especially for subgroups of NET grade plus site of origin. Additionally, subgroup analyses were descriptive in nature and no formal statistical tests were performed. Although [^18^F]fluorodeoxyglucose (^18^F-FDG) positron emission tomography (PET)/computed tomography (CT) imaging can play an important role in clinical decision-making for patients with advanced GEP-NETs, it is not yet considered part of routine radioligand therapy evaluations in many centres, and is not included in most ongoing clinical trials.^30^^,^^31^ Indeed, ^18^F-FDG-PET/CT imaging was not mandated in the NETTER-2 study protocol. Consequently, baseline ^18^F-FDG PET/CT imaging data are not available and subgroup analyses according to ^18^F-FDG PET/CT avidity could not be performed.

As noted previously,^4^ future research should explore the effectiveness of ^177^Lu-DOTATATE relative to other available therapies, optimal treatment sequencing for subsequent therapies, and potential access issues. Regarding the effectiveness of ^177^Lu-DOTATATE relative to other available therapies, an ongoing randomised phase 3 study (COMPOSE) is exploring the efficacy of another radioligand therapy (^177^Lu-edotreotide) relative to physicians’ choice (chemotherapy or everolimus) in patients with well-differentiated, higher-proliferative (Ki67 15–55%), somatostatin receptor-positive GEP-NETs.^32^ The results from COMPOSE may help support the effectiveness of radioligand therapy relative to other therapies and are expected to inform the optimal treatment options for this patient population. Regarding treatment sequencing, subsequent lines of treatment can be tailored based on tumour origin; for example, potential options for gastrointestinal NETs include everolimus, and potential options for pancreatic NETs include capecitabine plus temozolomide, everolimus, or sunitinib.^23^ However, further research is required to determine the optimal sequencing of such therapies.

Overall, our results suggest that first-line ^177^Lu-DOTATATE should be considered as a standard of care for patients with advanced, well-differentiated, higher grade 2 or 3 (Ki67 ≥10% and ≤55%), somatostatin receptor-positive GEP-NETs, and for whom chemotherapy is not considered the most appropriate treatment option. ^177^Lu-DOTATATE was shown to be efficacious in this patient population, with progression-free disease lasting nearly two years. Importantly, our subgroup analyses indicate that the efficacy benefit of ^177^Lu-DOTATATE is consistent regardless of NET grade (2 *vs* 3), site of origin (pancreas *vs* gastrointestinal), or baseline somatostatin receptor uptake score (3 *vs* 4). In particular, the high objective response rate observed in patients with more aggressive tumours indicates that ^177^Lu-DOTATATE—which has more robust prospective data to support its use a first-line treatment for advanced grade 2 and 3 GEP-NETs than chemotherapy—has the potential to be used as an alternative to chemotherapy when a reduction in tumour burden is a meaningful and achievable goal. Overall, our findings supplement the primary outcomes of NETTER-2 and provide valuable insights to fill the evidence gap in clinical treatment guidelines for grade 2–3 GEP-NETs.

## Contributors

MP, PLK, IF, YZ, WWdH, and SS were involved in study design. All authors (DF, MP, KH, PLK, SM, DH, BC, JC, AG-B, ST, SL, D-YO, CY, SF, TRH, MS, LG, ED, AF, IF, YZ, WWdH, and SS) were involved in data collection. All authors (DF, MP, KH, PLK, SM, DH, BC, JC, AG-B, ST, SL, D-YO, CY, SF, TRH, MS, LG, ED, AF, IF, YZ, WWdH, and SS) had access to the data and contributed to the analysis or interpretation of the data. DF, MP, KH, SM, DH, BC, JC, AG-B, D-YO, IF, WWdH, and SS vouch for the accuracy and integrity of the data. All authors (DF, MP, KH, PLK, SM, DH, BC, JC, AG-B, ST, SL, D-YO, CY, SF, TRH, MS, LG, ED, AF, IF, YZ, WWdH, and SS) were involved in the writing, reviewing, and amending of the manuscript with the assistance of a medical writer funded by the sponsor. All authors (DF, MP, KH, PLK, SM, DH, BC, JC, AG-B, ST, SL, D-YO, CY, SF, TRH, MS, LG, ED, AF, IF, YZ, WWdH, and SS) approved the final draft and had final responsibility for the decision to submit for publication.

## Data sharing statement

Novartis is committed to sharing with qualified external researchers, access to patient-level data and supporting clinical documents from eligible studies. These requests are reviewed and approved by an independent review panel on the basis of scientific merit. All data provided are anonymised to respect the privacy of patients who have participated in the trial in line with applicable laws and regulations. This trial data availability is according to the criteria and process described on www.clinicalstudydatarequest.com.

## Declaration of interests

DF reports grants or contracts from Camurus, honoraria from Advanced Accelerator Applications (a Novartis company) and Recordati Rare Diseases, advisory board participation for Recordati Rare Diseases, support for attending meetings from Camurus and Recordati Rare Diseases, and is the president elect of the Italian Society of Endocrinology. MP reports consulting fees from Advanced Accelerator Applications (a Novartis company), Boehringer Ingelheim, Ipsen, and ITM, honoraria from Advanced Accelerator Applications (a Novartis company), Boehringer Ingelheim, Ipsen, Lilly, Medscape, Merck, MSD, Novartis, Recordati, Sanofi, Serb, and Tairix, travel support from ENETS, Ipsen, and Recordati, safety monitoring committee membership for Advanced Accelerator Applications (a Novartis company), Crinetics, and the Gustave Roussy Institute (University Paris, Villejuif), and unpaid roles as AIO NET group leader of the German Cancer Foundation, ENETS advisory board member, ESMO education committee member, and as advisory board member for Netzwerk NET and INCA. KH reports grants or contracts from Novartis and SOFIE Biosciences, consulting fees from Advanced Accelerator Applications (a Novartis company), Amgen, AstraZeneca, Bain Capital, Bayer, Boston Scientific, Convergent, Curium, Debiopharm, EcoR1, Fusion, GE Healthcare, Immedica, Isotopen Technologien München, Janssen, Merck, Molecular Partners, NVision, Pfizer, POINT Biopharma, Radiopharm Theranostics, Rhine Pharma, Siemens Healthineers, SOFIE Biosciences, Telix, Theragnostics, and Y-mAbs, honoraria from PeerVoice, meeting support from Janssen, advisory board participation for Fusion and GE Healthcare, and stock or stock options for AdvanCell, Aktis Oncology, Convergent, NVision, Pharma15, and SOFIE Biosciences. PLK reports grants or contracts from Crinetics, Novartis, and RayzeBio. SM reports honoraria from Ipsen and Novartis Oncology, and support for attending meetings from Novartis Oncology. DH reports support for the present work from Novartis, grants or contracts from Camurus, Chimeric Therapeutics, Crinetics Pharmaceuticals, Genentech/Roche, ITM, Novartis, RayzeBio, and Thermo Fisher Scientific, consulting fees from AbbVie, Amryt, Boehringer Ingelheim, Camurus, Chimeric Therapeutics, Crinetics, Exelixis, HarbourBioMed, Harpoon Therapeutics, Ipsen, ITM, Lantheus, Novartis, Sanofi Aventis, TerSera, and Wren Laboratories, and participation on a Data Safety Monitoring Board for Alphamedix. BC reports advisory board participation for Advanced Accelerator Applications (a Novartis company) and Lantheus. JC reports grants or contracts from Advanced Accelerator Applications (a Novartis company), Bayer, Eisai, Esteve, Exelixis, Ipsen, Isotopen Technologien, Lilly, Merck Serono, Novartis, Pfizer, Roche/Genentech, and Sanofi, honoraria from Bayer, Eisai, Esteve, Hutchison MediPharma, Ipsen, Isotopen Technologien, Lilly, Merck Serono, Novartis, Pfizer, and Sanofi, and support for attending meetings from Eisai, Ipsen, and Pfizer. AG-B reports advisory/consulting fees from Advanced Accelerator Applications (a Novartis company), Bayer, and Sanofi, and speaker fees and meeting support from Novartis. ST reports consulting fees from Boehringer Ingelheim, Camurus, Deciphera, Esteve, Gentili, Ipsen, and Novartis. SL reports honoraria from, and advisory board participation for, Advanced Accelerator Applications (a Novartis company). D-YO reports grants or contracts from Array, AstraZeneca, BeiGene, Eli Lilly, Handok, Hanmi, MSD, Novartis, and Servier, and advisory board participation for AbbVie, Alligator Bioscience AB, ASLAN, Arcus Biosciences, Astellas, AstraZeneca, Basilea, Bayer, BeiGene, BMS/Celgene, Daewoong, Eutilex, Genentech/Roche, Halozyme, Hana Pharm, Hanmi, Idience, IQVIA, J-Pharma, LG Chem, Merck Serono, Mirati Therapeutics, Moderna, MSD, Novartis, Pfizer, Revolution Medicine, Taiho, Tallac Therapeutics, Turning Point, Yuhan, and Zymeworks. CY reports support for the present work from Novartis, grants or contracts from AstraZeneca, Bayer, Boehringer Ingelheim, Boryung, CKD Pharm, Ipsen, Lunit Inc, Ono Pharmaceuticals, and Servier, and honoraria from AstraZeneca, Autem Therapeutics, Bayer, Boehringer Ingelheim, Boryung, Bristol Myers Squibb, Celgene, Eisai, Elevar Therapeutics, HLB, Ipsen, MSD, Novartis, Qurient, Roche, and Servier. SF has nothing to declare. TRH reports grants from Advanced Accelerator Applications (a Novartis company), Camurus, Crinetics, ITM, Perspective Therapeutics, Rezolute, and Thermo Fisher Scientific, consulting fees from Abdera Therapeutics, Advanced Accelerator Applications (a Novartis company), Boehringer Ingelheim, Camurus, Crinetics, Curium, Exelixis, ITM, Perspective Therapeutics, Sanofi, and TerSera, honoraria from Banner MD Anderson, Binaytera Foundation, ISGIO Physicians’ Education Resource® LLC (PER®), and MD Education Ltd, and was president of NANETs from October 2023 to November 2024. MS has nothing to declare. LG reports honoraria from Advanced Accelerator Applications (a Novartis company). ED reports honoraria from GE, Ipsen, Janssen, and Novartis, support for attending meetings from GE and Novartis, advisory board participation for Ipsen and Novartis, and has an unpaid role as general secretary of the French Society of Nuclear Medicine. AF reports honoraria from GE Healthcare and Novartis, advisory board participation for Novartis, and support for meeting attendance from Bayer, GE Healthcare, and Novartis. IF and YZ report employment by Novartis and stock or stock options for Novartis. WWdH reports advisory board participation for Camurus, Ipsen, and Novartis, receiving travel grants from Recordati, and participation on data safety monitoring boards for trials sponsored by Erasmus MC and Rare Thyroid Therapeutics International AB. SS reports support for the present work from Advanced Accelerator Applications (a Novartis company), grants or contracts from Novartis, consulting fees from Camurus, Ipsen, and Novartis, and meeting attendance support from Ipsen and Novartis.

## Appendix A Supplementary data

Protocol
