# Supplementary material for: [177Lu]Lu-DOTA-TATE plus long-acting octreotide in patients with newly diagnosed, advanced, grade 2–3, gastroenteropancreatic neuroendocrine tumours: preplanned and post-hoc efficacy analyses from the randomised, phase 3 NETTER-2 trial
Source: eClinicalMedicine. 2026 Aug 7;98:104109. doi: 10.1016/j.eclinm.2026.104109 (PMC13476574; doi:10.1016/j.eclinm.2026.104109)
Supplement: Supplementary file 1 [file mmc1.pdf]

Advanced Accelerator Applications

Research and Development

LUTATHERA® (lutetium Lu 177 dotatate)

Clinical Trial Protocol CAAA601A22301

A phase III multi-center, randomized, open-label study to evaluate the efficacy and safety of Lutathera in patients with Grade 2 and Grade 3 advanced GEP-NET

Document type:	Amended Clinical Trial Protocol
EUDRACT number:	2019-001562-15
IND number	77219
Version number:	2.0
Clinical Trial Phase:	III
Release date:	05 October 2022

Property of Advanced Accelerator Applications
Confidential
May not be used, divulged, published, or otherwise disclosed
without the consent of Advanced Accelerator Applications

Table of contents

Table of contents 2
List of tables 5
List of figures 6
List of abbreviations 7
Glossary of terms 10
Protocol synopsis 12
Amendment rationale 27
1 Introduction 33
1.1 Background 33
1.2 Purpose 36
2 Objectives and endpoints 37
3 Study design 39
3.1 Screening Phase 40
3.2 Treatment Phase 40
3.3 Optional Treatment Extension Phase (cross-over) 41
3.4 Optional Re-Treatment Phase 41
3.5 Follow up Phase 42
4 Rationale 43
4.1 Rationale for study design 43
4.2 Rationale for dose/regimen and duration of treatment 44
4.3 Rationale for choice of control drug 45
4.4 Purpose and timing of interim analyses 46
4.5 Risks and benefits 46
5 Population 49
5.1 Inclusion criteria 49
5.2 Exclusion criteria 49
5.2.1 Eligibility Criteria for Optional Treatment Extension Phase (cross-over) 51
5.2.2 Eligibility Criteria for Optional Re-Treatment Phase 52
6 Treatment 54
6.1 Study treatments 54
6.1.1 Investigational and control drugs 54
6.2 Other treatment(s) 58
6.2.1 Rescue medication 58

6.2.2 Other anti-cancer treatments 58
6.2.3 Prohibited medication 58
6.2.4 SARS-CoV-2 vaccination 59
6.3 Subject numbering, treatment assignment, randomization 60
6.3.1 Subject numbering 60
6.3.2 Treatment assignment, randomization 60
6.4 Treatment blinding 60
6.5 Dose compliance, modification and discontinuation 60
6.5.1 Dose compliance 60
6.5.2 Dose modification 60
6.5.3 Dose discontinuation 66
6.6 Preparation and dispensation of Lutathera 66
6.6.1 Handling of study treatment and additional treatment 68
7 Informed consent procedures 70
8 Visit schedule and assessments 72
8.1 Demographics and Baseline Characteristics 75
8.1.1 Diagnosis and Extent of Cancer 75
8.1.2 Ki67 75
8.1.3 Prior Antineoplastic Medications / Radiotherapy / Surgery 75
8.1.4 SRI Tumor Uptake in Documented Lesions 75
8.2 Prior/Concomitant Medications 76
8.3 Efficacy Assessment 76
8.3.1 Progression Free Survival 76
8.3.2 Objective Response Rate (ORR), Duration of Response (DOR), Time to Second Progression (PFS2) 78
8.3.3 Overall Survival 78
8.4 Quality of Life 79
8.5 Safety and Tolerability 79
8.5.1 Adverse Events 79
8.5.2 Laboratory Assessments 79
8.5.3 Pregnancy Test 81
8.5.4 Cardiac Ejection Fraction 82
8.5.5 ECG 82
8.5.6 Physical Examination and Vital Signs 83
8.5.7 Karnofsky Performance Score 83
8.5.8 Ultrasonic examination of the gallbladder (Germany only) 83
8.5.9 SPECT/CT and whole body planar imaging (Germany only) 83

8.5.10 Assessments Schedule 84
8.6 Pharmacokinetics 91
8.6.1 Analytical method 91
8.7 Biomarkers 91
8.8 Imaging 92
9 Study discontinuation and completion 94
9.1 Discontinuation 94
9.1.1 Discontinuation of study treatment 94
9.1.2 Withdrawal of informed consent 95
9.1.3 Lost to follow-up 96
9.1.4 Early study termination by the sponsor 96
9.2 Study completion and post-study treatment 96
9.2.1 Post-trial access to Lutathera 98
10 Safety monitoring and reporting 99
10.1 Definition of adverse events and reporting requirements 99
10.1.1 Adverse events 99
10.1.2 Serious adverse events 102
10.1.3 Adverse Drug Reaction (ADR) 103
10.1.4 Suspected Unexpected Serious Adverse Reactions (SUSARs) 103
10.1.5 Adverse Events of Special Interest 103
10.1.6 SAE reporting 105
10.1.7 Pregnancy reporting 106
10.1.8 Reporting of study treatment errors including misuse/abuse 106
10.1.9 Annual Safety Report 106
10.2 Additional Safety Monitoring 107
10.2.1 Steering Committee 107
11 Data Collection and Database management 108
11.1 Data collection 108
11.2 Database management and quality control 108
11.3 Site monitoring 108
12 Data analysis and statistical methods 110
12.1 Analysis sets 110
12.2 Subject demographics and other baseline characteristics 110
12.3 Treatments 111
12.4 Analysis of the primary endpoint(s) 111
12.4.1 Definition of primary endpoint(s) 111
12.4.2 Statistical model, hypothesis, and method of analysis 111

12.4.3 Handling of missing values/censoring/discontinuations 112
12.4.4 Sensitivity and Supportive analyses.. 112
12.5 Analysis of secondary endpoints 113
12.5.1 Key secondary objectives 113
12.5.2 Other efficacy endpoints 114
12.5.3 Safety endpoints... 115
12.6 Analysis of exploratory endpoints 117
12.6.1 PFS2... 117
12.6.2 Patient reported outcomes not associated with key secondary endpoint118
12.6.3 Pharmacokinetics 118
12.6.4 Re-treatment with Lutathera 119
12.7 Interim analyses... 119
12.8 Sample size calculation 119
12.8.1 Primary endpoint(s) 119
13 Ethical considerations and administrative procedures 121
13.1 Regulatory and ethical compliance 121
13.2 Responsibilities of the investigator and IRB/IEC 121
13.3 Publication of study protocol and results 121
13.4 Quality Control and Quality Assurance 122
14 Protocol adherence 124
14.1 Protocol Amendments 125
15 References 126
16 Appendices.. 135
Appendix 1 – Precautions for Pregnancy 136
Appendix 2 – Lutathera Administration and Sterile Amino Acid Co-Infusion Scheme........ 138
Appendix 3 – Karnofsky Performance Scale 139
Appendix 4 – Radioprotection Precautions for Treated with Lutathera ^{177}Lu-DOTA^{0}-Tyr^{3}-Octreotate 140
Appendix 5 – Recommendations for Patients Treated with Lutathera................................. 141
Appendix 6 – Instructions for Shipment, Storage and Handling of ^{177}Lu-DOTA^{0}-Tyr^{3}-Octreotate (Lutathera) Solution for Infusion.. 145
Appendix 7 – National Cancer Institute Common Terminology Criteria for Adverse Events150
Appendix 8 – Determination of LUTATHERA Administered Radioactivity....................... 156

List of tables

Table 2-1. Study objectives and endpoints... 37
Table 6-2. Composition of the Lutathera® Drug Product (per vial).. 55

Table 6-3. 2.5% Lys-Arg sterile solution composition* 57
Table 6-4. Dose Modifications of Lutathera for Adverse Events 61
Table 6-5. Administration procedure of antiemetic, sterile amino acid solution and Lutathera68
Table 8-1. Centrally Performed Assessments.. 72
Table 8-2. Laboratory Assessments[1] 80
Table 8-3. Assessment Schedule Lutathera Arm 85
Table 8-4. Assessment Schedule Octreotide Long-Acting 60 mg Arm. 88
Table 8-5. Octreotide long-acting PK blood collection plan... 91
Table 10-1. Adverse events of special interest.. 104

List of figures

Figure 3-1. Study Design 39
Figure 6-1. Lutathera Arm: schedule for administration of Lutathera, 30 mg octreotide long-acting, and short acting octreotide sc[1] 67

List of abbreviations

^{90}Y	Yttrium-90
^{111}In	Indium-111
^{177}Lu	Lutetium-177
AAA	Advanced Accelerator Applications
ADR	Adverse Drug Reaction
AE	Adverse event
AESI	Adverse Event of Special Interest
ALAT/ALT	Alanine Aminotransferase
ALP	Alkaline Phosphatase
ASAT/AST	Aspartate Aminotransferase
BOR	Best Overall Response
BP	Blood Pressure
BUN	Blood Urea Nitrogen
BMI	Body Mass Index
BW	Body Weight
CgA	Chromogranin-A
COVID-19	Coronavirus disease 2019
CR	Complete Response
CRO	Clinical Research Organization
CT	Computerized Tomography
CTCAE	Common Terminology Criteria for Adverse Events
DCR	Disease Control Rate
DMT	Dose Modifying Toxicity
DOR	Duration of Response
DOTA	1,4,7,10-Tetraazacyclododecane-N,N',N'',N'''-tetraaacetic Acid
DOTATATE	DOTA-Tyr^{3}-octreotate
DOTATOC	DOTA-Tyr^{3}-octreotide
DTPA	Diethylene Triamine Pentaacetic Acid
EC	Ethics Committee
ECG	Electrocardiogram
E-CRF	Electronic Case Report Form
EDC	Electronic Data Capture
EMA	European Medicines Agency
EORTC	European Organization for Research and Treatment of Cancer
EOS	End of Study
EOT	End of Treatment
Erasmus MC	Erasmus Medical Centre, Rotterdam, NL
FAS	Full Analysis Set
FDA	Food and Drug Administration
fT4	Free Thyroxine
FSH	Follicle Stimulating Hormone
FUP	Follow Up
GGT or γ-GT	Gamma-Glutamyl Transferase
GBq	Giga Becquerel (Bq = unit of radioactivity)

GCP	Good Clinical Practice
GEP	Gastro-Entero-Pancreatic
GFR	Glomerular Filtration rate
GI	Gastrointestinal
GlycoHb	Glycosylated Haemoglobin (haemoglobin A1C)
GMP	Good Manufacturing Practice
Gy	Gray (unit of radiation exposure; equal to 100 rad)
H	Hours
Hb	Haemoglobin
IB	Investigator's Brochure
ICF	Informed Consent Form
ICH	International Conference of Harmonization
ICMJE	International Committee of Medical Journal Editors
ID	Identification (Number)
IEC	Independent Ethic Committee
irRC	Immune-related response criteria
I.M.	Intramuscular
IMP	Investigational Medicinal Product
IRC	Independent Review Committee
IRB	Institutional Review Board
ITT	Intention To Treat
I.V.	Intravenous
KPS	Karnofsky Performance Score
LAR	Long Acting Release
LDH	Lactic Dehydrogenase
LPF	Low-Power Field (microscopic exam)
MBq	Mega Becquerel (Bq = unit of radioactivity)
MCi	MilliCurie (unit of radioactivity; 1 mCi = 37 MBq)
MCV	Mean Corpuscular Volume (red blood cells)
MDS	Myelodysplastic Syndrome
MedDRA	Medical Dictionary for Regulatory Activities
Mg	Milligram(s)
Ml	Milliliter(s)
MM	Millimole
MRI	Magnetic Resonance Imaging
NaCl	Sodium Chloride
NCI	National Cancer Institute (USA)
NET	Neuroendocrine Tumor
NIH	National Institute of Health (USA)
NYHA	New York Heart Association
ORR	Objective Response Rate
OS	Overall Survival
PAS	Pharmacokinetic Analysis Set
PD	Progressive Disease or Pharmacodynamic(s)
PET	Positron Emission Tomography
PFS	Progression Free Survival
PFS2	Progression Free Survival (Second Progression)

PI	Principal Investigator
PLT	Platelets
PPS	Per Protocol Set
PK	Pharmacokinetics
PR	Partial Response
PRO	Patient Reported Outcome
PRRT	Peptide Receptor Radionuclide Therapy
QoL	Quality of Life
QC	Quality Control
QMS	Quality Management System
QP	Qualified Person
RBC	Red Blood Cells
RECIST	Response Evaluation Criteria in Solid Tumors
RVG	Radionuclide Ventriculography
SAE	Serious Adverse Event
SAF	Safety Set (SAF)
SAP	Statistical Analysis Plan
SARS-CoV-2	Severe acute respiratory syndrome coronavirus 2
S.C.	Subcutaneous
SD	Stable Disease
SOP	Standard Operating Procedures
SPECT	Single photon-emission computed tomography
Sstr2	Somatostatin Receptor Subtype 2
SRI	Somatostatin Receptor Imaging
SRS	Somatostatin Receptor Scintigraphy
SSAs	Somatostatin Analogs
SUSAR	Suspected Unexpected Serious Adverse Reactions
TTD	Time to deterioration
ULN	Upper Limit of Normal (according to local laboratory normal values)
WBC	White Blood Cells
WBPI	Whole body planar imaging
WHO	World Health Organization

Glossary of terms

Assessment	A procedure used to generate data required by the study.
Biologic Samples	A biological specimen including, for example, blood (plasma, serum), saliva, tissue, urine, stool, etc. taken from a study subject or study patient.
Control drug	A study treatment used as a comparator to reduce assessment bias, preserve blinding of investigational drug, assess internal study validity, and/or evaluate comparative effects of the investigational drug.
Dose level	The dose of drug given to the patient (total daily or weekly etc.)
Enrollment	Point/time of patient entry into the study; the point at which informed consent must be obtained (i.e. prior to starting any of the procedures described in the protocol).
GEP-NET	Gastroenteropancreatic neuroendocrine tumors a rare type of tumor that can form in the pancreas or in other parts of the gastrointestinal tract, including the stomach, small intestine, colon, rectum, and appendix. **Neuroendocrine tumors arising in the lung are not considered GEP-NETs.**
Investigational drug	The study treatment whose properties are being tested in the study; this definition is consistent with US CFR 21 Section 312.3 and is synonymous with "investigational new drug."
Investigational treatment	Drug whose properties are being tested in the study as well as their associated placebo and active treatment controls (when applicable). This also includes approved drugs used outside of their indication/approved dosage, or that are tested in a fixed combination. Investigational treatment generally does not include other study treatments administered as concomitant background therapy required or allowed by the protocol when used in within approved indication/dosage.
Medication number	A unique identifier on the label of each study treatment package which is linked to one of the treatment groups of a study.
Other study treatment	Any drug administered to the patient as part of the required study procedures that was not included in the investigational treatment.
Subject Number (Subject No.)	A unique identifying number assigned to each patient/subject/healthy volunteer who enrolls in the study.
Study Phase	A subdivision of the study timeline; divides stages into smaller functional segments such as screening, treatment, follow-up, etc.
Randomization number	A unique treatment identification code assigned to each randomized patient, corresponding to a specific treatment arm assignment.

Rescue therapy	A quick-relief or fast-acting medication besides the investigational drug or control that can alleviate symptoms due to disease or lack of efficacy of the study treatment. It acts quickly to stop symptoms and, the effects are not long-lasting.
Stage related to study timeline	A major subdivision of the study timeline; begins and ends with major study milestones such as enrollment, randomization, completion of treatment, etc.
Stage in cancer	The extent of a cancer in the body. Staging is usually based on the size of the tumor, whether lymph nodes contain cancer, and whether the cancer has spread from the original site to other parts of the body.
Stop study participation	Point/time at which the patient came in for a final evaluation visit or when study treatment was discontinued whichever is later.
Study treatment	Includes any drug or combination of drugs in any study treatment administered to the patient (subject) as part of the required study procedures, including placebo and active drug run-ins.<br>In specific examples, it is important to judge investigational treatment component relationship relative to a study treatment combination; study treatment in this case refers to the investigational and non-investigational treatments in combination.
Study treatment discontinuation	Point/time when patient permanently stops taking study treatment for any reason.
Variable	Identifier used in the data analysis; derived directly or indirectly from data collected using specified assessments at specified timepoints.
Withdrawal of consent	Withdrawal of consent occurs only when a patient does not want to participate in the study any longer, and does not want any further visits or assessments, and does not want any further study related contact.

Protocol synopsis

Protocol number	CAAA601A22301
Full Title	This is a multicenter, stratified, randomized, open-label comparator-controlled, Phase III study in patients with somatostatin receptor positive, well-differentiated G2 and G3, advanced GEP NETs, diagnosed within 6 months prior to screening, comparing treatment with Lutathera (7.4 GBq/200 mCi x 4 administrations every 8± 1 weeks; cumulative dose: 29.6 GBq/800mCi) plus octreotide long-acting (30 mg every 8 weeks during Lutathera treatment and every 4 weeks after last Lutathera treatment) and high dose octreotide long-acting (60 mg every 4 weeks).
Brief title	A Phase III multi-center, randomized, open-label study to evaluate the efficacy and safety of Lutathera in patients with Grade 2 and Grade 3 advanced GEP-NET.
Sponsor and Clinical Phase	Sponsored by Advanced Accelerator Applications, Phase III Study.
Purpose and rationale	The pivotal Phase III NETTER-1 study showed that Lutathera with best supportive care (30mg octreotide long-acting) provided a significant increase in PFS to patients with progressive midgut carcinoid tumors (at enrollment) compared to patients treated with high dose (60 mg) octreotide long-acting. The NETTER-1 patient population included 34.5% of patients with G2 NET (65.5% G1), while G3 NETs were excluded. Only patients progressive on SSAs were eligible (2nd line), SSA-naïve patients were excluded.<br><br>The aim of the NETTER-2 study is to determine if Lutathera in combination with octreotide long-acting prolongs PFS in GEP-NET patients with high proliferation rate tumors (G2 and G3), when given as a 1st line treatment in comparison to treatment with high dose (60 mg) octreotide long-acting. SSA-naïve patients are eligible, as well as patients previously treated with SSAs in the absence of progression.<br><br>Based on extensive experience with Lutathera as well as octreotide LAR in adult GEP NET patients, and the relevance of the molecular target in adolescent GEP NET patients, the study will be open to adolescents aged ≥ 15 years and >40 kg body weight (BW); younger patients are not expected to present with the disease meeting the severity criteria for this trial.
Primary Objective(s)	To demonstrate that Lutathera is superior to active comparator in delaying the time-to-first occurrence of progression or death (PFS) as first line treatment.
Key Secondary Objectives	-To demonstrate the superiority of Lutathera, compared to active comparator, in terms of objective response<br>-To demonstrate the superiority of Lutathera, compared to active comparator, in terms of time to deterioration in selected QoL items/scales.

Other Secondary Objectives	-To evaluate the efficacy of Lutathera, compared to active comparator, in keeping the disease under control<br>-To evaluate the efficacy of Lutathera, compared to active comparator, in terms of duration of response<br>-To evaluate the safety and tolerability of Lutathera<br>-To evaluate the effect of Lutathera on overall survival
Study design	Overall, 222 patients will be randomized (2:1 randomization ratio) to receive treatment with Lutathera (7.4GBq/200 mCi x 4 administrations every 8± 1 weeks; cumulative dose: 29.6 GBq/800mCi) plus octreotide long-acting (30 mg every 8 weeks during Lutathera treatment and every 4 weeks after last Lutathera treatment) or high dose octreotide long-acting (60 mg every 4 weeks). Randomization will be stratified by Grade (G2 vs G3) and tumor origin (pNET vs other origin).<br><br>The primary endpoint of the study is PFS centrally assessed (target HR=0.5; 90% power, 1-sided α=2.5%). The primary analysis will be performed after 99 PFS events (99 evaluable and centrally confirmed disease progressions or death events) have occurred.<br><br>The study consists of a Screening Phase, a Treatment Phase, an optional Treatment Extension Phase (cross-over), an optional Re-treatment Phase and a Follow up Phase.<br><br>**Screening Phase**<br><br>The screening phase must be shortened as much as possible, in order to treat the patients possibly within 2 weeks after the consent signature.<br><br>The randomization must be performed immediately after all eligibility criteria are verified. As Lutathera production and shipment will take circa 12 days to be arranged, if a patient is randomized in the Lutathera arm, Lutathera first dose must be ordered immediately after randomization.<br><br>The baseline CT/MRI scan should be taken possibly on the same day as randomization or immediately before (within 1 week) to ensure that it reflects the disease status closely before the therapy start.<br><br>**Treatment Phase**<br><br>During the Treatment Phase, objective tumor response will be assessed at W16±1, W24±1 and then every 12±1 weeks from the randomization date, according to RECIST 1.1 criteria (central + local assessment up to first progression, then only local assessment).<br><br>Duration of the Treatment Phase:<br><br>Before the PFS primary analysis (i.e. 99 evaluable and centrally confirmed disease progressions or death events), patients continue the Treatment Phase until progression; after the PFS primary analysis, the Treatment Phase duration is limited to 72 weeks.

	At any time during the study (before or after the PFS primary analysis) any progressive patient (based on central imaging assessment) immediately ceases the Treatment Phase and proceeds to the Follow-up Phase. Patients who experience disease progression in the Lutathera arm after having received and benefitted from 4 doses/cycles as initial treatment have the option, if eligible, to enroll for re-treatment with Lutathera. In addition, patients randomized in the control arm have the option, if eligible, to enroll for post-progression cross-over with Lutathera.<br><br>**Optional Treatment Extension Phase (cross-over)**<br><br>In the control arm, any patient with radiological progression according to RECIST (based on central assessment) has the option to enroll for post-progression cross-over, upon signature of a new informed consent, to receive maximum 4 cycles of Lutathera (7.4 GBq/200 mCi x 4 cycles; cumulative dose: 29.6 GBq / 800mCi) plus 30 mg octreotide long acting every 8 weeks. If RECIST progression occurs after Week 72 post the primary end point analysis, the decision to enroll the patient in the cross-over phase will be based on local assessment. The time window to start Lutathera during the cross-over phase is within 4 years after the last patient has been randomized.<br><br>**Optional Re-treatment Phase**<br><br>Patients in the Lutathera arm with radiological progression based on RECIST criteria in central assessment will be offered enrollment in the optional re-treatment phase upon signature of a new informed consent. If RECIST progression occurs after Week 72 post the primary end point analysis, the decision to enroll the patient in re-treatment will be based on local assessment.<br><br>The criteria to enter the re-treatment phase include achievement of stabilization of disease or radiological response to Lutathera initial treatment for at least 6 months after receiving the 4th Lutathera dose and good safety/tolerability. If a patient's response has changed from PR/CR to SD within 6 months after the 4th Lutathera dose, the patient is still eligible for re-treatment, provided there is no documented progression within 6 months. Patients who received other systemic treatments for GEP-NET after progression (except somatostatin analogues) are not eligible for re-treatment. The time window to start re-treatment in this study is within 4 years after the last patient has been randomized.<br><br>In the re-treatment phase, patients will initially receive 2 administrations of Lutathera 7.4 GBq/200 mCi at 8-week interval. Based on the physician's judgment of the clinical benefit derived from the first 2 doses, up to 2 additional doses of Lutathera may be administered. A maximum of 4 cycles of Lutathera is allowed during the re-treatment period. All safety and efficacy assessments in the re-treatment period will be performed locally (SRI images must also be submitted to the central images reading center possibly within 1 month) and following the schedule in the initial treatment

	period. Assessments will be continued until disease progression is documented by investigator or until End of Study, whichever occurs first.<br>**Follow up Phase**<br>At the end of the Treatment Phase or after discontinuation for any cause (including disease progression), all patients will continue to be followed up to 3 years to continue data collection for the secondary endpoints of the study, such as long term safety and overall survival. Patients included in the optional crossover or re-treatment phase will be followed up at least 6 months and up to 3 years (or until EoS, whatever comes first).<br>During the Follow-up Phase, serious adverse events and adverse events of special interest (AESI) related to the study treatment as well as AESI of secondary hematological malignancies irrespective of causality, will be reported. Anti-tumor treatments administered after progression/discontinuation, disease status based on local CT/MRI assessment, and OS data will be collected every 6 months in both arms.<br>**End of the Study**<br>The End of Study is after 4 years have elapsed from the randomization of the last patient or 6 months after the last cross-over or re-treatment dose in the study, whichever occurs last. The time window to start cross-over or re-treatment with Lutathera in this study is within 4 years after the last patient has been randomized. For patients in the Lutathera arm who progress beyond this window, access to re-treatment with Lutathera may be granted via Post Study Drug Supply (PSDS) programs.
Population	In this study, safety and efficacy of treatment with Lutathera plus octreotide long-acting (30 mg) versus high dose octreotide long-acting (60 mg) is evaluated in patients ≥15 years with somatostatin receptor positive, well differentiated G2 (Ki67 index ≥10% to ≤ 20%) and G3 (Ki67 > 20% and ≤ 55%) advanced GEP-NETs. Patients with documented RECIST progression to previous treatments for the current GEP-NET at any time prior to randomization are not eligible to participate in this study.
Key Inclusion criteria	Subjects eligible for inclusion in this study must meet **all** of the following criteria:<br>1. Presence of metastasized or locally advanced, inoperable (curative intent) histologically proven, well differentiated Grade 2 or Grade 3 gastroenteropancreatic neuroendocrine (GEP-NET) tumor diagnosed within 6 months prior to screening.<br>2. Ki67 index ≥10 and ≤ 55%.<br>3. Patients ≥15 years of age and a body weight of >40 kg at screening.<br>4. Expression of somatostatin receptors on all target lesions documented by CT/MRI scans, assessed by any of the following somatostatin receptor imaging (SRI) modalities within 3 months prior to randomization:

	[68Ga]-DOTA-TOC (e.g. Somakit-TOC®) PET/CT imaging (or MRI when applicable based on target lesions), [68Ga]-DOTA-TATE PET/CT (or MRI when applicable based on target lesions) imaging (e.g. NETSPOT®), Somatostatin Receptor scintigraphy (SRS) with [111In]-pentetreotide (Octreoscan® SPECT/CT), SRS with [99mTc]-Tektrotyd, [64Cu]-DOTA-TATE PET/CT imaging (or MRI when applicable based on target lesions).<br>5. The tumor uptake observed in the target lesions must be > normal liver uptake.<br>6. Karnofsky Performance Score (KPS) ≥60.<br>7. Presence of at least 1 measurable site of disease.<br>8. Patients who have provided a signed informed consent form to participate in the study, obtained prior to the start of any protocol related activities.
Key Exclusion criteria	Subjects meeting any of the following criteria are not eligible for inclusion in this study.<br>1. Creatinine clearance <40 mL/min calculated by the Cockroft Gault method.<br>2. Hb concentration <5.0 mmol/L (<8.0 g/dL); WBC $<2x10^9$/L (2000/mm^3); platelets $<75x10^9$/L ($75x10^3$/mm^3).<br>3. Total bilirubin >3 x ULN.<br>4. Serum albumin <3.0 g/dL unless prothrombin time is within the normal range.<br>5. Pregnancy or lactation.<br>6. A) Women of child-bearing potential, defined as all women physiologically capable of becoming pregnant, are not allowed to participate in this study UNLESS they are using highly effective methods of contraception throughout the study treatment period (including cross-over and re-treatment, if applicable) and for 7 months after study drug discontinuation.<br>B) Sexually active male patients, unless they agree to remain abstinent (refrain from heterosexual intercourse) or be willing to use condoms and highly effective methods of contraception with female partners of childbearing potential or pregnant female partners during the treatment period (including cross-over and re-treatment, if applicable) and for 4 months after study drug discontinuation. In addition, male patients must refrain from donating sperm during this same period.<br>7. Peptide receptor radionuclide therapy (PRRT) at any time prior to randomization in the study.

	8. Documented RECIST progression to previous treatments for the current GEP-NET at any time prior to randomization.<br>9. Patients for whom in the opinion of the investigator other therapeutic options (eg chemo-, targeted therapy) are considered more appropriate than the therapy offered in the study, based on patient and disease characteristics.<br>10. Any previous therapy with Interferons, Everolimus (mTOR-inhibitors), chemotherapy or other systemic therapies of GEP-NET administered for more than 1 month or within 12 weeks prior to randomization in the study.<br>11. Any previous radioembolization, chemoembolization and radiofrequency ablation for GEP-NET.<br>12. Any surgery within 12 weeks prior to randomization in the study.<br>13. Known brain metastases, unless these metastases have been treated and stabilized for at least 24 weeks, prior to screening in the study. Patients with a history of brain metastases must have a head CT or MRI with contrast to document stable disease prior to randomization in the study.<br>14. Uncontrolled congestive heart failure (NYHA II, III, IV). Patients with history of congestive heart failure who do not violate this exclusion criterion will undergo an evaluation of their cardiac ejection fraction prior to randomization via echocardiography. The results from an earlier assessment (not exceeding 30 days prior to randomization) may substitute the evaluation at the discretion of the Investigator, if no clinical worsening is noted. The patient's measured cardiac ejection fraction in these patients must be ≥40% before randomization.<br>15. QTcF > 470 msec for females and QTcF > 450 msec for males or congenital long QT syndrome.<br>16. Uncontrolled diabetes mellitus as defined by hemoglobin A1c value > 7.5%.<br>17. Hyperkaleamia >6.0 mmol/L (CTCAE Grade 3) which is not corrected prior to study enrolment.<br>18. Any patient receiving treatment with short-acting octreotide, which cannot be interrupted for 24 h before and 24 h after the administration of Lutathera, or any patient receiving treatment with SSAs (eg octreotide long-acting), which cannot be interrupted for at least 6 weeks before the administration of Lutathera.<br>19. Patients with any other significant medical, psychiatric, or surgical condition, currently uncontrolled by treatment, which may interfere with the completion of the study.<br>20. Prior external beam radiation therapy to more than 25% of the bone marrow.

	21. Current spontaneous urinary incontinence.<br>22. Other known co-existing malignancies except non-melanoma skin cancer and carcinoma in situ of the uterine cervix, unless definitively treated and proven no evidence of recurrence for 5 years.<br>23. Patient with known incompatibility to CT Scans with I.V. contrast due to allergic reaction or renal insufficiency. If such a patient can be imaged with MRI, then the patient would not be excluded.<br>24. Hypersensitivity to any somatostatin analogues, to the IMPs active substance or to any of the excipients.<br>25. Patients who have participated in any therapeutic clinical study/received any investigational agent within the last 30 days.
Study treatment	In this study, approximately 222 patients with advanced G2-3 GEP-NET will be randomized (2:1 randomization ratio) to receive treatment with Lutathera (7.4 GBq or 200 mCi x 4 administrations every 8± 1 weeks; cumulative dose: 29.6 GBq or 800 mCi) plus octreotide long-acting standard dose (30 mg every 8 weeks during Lutathera treatment and every 4 weeks after last Lutathera treatment) or octreotide long-acting high dose (60 mg every 4 weeks).<br>The investigational drug product Lutathera® (^{177}Lu-DOTA^{0}-Tyr^{3}-Octreotate) will be provided by the Sponsor. The Sponsor will also provide the 2.5% Lys-Arg sterile amino acid solution for infusion (if it can't be compounded at the hospital Pharmacy), as well as octreotide long-acting (Sandostatin® LAR Depot) for the entire duration of the Treatment Phase (and optional Treatment Extension Phase in case of cross-over) of the study. During the re-treatment period, Lutathera® and 2.5% Lys-Arg sterile amino acid solution for infusion (if it can't be compounded at the hospital Pharmacy) will be provided by the Sponsor. Patients will switch to prescribed drugs in the follow up phase.<br>Anti-emetics, SRI imaging agents, short-acting octreotide or any other supportive care medication will not be supplied by the Sponsor.
Efficacy assessments	During the Treatment Phase, objective tumor response will be assessed at W16±1, W24±1 and then every 12±1 weeks from the randomization date, according to RECIST 1.1 criteria (central + local assessment up to first progression, then only local assessment). During the Follow up Phase RECIST objective tumor response will be assessed locally (scans every 6 months).
Pharmacokinetic assessments	Patients in the control arm (all countries except for China) will yield PK data from blood sampling performed at pre-dose with respect to the octreotide long-acting i.m. injection for the 2nd, 4th, 5th and 7th treatment cycle (that is respectively at week 4, 12, 16 and 24) for the determination of plasma trough levels. Data will be processed using a population based model, for exploratory purposes.

Key safety assessments	All adverse events (AEs), whether or not spontaneously reported by the patient, will be recorded starting from the signing of the ICF until the end of the Treatment Phase/optional Treatment Extension Phase in case of cross-over/optional re-treatment. During the Follow up Phase only related serious adverse events and related AESI other than secondary hematological malignancies will be recorded. AESI of secondary hematological malignancies will be recorded during the whole study irrespective of causality.
Data analysis	The primary efficacy and safety analyses will be performed after observing approximately 99 PFS events. At that time a soft database lock will ensure the integrity of the data and make further data entry for the continuation of the trial possible.<br><br>The primary endpoint of the study is progression-free survival (PFS), defined as the time from the date of randomization to the date of the first documented progression or death due to any cause. PFS will be assessed via central review according to RECIST 1.1.<br><br>The following statistical hypotheses will be tested to address the primary efficacy objective:<br><br>H_{01} (null hypotheses): $\Theta_1 \geq 0$ vs. H_{a1} (alternative hypotheses): $\Theta_1 < 0$<br><br>Where Θ_1 is the log hazard ratio of PFS in the Lutathera plus Standard Dose octreotide long-acting (30 mg) (investigational) arm vs. High Dose octreotide long-acting (60 mg) (control) arm.<br><br>The primary efficacy analysis to test this hypothesis and compare the two treatment groups will consist of a stratified log-rank test at an overall one-sided 2.5% level of significance in favor of the Lutathera plus Standard Dose octreotide long-acting (30 mg) arm. The stratification will be based on the following randomization stratification factors (grade: G2 vs. G3; and tumor origin: pNET vs other origin).<br><br>Secondary objectives of the study are to compare the two treatment groups with respect to objective response rate, time to deterioration in selected EORTC QLQ-C30 and QLQ-G.I.NET21 QoL items/scales, disease control rate, duration of response, safety and overall survival. Objective response rate and time to deterioration in EORTC QLQ-C30 global health status, diarrhea, fatigue, and pain are identified as key secondary endpoints.<br><br>A hierarchical testing procedure will be used to control the overall type I error in assessing the primary and key secondary objectives.
Key words	GEP-NET, PRRT, Lutathera.

Advanced Accelerator Applications approval signatures for: Clinical Trial Protocol CAAA601A22301

Protocol version and release date: Version 2.0, 05 October 2022

Steering Committee

[redacted] [redacted] [redacted]

Date

Advanced Accelerator Application (Sponsor)

[redacted] [redacted]

Date: 2022.10.18 09:06:50 +02'00'

Signature Date

[redacted] [redacted]

Date: 2022.10.17 13:58:11 -04'00'

Signature Date

[redacted] [redacted]

Date: 2022.10.17 14:03:08 -04'00'

Signature Date

[redacted] [redacted]

Date: 2022.10.18 16:24:31 +02'00'

Signature Date

[redacted] [redacted]

Date: 2022.10.18 15:13:03 +02'00'

Signature Date

Investigator approval signatures for:
Clinical Trial Protocol CAAA601A22301

Protocol version and release date: Version 2.0, 05 October 2022

Investigator signature

I have read the protocol and agree to conduct this trial in accordance with all stipulations of the protocol, with applicable laws and regulations and in accordance with the ethical principles outlined in the Declaration of Helsinki.

______________________	______________________	______________
Investigator	Signature	Date

Center name and address: ____

Center name: ____

Address: ____

Protocol Amendment 2.0

Amendment rationale

At the time of this amendment release, patient recruitment in the study is completed (222 patients randomized as of September 28th, 2022).

The primary purpose of amendment version 2.0 is to implement modifications in contraception requirements in line with Lutathera Investigator's Brochure (IB) version 17, dated 09 March 2022. The IB and corresponding changes in the informed consent form (ICF) were sent after the release to all enrolling sites and shared with IRBs/ECs as needed.

The changes are based on Sponsor Guideline on Prevention of Pregnancies in Participants in Clinical Trials as well as the Clinical Trials Facilitation and Coordination Group (CTFG) guideline on recommendations related to contraception and pregnancy testing in clinical trials. According to these guidelines, for radioligand therapies, highly effective contraception should be used during treatment and for (5 x T ½ + 6 months) after treatment in women of childbearing potential, and condom should be used during treatment and contraception period in male patients. For Lutathera, the effective half-life is 49 hours, and therefore, as per the calculation, the period of contraception for female patients should continue for 6 months and 10 days after the last dose. However, as a precautionary measure, the Sponsor decided to extend the highly effective contraception period from 6 months to 7 months for female patients.

In addition, the contraception requirements for male patients have been modified by specifying the use of condom and highly effective methods of contraception with female partners of childbearing potential or pregnant female partners during the treatment period (including cross-over and re-treatment, if applicable) and for 4 months after study drug discontinuation. This duration is based on an exposure of 5 terminal half-lives (49 hours for Lutathera) plus 90 day (life span of spermatozoa of 60–75 days for sperm production + 10–14 days for transport to epididymis) (EMA "Safety Working Party recommendations on the duration of contraception following the end of treatment with a genotoxic drug" dated on 27-Feb-2020).

This protocol amendment also includes the following updates:

- It has been clarified that based on Lutathera mechanism of action as a radioligand therapy, it can cause fetal harm when administered to a pregnant women.
- Some minor updates were made to the language regarding the objective response rate (ORR) and disease control rate (DCR) in the objective, assessments, and statistical sections of the protocol.
- According to the global Sponsor position, Lutathera should be infused slowly with an infusion rate **up to** 400 mL/hr, depending on the patient venous status, resulting in a duration of the infusion time of 30 ± 10 minutes. The wording "up to" was missing in the previous protocol version, and has been added now.
- Sterile conditions of amino acid solution infusion have been specified in order to emphasize that sterile solution is required for infusion.
- An error in the formula to calculate creatinine clearance has been noted and corrected.

- It has been clarified that serum chromogranin A (CgA) assessment is discontinued in Q3 2022 in South Korea due to lab kits shortage in the country. CgA assessments performed before discontinuation will be included in the analyses. This discontinuation does not impact study primary, secondary or exploratory objectives.
- It has been clarified that CT/MRI scan does not need to be repeated at EOT/EOS visits if it has been already done shortly before the visits.
- The sentence mentioning that in case of discrepancies between the local and central tumor response assessment "the Investigator may request the assessment by a third evaluator for final adjudication" has been removed. This option is actually not foreseen in this study. The central review is independent, performed by two reviewers, with adjudication by a 3rd independent reviewer in case of discrepancy between the first two. As per process, the central review is not impacted by the local response assessment.
- It has been clarified that if a patient discontinues Lutathera before confirmed disease progression, the patient should be followed with tumor assessments as per initial CT/MRI schedule until confirmed disease progression. This is done via central assessment in the initial Lutathera treatment, and via local assessment in the Lutathera re-treatment phase.
- A typo related to Karnofsky Performance score (KPS) evaluation has been corrected in the assessment schedule. Protocol section 8.5.7 states: "*KPS forms must be completed by a medical professional at each treatment and follow-up visit";* however, the assessment was not added in error in the control arm (Table 8-4) assessment schedule with the protocol amendment version 1.0 (typo). This typo in Table 8-4 is now corrected.
- Added clarification on applicable processes in case of Lutathera supply disruption and/or quality finding.
- Added clarification on patient management in regards to SARS-CoV-2 vaccination.
- Added clarification on informed consent withdrawal.
- Added clarification on study documents retention.
- Added clarifications on SSTR uptake assessment in somatostatin receptor imaging and CT/MRI scans for cross-over and re-treatment eligibility visits.
- Added clarification on management of CT/MRI scans after the second progression (PFS2) during the follow-up period
- Added clarifications on allowed time window for follow-up visits and re-treatment start.
- Added clarification on concomitant medication collection during follow-up phase.
- Added clarifications on CT/MRI scans scheduled during cross-over/re-treatment phase.
- Added clarification on EORTC QoL scales which are captured in the key secondary analysis
- Added a footnote in the Dose modification table clarifying that lymphopenia is not a dose modifying toxicity.

- Editorial changes and text corrections made for clarification, where required.

Changes to the Protocol

Changes to specific sections of the protocol are shown in the track changes version of the protocol.

1. Protocol synopsis, Data Analysis: Removed reference to QLQ-G.I.NET21 for the key secondary endpoints
2. Protocol summary, Section 5.2, Appendix 1, Appendix 5:
    a. Exclusion criterion #6a: updated to reflect that female patients of child-bearing potential must use highly effective contraception during treatment and **for 7 months** (instead of 6 months) after the last Lutathera dose.
    b. Exclusion criterion #6b: updated to reflect the use of **"condoms and highly"** effective methods of contraception by male patients during treatment **and for 4 months** (instead of 6 months) after the last Lutathera dose.
3. Objectives and endpoints, Section 2
    a. Updated definitions for Objective response rate (ORR) and Disease control rate (DCR)
4. **"Sterile"** added before "**amino acid solution"** in all relevant instances where the solution is cited in the Protocol.
5. Sections 3.2 and 3.4: added a clarification that if a patient discontinues Lutathera before confirmed disease progression, the patient should be followed with tumor assessments as per initial CT/MRI schedule until confirmed disease progression.
6. Sections 3.5, 8.3.1, 8.5.2, 8.5.10 updated to clarify that the allowed time window for follow-up visits is 6 months ± 1 month.
7. Section 4: Added a new section (4.6) on rationale for public health emergency mitigation procedures under.
8. Updated Sections 4.2 and 5.2.2 to clarify that a window of -2 weeks is allowed for the confirmation of 6 months of SD/PR/CR response.
9. Updated Section 5.2.1, Section 5.2.2, Table 8-3 and Table 8-4 to clarify that post progression Cross-Over/Re-treatment Eligibility CT/MRI scan doesn't need to be repeated if performed within the previous 12 weeks. Both Cross-Over/Re-treatment Eligibility SRI and CT/MRI scans need to be submitted to the central imaging center for a second review: there is no central 'real time' assessment of the Cross-Over and Re-treatment Eligibility SRI uptake; however, the images should be submitted to the central imaging center possibly within 1 month.
10. Added Section 6.2.4 "SARS-CoV-2 vaccination" to provide clarifications on the risk/benefit assessment in regards to SARS-CoV-2 vaccination and vaccination data collection.
11. Table 6.4: added footnote "[1] No dose modification required for hematological toxicities Grade 3 or Grade 4 solely due to lymphopenia "
12. Table 6.5: added "up to" to the infusion rate of Lutathera (i.e. "up to 400 ml/hr").
13. Section 7: Study treatment "may involve unknown risks to the fetus if pregnancy were to occur during the study" has been replaced by "Lutathera can cause fetal harm when administered to a pregnant woman".
14. Section 8.2, 8.5.1, Table 8-3 and Table 8-4, updated to clarify that during the Follow-up Phase concomitant medications must be collected only if administered for related SAEs/AESI and/or for secondary hematological malignancies (in addition to anticancer medications).
15. Section 8.3.1 updated to clarify that for progressive patients participating in cross-over or re-treatment, CT/MRI scans performed after the cross-over or re-treatment eligibility will follow the visits schedule counted from the 1st Lutathera dose.
16. Section 8.3.2 updated to clarify that after the first progression is confirmed centrally and the second progression is confirmed locally (i.e. after PFS2 event), the sites which do not

routinely perform 6-monthly follow up CT/MRI scans for progressive GEP-NET patients can omit this examination at the subsequent patient's visits (no protocol deviation).

17. Section 8.3.2: Updated definition for Objective response rate (ORR)
18. Table 8-3: a footnote added to clarify that "For patients who entered the Lutathera re-treatment phase, since there are no procedures requiring in-person patient's attendance, the following visits can be performed by phone: Visits W32, 40, 44, 52, 56, 64 and 68 following Lutathera re-treatment (for Germany only: visits W56, 64 and 68)."
19. Table 8-4: Added Karnofsky Performance score (KPS) evaluation during the 3-years follow up phase for control arm.
20. Section 8.5.2. Creatinine clearance formula has been corrected
    *Est. Creatinine Clearance = [[140-age(yr)]*weight (kg)]/[~~48816~~ 0.814* serum Cr(~~mmol~~ umol/L)] (multiply by 0.85 for women)*
21. Section 8.5.5:
    - The sentence related to ECG entry in eCRF has been removed, since no ECG parameters are entered in eCRF (only clinical significance is entered).
    - The sentence related to ECG local review has been updated to clarify the process to document clinical significance of ECG findings.
22. Sections 8.7 and 12.5.3 and Table 8-2, Table 8-3, Table 8-4 updated to clarify that CgA assessment is discontinued in Q3 2022 in South Korea due to lab kits shortage (assessments performed before discontinuation will be included in the analyses).
23. Section 8.8: The sentence saying that "the Investigator may request the assessment by a third evaluator for final adjudication." has been removed.
24. Section 9.1.1 has been updated to describe applicable procedures in case of sudden temporary Lutathera supply disruption and/or a quality finding after the product release.
25. Section 9.1.2 has been updated to include clarifications on withdrawal of consent/opposition to use of data and/or biological samples.
26. Section 9.2, Table 8-3 and Table 8-4: clarified that at the EOT, a CT/MRI scan is required only if not taken in the previous 12 weeks. If no post-baseline scan is available, a scan is needed at EOT. At the EOS, a CT/MRI scan is required only if a scan was not taken in the previous 6 months.
27. Section 12.1: Added definitions for cross-over and re-treatment sets
28. Section 12.4.3: Fixed formatting for this section
29. Analysis of secondary endpoints, Section 12.5
    a. Updated definition of ORR as well as highlighted that the key secondary endpoint is calculated per central review
    b. Section 12.5.1: Emphasized that further details for best sensitivity and supplementary analyses for the key secondary endpoints will be provided in the SAP.
    c. Section 12.5.1: Removed reference to QLQ-G.I.NET21 for the key secondary endpoints
    d. Section 12.5.2: Updated definition of Disease Control Rate (DCR)
30. Section 13.4 has been updated to include clarifications on study documents verification and retention.

A detailed description of each modification is provided in the separate document '**TABLE OF CHANGES**'.

This amendment is classified as **substantial** by Sponsor.

All protocol changes have been reviewed and approved by the Study Steering Committee.

IRBs/IECs

A copy of this global amended protocol will be sent to Institutional Review Boards (IRBs)/Independent Ethics Committee (IECs) and Health Authorities.

The changes described in this amended protocol require IRB/IEC approval prior to implementation.

The changes herein affect the Informed Consent. Sites are required to update and submit for approval a revised Informed Consent that takes into account the changes described in this protocol amendment.

Protocol Amendment 1.0

Amendment rationale

At the time of this amendment release, patient recruitment in the study is ongoing. A total of 123 patients have been randomized in the study as of September 23rd, 2021. The amendment does not affect the primary and key secondary endpoints of the study.

The protocol has been amended for the following reasons:

Addition of Re-treatment phase:

The main purpose of this protocol amendment is to introduce optional re-treatment with additional doses of Lutathera for patients treated in the Lutathera arm upon disease progression. Once patients experience disease progression, they may receive any subsequent antineoplastic treatment. Based on a meta-analysis (Strosberg et al., 2021) including patients with progressive GEP-NETs (n=560) who received re-treatment with Lutathera (one to six doses during re-treatment, majority of patients received 200 mCi per administration), median PFS was 12.52 months (available in 414 patients), median OS was 26.78 months from the start of re-treatment (available in 194 patients), and DCR was 71% (available in 347 patients) supporting the clinical benefit of re-treatment. The safety profile of Lutathera re-treatment was similar to initial treatment. Hematologic grade 3/4 AEs were reported in 9% of patients who received PRRT re-treatment. Notably, AML and MDS occurred in <1% of patients who received re-treatment with Lutathera, comparable to the incidence observed with initial PRRT (2% in the studies included in the meta-analysis). These data indicate that re-treatment with Lutathera may be considered as a treatment approach to maximize benefit without compromising on safety upon careful assessment of clinical status of each individual patient.

Based on this body of evidence, the patient candidates for re-treatment with Lutathera in this protocol amendment, are patients with centrally documented tumor progression in Lutathera arm (if RECIST progression occurs after Week 72 post the primary end point analysis, the decision to enroll the patient in re-treatment will be based on local assessment), who have completed the 4 doses/cycles of Lutathera during the treatment phase, had CR/PR/SD as best response for at least 6 months after the 4th Lutathera dose, and had tolerated the treatment (according to criteria defined in Section 5.2.2). An SSTR uptake must be documented in target lesions by SRI before starting re-treatment.

Patients will be offered to receive initially two Lutathera doses in re-treatment period (Section 4.3). Based on the physician judgment of clinical benefit derived from the first 2 doses, patients may receive up to 2 additional doses of Lutathera (criteria for additional doses are listed in Section 3.4). A maximum of 4 doses of Lutathera is allowed during the re-treatment period.

All safety and efficacy assessments in the re-treatment period will be performed as per the schedule in initial treatment period (Table 8-2) and will be continued until disease progression is documented by investigator.

A descriptive analysis will be performed in all patients who received Lutathera re-treatment (Section 12.6.4). Safety will be presented in a similar manner to that in Section 12.5.3. All efficacy analyses for ORR, DoR, PFS, PFS2, and OS will be descriptive. Definitions for ORR, DoR, and PFS (as per local assessment) are the same definitions in Section 12.5.2 except that the baseline will be based on the tumor assessment which demonstrated progression thus allowing re-treatment with Lutathera. Definitions for OS and PFS2 are the same to that in Section 12.5.2 and Section 12.6.1, respectively.

The assessment of Lutathera re-treatment has been added as an exploratory objective in the study: *To explore the safety and efficacy (ORR, PFS, PFS2, DoR, OS) of re-treatment with Lutathera in progressive patients in Lutathera arm*. The following exploratory endpoints have been added:

- Safety (Re-treatment): Rate of adverse events and laboratory toxicities during re-treatment with Lutathera.
- ORR (Re-treatment): Rate of complete and partial responses after receiving re-treatment with Lutathera (CR, PR) (locally assessed according to RECIST 1.1).
- PFS (Re-treatment): Time from the 1st dose of re-treatment with Lutathera to objective tumor progression (locally assessed according to RECIST 1.1) or to death due to any cause.
- PFS2 (Re-treatment): Time from randomization to the objective tumor progression (locally assessed according to RECIST 1.1) or to death due to any cause after receiving re-treatment with Lutathera.
- DOR (Re-treatment): Time from initially meeting the criteria for response (CR or PR) after receiving re-treatment with Lutathera until the time of progression according to RECIST 1.1 (locally assessed) or death due to underlying disease only.
- OS (Re-treatment): Time from the randomization date until the day of death due to any cause in patients who have received re-treatment with Lutathera.

Other Protocol changes

- Dose modification rules have been updated, to clarify that toxicities meeting DMT criteria which are resolved before the next scheduled dose will not trigger a dose reduction (Section 6.5.2.1). The dose modification rules in the initial protocol reflected Lutathera label DMT guidance. However, patients in NETTER-2 have higher grade tumors (G2 and G3) as compared to the patients in the approved label (G1 and G2); therefore, the administration of a full dose (200 mCi/7.4 GBq) if a toxicity is resolved, even if this toxicity met DMT criteria, is considered to maximize the potential treatment effects. Therefore, in view of the higher GEP-NET severity in the NETTER-2 study, and in order to maintain a positive benefit/risk ratio, the protocol has been amended in Section 6.5.2.1. There are no changes to requirements for dose interruption if a toxicity is not resolved, nor to requirements for treatment discontinuation (Section 6.5.2, Section 6.5.3).

- Inclusion and exclusion criteria have been modified for clarity and consistency
    - Inclusion criterion 4: amended to include all available SSTR-directed imaging agents and modalities to evaluate baseline expression of somatostatin receptors on target lesions. The following imaging agents and modalities were added: [99mTc]-Tektrotyd, [64Cu]-DOTA-TATE PET/CT; in addition, option of PET/MRI has been added to all PET imaging modalities (when applicable based on target lesions).
    - Inclusion criterion 5: amended to delete the second part of the sentence *"The tumor uptake observed in the target lesions must be > normal liver uptake ~~observed on planar imaging~~"* (removed as not applicable in all SRI methods).
    - Exclusion criterion 6: amended to specify in exclusion criteria the contraception methods and sperm donation recommendation during the study for sexually active male patients, separately from requirements for female patients (important details were listed in the Appendix 1 but missing in the exclusion criteria). In addition, Appendix 1 was updated to align with CTFG guidelines for contraception (v1.1, Sep 2020).
    - Exclusion criterion 10: amended to specify in exclusion criteria that only systemic therapies for the studied disease (GEP-NET) are excluded (if administered for more than 1 month or within 12 weeks prior to randomization).
    - Exclusion criterion 11: amended to specify in exclusion criteria that only previous radioembolization, chemoembolization and radiofrequency ablation for the studied disease (GEP-NET) are excluded.
    - Exclusion criterion 13: amended to clarify that MRI is acceptable for assessment of brain metastasis, in addition to CT.
    - Exclusion criterion 14: amended to clarify that congestive heart failure history can be assessed via echocardiography only (gated equilibrium radionuclide ventriculography is not required).
    - Exclusion criterion 16: amended as the definition of uncontrolled diabetes ("fasting blood glucose > 2 ULN") was non-standard; the revised definition is "hemoglobin A1c > 7.5%" (NICE guideline on Type 2 diabetes in adults: management, 2015).
    - Exclusion criterion 18: amended to delete the second part of Exclusion Criterion 18, which is irrelevant in this study as the tumor uptake on target lesions must be greater than the liver uptake in all patients in this study (typo correction) (as described in inclusion criteria 5): *"Any patient receiving treatment with short-acting octreotide, which cannot be interrupted for 24 h before and 24 h after the administration of Lutathera, or any patient receiving treatment with SSAs (eg octreotide long-acting), which cannot be interrupted for at least 6 weeks before the administration of Lutathera~~, unless the tumor uptake on target lesions observed by study permitted somatostatin receptor imaging (SRI) modalities during continued long acting SSA treatment is greater than the liver uptake observed by planar imaging~~."*
    - Exclusion criterion 24: amended to exclude patients with hypersensitivity to the IMPs active substance or to any of the excipients.

- Included list of possible Octreotide Drug-Drug-Interactions (DDI) in Section 6.2.3, according to the Octreotide label.
- Clarified requirements for contraception (Section 8.5.3 and Appendix 1).
- Added Section 9.2.1 to clarify that for patients who are eligible to Lutathera re-treatment beyond the EoSs window a may receive post-trial access (PTA) to Lutathera. PTA to Lutathera may be granted via Post Study Drug Supply (PSDS) programs, based on local regulation in a non-trial setting. The PSDS must comply with local laws and regulations in the participating trial countries.
- Added clarifications on safety reporting procedures in Section 10.
    - During the treatment phase, all SAEs and AESI need to be reported to the Sponsor irrespective of causality. During the follow-up phase, SAEs and AESI need to be reported only if considered related to the study treatment except for all secondary hematological malignancies which need to be reported as AESI irrespective of causality.
    - Clarified the 4 categories of AESI (Hematotoxicity; Secondary hematological malignancies; Nephrotoxicity; Cardiovascular and electrolyte disorders) (Table 10-1), and removed the table with AESI by treatment type, as AESI must be reported for both arms.
    - Clarified that serious adverse events (SAEs) must be reported immediately, without undue delay and under no circumstances later than 24 hours following knowledge.
    - Clarified that progression of GEP-NET (including fatal outcomes), if documented by use of appropriate method, should not be reported as a serious adverse event, except if the investigator considers that progression of malignancy is related to study treatment.

- Explained methods of providing continuing care to the patients during COVID-19 pandemic (Sections 8, 8.2, 8.5.1, 8.5.2, 9.1.1, 10.1.1, 10.1.2, 14).

- Updated definition of EoS in Section 9.2: End of Study (EOS) is after 4 years have elapsed from the randomization of the last patient, or 6 months after the last dose of crossed over/re-treatment patient, which occurs last.

- Section 12 has been updated as follows:
    - Corrected the PFS definition throughout the document to align with currently approved Section 12.4.1 that define the PFS as follows: "The primary endpoint of the study is progression-free survival (PFS), defined as the time from the date of randomization to the date of the first documented progression or death due to any cause."
    - Added statistical hypothesis information in the protocol synopsis for the data analysis sub-section.
    - Updated language for PFS2 endpoint in the Objectives and Endpoints section to improve clarity.

    - Updated definition for DOR throughout protocol as follows (addition in ***bold/italic***): "DOR: The Duration of Response (DOR) is defined as the time from initially meeting the criteria for response (CR or PR) until the time of progression according to RECIST 1.1 ***or to death due to underlying disease only***"
    - Updated language at the beginning of Section 12 to further highlight the primary objective and the timing of analyses throughout the study.
    - Re-structured Section 12.5 with the following updates:
        - Separated key secondary endpoints into its own sub-section
        - Highlighted that TTD for key secondary endpoints was for EORTC QLQ-G.I.NET21 questionnaire, EORTC QLQ-C30 questionnaire: global health status (TTD)-diarrhea (TTD)-fatigue, (TTD)-pain (TTD).
        - Moved pharmacokinetics and patient related outcomes section (for PRO assessments that are not part of key secondary endpoints) to Section 12.6.
    - Added subsections to Section 12.6 including the addition of the sub-section for re-treatment of Lutathera.

- This protocol amendment specifies the procedures required locally only for particular countries, based on local requirements (the following procedures have already been implemented via local amendments and are included in the global protocol via this amendment):
    - Only for Germany and UK:
        - Additional pregnancy tests added in the assessment schedule (Table 8-2)
    - Only for Germany:
        - Added a section outlining that, according to the Octreotide label, ultrasonic examination of the gallbladder should be performed before and at six-monthly intervals during Octreotide LAR treatment in order to detect and prevent possible cholecystitis and biliary duct dilatation risks for the patients is recommended (Section 8.5.8).
        - SPECT/CT and whole body planar imaging (local assessment only) added to the assessment schedule (Section 8.5.9 and Table 8-2). The assessments should be performed after each Lutathera injection, as per local standards. The dosimetry results will be collected in eCRF.
    - Only for China:
        - Octreotide PK assessment will not be done in patients enrolled in China (Section 8.6).

- Updated Appendix 1 – Precautions for pregnancy to align with CTFG recommendations (CTFG 2020).

- Updated Appendix 2 to clarify that the detailed methods of administration of Lutathera and amino acids are described in the pharmacy manual.

- Updated Appendix 5 – Recommendations for Patients Treated with Lutathera to align with latest approved recommendations.

A detailed description of each modification is provided in the separate document '**TABLE OF CHANGES')**.

This amendment is classified as **substantial** by Sponsor.

All protocol changes have been reviewed and approved by the Study Steering Committee.

IRB/IEC

A copy of this amended protocol will be sent to the Institutional Review Board (IRBs)/Independent Ethics Committee (IECs) and Health Authorities (HA).

This amended protocol requires IRB/IEC/HA approval prior to implementation.

The Informed Consent has been updated according to the protocol amendment changes.

1 Introduction

1.1 Background

Neuroendocrine tumors (NET) are rare malignant neoplasms that can arise throughout the body, and account for approximately 1% of all human tumors. NETs have been classified according to their embryonic origin as foregut, midgut, or hindgut NETs. The World Health Organization (WHO) staging system classifies gastroenteropancreatic NET (GEP-NET) based on primary tumor localization, size, mitotic activity, invasiveness, and functional status (Klöppel, 2011). Despite certain common morphological and immunohistochemical features, there is significant heterogeneity in the prognosis and treatment strategies according to the primary site, histological differentiation (poorly or well-differentiated) and stage. Histologic differentiation and proliferative activity are the strongest predictors of survival. In terms of site of origin, the majority (50-70%) of NETs diagnosed in Western countries are gastrointestinal NETs (Modlin, Lye, & Kidd, 2003). Of 270 NETs originating in the midgut or hindgut, 62% (5- year survival rate (YSR), 95.2%) were Grade 1, 32% (5-YSR 82.0%) were Grade 2, and 6% (5-YSR, 51.4%) were Grade 3 NETs (Jann, et al., 2011). 50-70% of NETs of unknown origin have been reported to be well-differentiated (Catena, et al., 2011) and have a similar behavior and prognosis to midgut NETs (Kirshbom, Kherani, Onaitis, Feldman, & Tyler, 1998).

Surgical resection of the primary and metastatic lesions remains the mainstay of treatment, and the only way to obtain a cure. However, resection is often not possible as NETs are frequently detected in a more advanced tumor stage (Yao, et al., 2008).

In patients with inoperable NET, the treatment goal is to prolong survival, improve and maintain quality of life, to control tumor growth, and to control secretory symptoms (if the tumor is functional). Somatostatin analogs (SSAs) octreotide long-acting 30 mg (Sandostatin® LAR®) or lanreotide ATG 120 mg (lanreotide ATG, SOMATULINE® DEPOT or SOMATULINE® AUTOGEL®) have become the mainstay of treatment in patients with low- or intermediate-grade GEP-NET.

In the PROMID study, 42 patients with metastatic midgut NET who received octreotide long-acting 30 mg/month had more than double the time to tumor progression compared with 43 patients who received placebo (14.3 versus 6.0 months, respectively; P=0.000072). Overall, 67% of patients treated with octreotide long-acting achieved stable disease compared with 37% of patients who received placebo (Rinke, et al., 2009). In the CLARINET study, patients with G1-2 midgut and pancreatic NET treated with lanreotide 120 mg/month had significant prolongation of progression free survival (PFS) when compared to placebo (median PFS not reached vs. 18.0 months, P<0.001; HR for progression or death with lanreotide vs. placebo, 0.47; 95% CI 0.30 to 0.73) (Caplin, et al., 2014).

Where approved, the dose for octreotide long-acting for the treatment of advanced midgut NET is 30 mg/month. Higher doses of octreotide up to 120 mg per month have been used for symptom control in patients no longer responding adequately to standard doses. Higher doses have also been used for tumor control though controlled studies are lacking (Broder, Beenhouwer, Strosberg, Neary, & Cherepanov, 2015). Three studies have investigated the antiproliferative efficacy of high-dose treatment with either octreotide long-acting (up to 160 mg every 2 weeks) or lanreotide (up to 15 mg/d), showing disease stabilization in 37-75% of advanced midgut carcinoid and/or metastatic GEP-NET patients (Eriksson, Renstrup, Imam, & Oberg, 1997; Faiss, et al., 1999; Welin, et al., 2004).

Furthermore, retrospective analyses suggest that higher doses of octreotide may delay the time to tumor progression / other types of intervention (Lau, Abdel-Rahman, & Cheung, 2018; Chadha, et al., 2009). In a small (n=28) sequential study, increased frequency of dosing of somatostatin analogs resulted in delayed time to tumor progression and time to biochemical progression (Ferolla, et al., 2012).

In patients whose disease has progressed, mTOR therapy (everolimus), tyrosine kinase inhibitors (sunitinib) and peptide radionuclide receptor therapy (PRRT) provide treatment options (Yao, et al., 2010; Yao, et al., 2011; Pavel, et al., 2011; Yao, et al., 2016; Kwekkeboom & Krenning, Peptide Receptor Radionuclide Therapy in the Treatment of Neuroendocrine Tumors., 2016). Currently there is no broadly accepted standard chemotherapy for the treatment of NET. A combination of streptozotocin and 5-flourouracil or doxorubicin is frequently used in patients with well-differentiated pancreatic NET (PNET), with inoperable progressive liver metastasis, but there is no robust evidence to support the use of chemotherapy in patients with NETs of other origin (Pavel, et al., 2012).

Within the group of neuroendocrine neoplasm (NEN) G3, the distinction between NET G3 and neuroendocrine carcinomas (NEC) G3 is clinically meaningful. NET G3 and NEC are characterized by significant differences in Ki67 index (tumors with a Ki67 index between 20 and 55% are less aggressive than tumors with a Ki67 index above 55%). While platinum-based chemotherapy is effective in NEC, it seems to have limited value in NET G3. Treatments established for NET G2 such as temozolomide based chemotherapy or PRRT may be considered for the treatment of NET G3 (Rinke 2017).

Tumor-targeted peptide receptor radionuclide therapy (PRRT) has been under clinical evaluation since 1992 for tumors expressing somatostatin receptors. The biological basis for radionuclide receptor imaging and receptor targeted radionuclide therapy is the receptor-mediated internalization and intracellular retention of radiolabelled somatostatin analogues. Sst_2 receptors are an attractive target for PRRT because the receptor density is higher on tumor than on non-tumor tissue (Reubi, Waser, Schaer, & Laissue, 2001; Reubi J. C., 2003), and because sst_2 receptors internalize into cells after ligand binding. Consequently, the radioactivity delivered by the radiolabelled peptide is captured in the target cell after binding to the sst_2 receptor (Reubi, et al., 2000).

^{177}Lu-DOTA^0-Tyr^3-Octreotate (^{177}Lu-Dotatate, Lutathera®) consists of a somatostatin peptide analogue, coupled to the metal-ion chelating moiety, DOTA, and radiolabelled with ^{177}Lu. ^{177}Lu-DOTA^0-Tyr^3-Octreotate binds with high-affinity to somatostatin receptors and retains its binding properties and physiological functions when complexed with ^{177}Lu. ^{177}Lu emits low to intermediate-energy beta-particles with an E_{max} of 0.5 MeV, and which have a tissue penetration range of up to 2 mm. The relatively short penetration range of ^{177}Lu betas leaves more of the radiation dose in the tumor with less loss to the surrounding tissues.

There is rapid urinary clearance of radiolabelled ^{177}Lu-DOTA^0-Tyr^3-Octreotate from the circulation, which gives this radiopharmaceutical a major advantage over other approaches, such as cell targeted radiolabelled antibodies.

Because of ^{177}Lu-DOTA^0-Tyr^3-Octreotate mechanism of action, there is some radioactivity retention in the kidneys, however concomitant administration of the amino acids lysine and arginine reduces renal uptake of radioactivity without altering tumor uptake (Kwekkeboom, et al., 2001; Strosberg, et al., 2017). This phenomenon of protection mediated by co-infusion of a lysine

and arginine solution occurs by "blocking" the mechanism for renal tubular uptake of proteins or peptides (Hammond, et al., 1993; Rolleman, Valkema, Jong, Kooij, & Krenning, 2003). The co-administration of 2.5% lysine and arginine (Lys-Arg) amino acid solution yields about 33% inhibition of renal uptake of radioactivity at 24 hours and is better tolerable compared to the commercial amino acid solutions, specifically in terms of nausea and vomiting, due to the higher osmolality of the more complex formulated commercial AA solutions (Rolleman, Valkema, Jong, Kooij, & Krenning, 2003; Kwekkeboom, et al., 2008).

Common side effects of Lutathera include lymphopenia, increased GGT, AST and/or ALT, vomiting, nausea, hyperglycemia and hypokalemia. Serious side effects of Lutathera include myelosuppression, secondary myelodysplastic syndrome and leukemia, renal toxicity, hepatotoxicity, neuroendocrine hormonal crises and infertility.

Lutathera (lutetium (^{177}Lu) oxodotreotide) was approved in Europe on September 26, 2017 for the treatment of unresectable or metastatic, progressive, well differentiated (G1 and G2) GEP-NETs and in the USA on 26 January 2018 for the treatment of somatostatin receptor positive GEP-NETs.

The approval of Lutathera was supported by two studies:

- The first was the Phase III NETTER-1 study sponsored by Advanced Accelerator Applications. This was multicenter, stratified, open, randomized, comparator-controlled, parallel-group study, comparing treatment with ^{177}Lu-DOTA^0-Tyr^3-Octreotate (Lutathera, lutetium Lu 177 dotatate) plus best supportive care (30 mg octreotide long-acting) to treatment with high dose (60 mg) octreotide long-acting in patients with inoperable, somatostatin receptor positive, histologically proven midgut carcinoid tumors, progressive under octreotide long-acting (Strosberg, et al., 2017; Strosberg, et al., 2018). A significant improvement in PFS, the primary endpoint, was demonstrated. There were 21 confirmed events (disease progressions according to RECIST 1.1 centrally assessed or deaths without confirmed progression) in the Lutathera arm and 70 events in the octreotide long-acting 60 mg arm. The median PFS for the control arm was 8.5 months while the median PFS for the Lutathera arm had not yet been reached. The improvement in median PFS in the Lutathera arm was statistically significant with a hazard ratio of 0.18 (95% CI, 0.11 - 0.29), demonstrating a risk reduction of 79% in the likelihood of having a progression or death event when treated with Lutathera versus 60mg octreotide long-acting.
- The second study was based on data from 1,214 patients with somatostatin receptor-positive tumors, including GEP-NETS, who received Lutathera at the Erasmus Medical Centre in the Netherlands (Kwekkeboom, et al., 2008; Brabander, et al., Long-Term Efficacy, Survival, and Safety of [177Lu-DOTA0,Tyr3]octreotate in Patients with Gastroenteropancreatic and Bronchial Neuroendocrine Tumors., 2017a). Complete or partial tumor remission was reported in 39% of patients.

On June 02, 2016 and 15 December, 2016 Advanced Accelerator Applications obtained marketing approval from the FDA for NETSPOT™ (68Ga-Dotatate) and from the European Commission for Somakit-TOC™ (68Ga-Dotatoc), the diagnostic companions of Lutathera.

Neuroendocrine tumors (NET) are rarely seen in childhood. The incidence in children and adolescents is low at 2.8 per million persons under the age of 30 years. Despite their low incidence, NETs represent the most frequent tumor of the gastrointestinal tract in children (Howell & O'Dorisio, 2012). Currently all treatments approved for GEP-NETs are for adults only.

1.2 Purpose

The pivotal Phase III NETTER-1 study showed that Lutathera with best supportive care (30mg octreotide long-acting) provided a significant increase in PFS to patients with progressive midgut carcinoid tumors (at enrollment) compared to patients treated with high dose (60 mg) octreotide long-acting. The NETTER-1 patient population included 34.5% of patients with G2 NET (65.5% G1), while G3 NETs were excluded. Only patients progressive on SSAs were eligible (2nd line), SSA-naïve patients were excluded.

The aim of the NETTER-2 study is to determine if Lutathera in combination with octreotide long-acting prolongs PFS in GEP-NET patients with high proliferation rate tumors (G2 and G3), when given as a 1st line treatment in comparison to treatment with high dose (60 mg) octreotide long-acting. SSA-naïve patients are eligible, as well as patients previously treated with SSAs in the absence of progression. Based on extensive experience with Lutathera as well as octreotide LAR in adult GEP-NET patients, and the relevance of the molecular target in adolescent GEP-NET patients, the study will be open to adolescents aged ≥ 15 years and >40 kg body weight (BW); younger patients are not expected to present with the disease meeting the severity criteria for this trial. Due to the rarity of the disease, and specifically GEP-NETs with the severity assessed in this study, no minimum number of adolescent patients is required. The study has been open to adolescents to ensure that access is not unnecessarily denied.

In addition, in the NETTER-2 study, any progressive patient in the high dose octreotide long acting arm has the option, if eligible, to enroll for post-progression cross-over with Lutathera.

Furthermore, patients who experience disease progression in the Lutathera arm after having received and benefitted from 4 doses/cycles in an initial treatment have the option, if eligible according to criteria described in Section 5.2.2, to enroll for re-treatment phase with Lutathera.

The purpose of the NETTER-2 study is to evaluate the efficacy and safety of Lutathera in patients ≥15 years of age with Grade 2 and 3 advanced GEP-NET (Ki67 index of ≥ 10 and ≤ 55%), considered being a candidate for treatment with high dose octreotide long-acting.

2 Objectives and endpoints

The study primary, secondary, explorative objectives and endpoints are described in Table 2-1.

Table 2-1. Study objectives and endpoints

**Primary Objective**	**Endpoint for primary objective**
To demonstrate that Lutathera is superior to active comparator in delaying the time-to-first occurrence of progression or death (PFS) as first line treatment	PFS: Time from randomization to the first line progression (centrally assessed according to RECIST 1.1) or death due to any cause.
**Key Secondary Objectives**	**Endpoints for key secondary objectives**
-To demonstrate the superiority of Lutathera, compared to active comparator, in terms of objective response	-ORR: Rate of patients with best overall response of partial response (PR) or complete response (CR) (centrally assessed according to RECIST 1.1)
-To demonstrate the superiority of Lutathera, compared to active comparator, in terms of time to deterioration in selected QoL items/scales	-Time to decline (TTD) by 10 points from baseline in the following scores measured by the EORTC QLQ-G.I.NET21 questionnaire and EORTC QLQ-C30 questionnaire: global health status, diarrhea, fatigue, and pain.
**Other Secondary Objectives**	**Endpoints for other secondary objectives**
-To evaluate the efficacy of Lutathera, compared to active comparator, in keeping the disease under control	-DCR: Rate of patients with best overall response of partial response (PR), complete response (CR) or stable disease (SD) (centrally assessed according to RECIST 1.1)
-To evaluate the efficacy of Lutathera, compared to active comparator, in terms of duration of response	-DOR: The Duration of Response (DOR) is defined as the time from initially meeting the criteria for response (CR or PR) until the time of progression according to RECIST 1.1 or death due to underlying disease only.
-To evaluate the safety and tolerability of Lutathera	-Safety: Rate of adverse events and laboratory toxicities (scored according to CTCAE grade).
-To evaluate the effect of Lutathera on overall survival	-OS: Time from the randomization date until the day of death due to any cause.
**Exploratory Objectives**	**Endpoints for exploratory objectives**
-To explore the effect of Lutathera on Time to Second Progression (PFS2)	-PFS2: Time from randomization to objective tumor progression on next line treatment or

	death due to any cause (PFS2), assessed locally according to RECIST 1.1.
-To explore Health related quality of life (QoL) as measured by the EORTC QLQ-G.I.NET21, EORTC QLQ-C30, EQ-5D-5L questionnaires	-TTD for Items/Scales not included among key secondary endpoints. Change from baseline in the total score for health status from the EQ-5D questionnaire.
-To explore PK of octreotide long-acting at the high dose of 60 mg	-Steady-state trough plasma concentration of octreotide.
-To explore the safety and efficacy (ORR, PFS, PFS2, DoR, OS) of re-treatment with Lutathera in progressive patients in Lutathera arm	- Safety (Re-treatment): Rate of adverse events and laboratory toxicities during re-treatment with Lutathera.<br>- ORR (Re-treatment): Rate of patients with best overall response of partial response (PR) or complete response (CR) after receiving re-treatment with Lutathera (locally assessed according to RECIST 1.1).<br>- PFS (Re-treatment): Time from the 1st dose of re-treatment with Lutathera to objective tumor progression (locally assessed according to RECIST 1.1) or to death due to any cause.<br>- PFS2 (Re-treatment): Time from randomization to the objective tumor progression (locally assessed according to RECIST 1.1) or to death due to any cause after receiving re-treatment with Lutathera.<br>- DOR (Re-treatment): Time from initially meeting the criteria for response (CR or PR) after receiving re-treatment with Lutathera until the time of progression according to RECIST 1.1 (locally assessed) or death due to underlying disease only.<br>- OS (Re-treatment): Time from the randomization date until the day of death due to any cause in patients who have received re-treatment with Lutathera.

3 Study design

This is a multicenter, stratified, randomized, open-label comparator-controlled, Phase III study in patients ≥15 years with somatostatin receptor positive, well-differentiated G2 and G3, advanced GEP NETs, diagnosed within 6 months prior to screening.

Overall, 222 patients will be randomized (2:1 randomization ratio) to receive treatment with Lutathera (7.4GBq/200 mCi x 4 administrations every 8± 1 weeks; cumulative dose: 29.6 GBq/800mCi) plus octreotide long-acting (30 mg every 8 weeks during Lutathera treatment and every 4 weeks after last Lutathera treatment) or high dose octreotide long-acting (60 mg every 4 weeks). Randomization will be stratified by Grade (G2 vs G3) and tumor origin (pNET vs other origin) (Figure 3-1).

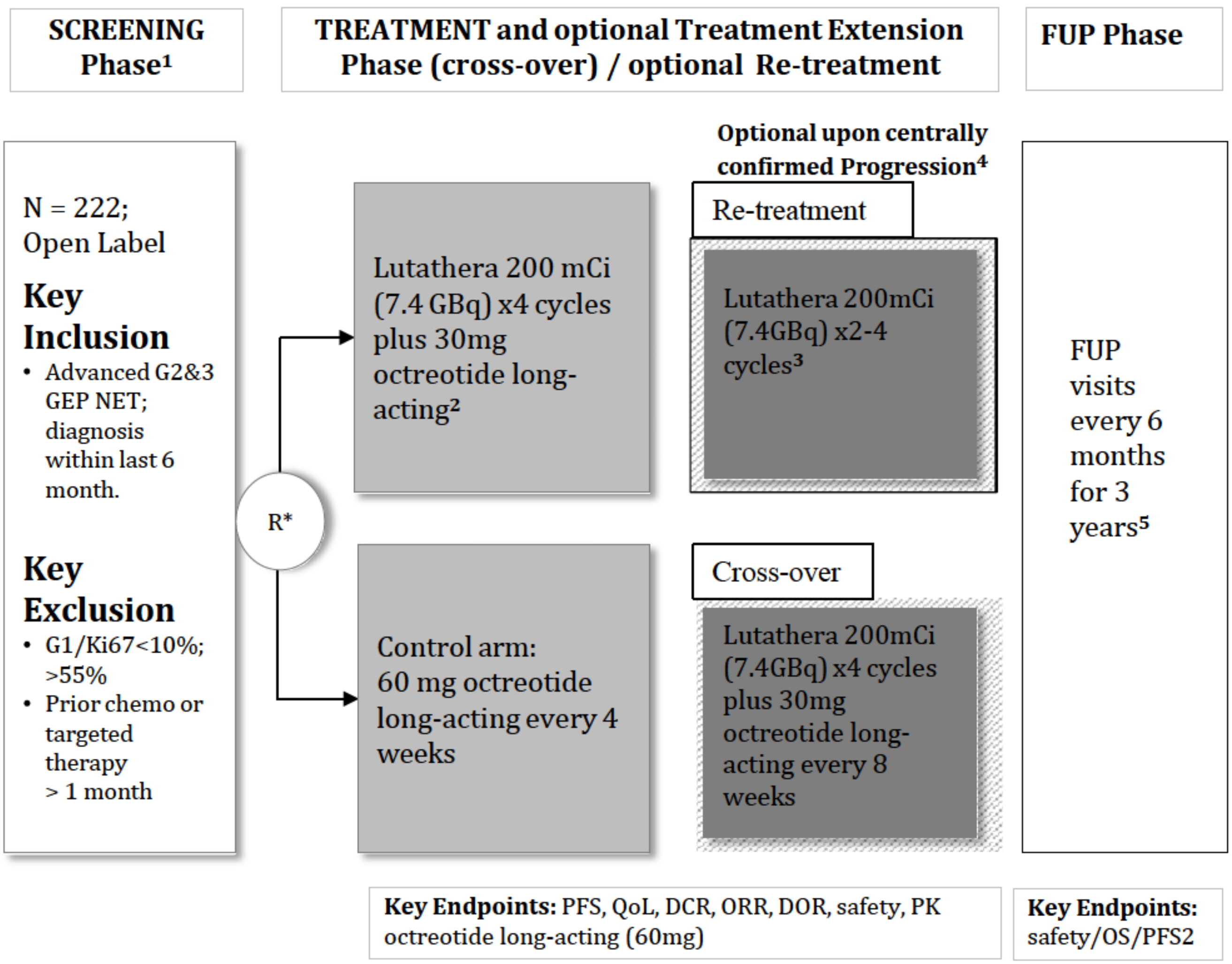

[1]Randomization ratio: 2:1. Stratification Factors: Grade (G2 vs G3) and Tumour Origin (pNET vs other origin).
[2]Octreotide long-acting 30 mg every 8 weeks during Lutathera treatment and every 4 weeks after last Lutathera treatment.
[3]Somatostatin analogues in re-treatment phase are at discretion of investigator
[4]If RECIST progression occurs after Week 72 post the primary end point analysis, the decision to enroll the patient in the cross-over or re-treatment phase will be based on local assessment.
[5]Patients included in the optional cross-over or re-treatment phase will be followed up for 3 years or until EoS, whichever occurs first.

For additional details on the efficacy and safety assessments schedule by visit, see Table 8-3, and Table 8-4.

Figure 3-1. Study Design

The primary endpoint of the study is PFS centrally assessed (target HR=0.5; 90% power, 1-sided α=2.5%). The primary analysis will be performed after 99 PFS events (99 evaluable and centrally confirmed disease progressions or death events) have occurred.

The study consists of a Screening Phase, a Treatment Phase, an optional Treatment Extension Phase (cross-over) / an optional Re-treatment Phase and a Follow up Phase.

3.1 Screening Phase

The screening phase should be shortened as much as possible, in order to treat the patients possibly within 2 weeks after the consent signature.

The randomization should be performed as soon as possible, after all eligibility criteria are verified. Patients under octreotide long-acting before study entry should have their last dose carefully programmed during the screening phase (in case of randomization in the Lutathera arm a washout period of 6 weeks from octreotide long-acting must be observed prior to Lutathera injection).

As Lutathera production and shipment will take circa 12 days to be arranged, if a patient is randomized in the Lutathera arm, Lutathera first dose should be ordered within 24h after randomization.

The baseline CT/MRI scan should be taken possibly on the same day as randomization or immediately before (within 1 week) to ensure that it reflects the disease status closely before the therapy start.

3.2 Treatment Phase

During the Treatment Phase, patients will be followed according to the assessments schedule in Table 8-3, and Table 8-4. Objective tumor response will be assessed at W16±1, W24±1 and then every 12±1 weeks from the randomization date, according to RECIST 1.1 criteria (central + local assessment up to first progression, then only local assessment).

Duration of the Treatment Phase:

- Before the PFS primary analysis:

  The Treatment Phase is not fixed and the patients who are randomized should continue to receive the study treatments until progression or death (patients randomized in the Lutathera arm, will continue octreotide long-acting 30 mg injections after the completion of the 4 Lutathera treatments until progression or death; patients randomized in the octreotide long-acting arm will continue octreotide long-acting 60 mg injections until progression or death).

- After the PFS primary analysis:

  The Treatment Phase becomes fixed and will stop at week 72 (patients randomized in the Lutathera arm, will continue octreotide long-acting 30 mg injections after the completion of the 4 Lutathera treatments until week 72 or earlier in case of progression or death; patients randomized in the octreotide long-acting arm will continue octreotide long-acting 60 mg injections until week 72 or earlier in case of progression or death).

In other words, before the PFS primary analysis (i.e. 99 evaluable and centrally confirmed disease progressions or death events), patients continue the Treatment Phase until progression; after the PFS primary analysis, the Treatment Phase duration is limited to 72 weeks.

At any time during the study (before or after the PFS primary analysis) any progressive patient (based on central imaging assessment) immediately ceases the Treatment Phase and proceeds to the Follow-up Phase (Table 8-3 and Table 8-4). If a patient discontinues Lutathera before centrally confirmed disease progression, the patient should be followed with tumor assessments as per initial CT/MRI schedule until centrally confirmed disease progression.

In addition, patients randomized in the control arm have the option, if eligible, to enroll for post-progression cross-over with Lutathera, see Table 8-4). Control arm patients who cross-over after progression will be allowed to complete the Lutathera treatments after week 72 or to cross-over during the Follow-up Phase, in case of late progression. Furthermore, patients who experience disease progression in the Lutathera arm have the option, if eligible, to enroll for re-treatment phase with Lutathera, see Table 8-3.

Patients included in the optional crossover or re-treatment phase will be followed up at least 6 months and up to 3 years (or until EoS, whatever comes first).

3.3 Optional Treatment Extension Phase (cross-over)

In the control arm, any RECIST progressive patient (based on central assessment) has the option to enroll for post-progression cross-over, upon signature of a new consent, to receive maximum 4 cycles of Lutathera (7.4 GBq/200 mCi x 4 cycles; cumulative dose: 29.6 GBq / 800mCi) plus 30 mg octreotide long acting every 8 weeks.

If RECIST progression occurs after Week 72 post the primary end point analysis, the decision to enroll the patient in cross-over will be based on local assessment.

The cross-over phase will last until the completion of four Lutathera administrations after which patients will undergo EOT visit (at Week 28), and proceed to the follow up phase.

After cross-over phase is complete, patients will be followed up for 3 years or until EoS, whichever occurs first (and at least 6 months after the last Lutathera dose administered during cross-over).

The time window to start cross-over treatment with Lutathera in this study is 4 years after last patient randomized.

3.4 Optional Re-Treatment Phase

In the Lutathera arm, patients with radiological progression based on RECIST criteria as per central assessment have the option to enroll for post-progression re-treatment, upon signature of a new informed consent, to receive 2 to 4 additional cycles of 7.4 GBq/200 mCi of Lutathera. If RECIST progression occurs after Week 72 post the primary end point analysis, the decision to enroll the patient in re-treatment will be based on local assessment.

Patients who have received Lutathera in Optional Treatment Extension Phase (cross-over) are not eligible for re-treatment with Lutathera.

For enrolment in the optional re-treatment phase, patients must undergo SSTR imaging and meet all requirements listed in Section 5.2.2.

After enrolment into re-treatment phase, patients will initially receive 2 administrations of Lutathera 7.4 GBq/200 mCi at 8-week interval. After that, the Investigator should determine if:

- The patient shows evidence of disease stabilization or response (i.e. absence of radiological progression, or assessed by clinical benefit).
- Has shown good tolerance to Lutathera re-treatment.

If the investigator considers this treatment option in the best interest of the participant and if the participant meets these criteria and agrees to continue with further treatment with Lutathera, the Investigator may administer up to 2 additional administrations of Lutathera 7.4 GBq/200 mCi at 8-week interval. A maximum of 4 administrations of Lutathera is allowed during the re-treatment period.

All safety and efficacy assessments in the re-treatment period will be performed locally and following the schedule in initial treatment period (expression of somatostatin receptors on all target lesions will be evaluated locally before re-treatment; there is no central 'real time' assessment of the SRI images, however the images should be submitted to the central imaging center possibly within 1 month for a second review).

During the re-treatment phase, the administration of SSAs is at investigator's discretion.

The re-treatment phase will last until locally confirmed disease progression or until EoS, whichever occurs first. If a patient discontinues Lutathera before locally confirmed disease progression in re-treatment, the patient should be followed with tumor assessments as per initial CT/MRI schedule until locally confirmed disease progression. After re-treatment phase is complete, patients will be followed up for 3 years or until EoS, whichever occurs first (and at least 6 months after the last re-treatment dose in the study).

The time window to start re-treatment in this study is within 4 years after the last patient randomized.

For patients in Lutathera arm who are eligible for re-treatment beyond this window, Lutathera access for re-treatment may be granted via Post Study Drug Supply (PSDS) programs, based on local regulation (see Section 9.2.1).

3.5 Follow up Phase

At the end of the Treatment Phase (or the optional Treatment Extension Phase in case of cross-over, or the optional Re-treatment Phase) or after discontinuation for any cause (including disease progression), all patients will continue to be followed up at least every 6 months and up to 3 years (or until EoS, whatever comes first) to continue data collection for the secondary endpoints of the study, such as long term safety and overall survival.

During the Follow-up Phase, SAEs and adverse events of special interest (AESI) other than secondary hematological malignancies, related to the study treatment and AESI of secondary hematological malignancies irrespective of causality, will be reported. Anti-tumor treatments administered after progression/discontinuation, disease status based on local CT/MRI assessment, and OS data will be collected every 6 months (± 1 month) in both arms (see schedule of assessments in Table 8-3, and Table 8-4).

4 Rationale

4.1 Rationale for study design

This study is justified by the markedly longer PFS and significantly higher response rate in 2nd line G1 and G2 midgut NETs demonstrated in the Lutathera arm of the Phase III NETTER-1 pivotal study (Strosberg, et al., 2017).

The WHO 2010 classification distinguishes G3 NETs (usually ≤55% Ki67) from G3 NECs (usually > 55%), recognizing the NET-specific features of G3-NETs (continuum between G2 and G3 NET) (Rindi 2010, Rindi 2014).

While the Erasmus MC Phase I-II study and the NETTER-1 studies have proven the safety and efficacy of Lutathera in well differentiated Grade 1 and 2 GEP-NETs (Kwekkeboom, et al., 2008; Strosberg, et al., 2017), Grade 3 GEP-NETs still represent high unmet medical need for several reasons:

- Historically, most studies have excluded G3 NET (~20% of all NETs);
- The number of patients diagnosed at G3 NET stage continues to increase (Dasari, et al., 2017);
- There is no universally accepted standard of care for the treatments of G3 NETs. ESMO (Oberg, Knigge, Kwekkeboom, Perren, & Group, 2012), ENET (Perren, et al., 2017), NANETS (Kunz, et al., 2013) and NET NCCN (Kulke, et al., 2015) guidelines outline chemotherapy as primary treatment; however physicians' practice may vary (SSA and/or chemotherapy used in 1st and 2nd line).
- Based on experts' considerations and from the NORDIC NEC study, a Ki-67 cutoff between 50% and 60% has been proposed as the minimum level that should be considered to use platinum-based chemotherapy. Therefore, it appears reasonable to manage well-differentiated G 3 and G 2 NET patients similarly (Vélayoudom-Céphise, et al., 2013; Heetfeld, et al., 2015). In addition, prognosis and response rate of well-differentiated G3 NET seem to be close to well differentiated G 1/2 NET with a worse overall survival (Coriat, Walter, Terris, Couvelard, & Ruszniewski, 2016).
- Efficacy of current 1st line treatments appear to be modest with low ORR with limited evidence of PFS/OS benefit (Coriat, Walter, Terris, Couvelard, & Ruszniewski, 2016). In addition, in non-pancreatic NET G-3, no chemotherapy regimen should be considered as a standard of first-line care, considering the very small amount of data available.

Therefore, in the current NETTER-2 study an advanced GEP-NET patient population of G2 with Ki67 ≥ 10% and G3 with Ki67 ≤ 55% has been included to evaluate a potential new treatment option for these patients.

In accordance with the recently finalized guidance for industry on considerations for the inclusion of adolescent patients in adult oncology clinical trials and the consensus expert opinion presented by the European multi-stakeholder platform ACCELERATE to include adolescents in adult oncology clinical trials when the histology and biologic behavior of the cancer under investigation is the same in, or the molecular target of the drug is relevant to, cancers in both adult and adolescent patients (FDA guidance), this study will include adolescents ≥15 years and >40 kg BW. Even though the number of adolescents ≥15 years fulfilling the inclusion criteria for this study is very small, the high unmet medical need in this population is justifying their enrollment. It is not

expected that adolescents below the age of 15 years will present with the disease meeting the inclusion criteria for this study.

Due to the radioactive nature of Lutathera and its method of infusion, and similarly to the NETTER-1 trial design, it is not possible to implement a fully double-blinded design for this study, however a central, blinded, real-time IRC (Independent Review Committee) assessment is implemented to ensure an independent evaluation of the tumor response according to RECIST 1.1 criteria.

Randomization will be stratified by Grade (G2 vs G3) and tumor origin (pNET vs other origin), preventing imbalance between treatment groups for known factors that influence prognosis.

For this trial a 2:1 randomization design is chosen to allow patients to have a higher chance of getting the treatment which is anticipated to be more effective. For the same reason and to minimize the possible higher drop-out rate in the control arm, patients will be offered to cross-over to Lutathera after centrally confirmed RECIST progression (or locally confirmed if it occurs after Week 72 post the primary end point analysis).

Patients randomized in the Lutathera arm will also be offered optional re-treatment with additional 2-4 Lutathera doses/cycles after centrally confirmed RECIST progression (or locally confirmed if it occurs after Week 72 post the primary end point analysis), based on the physician judgment of potential clinical benefit. Overall, therapeutic options in patients who progress after PRRT treatment are limited. Based on a meta-analysis (Strosberg et al., 2021) including patients with progressive GEP-NETs (n=560) who received re-treatment with Lutathera (one to six doses during re-treatment, majority of patients received 200 mCi per administration), the clinical benefit of re-treatment was supported by a median PFS of 12.52 months (available in 414 patients), median OS of 26.78 months from the start of re-treatment (available in 194 patients), and DCR of 71% (available in 347 patients). The safety profile of Lutathera re-treatment was similar to initial treatment. These data indicate that re-treatment with Lutathera may be considered as a treatment approach to maximize benefit without compromising on safety and with a careful assessment of clinical status of each individual patient.

4.2 Rationale for dose/regimen and duration of treatment

The Lutathera arm dose/regimen of the NETTER-2 study is identical to the established regimen used in the Erasmus MC Phase I/II study (Kwekkeboom, et al., 2008) and in the Phase III NETTER-1 pivotal study (Strosberg, et al., 2017) with Lutathera cumulative dose of 29.6 GBq (800 mCi), divided into 4 administrations every 8±1 weeks, plus octreotide long-acting (30 mg) every 4 weeks.

Lutathera was approved in Europe for the treatment of unresectable or metastatic, progressive, well differentiated (G1 and G2) GEP-NETs and in the USA for the treatment of somatostatin receptor positive GEP-NETs based on the results of the pivotal Phase III NETTER-1 study and data from 1,214 patients with somatostatin receptor-positive tumors, including GEP-NETS, who received Lutathera at the Erasmus Medical Centre in the Netherlands.

Treatment with Lutathera resulted in markedly longer progression-free survival and a significantly higher response rate than high-dose octreotide long-acting among patients with advanced midgut neuroendocrine tumors. Preliminary evidence of an overall survival benefit was seen in an interim

analysis; confirmation will be required in the planned final analysis. Clinically significant myelosuppression occurred in less than 10% of patients in the Lutathera group. The PFS-prolongation was similar in the two Ki67 strata (≤2% and 3-20%) suggesting a potential benefit for patients with advanced G2 and 3 GEP-NET to be included in the NETTER-2 study.

Optional re-treatment with additional doses of Lutathera will be offered to patients treated in the Lutathera arm upon disease progression, based on recent published data on re-treatment in GEP-NETs (Strosberg et al., 2021). The candidate patients for re-treatment with Lutathera in this protocol, are patients with centrally documented tumor progression in Lutathera arm, who have completed the 4 doses/cycles of Lutathera during the treatment phase, had CR/PR/SD as best response for at least 6 months (-2 weeks window is allowed) after the 4th Lutathera dose, and had tolerated the treatment (according to criteria defined in Section 5.2.2). Patients will be offered to receive initially two Lutathera doses in re-treatment period (Section 4.3). Based on the physician judgment of clinical benefit derived from the first 2 doses, patients may receive up to 2 additional doses of Lutathera (criteria for additional doses are listed in Section 4.3). A maximum of 4 doses of Lutathera is allowed during the re-treatment period. The dose level assessed in the re-treatment portion of this study will be the same as the approved level of 7.4 GBq per cycle, based on the published meta-analysis data that showed that majority of patients received this dose (Strosberg et al., 2021).

4.3 Rationale for choice of control drug

The control arm dose/regimen of the NETTER-2 study is identical to the dose/regimen used in the NETTER-1 study (octreotide long-acting (60 mg) every 4 weeks).
The clinical experience with octreotide is extensive, but there is currently a lack of data in the use of somatostatin analogues in G3 GEP-NET patients.

Patients in the comparator arm of the present NETTER-2 study will receive 60 mg octreotide long-acting at 4-weeks intervals. The dose is supported by findings of Broder et al. (2015), who performed a systematic review of the literature to analyze the anti-proliferative benefit of somatostatin analogs. They confirmed that doses of >30 mg octreotide long-acting are commonly used by clinical experts for symptom and tumor progression control in NET patients. This dose (60 mg) was also tested in comparator arm of the pivotal Phase III NETTER-1 study (Strosberg, et al., 2017). In current clinical practice, it is likely that even a higher percentage of patients receive >30 mg of Octreotide LAR (Anthony & Vinik, 2011; Joseph, et al., 2010; Wolin, 2012; Broder, Beenhouwer, Strosberg, Neary, & Cherepanov, 2015). The 4-week interval injections of 60 mg octreotide long-acting is a higher dose than the 4-week interval injections with 20 mg or 30 mg, which is presently the registered dose for Sandostatin® LAR Depot. With this treatment, the majority of symptomatic patients show an improvement in QoL, and most patients obtain temporary stable disease based on CT scans (Faiss, et al., 1999; Faiss, et al., 2003; Rinke, et al., 2009; Ludlam & Anthony, 2011; Anthony & Vinik, 2011; Wolin, 2012; Broder, Beenhouwer, Strosberg, Neary, & Cherepanov, 2015).

Furthermore, according to the Phase III NETTER-1 study results, patients with metastatic midgut tumors progressive after standard dose (30 mg) SSAs treated in the control arm of the study with high dose (60 mg) octreotide long-acting had a median time free from relapse of 8.4 months

(Strosberg, et al., 2017) without notable side effects, confirming that this regimen has anti-tumor benefit and is well tolerated.

In a clinical study published by Astruc et al. (Astruc, et al., 2005), in which healthy subjects were treated with 20 mg and 60 mg of Octreotide LAR, the PK data showed that the plasma exposure was dose-proportional and the treatment was well tolerated at both doses.
Available pharmacokinetic (PK) data in children at 40 mg Octreotide LAR show that the plasma exposure, corrected for the dose, is similar in adults and young patients. Moreover, Octreotide LAR was well tolerated in children at 40 mg. It should be noted that the same dose was given to <12 and >12 years old patients.
Therefore, in accordance with available PK data and in line with FDA guidance for inclusion of adolescents in the adult oncology trials and the consensus expert opinion presented by the European multi-stakeholder platform ACCELERATE it is considered acceptable to include adolescents in the NETTER-2 study at the same fixed dose administered in adults, considering a population ≥ 15 y and > 40 kg.

Based on currently available data of high dose somatostatin analogues, the use as a comparator in this study, at 60 mg dosage, is justified considering the high unmet medical need in a population not candidate for chemo or targeted therapy in the investigator opinion.

4.4 Purpose and timing of interim analyses

At the time of the PFS primary analysis, an estimate of overall survival will be calculated in terms of hazard ratio (point estimate) and 95% confidence interval.

4.5 Risks and benefits

Neuroendocrine tumors (NETs) represent a small proportion of cancers but are increasing in incidence due to incidental diagnosis. Prognosis for grade 1 and localized NETs has steadily improved, however patients with distant and/or grade 3 NETs continue to fare poorly (Sackstein, O'Neil, Neugut, Chabot, & Fojo, 2018).

Patient management poses a significant challenge because of the heterogeneous clinical presentations and varying degrees of aggressiveness.

Randomization will be stratified by tumor origin (pNET vs other origin) and Grade (G2 vs G3), preventing imbalance between treatment groups for known factors that influence prognosis.

The population of G2 and G3 GEP-NET patients included in the NETTER-2 study is not candidate for chemo or targeted therapy in the investigator opinion. G3 GEP-NET patients with Ki67 ≤ 55 are a category of G3 neuroendocrine neoplasm (NEN) where tumors retain their well-differentiated characteristics, continue to express somatostatin receptors, but have a higher proliferation rate then the majority of GEP-NETs (Rindi, et al., 2018). Aggressive G2 GEP-NETs patients with Ki67 range 10% to 20% share with G3 GEP-NETs the greater likelihood of poor prognosis, and limited treatment options. Despite advances in medical therapy for these tumors, there is still a high unmet medical need to control tumor growth, especially for G3 GEP-NETs (~10-20% of all NETs), which historically have been excluded from most studies (Sorbye, et al., 2019; Coriat, Walter, Terris, Couvelard, & Ruszniewski, 2016; Dasari, et al., 2017).

The most important risk mitigating factor for GEP-NET patients who are considered for treatment with Lutathera, is the ability to use receptor scintigraphy imaging to select those patients who are

most likely to respond to PRRT, and to exclude patients who would not benefit from the treatment because their tumors do not express high enough levels of the somatostatin receptor.

Based on the results achieved in other PRRT studies using ^{177}Lu-dotatate (Strosberg 2017; 2018 Brabander 2016), the major expected benefits to patients treated with Lutathera include the high probability of extending progression free survival, increasing overall survival and increasing time to deterioration of quality of life.

For this trial a 2:1 randomization design is chosen to allow patients to have a higher chance of getting the treatment which is anticipated to be more effective. For the same reason and to minimize the possible higher drop-out rate in the control arm, patients will be offered to cross-over to Lutathera after centrally confirmed RECIST progression (or locally confirmed if it occurs after Week 72 post the primary end point analysis).

The highest risks arising from treatment with Lutathera are radiation toxicities affecting either bone marrow or kidney function (Bergsma, et al., 2016a; Bergsma, et al., 2016b).

Kidney function risks are largely eliminated by the co-infusion of 2.5% Lys-Arg solutions during administration of Lutathera, which reduces the radiation dose to the kidney by approximately 45% (Rolleman, Valkema, Jong, Kooij, & Krenning, 2003). In the NETTER-1 trial no evidence of renal toxicities occurred within the period of observation (Strosberg, et al., 2017).

Bone marrow toxicity may occur in two forms: 1) acute toxicity that occurs after administration of Lutathera, during the treatment period; and 2) delayed toxicity that occurs in 1.5 to 2% of patients. The potential observed effects of short-term bone marrow toxicity are anemia, thrombocytopenia, and neutropenia which are usually mild and transient. In the NETTER-1 trial, grade 3 or 4 neutropenia, thrombocytopenia, and lymphopenia occurred in 1%, 2%, and 9% of patients (Strosberg, et al., 2017). Where hematological toxicity occurs, a trend towards stabilization followed by improvement in patients with longer follow-up is observed. It is important to note that the lymphocyte toxicity observed following PRRT is not a major concern regarding risk of infections, since only B lymphocytes are affected, a subtype which is not directly involved in infection defense (Sierra, et al., 2009).

There is evidence that males may be at risk of decreased spermatogenesis following PRRT with ^{177}Lu-dotatate. In a study by (Teunissen, et al., 2009), a significant decrease of mean serum inhibin B levels was observed in male patients (N=35) treated with ^{177}Lu-dotatate. It has been demonstrated that serum inhibin B levels are positively correlated with spermatogenic status and sperm count (Pierik, Vreeburg, Stijnen, De Jong, & Weber, 1998). The study (Teunissen, et al., 2009) also found recovery to almost pretreatment levels after 24 months. The potential long-term genetic damage to spermatogenic cells has not been studied.

Women and men should not procreate until six months after the end of their last treatment with Lutathera. However, due to additional exposure from CT scans taken during the study, women in both arms should also not procreate during the whole treatment period of the study. Women of child bearing potential and sexually active males must be informed that taking the study treatment may involve unknown risks to the fetus if pregnancy were to occur during the study, and agree that in order to participate in the study they must adhere to the contraception requirements outlined in the exclusion criteria and in Appendix 1. If there is any question that the subject will not reliably comply, they should not be entered or continue in the study.

Based on the FDA guidance for inclusion of adolescents in the adult oncology trials and the consensus expert opinion presented by the European multi-stakeholder platform ACCELERATE it is recommended that adolescents can receive the same dose administered in adults if there is no relevant effect of body size on pharmacokinetics, which is the case for both, Lutathera and octreotide long-acting. Based on the similar clinical presentation of the disease under evaluation in adolescents, the following inclusion criteria have been chosen for this study: ≥15 years old and >40 kg BW (aligned with the BW recommendation from the FDA guidance).

With regards to re-treatment with Lutathera, the results of a meta-analysis of patients with progressive GEP-NETs who received re-treatment with peptide receptor radionuclide therapy (PRRT), are supportive of administration of additional Lutathera doses after progression on initial treatment. It was shown that the safety profile of Lutathera re-treatment was similar to initial treatment (Strosberg et al., 2021). In this meta-analysis, additional treatment courses with Lutathera ranged from one to six doses, and majority of patients received 200 mCi per administration. Hematologic grade 3/4 AEs were reported in 9% of patients who received PRRT re-treatment. Notably, AML and MDS occurred in <1% of patients who received re-treatment with Lutathera, comparable to the incidence observed with initial PRRT. Re-treatment with Lutathera provided encouraging median PFS. This data indicates that re-treatment with Lutathera can be offered to patients to maximize benefit without compromising on safety and with a careful assessment of clinical status of each individual patient.

Dose modifying toxicity rules have been implemented in this study taking into account the higher GEP-NET severity in the NETTER-2 study compared to the approved indication. The administration of a full Lutathera dose (200 mCi/7.4 GBq) if a toxicity is resolved will maximize the potential treatment effects and maintain a positive benefit/risk ratio.

To ensure the safety of the subjects in this trial, appropriate eligibility criteria and study procedures, as well as close clinical monitoring, following dose modifying toxicity rules, and Steering Committee study oversight are included in this protocol.

In light of all the above-mentioned elements, the benefit-risk balance of Lutathera within the overall clinical context defined in the clinical trial appears to weight in favor of patient's benefit.

4.6 Rationale for public health emergency mitigation procedures

In the event of a public health emergency as declared by local or regional authorities (i.e. pandemic, epidemic or natural disaster), mitigation procedures may be required to ensure participant safety and trial integrity and are listed in relevant sections of the study protocol. Notification of the public health emergency should be discussed with Sponsor prior to implementation of mitigation procedures and permitted/approved by local or regional health authorities and ethics committees as appropriate.

5 Population

In this study, safety and efficacy of treatment with Lutathera plus octreotide long-acting (30 mg) versus high dose octreotide long-acting (60 mg) is evaluated in patients ≥15 years old with somatostatin receptor positive, well differentiated G2 (Ki67 index ≥10%) and G3 (Ki67 ≤ 55%) advanced GEP-NETs. Patients with documented RECIST progression to previous treatments for the current GEP-NET at any time prior to randomization are not eligible to participate in this study.

5.1 Inclusion criteria

Subjects eligible for inclusion in this study must meet **all** of the following criteria:

1. Presence of metastasized or locally advanced, inoperable (curative intent) histologically proven, well differentiated Grade 2 or Grade 3 gastroenteropancreatic neuroendocrine (GEP-NET) tumor diagnosed within 6 months prior to screening.
2. Ki67 index ≥10 and ≤ 55%.
3. Patients ≥15 years of age and a body weight of >40 kg at screening.
4. Expression of somatostatin receptors on all target lesions documented by CT/MRI scans, assessed by any of the following somatostatin receptor imaging (SRI) modalities within 3 months prior to randomization: [68Ga]-DOTA-TOC (e.g. Somakit-TOC®) PET/CT (or MRI when applicable based on target lesions) imaging, [68Ga]-DOTA-TATE PET/CT (or MRI when applicable based on target lesions) imaging (e.g. NETSPOT®), Somatostatin Receptor scintigraphy (SRS) with [111In]-pentetreotide (Octreoscan® SPECT/CT), SRS with [99mTc]-Tektrotyd, [64Cu]-DOTA-TATE PET/CT (or MRI when applicable based on target lesions) imaging.
5. The tumor uptake observed in the target lesions must be > normal liver uptake.
6. Karnofsky Performance Score (KPS) ≥60.
7. Presence of at least 1 measurable site of disease.
8. Patients who have provided a signed informed consent form to participate in the study, obtained prior to the start of any protocol related activities

5.2 Exclusion criteria

Subjects meeting any of the following criteria are not eligible for inclusion in this study.

1. Creatinine clearance <40 mL/min calculated by the Cockroft Gault method.
2. Hb concentration <5.0 mmol/L (<8.0 g/dL); WBC $<2x10^9$/L (2000/mm^3); platelets $<75x10^9$/L ($75x10^3$/mm^3).
3. Total bilirubin >3 x ULN.
4. Serum albumin <3.0 g/dL unless prothrombin time is within the normal range.
5. Pregnancy or lactation.

6. A) Women of child-bearing potential, defined as all women physiologically capable of becoming pregnant, are not allowed to participate in this study UNLESS they are using highly effective methods of contraception throughout the study treatment period (including cross-over and re-treatment, if applicable) and for 7 months after study drug discontinuation. Highly effective contraception methods include:

   - True abstinence when this is in line with the preferred and usual lifestyle of the patient. Periodic abstinence (e.g., calendar, ovulation, symptothermal, post-ovulation methods), declaration of abstinence for the duration of exposure to IMP, and withdrawal are not acceptable methods of contraception.
   - Male or female sterilization
   - Combination of any two of the following (a+b or a+c or b+c):

   a. Use of oral, injected, or implanted hormonal methods of contraception. In case of use of oral contraception, women should be stable on the same pill for a minimum of 3 months before taking study treatment.

   b. Placement of an intrauterine device (IUD) or intrauterine system (IUS)

   c. Barrier methods of contraception: condom or occlusive cap (diaphragm or cervical/vault caps) with spermicidal foam/gel/film/cream/vaginal suppository. Post-menopausal women are allowed to participate in this study. Women are considered post-menopausal and not of child bearing potential if they have had 12 months of natural (spontaneous) amenorrhea with an appropriate clinical profile (e.g. age appropriate, history of vasomotor symptoms) confirmed by a high follicle stimulating hormone (FSH) level, or have had surgical bilateral oophorectomy (with or without hysterectomy) or tubal ligation at least six weeks prior to screening. In the case of oophorectomy alone, only when the reproductive status of the woman has been confirmed by follow up hormone level assessment is she considered not of child bearing potential.

   B) Sexually active male patients, unless they agree to remain abstinent (refrain from heterosexual intercourse) or be willing to use condoms and highly effective methods of contraception with female partners of childbearing potential or pregnant female partners during the treatment period (including cross-over and re-treatment, if applicable) and for 4 months after study drug discontinuation. In addition, male patients must refrain from donating sperm during this same period.

7. Peptide receptor radionuclide therapy (PRRT) at any time prior to randomization in the study.
8. Documented RECIST progression to previous treatments for the current GEP-NET at any time prior to randomization.
9. Patients for whom in the opinion of the investigator other therapeutic options (eg chemo-, targeted therapy) are considered more appropriate than the therapy offered in the study, based on patient and disease characteristics.
10. Any previous therapy with Interferons, Everolimus (mTOR-inhibitors), chemotherapy or other systemic therapies of GEP-NET administered for more than 1 month or within 12 weeks prior to randomization in the study.
11. Any previous radioembolization, chemoembolization and radiofrequency ablation for GEP-NET.

12. Any surgery within 12 weeks prior to randomization in the study.
13. Known brain metastases, unless these metastases have been treated and stabilized for at least 24 weeks, prior to screening in the study. Patients with a history of brain metastases must have a head CT or MRI with contrast to document stable disease prior to randomization in the study.
14. Uncontrolled congestive heart failure (NYHA II, III, IV). Patients with history of congestive heart failure who do not violate this exclusion criterion will undergo an evaluation of their cardiac ejection fraction prior to randomization via echocardiography. The results from an earlier assessment (not exceeding 30 days prior to randomization) may substitute the evaluation at the discretion of the Investigator, if no clinical worsening is noted. The patient's measured cardiac ejection fraction in these patients must be ≥40% before randomization.
15. QTcF > 470 msec for females and QTcF > 450 msec for males or congenital long QT syndrome.
16. Uncontrolled diabetes mellitus as defined by hemoglobin A1c value > 7.5%.
17. Hyperkaleamia >6.0 mmol/L (CTCAE Grade 3) which is not corrected prior to study enrolment.
18. Any patient receiving treatment with short-acting octreotide, which cannot be interrupted for 24 h before and 24 h after the administration of Lutathera, or any patient receiving treatment with SSAs (eg octreotide long-acting), which cannot be interrupted for at least 6 weeks before the administration of Lutathera.
19. Patients with any other significant medical, psychiatric, or surgical condition, currently uncontrolled by treatment, which may interfere with the completion of the study.
20. Prior external beam radiation therapy to more than 25% of the bone marrow.
21. Current spontaneous urinary incontinence.
22. Other known co-existing malignancies except non-melanoma skin cancer and carcinoma in situ of the uterine cervix, unless definitively treated and proven no evidence of recurrence for 5 years.
23. Patient with known incompatibility to CT Scans with I.V. contrast due to allergic reaction or renal insufficiency. If such a patient can be imaged with MRI, then the patient would not be excluded.
24. Hypersensitivity to any somatostatin analogues, the IMPs active substance or to any of the excipients.
25. Patients who have participated in any therapeutic clinical study/received any investigational agent within the last 30 days.

5.2.1 Eligibility Criteria for Optional Treatment Extension Phase (cross-over)

Any centrally confirmed RECIST progressive patient has the option to enroll for post-progression cross-over and receive maximum 4 cycles of Lutathera (7.4 GBq/200 mCi x 4 cycles; cumulative dose: 29.6 GBq / 800mCi) plus octreotide long-acting (30 mg every 8 weeks), provided that the following criteria are met:

- Patients have provided a signed informed consent form to participate in the study treatment extension phase, obtained prior to the start of any related procedures.
- Expression of somatostatin receptors on all target lesions (based on post-progression new baseline assessment) documented by CT/MRI scans (Note: Cross-over Eligibility CT/MRI scan doesn't need to be repeated if performed within the previous 12 weeks), assessed by any of the following somatostatin receptor imaging (SRI) modalities within 3 months prior to Lutathera treatment: [68Ga]-DOTA-TOC (e.g. Somakit-TOC®) PET/CT imaging (or MRI when applicable based on target lesions), [68Ga]-DOTA-TATE PET/CT (or MRI when applicable based on target lesions) imaging (e.g. NETSPOT®), Somatostatin Receptor scintigraphy (SRS) with [111In]-pentetreotide (Octreoscan® SPECT/CT), SRS with [99mTc]-Tektrotyd, [64Cu]-DOTA-TATE PET/CT imaging (or MRI when applicable based on target lesions).
- All protocol inclusion/exclusion criteria are met (see Sections 5.1 and 5.2).
- The time window to start cross-over is 4 years after last patient randomized.
- Note: Prior each Lutathera administration a 6-week washout period from last octreotide long-acting administration has to be applied.

If RECIST progression occurs after Week 72 post the primary end point analysis, the decision to enroll the patient in cross-over will be based on local assessment.

Images (Cross-over Eligibility SRI and CT or MRI scan) will be also submitted to the central imaging center for a second review: there is no central 'real time' assessment of the Cross-Over Eligibility SRI uptake; however, the images should be submitted to the central imaging center possibly within 1 month. Images submission is not required for screening failure patients.

5.2.2 Eligibility Criteria for Optional Re-Treatment Phase

In the Lutathera arm, patients with radiological progression based on RECIST criteria in central assessment, have the option to enroll for post-progression re-treatment, upon signature of a new informed consent, to receive 2 to 4 additional cycles of 7.4 GBq/200 mCi of Lutathera.

For enrolment in the optional re-treatment phase, patients must meet all following requirements:

- Randomized to Lutathera arm, received 4 doses of Lutathera in initial treatment and experienced either disease stabilization (SD) or objective response (PR/CR) (centrally confirmed) for at least 6 months after the 4th Lutathera dose. If a patient's response has changed from PR/CR to SD within 6 months after the 4th Lutathera dose, the patient is still eligible for re-treatment, provided there is no documented progression within 6 months. A window of -2 weeks is allowed for the confirmation of 6 months of SD/PR/CR response.
- Lutathera was overall well tolerated; no recorded SAEs related to Lutathera that were not resolved before the next Lutathera dose and led to treatment interruption.
- After the SD/PR/CR response, experienced radiological RECIST progression (centrally confirmed). If RECIST progression occurs after Week 72 post the primary end point analysis, the decision to enroll the patient in re-treatment will be based on local assessment.
- Patient did not receive any other systemic treatment for GEP-NET after progression (somatostatin analogues are allowed).

- Investigator's agreement that re-treatment is in the best interest for the patient
- Patients have provided a signed informed consent form to participate in the re-treatment phase, obtained prior to the start of any related procedures.
- Time window to start re-treatment: 4 years after last patient randomized (see Section 9.2).
- Expression of somatostatin receptors on all target lesions (based on post-progression new baseline assessment) documented by CT/MRI scans (Note: Re-treatment Eligibility CT/MRI scan doesn't need to be repeated if performed within the previous 12 weeks), assessed by any of the following somatostatin receptor imaging (SRI) modalities within 3 months prior to Lutathera re-treatment: [68Ga]-DOTA-TOC (e.g. Somakit-TOC®) PET/CT imaging (or MRI when applicable based on target lesions), [68Ga]-DOTA-TATE PET/CT (or MRI when applicable based on target lesions) imaging (e.g. NETSPOT®), Somatostatin Receptor scintigraphy (SRS) with [111In]-pentetreotide (Octreoscan® SPECT/CT), SRS with [99mTc]-Tektrotyd, [64Cu]-DOTA-TATE PET/CT imaging (or MRI when applicable based on target lesions).

Images (Re-treatment Eligibility SRI and CT or MRI scan) will be also submitted to the central imaging center for a second review: there is no central 'real time' assessment of the Re-treatment Eligibility SRI uptake, however the images should be submitted to the central imaging center possibly within 1 month. Images submission is not required for screening failure patients.

6 Treatment

In this study, approximately 222 patients with advanced G2-3 GEP-NET will be randomized (2:1 randomization ratio) to receive treatment with Lutathera (7.4 GBq or 200 mCi x 4 administrations every 8± 1 weeks; cumulative dose: 29.6 GBq or 800 mCi) plus octreotide long-acting standard dose (30 mg every 8 weeks during Lutathera treatment and every 4 weeks after last Lutathera treatment) or octreotide long-acting high dose (60 mg every 4 weeks).

The investigational drug product Lutathera® (^{177}Lu-DOTA^{0}-Tyr^{3}-Octreotate) will be provided by the Sponsor. The Sponsor will also provide the 2.5% Lys-Arg sterile amino acid solution for infusion (if it can't be compounded at the hospital Pharmacy), as well as octreotide long-acting (Sandostatin® LAR Depot) for the entire duration of the Treatment Phase (and optional Treatment Extension Phase) of the study. For the optional Re-treatment Phase, the Sponsor will provide Lutathera and 2.5% Lys-Arg sterile amino acid solution; octreotide-long acting administration is not mandatory and is at investigator discretion. Patients will switch to prescribed drugs in the follow up phase.

Anti-emetics, SRI imaging agents, short-acting octreotide or any other supportive care medication will not be supplied by the Sponsor.

6.1 Study treatments

6.1.1 Investigational and control drugs

The investigational and control drugs supplied by Sponsor are listed in Table 6-1.

Table 6-1. Investigational and control drug

Investigational/ Control Drug (Name and Strength)	Pharmaceutical Dosage Form	Route of Administration	Supply type	Supplier	Study arm
Lutathera®	*Radiopharmaceutical solution for infusion (7.4 GBq of Lutathera per 30 ml vial)*	*Intravenous use*	*Open label; vials*	*Sponsor*	*Lutathera*
2.5% Lys-Arg sterile *amino acid solution*	25g L-lysine-HCl and 25g L-arginine-HCl in 1000 mL	*Intravenous use*	*Open label; flex bags*	*Sponsor**	*Lutathera*
Octreotide long-acting (Sandostatin LAR® Depot)	*For injectable suspension: strengths 10 mg per 6 mL, 20 mg per 6 mL, or 30 mg per 6 mL vials*	*Intragluteal injection*	*Open label, vials*	*Sponsor*	*Lutathera; Control*

*Sponsor will provide the 2.5% Lys-Arg sterile amino acid solution for infusion (if it can't be compounded at the hospital Pharmacy).

6.1.1.1 Lutathera arm

6.1.1.1.1 Investigational Drug Product: Lutathera® (^{177}Lu-Dotatate)

Lutathera is a sterile radiopharmaceutical supplied as a ready-to-use solution for infusion containing ^{177}Lu-DOTA^{0}-Tyr^{3}-Octreotate as Drug Substance with a volumetric activity of 370 MBq/mL at reference date and time (calibration time (tc)).

On the day of Lutathera treatment, an intravenous bolus of anti-emetic is given. For renal protection, a sterile amino acids solution is infused 30 minutes before the start of Lutathera, and continues for a total of 4 h. See further details in Section 6.6.

Lutathera is a radiopharmaceutical solution for infusion supplied as a ready-to-use product. The only Quality Control (QC) tests that must be performed at the clinical site are; 1) confirm correct product certificate; 2) determine total radioactivity; 3) confirm visual appearance. Since Lutathera is manufactured in centralized GMP facilities, the majority of required QC tests are performed before product shipment. A batch release certificate of the product will be sent to the investigational centres. This batch release certificate is provided by the Qualified Person (QP) of the manufacturing site to ensure that the product is suitable for administration and that it meets the specifications indicated in the product leaflet.

Manufacturing site prepares single doses calibrated within the range of 7.4 GBq ± 10 % (200 mCi) between t0+6h and t0+52h after the end of production. Certificate of release reports both the exact activity provided and the time when this activity is reached. The total amount of radioactivity per single dose vial is 7,400 MBq at the date and time of infusion. Given the fixed volumetric activity of 370 MBq/ml at the date and time of calibration, the volume of the solution is adjusted between 20.5 ml and 25.0 ml in order to provide the required amount of radioactivity at the date and time of infusion.

The composition of the drug product is listed in Table 6-2 below.

Table 6-2. Composition of the Lutathera® Drug Product (per vial)

Component	Content (Unit and/or percentage)		Function	Quality standards
	V= [redacted] mL (min volume)	V= [redacted] mL (max volume)		
Drug Substance: ^{177}Lu-DOTA^{0}-Tyr^{3}-Octreotate (volumetric activity)	[redacted] MBq/mL ± [redacted] % at Tc (EoP)		Drug Substance	-
Activity at Injection time	[redacted] GBq ± [redacted] %		-	-
$^{176+177}$Lu-DOTA^{0}-Tyr^{3}-Octreotate[2]	[redacted] µg	[redacted] µg	-	NA[1]
DOTA-Tyr^{3}-Octreotate	[redacted] µg	[redacted] µg	Chemical precursor	-
Excipients*				
Acetic acid	[redacted] mg	[redacted] mg	pH adjuster	In-house
Sodium acetate	[redacted] mg	[redacted] mg	pH adjuster	In-house

Component	Content (Unit and/or percentage)		Function	Quality standards
	V= [redacted] mL (min volume)	V= [redacted] mL (max volume)		
Gentisic acid	[redacted] mg	[redacted] mg	RSE	In-house
Ascorbic acid	[redacted] mg	[redacted] mg	RSE	In-house
Diethylene triamine pentaacetic acid (DTPA)	[redacted] mg	[redacted] mg	Sequestering agent	In-house
Sodium chloride (NaCl)	[redacted] mg	[redacted] mg	Isotonizing agent	BP monograph "Sodium Chloride infusion"
Sodium hydroxyde (NaOH)	[redacted] mg	[redacted] mg	pH adjuster	Ph. Eur. 0677
Water for injection	Ad [redacted] mL**	Ad [redacted] mL**	Solvent	Ph. Eur. 0169/ USP

Tc= calibration time =EOP: End of Production = t_0 = activity measurement of the first vial
RSE: Radiation Stability Enhancer
[1]*The synthesis of the Drug Substance and formulation into the Drug Product are parts of a continuous process and therefore the Drug Substance is not isolated*
[2] *$^{176+177}$Lu-DOTA^0-Tyr^3-Octreotate represents the fraction of peptide labeled to lutetium (radioactive and non-radioactive lutetium) and corresponds to the sum of ^{176}Lu-DOTA^0-Tyr^3-Octreotate and ^{177}Lu-DOTA^0-Tyr^3-Octreotate*
**Indicated values calculated at calibration times based on ^{177}Lu specific activity of 740 GBq/mL (20 Ci/mg) at labeling time and a mean synthesis yield of 80 % and radiochemical purity ≥ 97 %.*
*** This amount includes as well the water for injection filled in the bulk solution bottle (0.5 ± 0.1 mL) and in the primary packaging (0.20 ± 0.02 mL) during the sterilization process as added amount is considered negligible.*

Additional information on the study drug preparation, radioprotection notes and recommendations for treated patients are provided in Appendix 2, Appendix 4, Appendix 5, Appendix 6, Appendix 8 and in the pharmacy manual.

6.1.1.1.2 Concomitant Treatment: 2.5% Lys-Arg sterile amino acid solution

In the present study patients randomized in Lutathera arm will receive concomitant 2.5% Lys-Arg solution for kidney protection (see Table 6-5). For renal protection purpose, the 2.5% Lys-Arg solution must be administered intravenously for 4 hours (infusion rate: 250 ml/h); the infusion should start 30 minutes prior to the start of the Lutathera infusion, and continue during and up to at least 3 hours after the Lutathera infusion. See further details in Section 6.6. Hyperkaleamia must be corrected prior to 2.5% Lys-Arg infusion if >6.0 mmol/L (CTCAE Grade 3).

The 2.5% Lys-Arg solution will be supplied by Sponsor if it can't be compounded at the hospital Pharmacy. The composition of the 2.5% Lys-Arg solution is shown in Table 6-3 below.

Table 6-3. 2.5% Lys-Arg sterile solution composition*

Component	Quantity/1000 ml
L-lysine HCL	25g
L-arginine HCl	25g
Water for injection	qs 1000 ml

*The recommended pH range of the 2.5% Lys-Arg sterile solution is 5.0-7.0 with an osmolarity of 420-480 mOsm/kg.

In addition to the antiemetic premedication (see Section 6.6), in case of persistent nausea or vomiting during the administration of amino acids, Investigators are advised to use antiemetics which are commonly prescribed in their institutions for treatment of nausea induced by chemotherapeutic drugs. Among such antiemetics, the use of Aprepitant (Emend®) should be considered. Haloperidol (Haldol®) could also be considered as an adjunct treatment (either i.v. or oral) in case the advised antiemetic regimens are not successful and patients continue to vomit as well as Lorazepam (Ativan®).

In case where the treatments previously provided for nausea and vomiting are insufficient, a single dose of corticosteroids can be used, as long as it is not given before initiating or within one hour after the end of Lutathera infusion (please see Section 6.2.3).

6.1.1.1.3 Concomitant Treatment: 30 mg octreotide long-acting (Sandostatin® LAR Depot)

Sandostatin® LAR Depot (octreotide long-acting) is a pharmaceutical that is available in a single-use kit containing a 6-mL vial of 10 mg, 20 mg, or 30 mg strength for intramuscular injection, a syringe containing 2.5 mL of diluent, two sterile 1½” 19 gauge needles, and two alcohol wipes. For prolonged storage, Sandostatin® LAR Depot should be stored at refrigerated temperatures between 2°C and 8°C (36°F-46°F) and protected from light until the time of use. An instruction booklet for the preparation of drug suspension for injection is also included with each kit.

Octreotide long-acting 30 mg (Sandostatin® LAR Depot) is preferably administered the day after each administration of Lutathera and no earlier than 4 hours after completion of the Lutathera infusion. Due to the 6-weeks washout period before each Lutathera injection, no additional octreotide long-acting administrations are recommended between Lutathera treatments. Once Lutathera treatment completed, and also in case the Lutathera infusions have been suspended (e.g due to Dose Modifying Toxicity), patients will continue the 4-week interval administrations of 30 mg octreotide long-acting until the completion of the Treatment Phase (see Treatment Phase definition in Section 3).

6.1.1.2 Control arm

6.1.1.2.1 60 mg octreotide long-acting (Sandostatin® LAR Depot)

Sandostatin® LAR Depot (octreotide long-acting) is a pharmaceutical that is available in single-use kits containing a 6-mL vial of 10 mg, 20 mg, or 30 mg strength for intramuscular injection, a

syringe containing 2.5 mL of diluent, two sterile 1½” 19 gauge needles, and two alcohol wipes. For prolonged storage, Sandostatin® LAR Depot should be stored at refrigerated temperatures between 2°C and 8°C (36°F-46°F) and protected from light until the time of use. An instruction booklet for the preparation of drug suspension for injection is also included with each kit.

In the control arm, patients will receive administrations of octreotide long-acting 60 mg (Sandostatin® LAR Depot) at 4-week intervals (+/- 3 days) until the completion of the Treatment Phase (see Treatment Phase definition in Section 3), according to the schedule in Table 8-4 (i.e., two injections of Sandostatin® LAR 30 mg per treatment), unless the patient progresses or dies.

Patients who were SSA-naïve before enrolment in the study, should receive first planned dose at 30mg and the second (and subsequent) planned dose(s) at 60mg (full dose).

6.2 Other treatment(s)

6.2.1 Rescue medication

6.2.1.1 Short-acting Octreotide

Subcutaneous, short-acting octreotide injections may be indicated for control of symptoms (i.e. diarrhoea and flushing) in patients in both study arms, in accordance with the manufacturer’s prescribing information. Short-acting octreotide for symptom control is administered by the patient (at home) at investigator’s discretion.

6.2.2 Other anti-cancer treatments

The patient may not receive any other systemic therapy for the treatment of GEP-NET (chemotherapeutic, biologic, or any investigational agent) other than Lutathera and/or short-acting octreotide and/or octreotide long-acting during the study Treatment Phase. Localized therapy such as surgery or external beam irradiation may be performed on additional site(s), provided that it does not affect treatment response assessment; no surgeries are allowed within 12 weeks prior to Lutathera administration.

The last administration of long-acting SSA before the start of the study treatment is allowed during the screening period, before randomization, but should be carefully planned to allow a 6 weeks washout period prior to the administration of Lutathera.

New anti-cancer treatments administered after progression or during the Follow up Phase must be registered in the eCRF.

6.2.3 Prohibited medication

Somatostatin and its analogues competitively bind to somatostatin receptors. Therefore, administration of long-acting somatostatin analogues should be avoided within 6 weeks prior to the administration of Lutathera. If necessary, patients may be treated with short acting somatostatin analogues during the 6 weeks until 24 hours preceding Lutathera administration.

There is some evidence that corticosteroids can induce down-regulation of SST2 receptors. Therefore, as a matter of caution, repeated administration of high-doses of glucocorticosteroids should be avoided during Lutathera treatment. Patients with a history of chronic use of glucocorticosteroids should be carefully evaluated for sufficient somatostatin receptor expression. It is not known if there is any interaction between glucocorticosteroids used intermittently for the

prevention of nausea and vomiting during Lutathera administration. Therefore, glucocorticosteroids should be avoided as preventive anti-emetic treatment. In the case where the treatments previously provided for nausea and vomiting are insufficient, a single dose of corticosteroids can be used, as long as it is not given before initiating or within one hour after the end of Lutathera infusion.

The absence of inhibition or significant induction of the human CYP450 enzymes, the absence of specific interaction with P-glycoprotein (efflux transporter) as well as OAT1, OAT3, OCT1, OCT2, OATP1B1, OATP1B3 and BCRP transporters in pre-clinical studies suggest that Lutathera has a low probability of causing clinically relevant metabolism- or transporter-mediated interactions.

The following cautions regarding possible Octreotide Drug-Drug-Interactions (DDI) must be observed (according to the Octreotide label):

- Dose adjustment of medicinal products such as beta blockers, calcium channel blockers, or agents to control fluid and electrolyte balance may be necessary when administered concomitantly.
- Dose adjustments of insulin and antidiabetic medicinal products may be required when administered concomitantly.
- Octreotide has been found to reduce the intestinal absorption of ciclosporin and to delay that of cimetidine.
- Concomitant administration of octreotide and bromocriptine increases the bioavailability of bromocriptine.
- Drugs mainly metabolized by CYP3A4 and which have a low therapeutic index should be used with caution (e.g. quinidine, terfenadine).
- Octreotide may alter absorption of dietary fats in some patients.
- Depressed vitamin B12 levels and abnormal Schilling's tests have been observed in some patients receiving octreotide therapy. Monitoring of vitamin B12 levels is recommended during therapy with Octreotide LAR in patients.

6.2.4 SARS-CoV-2 vaccination

Patients are allowed to receive approved SARS-CoV-2 vaccines (inactivated, viral-vector-, or mRNA based Sars-CoV-2 vaccines approved by the Health Authorities in the country). The decision for vaccination should be done on a case-by-case basis and at the discretion of the treating physician, with patient consent, and in alignment with institutional guidelines, if available.

Investigators are expected to evaluate potential risks/benefits in the context of individual patient characteristics, and make an individualized decision for vaccination. Investigators should refer to the prescribing information for the SARS-CoV-2 vaccine planned to be used and/or follow local Health Authorities or institutional guidelines, if available.

It is encouraged to complete the vaccination for COVID-19 (i.e. receive the two vaccine doses if necessary) prior to initiation of study treatment. If the vaccination should occur while a patient is on-treatment, vaccine may be administered per routine clinical practice.

Patients should be instructed to contact their GP and/or the study site in case of side effects after vaccination.

Vaccine must be reported as a prior/concomitant medication according to the protocol guidelines. Any AEs after vaccination must be monitored and reported in eCRF.

6.3 Subject numbering, treatment assignment, randomization

6.3.1 Subject numbering

For all patients who have signed the ICF, a screening number will be assigned in chronological order starting with the lowest number available on site. Patients will be identified by a unique patient identification number (Patient ID No.) composed of the center number and the screening number.

6.3.2 Treatment assignment, randomization

After the screening period, eligible patients will be randomly assigned (ratio 2:1) to one of the two study groups for treatment with Lutathera plus octreotide long-acting (30 mg) or high dose octreotide long-acting (60 mg). The randomization system will assign a unique randomization number to the patient, which will be used to link the patient to a treatment arm. Randomization will be stratified by tumor Grade (G2 vs G3) and tumor origin (pNET vs other origin) according to a stratified permuted block scheme.

The details of the procedure to obtain the patient randomization number will be described in the Investigator's Manual.

6.4 Treatment blinding

Due to the radioactive nature of Lutathera and its method of infusion, it is not possible to implement a blinded design for this study.

6.5 Dose compliance, modification and discontinuation

6.5.1 Dose compliance

For each single-dose of Lutathera a deviation of ±10% from the scheduled dose is allowed.

6.5.2 Dose modification

Modifying Toxicities (DMT) will apply in both arms as discussed in Sections 6.5.2.1 and 6.5.2.2. Patients whose treatment is interrupted or permanently discontinued due to toxicities that fulfil the criteria for a DMT, must be followed up at regular intervals (at least once a week), until resolution or stabilization of the event(s), whichever comes first.

Appropriate clinical experts such as cardiologists, endocrinologists, hepatologists, nephrologists etc. should be consulted as deemed necessary.

6.5.2.1 Dose modifying toxicities (DMTs) for Lutathera

For patients who do not tolerate the protocol-specified dosing schedule, dose adjustments are permitted to allow the patient to continue the study treatment.

The criteria for dose modifications of Lutathera for toxicities are outlined in Table 6-4.

If a patient requires a dose delay of > 16 consecutive weeks of Lutathera from the day of the previous dose, then the patient should be discontinued from the study treatment. In exceptional situations, if the patient is clearly benefiting from the study treatment (i.e. stable disease, partial response, complete response), and in the opinion of the investigator no safety concerns are present, after discussion with AAA Medical Monitor, the patient may remain on the study treatment.

For each patient, a maximum of one dose reduction will be allowed after which the patient will be discontinued from the study.

These dose changes must be recorded on the appropriate electronic case report form (eCRF).

Table 6-4. Dose Modifications of Lutathera for Adverse Events

Adverse Reaction	Worst Toxicity CTCAE Grade (unless otherwise specified)	Dose Modification
Thrombocytopenia	First occurrence of:<br><br>Grade 2 (PLT < 75 - 50 x 10^9/L)<br>Grade 3 (PLT < 50 - 25 x 10^9/L)<br>Grade 4 (PLT < 25 x 10^9/L)	Withhold dose until complete or partial resolution (Grade 0 to 1).<br><br>If AE resolves before next planned Lutathera dose (i.e. within 9 weeks after the previous dose), administer next full dose of Lutathera (7.4 GBq / 200 mCi).<br><br>If AE resolves within 9 – 16 weeks after the previous dose, resume Lutathera at 3.7 GBq (100 mCi). If reduced dose does not result in Grade 2, 3 or 4 thrombocytopenia, administer Lutathera at 7.4 GBq (200 mCi) for next dose.<br><br>If AE does not resolve within 16 weeks after the previous dose, permanently discontinue Lutathera.
	Recurrent Grade 2, 3 or 4	Permanently discontinue Lutathera.
Anemia	First occurrence of:<br><br>Grade 3 (Hgb < 8.0 g/dL); transfusion indicated<br>Grade 4 (life threatening consequences)	Withhold dose until complete or partial resolution (Grade 0, 1, or 2).<br><br>If AE resolves before next planned Lutathera dose (i.e. within 9 weeks after the previous dose), administer next full dose of Lutathera (7.4 GBq / 200 mCi).

		If AE resolves within 9 – 16 weeks after the previous dose, resume Lutathera at 3.7 GBq (100 mCi). If reduced dose does not result in Grade 3 or 4 anemia, administer Lutathera at 7.4 GBq (200 mCi) for next dose.<br><br>If AE does not resolve within 16 weeks after the previous dose, permanently discontinue Lutathera.
	Recurrent Grade 3 or 4	Permanently discontinue Lutathera.
Neutropenia	First occurrence of:<br><br>Grade 3 (ANC < 1.0 - 0.5 x 10^9/L)<br>Grade 4 (ANC < 0.5 x 10^9/L)	Withhold dose until complete or partial resolution (Grade 0, 1, or 2).<br><br>If AE resolves before next planned Lutathera dose (i.e. within 9 weeks after the previous dose), administer next full dose of Lutathera (7.4 GBq / 200 mCi).<br><br>If AE resolves within 9 – 16 weeks after the previous dose, resume Lutathera at 3.7 GBq (100 mCi). If reduced dose does not result in Grade 3 or 4 neutropenia, administer Lutathera at 7.4 GBq (200 mCi) for next dose.<br><br>If AE does not resolve within 16 weeks after the previous dose, permanently discontinue Lutathera.
	Recurrent Grade 3 or 4	Permanently discontinue Lutathera.
Renal Toxicity	First occurrence of:<br>• Creatinine clearance less than 40 mL/min; calculated using Cockcroft Gault formula with actual body weight, or<br><br>• 40% increase from baseline serum creatinine, or<br><br>• 40% decrease from baseline creatinine clearance; calculated using Cockcroft Gault formula with actual body weight.	Withhold dose until complete resolution.<br><br>If AE resolves before next planned Lutathera dose (i.e. within 9 weeks after the previous dose), administer next full dose of Lutathera (7.4 GBq / 200 mCi).<br><br>If AE resolves within 9 – 16 weeks after the previous dose, resume Lutathera at 3.7 GBq (100 mCi). If reduced dose does not result in renal toxicity, administer Lutathera at 7.4 GBq (200 mCi) as next dose.

		If AE does not resolve within 16 weeks after the previous dose, permanently discontinue Lutathera.
	Recurrent renal toxicity Grade 3 or 4	Permanently discontinue Lutathera.
Hepatotoxicity	First occurrence of:<br>Bilirubinemia greater than 3 times the upper limit of normal (Grade 3 or 4), or	Withhold dose until complete or partial resolution (Grade 0, 1 or 2).<br><br>If AE resolves before next planned Lutathera dose (i.e. within 9 weeks after the previous dose), administer next full dose of Lutathera (7.4 GBq / 200 mCi).<br><br>If AE resolves within 9 – 16 weeks after the previous dose, resume Lutathera at 3.7 GBq (100 mCi). If reduced Lutathera dose does not result in G3 or 4 bilirubinemia, administer Lutathera at 7.4 GBq (200 mCi) as next dose.<br><br>If AE does not resolve within 16 weeks after the previous dose, permanently discontinue Lutathera.
	Defined as:<br>Hypoalbuminemia less than 30 g/L with International Normalized Ratio (INR) >1.5.	Withhold dose until complete or partial resolution (Grade 0 to 1).<br><br>If AE resolves before next planned Lutathera dose (i.e. within 9 weeks after the previous dose), administer next full dose of Lutathera (7.4 GBq / 200 mCi).<br><br>If AE resolves within 9 – 16 weeks after the previous dose, resume Lutathera at 3.7 GBq (100 mCi). If reduced Lutathera dose does not result in G2, 3 or 4 hypoalbuminemia, administer Lutathera at 7.4 GBq (200 mCi) as next dose.<br><br>If AE does not resolve within 16 weeks after the previous dose, permanently discontinue Lutathera.
	Recurrent hepatotoxicity	Permanently discontinue Lutathera.
Any other CTCAE* Grade 3 or Grade 4 AE[1]	First occurrence of Grade 3 or 4	Withhold dose until complete or partial resolution (Grade 0 to 2).

		If AE resolves before next planned Lutathera dose (i.e. within 9 weeks after the previous dose), administer next full dose of Lutathera (7.4 GBq / 200 mCi).<br><br>If AE resolves within 9 – 16 weeks after the previous dose, resume Lutathera at 3.7 GBq (100 mCi). If reduced dose does not result in Grade 3 or 4 toxicity, administer Lutathera at 7.4 GBq (200 mCi) as next dose.<br><br>If AE does not resolve within 16 weeks after the previous dose, permanently discontinue Lutathera.
	Recurrent Grade 3 or 4	Permanently discontinue Lutathera.
Severe heart failure	NYHA class III or IV	Permanently discontinue Lutathera.
[1] No dose modification required for hematological toxicities Grade 3 or Grade 4 solely due to lymphopenia<br>*CTCAE: Common Terminology Criteria for Adverse Events, National Cancer Institute		

In addition,

- permanently discontinue treatment with Lutathera if hypersensitivity due to the active substance or any of excipients of Lutathera is observed;
- Temporarily discontinue treatment with Lutathera, until resolution or stabilisation before treatment can be resumed.
  - Occurrence of an intercurrent disease (e.g. urinary tract infection);
  - Major surgery: wait 12 weeks after the date of surgery, then restart the treatment with Lutathera.

After resolution of a DMT, a patient may receive subsequent planned treatment(s) at the full dose (if resolved within 9 weeks after the previous dose), or at 50% of the standard treatment dose (if resolved within 9 – 16 weeks after the previous dose), if this is felt to be safe for the patient, or the risk-benefit assessment is favourable. If the same DMT reoccurs after treatment with the reduced Lutathera dose, the patient will remain in the study and continue the scheduled clinical / tumor assessments until tumor progression, but no further Lutathera treatment will be given. Octreotide long-acting 30 mg will be continued at monthly intervals. If the DMT event does not reoccur, the next treatment is at full dose, if it is considered to be safe for the patient, or the risk-benefit assessment is favourable.

If a patient experiences a DMT during Lutathera therapy, subsequent treatments with Lutathera are permissible, provided the DMT resolves within 16 weeks following the non-tolerated administration. In any case, the patient will continue the administration of 30 mg octreotide long-acting.

Lutathera overdose, has a very low probability of occurring since it will be supplied as a single dose "ready to use product" in order to avoid any manipulation outside the production facilities. In addition, the infusion system methods do not allow the concurrent use of two separate Lutathera solution vials (see pharmacy manual). No doubling of the administered radioactivity is ever allowed either in absolute amount or by shortening the time intervals between treatments. Treatments (amount of radioactivity and time of administration) will be monitored during the study and any unallowed treatment modification will be considered a major protocol violation.

6.5.2.2 Dose modifying toxicities (DMTs) for Octreotide LAR

Control arm (Octreotide LAR 60 mg)

In the treatment arm with octreotide long-acting, a dose adjustment will be applied in case of Grade 3 or 4 toxicity, especially in presence of severe abdominal symptoms, and hypoglycaemia/ hyperglycemia possibly related to octreotide long-acting. The dose adjustment will also be applied if such adverse events are observed which are unlikely related to the study drug, but to other possible or concomitant causes, and the full administration of octreotide long-acting would represent a safety risk for the patient.

If a patient experiences a DMT during octreotide long-acting treatment, the subsequent treatment dose will be reduced from 60 mg to the previous well-tolerated dose (or even temporarily suspended) and then at the next treatment, the dose will be increased to the initial 60 mg dose of octreotide long-acting, if this is felt to be safe for the patient, or the risk-benefit assessment is favorable.

In any case (also in case of octreotide long-acting treatment dose suspension), the patient remains in the study and continues the scheduled clinical / tumor assessments until tumor progression, unless the patient's withdrawal becomes inevitable (Section 9 – Study discontinuation).

Lutathera arm (Octreotide LAR 30 mg)

All DMT recommendations in the control arm (Octreotide LAR 60 mg) are also applicable to the Lutathera arm, where Octreotide LAR is administered at the standard approved dosage (30 mg). Overall, the exact dose reduction levels, duration of suspension and criteria for re-increase and continuation of dose in the control arm will be defined by the treating physician based on clinical judgment and product leaflet.

6.5.3 Dose discontinuation

The discontinuation of either study treatments in both arms (Lutathera or octreotide long-acting) is not a reason for patient's withdrawal either from the clinical/tumor assessments until tumor progression, or for early study termination (reasons for the patient's withdrawal are discussed in Section 9 – Study discontinuation). However, in case of a patient's withdrawal from the clinical/tumor assessments or early study termination (based on either the patient's or the Investigator's decision), patients will undergo all exams scheduled for the End of Treatment (EOT) visit (Table 8-3, Table 8-4).

If the treatment discontinuation occurs because of laboratory abnormality, or any evidence of toxicity, the Investigator will collect additional specimens for repeat or additional analyses, at intervals appropriate to the abnormality. The patient will be closely followed until sufficient information is obtained to determine the cause or the value regresses. Appropriate remedial measures should be taken and the response recorded in the eCRF.

6.6 Preparation and dispensation of Lutathera

There is no need for the patients to fast before treatment.

Treatment with Lutathera will consist of a cumulative amount of radioactivity of 29.6 GBq (800 mCi) with the dosing divided among 4 administrations of Lutathera at 8±1-week intervals or up to 16 weeks to accommodate resolving acute toxicity (see Section 6.5.2.1). Each dose is infused over 30 minutes.

In addition to treatment with Lutathera, patients will receive 30 mg octreotide long-acting until the completion of the Treatment Phase (see Treatment Phase definition in Section 3).

Before each Lutathera treatment:

1. Unless clinically impossible, octreotide long-acting must not be administered within 6 weeks of the next treatment of Lutathera. In other words, between 2 Lutathera injections performed at regular interval (8 weeks) only one 30mg octreotide long-acting injection can be administered (possibly one day after Lutathera administration or at least after 4 hours after the end of Lutathera infusion). While not treated with octreotide long-acting, the patient must be treated with an equivalent dosing of short acting octreotide s.c. "Clinically impossible" means that the actual clinical condition of the patient would contraindicate the suspension of treatment because of an otherwise untreatable carcinoid syndrome. After enrollment, octreotide long-acting injections should be planned in order to allow for suitable washout time before first Lutathera treatment. If the time interval between treatments with Lutathera (see Section 6.5.2.1) is prolonged for any reason, octreotide long-acting administration continues every 4 weeks, however it should not be administered within 6 weeks of the next Lutathera treatment.
2. Short-acting octreotide is not allowed during the 24 h before the Lutathera treatment date, unless the actual patient clinical conditions would contraindicate the treatment suspension due to otherwise untreatable carcinoid syndrome symptoms. Short-acting octreotide can only

be continued if the tumor uptake on SRI imaging during continued somatostatin analogue medication is greater than liver uptake (see Exclusion criteria).

3. Treatment with 30 mg octreotide long-acting can be resumed after the administration of Lutathera. Specifically, the recommended period before resuming octreotide long-acting (or short acting octreotide s.c.) is one day, however the minimum interval to receive octreotide long-acting (or short acting octreotide s.c.) after Lutathera is 4 hours, unless contraindicated as noted above.

The total amount of administered radioactivity is determined by measuring the radioactivity in the Lutathera vial before and after administration (the procedure is provided in Appendix 8).

The scheme for supportive treatment with 30 mg octreotide long-acting is presented in Figure 6-1.

Figure 6-1. Lutathera Arm: schedule for administration of Lutathera, 30 mg octreotide long-acting, and short acting octreotide sc[1].

Treatment	Wk0	Wk1	Wk2	Wk3	Wk4	Wk5	Wk6	Wk7	Wk8
Lutathera[2,3]	X· · · · · ·	·	·	·	·	·	·	·	X· · · · · ·
Octreotide long-acting 30 mg[4]	·X· · · · ·	·	·	·	·	·	·	·	·X· · · · ·
Octreotide s.c. short acting	· · · · · · ·	·	·	·	X→	X→	X→	XXXXXX·	· · · · · · ·

↑ Octreotide long-acting is resumed the day after Lutathera treatment

Octreotide S.C. stopped 24hr before Lutathera treatment ↑

1 Symbols: (X) treatment with Lutathera, (X) treatment with octreotide long-acting, (X, X→) daily injections of octreotide s.c., (·) no treatment
2 Lutathera treatment interval can be increased to 16 weeks to resolve acute toxicity
3 If Lutathera treatment is delayed due to DMT, octreotide long-acting may be continued at 4-week intervals, but should not be administered within 6 weeks before next Lutathera treatment; patient should use octreotide s.c. during the washout period
4 After patients receive all 4 Lutathera administrations at 8±1 week intervals, or Lutathera treatment stopped because of toxicity, patients continue to receive 30 mg octreotide long-acting (1 im injection every 4 weeks) until end of Treatment Phase, unless progression occurs.

On the day of Lutathera treatment, and before the infusion with 2.5% Lys-Arg solution is started, an intravenous bolus of anti-emetic is given (suggested options: Granisetron (3 mg), or Ondansetron (8 mg), or Tropisetron (5 mg)). Prednisone must be avoided as preventive anti-emetic treatment because of potential somatostatin receptor down-regulation.

Hyperkaleamia must be corrected prior to 2.5% Lys-Arg infusion if >6.0 mmol/L (CTCAE Grade 3).

In case nausea or vomiting occurs despite this medication, patients can be treated with other anti-emetic drugs at the discretion of the physician.

The sterile amino acid solution and Lutathera are administered in parallel by peripheral vein infusion at a constant infusion rate through pumps or any other infusion system. The infusion with amino acids starts 30 minutes before the start of Lutathera infusion, and continues for a total of 4 h (extension up to 6 h is allowed in case of adverse reactions that require interruption or slowing the infusion rate). During amino acid infusion patient is allowed to void.

Infusion rates are listed in Table 6-5 and see Pharmacy Manual for the infusion system scheme.

Table 6-5. Administration procedure of antiemetic, sterile amino acid solution and Lutathera

Administered agents	Start time (min)	Infusion rate (mL/h)	Duration
Antiemetic: Granisetron 3 mg (or alternative)	0	as per prescribing information	as per prescribing information
2.5% Lys-Arg sterile amino acid solution (1L)	30	250	4 hours
Lutathera with sodium chloride 9 mg/mL (0.9%) solution for injection	60	Up to 400	30 ± 10 minutes

Following administration of Lutathera, patients should remain at the clinical site for an additional 4 to 5 hours in an area with suitable radiation shielding to protect others from unnecessary exposure (Appendix 4). At the time of release, patients are given written instructions (Appendix 5) which outline the precautions the patient must take to minimize radiation exposure to people around them.

Crises due to excessive release of hormones or bioactive substances may occur following treatment, therefore, observation of patients by overnight hospitalization should be considered. Recommended treatments of patients with hormonal crises are: i.v. high dose somatostatin analogues, i.v. fluids, corticosteroids, and correction of electrolyte disturbances in patients with diarrhea and vomiting.

6.6.1 Handling of study treatment and additional treatment

Lutathera, amino acids and octreotide long-acting must be administered at the investigational site. Short-acting octreotide is self-administered by the patient.

The study medication must be stored, handled and administered only by qualified/authorized personnel and must be prepared in accordance with pharmaceutical quality requirements, and radiation safety regulations for Lutathera.

Drug inventory and accountability records for the study medication and rescue medication, as well as drug returns by the patient, will be kept by the Investigator/Pharmacist, and must be documented throughout the study. Returned supplies should not be distributed again, not even to the same patient. The Investigator will not supply investigative study medication to any person, except the patients in this study.

The octreotide long-acting and amino acids not used must be stored at site and made available till the monitoring visits, to allow the CRA to monitor the drug accountability.

The used/unused medications, except for Lutathera, which will be locally discarded according to all disposal requirements for radioactive materials, will be returned to the proper local depot for destruction at the study completion or upon expiration, according to IPM/Sponsor decision and approval.

On an ongoing basis the Investigator/Pharmacist agrees to conduct a study medication supply inventory and to record the results of this inventory on the study Medication Accountability Record. It must be possible to reconcile delivery records with those of used and unused medication. Any discrepancies must be accounted for and explained. Appropriate forms of deliveries and returns must be signed and dated by the responsible person at the clinical site and maintained as records. The return or disposal of all study medication will be documented appropriately.

7 Informed consent procedures

Eligible subjects may only be included in the study after providing (witnessed, where required by law or regulation), IRB/IEC-approved informed consent.

If applicable, in cases where the subject's representative(s) gives consent (if allowed according to local requirements), the subject must be informed about the study to the extent possible given his/her understanding. If the subject is capable of doing so, he/she must indicate agreement by personally signing and dating the written informed consent document.

Informed consent must be obtained before conducting any study-specific procedures (e.g. all of the procedures described in the protocol). The process of obtaining informed consent must be documented in the subject source documents.

Advanced Accelerator Applications will provide to investigators in a separate document a proposed informed consent form that complies with the ICH GCP guidelines and regulatory requirements and is considered appropriate for this study. Any changes to the proposed consent form suggested by the investigator must be agreed by Advanced Accelerator Applications before submission to the IRB/IEC.

Information about common side effects already known about the investigational drug can be found in the IB. This information will be included in the subject informed consent and should be discussed with the subject during the study as needed. Any new information regarding the safety profile of the investigational drug that is identified between IB updates will be communicated as appropriate, for example, via an investigator notification or an aggregate safety finding. New information might require an update to the informed consent and then must be discussed with the subject.

Women of child bearing potential must be informed that taking Lutathera can cause fetal harm when administered to a pregnant woman and agree that in order to participate in the study they must adhere to the contraception requirements. Male subjects must be informed that if a female partner becomes pregnant while he is enrolled in the study, contact with the female partner will be attempted to request her consent to collect pregnancy outcome information. Subjects must be informed of the contraception requirements outlined in the Inclusion/Exclusion Criteria (Section 5).

A copy of the approved version of all consent forms must be provided to Advanced Accelerator Applications after IRB/IEC approval.

It is recommended that the Investigator inform the patient’s general practitioner of his/her participation in the study, provided that the patient has a general practitioner and the patient agrees to disclose this information.

The informed consent form (ICF) signed at the time of the inclusion foresees the patient’s participation in the Study until the end of the Follow-up Phase (adolescents will sign a dedicated informed consent). Patients should sign an addendum to the consent to participate in the Follow-up Phase assessment phase only in case they have withdrawn the initially signed consent.

After signing an ICF for participation in the study, each patient is given a patient card, which indicates the contact details of the Investigator (e.g. stamp with telephone number), the patient's Subject ID, as well as the medication number. The patient shall carry this card with him/her during participation in the study so that the Investigator may be contacted in case of emergency.

Patients randomized in the Lutathera arm will also receive written instructions which outline the precautions the patient must take to minimize radiation exposure to people around them.

Patients who will decide to participate after progression to the optional Treatment Extension Phase (cross-over) or Re-treatment Phase will need to sign a new informed consent before starting the treatment.

8 Visit schedule and assessments

Randomization must be performed once all screening/baseline assessments are complete.

The baseline CT/MRI scan should be taken possibly on the same day of randomization or immediately before (within 1 week) to ensure that it reflects the disease status closely before the therapy start. The results of baseline CT/MRI must be assessed by Investigator before randomization.

The screening phase must be shortened as much as possible in order to treat the patients shortly after the informed consent signature.

Randomization should occur as soon as possible after all screening assessments have been completed and eligibility confirmed. As Lutathera production and shipment will take approximately 12 days, if a patient is randomized in the Lutathera arm, Lutathera first dose must be ordered immediately after randomization.

During the study, patients will be evaluated for safety and tolerability in accordance with the Visit Schedules for the Lutathera arm and the octreotide long-acting arm as indicated in Table 8-3, and Table 8-4 (variations of ±1 week in the visits schedule are allowed).

Subjects should be seen for all visits/assessments as outlined in the assessment schedule or as close to the designated day/time as possible. Missed or rescheduled visits should not lead to automatic discontinuation. Subjects who prematurely discontinue the study treatment for any reason should be scheduled for a visit as soon as possible, at which time all of the assessments listed for the EOT/Early Termination visit will be performed (Table 8-3). At the EOT/Early Termination visit, all dispensed investigational product should be reconciled, and the adverse event and concomitant medications recorded on the eCRF.

As the date of randomization and the date of first treatment might not be the same, it should be observed that a misalignment may occur between the tumor imaging exams (CT/MRI scans) which are scheduled from the date of randomization, and the other clinical and laboratory assessments which are scheduled from the first treatment date.

The assessments listed in Table 8-1 will be performed centrally. Procedures for centralized evaluations will be detailed in the Laboratory Manuals provided to each participating site.

Assessment schedule lists all of the assessments and when they are performed. All data obtained from these assessments must be supported in the subject's source documentation.

Table 8-1. Centrally Performed Assessments.

Assessment	Material to be Delivered	Study Phase	Section
SRI tumor uptake in all documented target lesions will be assessed locally at screening to verify that tumor uptake in all target lesions is greater than the liver uptake.	SRI modality: [68Ga]-DOTA-TOC (e.g. Somakit-TOC®) PET/CT (or MRI when applicable based on target	Screening & before cross-	8.1.4

If the patient will participate to the optional Treatment Extension Phase after progression, SRI tumor uptake in all documented target lesions will be re-assessed locally before cross-over.<br><br>*Images will be also submitted to the central imaging center for a second review (there is no central 'real time' assessment of the SRI images, however the images should be submitted to the central imaging center possibly within 1 month. SRI images submission is not required for screening failure patients.*	lesions) imaging or [68Ga]-DOTA-TATE PET/CT (or MRI when applicable based on target lesions) imaging (e.g. NETSPOT®) Somatostatin Receptor scintigraphy (SRS) with [111In]-pentetreotide (Octreoscan® SPECT/CT), SRS with [99mTc]-Tektrotyd, [64Cu]-DOTA-TATE PET/CT (or MRI when applicable based on target lesions) imaging.<br>*SRI obtained within 3 months of randomization will be accepted for inclusion; if older, the exam will need to be repeated at screening.*<br>*If the patient participates to the optional Treatment Extension or re-treatment Phase (after progression), SRI imaging must be repeated before cross-over/re-treatment. The SRI images should be submitted to the central imaging center (uploading into the dedicated e-platform) possibly within 1 month.*<br>*See Imaging Manual for further details.*	over/re-treatment	
Objective tumor response according to RECIST Criteria.<br>*Tumor response according to RECIST is re-evaluated centrally (real-time reading) during the study.*	CT/MRI scans.<br>It is recommended that for each patient identical acquisition and reconstruction protocols be used at all time-points.<br>*The images must be submitted (uploading into the dedicated e-platform) to the central imaging center as soon as possible for real-time assessment.*<br>*See Imaging Manual for further details.*	Treatment Phase	8.3
Measurements of the RR, PR, QRS, and QT interval durations.	12-lead ECGs will be performed using the device provided by Sponsor.	Baseline & Treatment Phase	8.5.5

12-lead ECGs in triplicate will be re-assessed centrally at baseline and during the study.	*Data will be transferred after acquisition to the central laboratory for the central cardiologist assessment.*<br>*See ECG Manual for further details.*		
PK of high dose octreotide long-acting (60 mg), to evaluate steady-state trough plasma concentration assessment (all Countries except China).<br><br>*Blood samples (2.5 mL each sample) will be collected at the pre-specified pre-dose time-points to yield approximately 1 mL plasma for analysis of octreotide concentration.*	Blood will be collected using the sampling tubes provided by Sponsor. The storage temperature for PK samples should be at or below minus 20°C (-20°C).<br><br>*Samples will be shipped regularly to the central laboratory for central PK assessment.*<br>*See PK Manual for further details.*	Treatment Phase/control arm only	8.6

If the COVID-19 pandemic limits or prevents on-site study visits, alternative methods of providing continuing care may be implemented. Phone calls and virtual contacts (e.g. teleconsult) can be implemented for the duration of the pandemic until it is safe for the participant to visit the site again. To follow is a set of general recommendations on the management of patient's visits during COVID-19:

-Study patients should be instructed to contact the study investigator (by telephone or email), if there is a change in their health status.

-The study investigator may consider contacting patients more frequently than is required by the study protocol, with frequency selected according to circumstances and including the individual patient's vulnerability to COVID-19 based on their medical condition. When contacting patients, whether by telephone or in writing, the study investigator should record that contact or save written correspondence to the patient medical record or chart so as to assure a proper source documentation for the trial.

-Safety lab testing - The investigator should consider whether it is possible to arrange for the laboratory panels (e.g. chemistry and hematology) to be performed at a local laboratory for those patients who cannot come to the clinical site as scheduled. The investigator should collect and file all local lab reports in the patient medical record or chart.

-Scheduled or unscheduled patient study visits can be performed once feasible and considered safe for patients to attend the clinical appointments.

If contact is made with a patient via telephone/video call, the Visit Date should be entered into the eCRF for the corresponding visit / next closest visit. If multiple contacts are made prior to the next scheduled visit, an unscheduled visit per contact can be entered to capture the Visit Date.

CRFs that can be completed as a result of the telephone/video-visit should be completed, e.g. Adverse Events, Concomitant Medications, and Dose Administration Record. If any protocol required assessments were not completed, CRFs should be 'Inactivated' or marked 'Not Done'.

The Protocol Deviation process will be used to capture visits not performed at site due to COVID-19 (see Section 14).

8.1 Demographics and Baseline Characteristics

Each patient's date of birth, gender, ethnicity, weight, height and relevant baseline characteristics will be recorded in the e-CRF.

8.1.1 Diagnosis and Extent of Cancer

The patient's disease history, including documented primary diagnosis of gastro-entero-pancreatic tumor, date of diagnosis (diagnosis date of metastasized or locally advanced disease should not be greater than 6 months prior to screening), as well as disease status at study entry, will be collected. This includes the date of first diagnosis and presence of metastases with specification of the metastatic site(s). TNM criteria will be used for the determination of the stage of disease at the time of first diagnosis.

8.1.2 Ki67

All patients are required to have documented local assessment of the Ki67 proliferation index based on surgery/biopsy specimens of the primary tumor or liver metastases or soft tissue metastases and assessed by microscopy and immunohistochemical staining.

Ki67 must be ≥10% and ≤ 55% for a patient to be eligible.

8.1.3 Prior Antineoplastic Medications / Radiotherapy / Surgery

Information pertaining to any chemotherapy, hormonal therapy, immunotherapy, radiation, or surgery the patient has previously received will be documented. Previous treatment with somatostatin analogs will be also documented if applicable.

8.1.4 SRI Tumor Uptake in Documented Lesions

All patients are required to have [68Ga]-DOTA-TOC (e.g. Somakit-TOC®) PET/CT (or MRI when applicable based on target lesions) imaging or [68Ga]-DOTA-TATE PET/CT (or MRI when applicable based on target lesions) imaging (e.g. NETSPOT®) or Somatostatin Receptor scintigraphy (SRS) with [111In]-pentetreotide (Octreoscan® SPECT/CT) or SRS with [99mTc]-Tektrotyd or [64Cu]-DOTA-TATE PET/CT (or MRI when applicable based on target lesions) imaging performed within 3 months prior to the projected randomization date in the study (available exams older than 3 months need to be repeated to evaluate the patient's eligibility).

SRI imaging must be repeated before cross-over (after progression in the control arm), if the patient participates to the optional Treatment Extension Phase, and before the optional re-treatment (after progression in the Lutathera arm).

SRI tumor uptake in documented target lesions will be assessed locally to evaluate patient's eligibility before randomization, cross-over and re-treatment. Tumor uptake in all target lesions must be greater than the liver uptake.

Images will be also submitted to the central imaging center for a second review: there is no central 'real time' assessment of the SRI images, however the images should be submitted to the central imaging center possibly within 1 month. SRI images submission is not required for screening failure patients.

Lytic bone lesions, with an identifiable soft tissue component, evaluated by CT or MRI, can be considered as measurable lesions if the soft tissue component otherwise meets the definition of measurability according to RECIST 1.1 (in any case, blastic bone lesions are not measurable). Therefore, if the bone lesion has the characteristic as above, and SRI is positive, the patient may be enrolled.

If the primary tumor has been resected, tumor evaluations including SRI will be performed on metastases.

Operating details and quality certificates are specified in the Imaging Manual and Imaging Charter.

8.2 Prior/Concomitant Medications

All medications taken at the start of screening until the end of the Treatment Phase/optional Treatment Extension Phase/optional Re-treatment Phase, or early termination, are to be recorded. This includes prescription and over-the-counter medications taken during this time frame.

During the Follow up Phase, concomitant medications must be collected only if administered for related SAEs/AESI and/or for secondary hematological malignancies. In addition, further anti-tumor treatments administered after progression must be reported until the end of the Follow up Phase.

If any additional medication or procedure has been given for treatment of suspected or confirmed COVID-19 during the study, the site is requested to please enter them as a concomitant medication or procedure (e.g. intubation) and state/ select 'reason' to match the relevant Adverse Event reported when applicable.

Tests performed to diagnose symptoms of COVID-19 should be reported as Adverse Events only per Section 10.1.1 and not duplicated in Concomitant Medications/ Surgical and Medical Procedures or equivalent CRFs.

8.3 Efficacy Assessment

8.3.1 Progression Free Survival

The primary efficacy end-point is PFS as measured by objective tumor response, which is determined by RECIST criteria, Version 1.1 (Eisenhauer, et al., 2009). Tumor response will be assessed locally and centrally.

Triphasic CT imaging is the preferred modality over MRI for determining objective tumor response. Combined PET/CT may be used only if the CT is of similar diagnostic quality as a CT performed without PET, including the utilization of IV contrast media.

The baseline CT/MRI scan should be taken possibly on the same day of randomization or immediately before (within 1 week) to ensure that it reflects the disease status closely before the therapy start. The results of baseline CT/MRI must be assessed by the Investigator before randomization.

The tumor diameters of indicator lesions used for response assessment should be measured in the closest position to that used for the baseline CT or MRI assessment.

Restaging is scheduled at regular intervals in the two treatment arms (week 16±1, week 24±1 and then every 12 ±1 weeks) starting from the date of randomization (see Table 8-3, and Table 8-4). Every effort should be done to avoid differences between these timings for patients in the two arms. In case of delays, the reason of the delay has to be documented and the CT/MRI assessment has to be done as soon as possible.

If a CT/MRI scan is done on the same day of a Lutathera administration (e.g. in case of therapy postponement due to DMT or scheduling issues), the scan should be taken before the drug administration. When a CT/MRI assessment is >6 weeks earlier or later than the original schedule, the next reassessments have to be discussed and adjusted with the Medical Monitor of the study in a way to progressively come back to the original schedule.

It is recommended that for each patient identical acquisition and reconstruction protocols be used at all time-points. Central, blinded, real-time IRC (Independent Review Committee) assessment will be conducted for determining progressive disease. Changes from randomization date will be assessed at week 16±1, week 24±1 and then every 12 ±1 weeks until the PFS primary analysis End-Point has been reached, then until Week 72 after randomization, unless the patient progresses or dies.

Sponsor will notify all the Centers and their Ethic Committees as soon as the 99 PFS events have occurred.

Additional PFS/PFS2 data will be collected up to 3 years from the end of the Treatment Phase of the last patient (during the follow-up phase RECIST 1.1 assessment will be performed only locally every 6 months (± 1 month)).

Additional imaging assessments may be performed at any time during the study at the investigator's discretion to support the efficacy evaluations for a subject, as necessary. Clinical suspicion of disease progression at any time requires a physical examination and imaging assessments to be performed promptly rather than waiting for the next scheduled imaging assessment. These additional scans should be provided to the IRC for central assessment. If an off-schedule imaging assessment is performed because progression is suspected, subsequent imaging assessments should be performed in accordance with the original imaging schedule.

After progression and during the follow-up every effort must be made by the Investigator to collect additional information on further anti-tumor therapies and scan assessments outcome (RECIST 1.1 local evaluation) to evaluate the Time to Second Progression (PFS2) in the two study arms.

For progressive patients participating in cross-over or re-treatment, CT/MRI scans performed after the cross-over or re-treatment eligibility will follow the visits schedule counted from the 1st Lutathera dose.

For patients that are re-treated with Lutathera, a separate PFS analysis will be conducted during the re-treatment phase using the same definition as in Section 12.5.2 except that the baseline will be based on the tumor assessment which demonstrated progression thus allowing re-treatment with Lutathera. This PFS analysis during re-treatment will be descriptive only.

Operating details and quality certificates are specified in the Imaging Manual and Imaging Charter. See Section 8.8 for further details.

8.3.2 Objective Response Rate (ORR), Duration of Response (DOR), Time to Second Progression (PFS2)

Objective Response Rate (ORR) will be calculated as the rate of patients with a best overall response of partial response (PR) or complete response (CR). Response duration will be calculated from the time of initial response until documented tumor progression.

The Duration of Response (DOR) is defined as the time from initially meeting the criteria for response (CR or PR) until the time of progression by RECIST or death due to underlying disease. DOR will be reported descriptively for each group without comparison between groups.

As additional secondary exploratory end-point (local RECIST assessment), the Time to Second Progression (PFS2) will be assessed in the two study arms. PFS2 is defined as the time from randomization to the time of disease progression or death due to any cause (on any treatment) following the first episode of disease progression.

For patients that are re-treated with Lutathera, a separate analysis for ORR, DoR, and PFS2 will be conducted during the re-treatment phase using the same definition as in Section 12.5.2 for ORR and DoR, except that the new baseline will be based on the tumor assessment which demonstrated progression thus allowing re-treatment with Lutathera. The PFS2 definition is the same as that in Section 12.6.1. This ORR, DoR, and PFS2 analysis during re-treatment will be descriptive only.

It is recognized that the limitation of including cross-over and the lower scans frequency during follow-up will reduce the possibility to reach firm conclusions on PFS2.

After the first progression is confirmed centrally and the second progression is confirmed locally (i.e. after PFS2 event), the sites which do not routinely perform 6-monthly follow up CT/MRI scans for progressive GEP-NET patents can omit this examination at the subsequent patient's visits (no protocol deviation).

8.3.3 Overall Survival

Overall Survival (OS) will be calculated from the randomization date until the day of death due to any cause; OS will not be censored if a patient receives other anti-tumor treatments after study medication.

Overall Survival will also be separately estimated for patients that receive re-treatment with Lutathera. The definition for OS is the same to that in Section 12.5.2, and this analysis with re-treatment patients will be descriptive only.

Survival data will be analyzed at the time of the analysis of the primary end-point (PFS), and will continue to be assessed up to 4 years from the randomization of the last patient or 6 months after the last cross-over/re-treatment dose in the study, whichever occurs last.

8.4 Quality of Life

The impact of treatment on health related QoL will be assessed using the EORTC QLQ-G.I.NET21, EORTC QLQ-C30 and EQ-5D-L5 questionnaires, which will be filled in by the patient prior to know CT scan/MRI result. Changes from baseline will be assessed every 12±1 week from the first treatment date until the end of treatment.

Forms in Country-specific languages will be provided by the Sponsor.

8.5 Safety and Tolerability

8.5.1 Adverse Events

All adverse events (AEs), whether or not spontaneously reported by the patient, will be recorded starting from the signing of the ICF until the end of the Treatment Phase/optional Treatment Extension Phase.

During the Follow up Phase only related SAEs and related AESI other than secondary hematological malignancies will be recorded. AESI of secondary hematological malignancies will be recorded during the whole study irrespective of causality. Definitions and reporting procedures are outlined in Section 10. An Independent Steering Committee Board will evaluate patient's safety throughout the study (Section 10.2). During the Follow-up Phase, concomitant medications must be collected in the eCRF if administered for related SAEs/AESI and/or for secondary hematological malignancies.

During the COVID-19 pandemic that limits or prevents on-site study visits, regular phone or virtual calls will occur for safety monitoring and discussion of the participant´s health status until the participant can again visit the site.

8.5.2 Laboratory Assessments

The laboratory assessments require that blood samples for hematology and blood chemistry, and a urine sample for urinalysis are taken (Table 8-3, and Table 8-4). Laboratory assessments will be performed at the investigational site.

<u>At Screening</u>: all patients will have screening laboratory assessments including hematology and blood chemistry within 3 weeks (preferably 2 weeks in the Lutathera arm) before the projected first treatment date.

<u>During the Treatment Phase (including cross-over and re-treatment):</u>

- In the Lutathera arm: within 2 weeks before and 4±1 weeks after each Lutathera treatment. In addition, for the second, third, and fourth Lutathera treatment, an additional laboratory assessment will be performed on the same day, or within one day prior Lutathera administration. Blood tests performed 4 weeks after any treatment cannot serve as baseline values for the next treatment. After the end of Lutathera treatment, laboratory assessments will be performed every 12±1 weeks (see Table 8-2 and Visit Schedule in Table 8-3).
- In the 60 mg octreotide long-acting arm: throughout the study laboratory assessments will be performed 4 weeks (±3 days) after the first treatment till Week 28, and every 12±1 weeks from Week 36 onwards (see Visit Schedule in Table 8-4).

In the event of a significant laboratory abnormality, or if clinical or laboratory evidence of toxicity occurs, the Investigator will collect additional specimens for repeat or additional analyses, at intervals appropriate to the abnormality. The patient will be closely followed until sufficient information is obtained to determine the cause or the value regresses. Appropriate remedial measures should be taken and the response recorded.

All safety laboratory results must be evaluated by the Investigator before administration of study medication.

Any clinically relevant change from baseline onwards will be recorded on the Adverse Event page of the e-CRF, possibly with a single diagnosis encompassing all changes possibly supporting to the single diagnosis.

During the COVID-19 pandemic, the investigator should consider whether it is possible to arrange for the laboratory tests to be performed at a local laboratory for those patients who cannot come to the clinical site as scheduled. The investigator should collect and file all local lab reports in the patient medical record or chart.

Table 8-2. Laboratory Assessments[1].

Haematology	Blood Chemistry	Pregnancy test
• WBC with differential[2]<br>• Platelets[2]<br>• Hb[2]<br>• MCV<br>• Haematocrit	• BUN or urea<br>• Serum creatinine[3]<br>• Creatinine Clearance[3]<br>• Uric acid<br>• Albumin<br>• Total bilirubin[3]<br>• AP<br>• AST/ASAT<br>• ALT/ALAT<br>• Gamma-GT<br>• Sodium<br>• Potassium<br>• Magnesium<br>• LDH<br>• CgA[4]<br>• GlycoHb (haemoglobin A1C)	• Pregnancy test, *if applicable* (urine pregnancy test is accepted[5])

	• TSH (and free Thyroxine (fT4) if TSH is abnormal)<br>• Calcium<br>• Fasting blood Glucose	

[1] Laboratory assessments performed on the same day or within one day prior to administration of the second, third, and fourth doses of Lutathera must include at minimum:

- serum urea or blood urea nitrogen and creatinine (including creatinine clearance calculation)
- serum potassium, serum total bilirubin, aspartate aminotransferase and alanine aminotransferase
- hemoglobin, platelet count, total leukocyte count, and absolute neutrophil count.

[2] Before each Lutathera dose, patients must meet the criteria for Hb, WBC, and platelets, as defined in the inclusion criteria, at baseline and before each subsequent treatment. If the patient cannot be retreated due to haematological abnormalities, the evaluation must be repeated at least once weekly until re-treatment.

[3] Before each Lutathera dose, patients must meet the criteria for creatinine clearance and total bilirubin as defined in the eligibility criteria at baseline and before every re-treatment. As entry criterion, patients must not have creatinine clearance <40 mL/min calculated by the Cockroft Gault method. During the course of the study, if in the Lutathera arm a 40% increase over the baseline serum creatinine value occurs during the course of treatment, or a decrease of over 40% in creatinine clearance as calculated from serum creatinine concentrations according to Cockcroft-Gault formula, DMT rules must apply (Table 6-4) and patients must also have a measured creatinine clearance (or GFR) performed. Measured creatinine clearance should be through a 24-h urine collection. Total urinary protein should also be measured in this collection. If the measured urinary creatinine clearance shows decrease of ≤40% compared to baseline, treatment can continue.
The Cockcroft-Gault formula allows this estimation based on the occurrence of creatininemia, and correlating patient muscular mass (and so the consequent creatinine production) to weight, gender and age:
*Est. Creatinine Clearance = [[140 - age (yr)]*weight (kg)]/[72*serum Cr(**mg/dL**)]*
(multiply by 0.85 for women)
Or
*Est. Creatinine Clearance = [[140-age(yr)]*weight (kg)]/[0.814* serum Cr(**umol/L**)]*
(multiply by 0.85 for women)

[4] In South Korea only, CgA assessment being discontinued in Q3 2022 due to lab kits shortage

[5] For UK, serum pregnancy test is mandatory at baseline

During the Follow-up Phase, in both study arms laboratory assessments (hematology and blood chemistry) will be performed every 6 months (± 1 month).

8.5.3 Pregnancy Test

Women who receive Lutathera in the study, including those who crossed over to Lutathera and those re-treated with Lutathera, should not procreate during Lutathera treatment and until six months after the end of their last treatment with Lutathera, or until the end of treatment period of the study, whichever is longer. Women in the control arm should not procreate until the end of treatment period.

A pregnancy test (either on urine or blood) must be performed at baseline and within 14 days (preferably 7 days) prior to each Lutathera treatment for every female patient of childbearing potential (Section 5 and Appendix 1).

Ionizing radiations of lutetium (177Lu) oxodotreotide may potentially have temporary toxic effects on female and male gonads (decrease of inhibin-B and concomitant increase of FSH, suggesting radiation damage to Sertoli cells). Fertility is usually restored 12 to 18 months after treatment. The participant will be recommended to seek genetic consultation before starting treatment if the patient wishes to have children after treatment, which includes the option of cryopreservation of sperm or eggs before treatment starts.

For UK only:

A pregnancy test (blood) must be performed at baseline in both arms and within 7 days prior to the first Lutathera treatment. A urine or blood pregnancy test must be performed within 7 days prior to each Lutathera treatment for every female patient of childbearing potential (Section 5 and Appendix 1). A urine or blood pregnancy test must be performed in both arms at the end of the relevant systemic exposure to octreotide.

For Germany only:
A pregnancy test must be performed at baseline in both arms (either on urine or blood). In the Lutathera arm, a urine or blood pregnancy test must be performed monthly during the treatment phase (within 7 days prior to each Lutathera treatment) (Section 5 and Appendix 1) and for the following six months after the end of treatment with Lutathera for every female patient of childbearing potential.

8.5.4 Cardiac Ejection Fraction

Patients with uncontrolled congestive heart failure (NYHA II, III, IV) are not eligible according to exclusion criteria.

Patients with history of congestive heart failure who do not violate this exclusion criterion will undergo an evaluation of their cardiac ejection fraction prior to randomization via echocardiography. The results from an earlier assessment (not exceeding 30 days prior to randomization) may substitute the evaluation at the discretion of the Investigator, if no clinical worsening is noted. The patient's measured cardiac ejection fraction in these patients must be ≥40% before randomization.

8.5.5 ECG

Standard 12-lead ECGs will be performed using the device provided by the Sponsor (central assessment). ECGs will be recorded at baseline, after each Lutathera treatment procedure (following the completion of the amino acids infusion), at the end of study treatment and at the end of study follow up phase to measure the different ECG intervals (RR, PR, QRS, and a more extended QT evaluation according to ICH E14). ECGs will be taken also in the 60 mg octreotide long-acting arm at same time points (before the octreotide long-acting injection). See ECG Manual for further details and visits schedule in Table 8-3, and Table 8-4).

An ECG in triplicate (at least 5 minutes apart) will be taken supine, after 10 minutes rest, and not immediately after a meal. The parameters will be measured as a mean value of minimally 3 beats. The preferred sequence of cardiovascular data collection during study visits is ECG collection first, followed by vital signs, and blood sampling.

Additional, unscheduled, safety ECGs may be repeated at the discretion of the investigator at any time during the study as clinically indicated. Unscheduled ECGs with clinically significant findings should be collected in triplicate. Local cardiologist ECG assessments may also be performed at any time during the study at the discretion of the investigator.

All ECGs, including unscheduled triplicate safety ECGs with clinically relevant findings, collected during the study should be transmitted to the central core ECG laboratory for review.

The Investigator/local cardiologist will note in the source documents (and in the eCRF) whether the ECG is normal or abnormal, as well as the clinical relevance of abnormal ECGs results based on their own review of the ECG tracing. Relevant abnormalities at baseline will be recorded in the medical history page, while changes during the study will be recorded on the Adverse Event page of the eCRF.

The original ECGs on non-heat-sensitive paper or a certified copy on non-heat sensitive paper, appropriately signed, must be collected and archived at the study site. Any identifier details must be redacted e.g. subject initials, date of birth. Each ECG tracing should be labeled with the study number, subject initials (where regulations permit), subject number, date, and kept in the source documents at the study site.

The results of the centrally assessed ECGs are automatically transferred into the clinical database.

8.5.6 Physical Examination and Vital Signs

Physical examinations will be performed by the Investigator, or qualified designee. All body systems will be examined and any relevant findings will be documented in the source documents and eCRF. Physical examinations should include heart rate, blood pressure and weight measurement (height will only be measured at baseline). Blood pressure and pulse rate will be performed after the patient rests for 5 minutes. For each patient, all blood pressure recordings shall be made using the same type of instrument (i.e., manual BP recording vs. automatic digital vital signs monitor) on the same arm.

Significant findings that are present prior to baseline will be recorded in the medical history page, while changes during the study (including significant changes of the symptoms due to the underlying disease vs baseline) will be recorded on the Adverse Event page of the eCRF.

8.5.7 Karnofsky Performance Score

KPS forms must be completed by a medical professional at each treatment and follow-up visit, and before any current clinical information is given to the patient (Appendix 3).

8.5.8 Ultrasonic examination of the gallbladder (Germany only)

Only for Germany:

Ultrasonic examination of the gallbladder before and at six-monthly intervals during Octreotide LAR treatment in order to detect and prevent possible cholecystitis and biliary duct dilatation risks for the patients is recommended according to the Octreotide label.

8.5.9 SPECT/CT and whole body planar imaging (Germany only)

Only for Germany:

SPECT/CT imaging and whole body planar imaging should be performed after each Lutathera injection (local assessment only), as per local standards. The dosimetry results will be collected in eCRF.

In other Countries, physicians may also decide to perform SPECT/CT scans for treatment verification purposes following the drug administration(s), based on institutional guidelines and their discretion.

8.5.10 Assessments Schedule

The Assessment Schedule in the two study arms is shown in Table 8-3, and Table 8-4.

In the Lutathera arm, safety laboratory assessments will be performed within 2 weeks before and 4±1 weeks after each Lutathera treatment. In addition, for the second, third and fourth Lutathera treatment, an additional safety laboratory assessment will be performed on the same day or within one day before each treatment (see Table 8-2).

The Lutathera treatment / assessment schedule shown in Table 8-3 is idealized in the sense that all Lutathera treatments occur at defined 8-week interval (±1 week). In the event that the intervals are greater than the defined 8-week interval (±1 week), the investigator will need to ensure that the correct safety assessments before and after each treatment have been conducted (safety assessments must be performed within 2 weeks before and 4 weeks after each Lutathera treatment, see Table 8-3).

Exams to be done in relation to the Lutathera treatments (within two weeks before the infusion, one the day of the infusion, 4±1 weeks after infusion) can be combined with those required at other visits during the study, if less than 2 weeks apart.

During the Follow-up Phase, in both study arms study visits will be performed every 6 months (± 1 month) up to 3 years.

Table 8-3. Assessment Schedule Lutathera Arm.

Visit Name	Screening	Treatment																						Further Visits after 72 weeks[1]		EOT or early termination	Optional re-treatment (2-4 Lutathera cycles)	3 years follow-up	EOS or early FUP termination
**Weeks**	**-2 - 0**	**0**	**4**	**6**	**8**	**12**	**14**	**16**	**20**	**22**	**24**	**28**	**32**	**36**	**40**	**44**	**48**	**52**	**56**	**60**	**64**	**68**	**72**	**Every 4 weeks**	**Every 12 weeks**	**Within 4 weeks from last treatment**		**Every 6 months**	**Last FUP visit**
**Treatment**		⬇ ↓			⬇ ↓			⬇ ↓			⬇ ↓	↓	↓	↓	↓	↓	↓	↓	↓	↓	↓	↓	↓	↓	↓		2-4x⬇		
Informed consent	X																										Complete all assessments and procedures in Table 8-3 from week -2 (except demography, INC/EXC criteria, cancer diagnosis and grading, medical history, randomization, QoL) until disease progression, after which EoT visit is done and patient proceeds into follow up. Patients must sign the new consent and have SRI imaging documenting tumor uptake before re-treatment. SSA administration at investigator judgment.		
Demography	X																												
Inclusion/exclusion criteria	X																												
Cancer diagnosis and grading	X																												
Prior therapy for carcinoid tumor	X																												
Medical history	X																												
Physical Exam	X	X				X					X			X			X			X			X		X	X			X
Height	X																												
Weight	X	X	X	X	X	X	X	X	X	X	X	X		X			X			X			X		X	X			X
Vital Signs	X	X			X	X		X			X			X			X			X			X		X	X			X
Karnofsky Performance	X	X				X					X			X			X			X			X		X	X		X	X
Cardiac Ejection Fraction[2]	X																												
Randomization and Lutathera order (after all elig bility criteria are verified)[3]	X																												
Blood chemistry	X		X	X	X	X	X	X	X	X	X	X		X			X			X			X		X	X		X	X
Hematology	X		X	X	X	X	X	X	X	X	X	X		X			X			X			X		X	X		X	X

Assessment Schedule Lutathera Arm – cont.

Visit Name	Screening	Treatment																					Further Visits after 72 weeks[1]	EOT or early termination		Optional re-treatment (2-4 Lutathera cycles)	3 years follow-up	EOS or early FUP termination
**Weeks**	-2 - 0	0	4	6	8	12	14	16	20	22	24	28	32	36	40	44	48	52	56	60	64	68	72	Every 4 weeks	Every 12 weeks	Within 4 weeks from last treatment		Every 6 months	Last FUP visit
**Treatment**		↓ ↓			↓ ↓			↓ ↓			↓ ↓	↓	↓	↓	↓	↓	↓	↓	↓	↓	↓	↓	↓	↓	↓		2-4x ↓↓		
Pregnancy Test	X		X[4]		X	X[4]		X	X[4]		X	X[4]	X[4]	X[4]	X[4]	X[4]	X[4]	X[4]								X[4]	Complete all assessments and procedures in Table 8-3 from week -2 (except demography, INC/EXC criteria cancer diagnosis and grading, medical history, randomization, QoL) until disease progression, after which EoT visit is done and patient proceeds into follow up. Patients must sign the new consent and have SRI imaging documenting tumor uptake before re-treatment. SSA administration at investigator judgment[10].		
Serum CgA[5]	X					X					X			X			X			X			X		X	X			X
SRI (eg SomaKit or Netspot or OctreoScan)[6]	X																												
SPECT/CT and WBPI[7]		X[7]			X[7]			X[7]			X[7]																		
CT/MRI (central re-assessment during treatment phase)	X							X			X			X			X			X			X		X	X[9]		X	X[11]
ECG (central re-assessment; Sponsor device) [at the end of amino acids infusion during treatment]	X	X			X			X			X															X			X
Adverse Events[8]	X	X	X	X	X	X	X	X	X	X	X	X	X	X	X	X	X	X	X	X	X	X	X	X	X	X		X	X
Prior/Concomitant Medications/New Anticancer therapy	X	X	X	X	X	X	X	X	X	X	X	X	X	X	X	X	X	X	X	X	X	X	X	X	X	X		X[12]	X[12]
Lutathera/Amino Acid/Anti-emetics		X			X			X			X																		
Octreotide long-acting 30 mg	Injections to be administered at least after 4 hours after each Lutathera treatment or the day after (preferred option), Last injection should be administered at least 6 weeks before each Lutathera infusion																												
Octreotide SC	Octreotide s.c. injection can be given as needed but must be stopped 24h before each Lutathera infusion																												
QoL(QLQ-G.I.NET21, QLQ-C30, EQ-5D-5L)	X	X				X					X			X			X			X			X		X	X			X
Survival information	X	X	X	X	X	X	X	X	X	X	X	X	X	X	X	X	X	X	X	X	X	X	X	X	X	X		X	X

-TREATMENT PHASE:

- TREATMENT: Lutathera; 4 administrations at 8±1-week intervals
- TREATMENT: 30 mg octreotide long-acting injections to be administered the day after each Lutathera infusion

Due to the 6 weeks washout prior to each Lutathera injection, octreotide long-acting 30 mg is administered every 8 weeks during Lutathera treatment (including during the optional treatment extension phase) and every 4 weeks after last Lutathera treatment.
IF REQUIRED, RESCUE TREATMENTS: Octreotide s.c. injections (to be stopped 24 h before each Lutathera infusion)

1- Before the PFS primary analysis (i.e. 99 evaluable and centrally confirmed disease progressions or death events), patients continue the Treatment Phase until progression. After the PFS primary analysis, the Treatment Phase duration becomes fixed (72 weeks).
2- Via echocardiography, only in patients with history of congestive heart failure (patients with uncontrolled congestive heart failure (NYHA II, III, IV) aren't eligible according to exclusion criteria)
3- Randomization must be performed once all screening assessments are complete. The screening phase must be shortened as much as possible in order to treat the patients possibly within 2 weeks from the consent date. As Lutathera production and shipment will take approximately 12 days to be arranged, if a patient is randomized in the Lutathera arm, Lutathera first dose must be ordered immediately after randomization.
The baseline CT/MRI scan should be taken possibly on the same day of randomization or immediately before (within 1 week) to ensure that it reflects the disease status closely before the therapy start.
4- Additional pregnancy tests for Germany only. The pregnancy test at EoT is for Germany and UK.
5- CgA assessment being discontinued in Q3 2022 due to lab kits shortage in South Korea only
6- There is no central ‘real time’ assessment of the SRI images; the images should be submitted to the central imaging center possibly within 1 month.
7- SPECT/CT and WBPI post Lutathera injection requested in Germany only.
8- Only related SAEs & AESI will be collected during the Follow-up Phase, with the exception of AESI of secondary hematological malignancies which will be collected during the Follow-up Phase irrespective of causality (see Section 10.1.5 for AESI definition).
9- Only if a scan was not taken in the previous 12 weeks; if no post-baseline scan is available, a scan is needed at EOT.
10- For patients who entered the Lutathera re-treatment phase, since there are no procedures requiring in-person patient’s attendance, the following visits can be performed by phone: Visits W32, 40, 44, 52, 56, 64 and 68 following Lutathera re-treatment (for Germany only: visits W56, 64 and 68).
Post progression Re-Treatment Eligibility CT/MRI scan doesn’t need to be repeated if performed within the previous 12 weeks. Both Re-treatment Eligibility SRI and CT/MRI scans need to be submitted to the central imaging center for a second review: there is no central ‘real time’ assessment of the Re-treatment Eligibility SRI uptake; however, the images should be submitted to the central imaging center possibly within 1 month.
11- Only if a scan was not taken in the previous 6 months.
12- During Follow-up Phase and at the EOS visit, concomitant medications must be collected only if administered for related SAEs/AESI and/or for secondary hematological malignancies. In addition, all new anticancer therapies administered must be collected till EOS.

Table 8-4. Assessment Schedule Octreotide Long-Acting 60 mg Arm.

Visit Name	Screening	Treatment																			Further Visits after 72 weeks[1]		EOT or early termination	Optional cross-over (4 Lutathera cycles)[2]	3 years follow-up	EOS or early FUP termination
**Weeks**	**-2 - 0**	**0**	**4**	**8**	**12**	**16**	**20**	**24**	**28**	**32**	**36**	**40**	**44**	**48**	**52**	**56**	**60**	**64**	**68**	**72**	**Every 4 weeks**	**Every 12 weeks**	**Within 4 weeks from last treatment**		**Every 6 months**	**Last FUP visit**
**Treatment**		↓	↓	↓	↓	↓	↓	↓	↓	↓	↓	↓	↓	↓	↓	↓	↓	↓	↓	↓	↓	↓		4x ↓↓		
Obtain informed consent	X																							Complete all assessments in Table 8-3 from week -2 (except demography, cancer diagnosis and grading, medical history, randomization) to Week 24 (last Lutathera dose) after which EoT visit is done and patient proceeds into follow up. Patients must sign the new consent before cross-over and have SRI imaging documenting tumor uptake. All protocol inclusion/exclusion criteria must be re-verified.		
Demography	X																									
Inclusion/exclusion criteria	X																									
Cancer diagnosis and grading	X																									
Prior therapy for carcinoid tumor	X																									
Medical history	X																									
Physical Exam	X	X			X			X			X			X			X			X		X	X			X
Height	X																									
Weight	X	X	X	X	X	X	X	X	X		X			X			X			X		X	X			X
Vital Signs	X	X		X	X	X		X			X			X			X			X		X	X			X
Karnofsky Performance	X	X			X			X			X			X			X			X		X	X		X	X
Cardiac Ejection Fraction[3]	X																									
Randomization (after all eligibility criteria are verified)[4]	X																									
Blood chemistry	X		X	X	X	X	X	X	X		X			X			X			X		X	X		X	X
Hematology	X		X	X	X	X	X	X	X		X			X			X			X		X	X		X	X

Assessment Schedule Octreotide Long-Acting 60 mg Arm – cont.

Visit Name	Screening	Treatment																			Further Visits after 72 weeks[1]		EOT or early termination	Optional Treatment Extension (4 Lutathera cycles)[2]	3 years follow-up	EOS or early FUP termination
**Weeks**	**-2 - 0**	**0**	**4**	**8**	**12**	**16**	**20**	**24**	**28**	**32**	**36**	**40**	**44**	**48**	**52**	**56**	**60**	**64**	**68**	**72**	**Every 4 weeks**	**Every 12 weeks**	**Within 4 weeks from last treatment**		**Every 6 months**	**Last FUP visit**
**Treatment**		↓	↓	↓	↓	↓	↓	↓	↓	↓	↓	↓	↓	↓	↓	↓	↓	↓	↓	↓	↓	↓		4x ↓↓		
Pregnancy Test	X																						X[5]	Complete all assessments in Table 8-3 from week -2 (except demography, cancer diagnosis and grading, medical history, randomization) to Week 24 (last Lutathera dose), after which EoT visit is done and patient proceeds into follow up. Patients must sign the new consent before cross-over and have SRI imaging documenting tumor uptake. All protocol inclusion/exclusion criteria must be re-verified.		
Serum CgA	X				X			X			X			X			X			X		X	X			X
SRI (SomaKit or Netspot or OctreoScan)[7]	X																									
CT/MRI (central re-assessment during treatment phase)	X					X		X			X			X			X			X		X	X[10]		X	X[11]
ECG (central re-assessment; Sponsor device)	X	X		X		X		X															X			X
PK (central assessment)[8]			X		X	X		X																		
Adverse Events[9]	X	X	X	X	X	X	X	X	X	X	X	X	X	X	X	X	X	X	X	X	X	X	X		X	X
Prior/Concomitant Medications/New Anticancer therapy	X	X	X	X	X	X	X	X	X	X	X	X	X	X	X	X	X	X	X	X	X	X	X		X[12]	X[12]
Octreotide long-acting 60 mg	Injections to be administered every 4 weeks. Patients who were SSA-naïve before enrolment in the study, should receive first planned dose at 30mg and the second (and subsequent) planned dose(s) at 60mg (full dose).																									
Octreotide SC	Octreotide s.c. injection can be given as needed																									
QoL(QLQ-G.I.NET21, QLQ-C30, EQ-5D-5L)	X	X			X			X			X			X			X			X		X	X			X
Survival information	X	X	X	X	X	X	X	X	X	X	X	X	X	X	X	X	X	X	X	X	X	X	X	X	X	X

-TREATMENT PHASE:
↓ TREATMENT: 60 mg octreotide long-acting injections every 4 weeks (±3 days)
↓ TREATMENT: Lutathera; 4 administrations at 8±1-week intervals
-OPTIONAL EXTENSION TREATMENT PHASE:
↓ TREATMENT: 30 mg octreotide long-acting injections to be administered the day after each Lutathera infusion

Due to the 6 weeks washout prior to each Lutathera injection, octreotide long-acting 30 mg is administered every 8 weeks during Lutathera treatment. IF REQUIRED, RESCUE TREATMENTS: Octreotide s.c. injections (to be stopped 24 h before each Lutathera infusion).

1- Before the PFS primary analysis (i.e. 99 evaluable and centrally confirmed disease progressions or death events), patients continue the Treatment Phase until progression. After the PFS primary analysis, the Treatment Phase duration becomes fixed (72 weeks).
2- Any progressive patient (based on central imaging assessment) has the option to enroll for post-progression cross-over to Lutathera, upon signature of a new consent, if the eligibility criteria are met. If RECIST progression occurs after Week 72 post the primary end point analysis, the decision to enroll the patient in cross-over will be based on local assessment.
The EoT visit and evaluation of cross-over eligibility can occur on the same day. If these two visits are done on separate days, EoT can be done remotely by phone, and all applicable assessments can be done at the cross-over eligibility visit. If a patient continues to cross-over, 28-day window for EoT visit does not need to apply.
3- Via echocardiography, only in patients with history of congestive heart failure (patients with uncontrolled congestive heart failure (NYHA II, III, IV) aren't eligible according to exclusion criteria).
4- Randomization must be performed once all screening assessments are complete. The screening phase must be shortened as much as possible in order to treat the patients possibly within 2 weeks from the consent date.
The baseline CT/MRI scan should be taken possibly on the same day of randomization or immediately before (within 1 week) to ensure that it reflects the disease status closely before the therapy start.
5- Additional pregnancy test for UK only.
6- CgA assessment being discontinued in Q3 2022 due to lab kits shortage in South Korea only
7- There is no central 'real time' assessment of the SRI images; the images should be submitted to the central imaging center possibly within 1 month.
8- PK assessment is done in all Countries, except China.
9- Only related SAEs & AESI will be collected during the Follow-up Phase, with the exception of AESI of secondary hematological malignancies which will be collected during the Follow-up Phase irrespective of causality (see Section 10.1.5 for AESI definition).
10- Only if a scan was not taken in the previous 12 weeks; if no post-baseline scan is available, a scan is needed at EOT.
Post progression Cross-Over Eligibility CT/MRI scan doesn't need to be repeated if performed within the previous 12 weeks. Both Cross-over Eligibility SRI and CT/MRI scans need to be submitted to the central imaging center for a second review: there is no central 'real time' assessment of the Cross-over Eligibility SRI uptake; however, the images should be submitted to the central imaging center possibly within 1 month.
11- Only if a scan was not taken in the previous 6 months
12- During Follow-up and at the EOS visit, concomitant medications must be collected only if administered for related SAEs/AESI and/or for secondary hematological malignancies. In addition, all new anticancer therapies administered must be collected till EOS.

8.6 Pharmacokinetics

This section is applicable to all countries, except for China. No PK samples will be collected in China.

The steady-state plasma concentrations of high dose octreotide long-acting will be assessed in the control arm of the study.

All samples will be taken by either direct venipuncture or indwelling cannula inserted in a forearm vein. Blood samples (2.5 mL each sample) will be collected to yield approximately 1 mL plasma for analysis of octreotide concentration. Immediately after blood is drawn into K3EDTA tubes, they should be inverted gently several times to ensure the mixing of tube contents. Prolonged exposure to rubber stopper should be avoided. The tube should be placed upright in tube rack surrounded by ice until centrifugation. Within 20 minutes, the sample should be centrifuged between 3ºC and 5ºC for 10 minutes at approximately 1000 x g. Immediately after centrifugation, 1.0 mL plasma should be transferred to a polypropylene screw-cap tube immersed in dry ice. The storage temperature for PK samples should be at or below minus 20°C (-20°C). The samples will be sent to an external laboratory for central assessment (see further details in the PK Manual).

Table 8-5. Octreotide long-acting PK blood collection plan

CONTROL ARM				
Sample	Volume (mL)	Week	PK Collection #	Scheduled time (h)
Blood	2.5	4	1	0 (pre-dose)*
Blood	2.5	12	2	0 (pre-dose)*
Blood	2.5	16	3	0 (pre-dose)*
Blood	2.5	24	4	0 (pre-dose)*
*Blood sample for PK must be collected before octreotide long-acting injection.				

8.6.1 Analytical method

Octreotide plasma concentration will be measured using a validated radio-immunoassay (RIA). All PK samples will be shipped to the central laboratory where they will be stored and forwarded to the pharmacokinetic facility for bioanalysis.

8.7 Biomarkers

Serum Chromogranin A (CgA) will be locally assessed at 12-weeks intervals during the treatment. The CgA assessment is discontinued in South Korea in Q3 2022 due to lab kits shortage.

8.8 Imaging

Central, blinded, real-time IRC (Independent Review Committee) CT/MRI assessment is implemented in this trial. Images will be also assessed by the site local radiologist.

The primary efficacy end-point of the study is PFS as measured by objective tumor response, which is determined by RECIST criteria, Version 1.1 (Eisenhauer, et al., 2009). Sponsor will notify all the Centers and their Ethic Committees as soon as the 99 PFS primary End-Points have occurred.

Changes from randomization date will be assessed centrally until the completion of the Treatment Phase, unless the patient progresses or dies. Additional PFS data will be collected up to 3 years (follow-up phase) from the end of Treatment Phase of the last patient. If a patient participates after progression to the optional Treatment Extension Phase or optional Re-treatment Phase, scans will be assessed locally according RECIST 1.1, according to the frequency indicated in Table 8-3, and Table 8-4.

During the Treatment Phase, restaging is scheduled at week 16±1, week 24±1 and then every 12 ±1 weeks (from the date of randomization). The first CT/MRI re-staging at week 16 will ensure that control arm patients who are SSA-naïve will receive at least 12 weeks of octreotide long-acting full dose (60mg) after the completion of the dose escalation phase (see Section 6.5.2), while patients in the Lutathera arm will receive at least 2 Lutathera doses before the first tumor response assessment. As described in Section 12.8.1, the study sample size has been estimated considering a median PFS of approximately 15 months in the control arm and 30 months in the Lutathera arm, therefore the proposed CT/MRI schedule, and in particular, the first re-staging at week 16, is consistent with the protocol assumptions.

Clinical suspicion of disease progression before week 16 and at any time during the study requires a physical examination and imaging assessments to be performed promptly rather than waiting for the next scheduled imaging assessment. These additional scans should be provided to the IRC for central assessment. If an off-schedule imaging assessment is performed because progression is suspected, subsequent imaging assessments should be performed in accordance with the original imaging schedule.

In case of delays, the reason of the delay must be documented, and the CT/MRI assessment must be done as soon as possible. When a CT/MRI assessment is > 6 weeks earlier or later than the original schedule, the next reassessments must be discussed and adjusted with the Medical Monitor of the study in a way to progressively return to the original schedule.

Every effort should be made to avoid differences between these timings for patients in the two arms, unless this would result in a deviation of the protocol (re-staging at week 16±1, week 24±1 and then every 12 ±1 weeks from randomization). The CT/MRI scans should be avoided in the 6-weeks period following Lutathera administration due to the PRRT-induced pseudo-progression phenomenon described in literature (Brabander, et al., Pitfalls in the response evaluation after peptide receptor radionuclide therapy with [177Lu-DOTA0,Tyr3]octreotate., 2017b). If a CT/MRI scan is planned on the same day of a Lutathera treatment, the scan must be taken before the drug administration. As reported by Brabander et al. (2017b), an increase in the diameter of lesions of 10% or more on imaging was seen in 9% of patients with SD at 6 weeks

post PRRT and was reversible at 6 months follow-up in 50% of patients. This transient increase was probably caused by inflammation causing localized oedematous tissue at the site of the metastases and not based on progression. This radiogenic oedema has been described previously for PRRT and external beam radiation of brain tumors (Kneifel, et al., 2006; Barani & Larson, 2015). In studies with cytokines, cancer vaccines and monoclonal antibodies an early increase in tumor burden has been demonstrated in patients with CR, PR or SD as treatment outcome. This phenomenon was described as pseudo-progression and resulted in a new response method, the immune-related response criteria (irRC) (Wolchok, et al., 2009). This increase in the tumor size after immunotherapy is probably related to infusion of lymphocytes and macrophages in the tumor and new lesions are not always considered as PD.

In case of discrepancies between Investigator and central assessor on the evaluation of the progression of disease, the following is recommended:

1. Investigator assessment: non-PD; central assessment: PD. The Treatment Phase of the study is terminated and the patient should proceed to the Follow-up Phase. The investigator may request the continuation of treatment (Lutathera until the cumulative dose limit has been reached and/or octreotide long-acting provided by the Sponsor according to randomization; 12-week local tumor assessments; safety assessments as in the treatment/assessment phase of the study) until progression has been documented by the Investigator, thereafter, the patient will proceed to the follow-up assessment phase. Alternatively, the patient can be enrolled in the optional Treatment Extension Phase, if the eligibility criteria are met (see Section 5.2.1).
2. Investigator assessment: PD; central assessment: non-PD. The Treatment Phase of the study should be continued as planned. However, the Investigator may decide to withdraw the patient from the Treatment Phase because of unethical continuation of the study in his/her opinion, and start the Follow-up Phase of the study. Such cases should be limited as much as possible and discussed with the Sponsor Medical Monitor before withdrawing the patient and all attempts should be made to continue central scans assessment until central confirmation of progression according to RECIST.

The coded medical images will be used primarily for analysis as described in this protocol; however, the images may also be used for the development and evaluation of new analysis methods directly related to the area of research that this study covers.

The methods for assessment and recording are specified in the imaging manual and imaging charter.

See Section 8.3.1 for further details.

9 Study discontinuation and completion

9.1 Discontinuation

9.1.1 Discontinuation of study treatment

Discontinuation of study treatment for a subject occurs when study treatment is stopped earlier than the protocol planned duration, and can be initiated by either the subject or the investigator.

The investigator must discontinue study treatment for a given subject if, he/she believes that continuation would negatively impact the subject's well-being.

Study treatment must be discontinued under the following circumstances:

- Disease progression
- Unacceptable adverse event, including meeting stopping criteria as described in Section 6.5.2
- Subject/guardian decision
- Pregnancy
- Use of prohibited treatment as per recommendations in Section 6.2.3
- Any situation in which study participation might result in a safety risk to the subject.

If discontinuation of study treatment occurs, the investigator should make a reasonable effort to understand the primary reason for the subject's premature discontinuation of study treatment and record this information.

In extraordinary situations due to the very short half-life of radionuclides, e.g. a sudden temporary supply disruption and/or a quality finding after release of the product, the investigator may immediately be:

1. advised on how to manage participants in screening or on treatment;
2. informed to withhold doses from impacted batch and not administer them to the participant;

and/or

3. asked to closely monitor participants who have received a dose from an impacted batch for adverse events.

Subjects who discontinue study treatment or who decide they do not wish to participate in the study further should NOT be considered withdrawn from the study UNLESS they withdraw their consent. Where possible, they should return for the assessments indicated in the assessment schedule. If they fail to return for these assessments for unknown reasons, every effort (e.g. telephone, e-mail, letter) should be made to contact the subject/predesignated contact as specified in the lost to follow-up section (Section 9.1.3). This contact should preferably be done according to the study visit schedule.

If the subject cannot or is unwilling to attend any visit(s), the site staff should maintain regular telephone contact with the subject, or with a person pre-designated by the subject. This telephone contact should preferably be done according to the study visit schedule.

After study treatment discontinuation, at a minimum, in abbreviated visits, the following data should be collected at clinic visits or via telephone/email contact (per assessment schedule in Table 8-3 and Table 8-4):

- new / concomitant treatments including any radiotherapy, surgical procedure or new anticancer therapy
- adverse events/Serious Adverse Events
- survival status

During the COVID-19 pandemic, for patients who are unable to come to the study site for their scheduled visit, conducting patient study visits on schedule by telephone may be possible. As patient safety is the number one concern, patients may need to discontinue treatment based on the investigator's clinical judgment or patient's own decision. The investigator should document in the source notes as clearly as possible the decisions made around continuing or discontinuing study treatment for a patient and the risk-benefit for each case as it may arise. The investigator should consider if alternative therapeutic options exist and may be warranted based on the individual benefit-risk for the patient.

9.1.1.1 Replacement policy

Not applicable. Subjects will not be replaced on study.

A subject can be re-screened with a new study number when intercurrent conditions emerging after consent signature and impeding the planned randomization are resolved. Such cases must be discussed and approved by the Sponsor. The site will be informed about the procedures to be followed for re-screening.

9.1.2 Withdrawal of informed consent

Withdrawal of consent/opposition to use of data and/or biological samples occurs in countries where the legal justification to collect and process the data is based on consent and when a participant:

- Explicitly requests to stop use of their data

and

- No longer wishes to receive study treatment

and

- Does not want any further visits or assessments (including further study-related contacts)

In this situation, the investigator should make a reasonable effort (e.g. telephone, e-mail, letter) to understand the primary reason for the subject's decision to withdraw his/her consent and record this information.

Study treatment must be discontinued, and no further assessments conducted, and the data that would have been collected at subsequent visits will be considered missing. All efforts should be made to have any study drug returned to the Investigator.

Further attempts to contact the subject are not allowed unless safety findings require communicating or follow-up. All efforts should be made to complete the assessments prior to study withdrawal. A final evaluation at the time of the subject's study withdrawal should be made as detailed in the assessment table.

Advanced Accelerator Applications will continue to retain and use all research results (data) that have already been collected for the study evaluation.

9.1.3 Lost to follow-up

For subjects whose status is unclear because they fail to appear for study visits without stating an intention to discontinue or withdraw, the investigator must show "due diligence" by documenting in the source documents steps taken to contact the subject, e.g. dates of telephone calls, registered letters, etc. A subject should not be considered as lost to follow-up until due diligence has been completed or until the end of the study.

9.1.4 Early study termination by the sponsor

The study can be terminated by Advanced Accelerator Applications at any time for any reason. This may include reasons related to the benefit/ risk assessment of participating in the study, practical reasons (including slow enrollment), or for regulatory or medical reasons. In taking the decision to terminate, Advanced Accelerator Applications will always consider the subject welfare and safety. Should early termination be necessary, subjects must be seen as soon as possible (provide instruction for contacting the subject, when the subject should stop taking drug, when the subject should come for a final visit) and treated as a prematurely withdrawn subject. The investigator may be informed of additional procedures to be followed in order to ensure that adequate consideration is given to the protection of the subject′s interests. The investigator or sponsor depending on the local regulation will be responsible for informing IRBs/IECs of the early termination of the trial.

9.2 Study completion and post-study treatment

Subjects will be followed during the study per the schedule of assessments (refer to Table 8-3 and Table 8-4).

Before the PFS primary analysis (99 evaluable and centrally confirmed disease progressions or death events), patients continue the Treatment Phase until progression; after the PFS primary analysis, the Treatment Phase duration becomes fixed (72 weeks).

Following the end of treatment or early treatment discontinuation, alive patients will enter in the Follow up Phase unless unfeasible (e.g. consent withdrawal, patient lost to follow-up). All treated subjects should have a safety follow up visit conducted 30 days after last administration of study treatment (EoT visit). The information collected is kept as source documentation. Documentation of attempts to contact the subject should be recorded in the source documentation.

At any time during the study (before or after the primary End-Point analysis) any progressive patient (based on central imaging assessment) immediately ceases the Treatment Phase and proceeds to the Follow-up Phase assessment.

Patients randomized in the control arm have the option to enroll for post-progression cross-over upon signature of a new consent (optional Treatment Extension Phase) and receive maximum 4 cycles of Lutathera (7.4 GBq / 200 mCi x 4 cycles; cumulative dose: 29.6 MBq / 800mCi) plus octreotide long-acting every 8 weeks. For these patients, the EoT visit and evaluation of cross-over eligibility can occur on the same day. If these two visits are done on separate days, EoT can be done remotely by phone, and all applicable assessments can be done at the cross-over eligibility visit. If a patient continues to cross-over, 28-day window for EoT visit does not need to apply.

Patients randomized in the Lutathera arm have the option to enroll for post-progression Re-treatment Phase upon signature of a new consent and receive 2-4 additional cycles of Lutathera (7.4 GBq / 200 mCi) every 8 weeks. Re-treatment Phase will last until a locally confirmed disease progression or until EoS, whichever occurs first.

The control arm patients who will consent to post-progression cross-over with Lutathera, and the Lutathera arm patients who will consent to post-progression re-treatment with Lutathera, will continue study assessments as planned in Table 8-4; these patients will enter in the Follow up Phase after the end of Lutathera treatment.

The End of Study (EOS) is when 4 years have elapsed from the randomization of the last patient, or 6 months after the last cross-over or re-treatment dose, whichever occurs last. The time window to start cross-over/re-treatment is further discussed in Sections 5.2.1, 5.2.2 and 9.2.1.

At the End of Treatment (EOT) or End of Study (EOS) or early discontinuation visit, any repeat assessments associated with this visit should have been documented and followed-up appropriately by the Investigator, or in the event of an early study termination decision, the date and reason of that decision.

At the EoT, a CT/MRI scan for RECIST assessment is required only if not taken in the previous 12 weeks. If no post-baseline scan is available, a scan is needed at EoT. At the EoS, a CT/MRI scan for RECIST assessment is required only if a scan was not taken in the previous 6 months.

Each subject will complete their EOS visit at the end of the study (after 3 years of follow up from the end of the Treatment Phase, at early withdrawal from the study, death, withdrawal of consent, lost to follow-up or when the end of study is communicated by Sponsor). The last 6-monthly follow-up visit will be considered EOS visit, when all assessments need to be performed as indicated in the Assessment Schedule for EOS.

Post relapse therapy will be permitted during the study in the Follow-up Period per Investigator discretion, but not provided or administered as study treatment.

The final OS analysis will be performed when the last subject completes their EOS visit. All available data from all subjects up to this cut-off date will be analyzed. All adverse events during the study period must be reported as described in Section 10.

In the event the study is terminated due to futility, study subjects currently on study treatment in the Treatment Phase and who are still deriving clinical benefit will be eligible to continue their Lutathera treatment through a locally established Advanced Accelerator Applications program. Lutathera treatment through a locally established Advanced Accelerator Applications program will not be part of this clinical trial and will follow the applicable local regulations.

9.2.1 Post-trial access to Lutathera

The time window to start re-treatment in this study is within 4 years after the last patient has been randomized (Section 3.4). Patients who are eligible to Lutathera re-treatment beyond this window and would derive clinical benefit from it based on the investigator's evaluation may receive post-trial access (PTA) to Lutathera. PTA to Lutathera may be granted via Post Study Drug Supply (PSDS) programs, based on local regulation in a non-trial setting. The PSDS must comply with local laws and regulations in the participating trial countries.

10 Safety monitoring and reporting

10.1 Definition of adverse events and reporting requirements

10.1.1 Adverse events

An adverse event (AE) is any untoward medical occurrence (e.g., any unfavorable and unintended sign [including abnormal laboratory findings], symptom or disease) in a subject or clinical investigation subject after providing written informed consent for participation in the study. Therefore, an AE may or may not be temporally or causally associated with the use of a medicinal (investigational) product.

The investigator has the responsibility for managing the safety of individual subject and identifying adverse events.

Abnormal laboratory values or test results occurring after informed consent constitute adverse events only if they induce clinical signs or symptoms, are considered clinically significant, require therapy (e.g., hematologic abnormality that requires transfusion or hematological stem cell support), or require changes in study medication(s).

The investigator has the responsibility for managing the safety of individual subject and identifying adverse events.

AAA qualified medical personnel will be readily available to advise on trial related medical questions or problems.

Adverse events that begin or worsen after informed consent must be recorded in the Adverse Events eCRF. Conditions that were already present at the time of informed consent must be recorded in the Medical History page of the subject's eCRF. Adverse event monitoring must be continued for at least 30 days (or 5 half-lives, whichever is longer) following the last dose of study treatment. Adverse events (including lab abnormalities that constitute AEs) must be described using a diagnosis whenever possible, rather than individual underlying signs and symptoms. When a clear diagnosis cannot be identified, each sign or symptom must be reported as a separate Adverse Event.

The occurrence of adverse events must be sought by non-directive questioning of the subject at each visit during the study. Adverse events also may be detected when they are volunteered by the subject during or between visits or through physical examination findings, laboratory test findings, or other assessments.

Adverse events must be recorded under the signs, symptoms, or diagnosis associated with them, accompanied by the following information (as far as possible) (if the event is serious refer to Section 10.1.2):

1. The Severity grade OR the Common Toxicity Criteria (CTC) AE grade.
    a. If "severity grade" is selected, include the following:
        i. mild: usually transient in nature and generally not interfering with normal activities

   ii. moderate: sufficiently discomforting to interfere with normal activities
   iii. severe: prevents normal activities
  b. If "CTCAE grade" is selected, include the following:
   i. Adverse events will be assessed and graded from 1 to 5.
2. Its relationship to the study treatment. If the event is due to lack of efficacy or progression of underlying illness (i.e. progression of the study indication) the assessment of causality will usually be 'Not suspected.' The rationale for this guidance is that the symptoms of a lack of efficacy or progression of underlying illness are not caused by the trial drug, they happen in spite of its administration and/or both lack of efficacy and progression of underlying disease can only be evaluated meaningfully by an analysis of cohorts, not on a single patient
3. Its duration (start and end dates) or if the event is ongoing, an outcome of not recovered/not resolved must be reported.
4. Whether it constitutes a SAE (see Section 10.1.2 for definition of SAE) and which seriousness criteria have been met.
5. Action taken regarding with study treatment
6. All adverse events must be treated appropriately. Treatment may include one or more of the following:
   - Dose not changed
   - Dose Reduced
   - Drug interrupted/withdrawn
7. Its outcome i.e., its recovery status or whether it was fatal

If the event worsens the event should be reported a second time in the eCRF noting the start date when the event worsens in toxicity. For grade 3 and 4 adverse events only, if improvement to a lower grade is determined a new entry for this event should be reported in the eCRF noting the start date when the event improved from having been Grade 3 or Grade 4.

Conditions that were already present at the time of informed consent should be recorded in the medical history of the patient.

Adverse events (including lab abnormalities that constitute AEs) should be described using a diagnosis whenever possible, rather than individual underlying signs and symptoms.

Once an adverse event is detected, it must be followed until its resolution or until it is judged to be permanent (e.g. continuing at the end of the study), and assessment must be made at each visit (or more frequently, if necessary) of any changes in severity, the suspected relationship to the interventions required to treat it, and the outcome.

Information about adverse drug reactions for the investigational drug can be found in the Investigator's Brochure (IB).

Abnormal laboratory values or test results constitute adverse events only if they fulfill at least one of the following criteria:

- they induce clinical signs or symptoms
- they are considered clinically significant
- they require therapy

Clinically significant abnormal laboratory values or test results must be identified through a review of values outside of normal ranges/clinically notable ranges, significant changes from baseline or the previous visit, or values which are considered to be non-typical in subjects with the underlying disease.

Progression of GEP-NET (including fatal outcomes), if documented by use of appropriate method, should not be reported as a serious adverse event, except if the investigator considers that progression of malignancy is related to study treatment.

If patients in the study have been infected and/or tested for SARS-CoV-2 (COVID-19), sites should capture the patient-relevant details in the patient charts/source data, i.e. start and end dates of suspected infection when information is available, any date of test(s) performed, test name/method together with the result (positive/ negative), treatment interruptions or discontinuations for each of the impacted patients enrolled in the trial.

If an infection is suspected based on symptoms suggestive of COVID-19, sites are requested to enter an AE with AE term as 'Suspected COVID-19' or as 'Confirmed COVID-19' if confirmed by testing (e.g. antigen/radiologic)

• For Suspected cases, if a test is performed and confirms COVID-19, sites are requested to update the Suspected COVID-19 AE record with AE term as 'Confirmed COVID-19', however, not altering the start date.

• For Suspected cases, if a test is performed and does not confirm COVID-19, sites are requested to follow the normal Adverse Event reporting process and record the AE term as the diagnosis, or if no diagnosis, the symptoms. If COVID-19 is still suspected (e.g. false negative suspected), the AE term can remain as 'Suspected COVID-19' per the Investigator's judgement.

In normal circumstances, "testing" for COVID-19 would not be reported as an Adverse Event, however, in order to adequately capture the impact of COVID-19 on trial patients, these will be required to understand the patients' health status and sequence of events for tests performed to diagnose symptoms of COVID-19.
- If a test was/tests were performed; sites are requested to enter additional AE(s) with appropriate AE term for the type of test with the result, such as 'SARS-CoV-2 test negative', 'SARS-CoV-2 test positive', etc.
- Start Date and End Date should be entered as the date of the test

- All other fields on Adverse Event CRFs should remain blank. Any resulting queries will be closed by Data Management
- Tests performed after a patient has died should not be reported as Adverse Events

The infection is considered serious and to be reported as Serious Adverse Event (SAE) only when it fulfills the protocol definition of a Serious Adverse Events (see Section 10.1.2 and Section 10.1.6).

10.1.2 Serious adverse events

An SAE is defined as any adverse event [appearance of (or worsening of any pre-existing)] undesirable sign(s), symptom(s) or medical conditions(s)) which meets any one of the following criteria:

- fatal
- life-threatening
- Life-threatening in the context of a SAE refers to a reaction in which the subject was at risk of death at the time of the reaction; it does not refer to a reaction that hypothetically might have caused death if it were more severe (please refer to the ICH-E2D Guidelines).
- results in persistent or significant disability/incapacity
- constitutes a congenital anomaly/birth defect
- requires inpatient hospitalization or prolongation of existing hospitalization, unless hospitalization is for:
  - Routine treatment or monitoring of the studied indication not associated with any deterioration in condition.
  - Elective or pre-planned treatment for a pre-existing condition that is unrelated to the indication under study and has not worsened since signing the information consent
  - social reasons and respite care in the absence of any deterioration in the subject's general condition
  - treatment on an emergency outpatient basis for an event not fulfilling any of the definitions of a SAE given above and not resulting in hospital admission
- medically significant, e.g. defined as an event that jeopardizes the subject or may require medical or surgical intervention to prevent one of the outcomes listed above.

Medical and scientific judgment should be exercised in deciding whether other situations should be considered serious reactions, such as important medical events that might not be immediately life threatening or result in death or hospitalization but might jeopardize the subject or might require intervention to prevent one of the other outcomes listed above. Such events should be considered as "medically significant". Examples of such events are intensive treatment in an emergency room or at home for allergic bronchospasm, blood dyscrasias or convulsions that do

not result in hospitalization or development of dependency or abuse (please refer to the ICH-E2D Guidelines).

All malignant neoplasms will be assessed as serious under “medically significant” if other seriousness criteria are not met and the malignant neoplasm is not a disease progression of the study indication.

Any suspected transmission via a medicinal product of an infectious agent is also considered a serious adverse reaction.

All reports of intentional misuse and abuse of the product are also considered serious adverse event irrespective if a clinical event has occurred.

COVID-19 infections are considered serious and have to be reported as Serious Adverse Event (SAE) only when it fulfills the protocol definition of a Serious Adverse Events. All SAE data should be entered as per the study level CRF completion guidelines e.g. report outcome as ‘fatal’ if the patient died due to COVID-19.
In case a patient died due to COVID-19, it must be clear from the eCRF if the death was due to underlying COVID-19 or a different reason. Therefore, ‘Primary Cause/ Reason for Death’ should indicate ‘Suspected COVID-19’ or ‘Confirmed COVID-19’. If a test is performed after the patient died and the assessment indicates death was due to COVID-19 infection, the ‘Primary Cause/ Reason for Death’ should be retrospectively changed from ‘Suspected COVID-19’ to ‘Confirmed COVID-19’.

10.1.3 Adverse Drug Reaction (ADR)

An ADR is any noxious and unintended response to an IMP related to any dose with at least a reasonably possible causal relationship with the IMP. Briefly, an ADR is an AE which is suspected to be possibly related to IMP by either the investigator or the study sponsor.

10.1.4 Suspected Unexpected Serious Adverse Reactions (SUSARs)

An ADR will be assessed to be “unexpected″ if the nature, severity or frequency of the event is not consistent with the applicable product information available for the IMP. An ADR will be assessed to be “expected″ if it is listed in the Investigator’s Brochure.

A SUSAR is an adverse event regarded as serious with at least possible causal relationship to the drug, the nature, severity or frequency of which is not consistent with the applicable information available in the reference documents available for the IMP.

In the case of octreotide, “expectedness” will be assessed based on octreotide Reference Safety Information (RSI) reported in the prescribing information.

10.1.5 Adverse Events of Special Interest

AESI are defined as events (serious or non-serious) which are ones of scientific and medical concern specific to the Sponsor’s product or program, for which ongoing monitoring and rapid

communication by the Investigator to the Sponsor is appropriate. Such events require further investigation in order to characterize and understand them.

AESI are defined based on the NETTER-1 study established AESI list as well as on the basis of an ongoing review of the safety data collected during Lutathera clinical programs and post marketing.

These potential risks deserve special attention even if they do not fulfill any of the seriousness criteria. AESI occurring in patients enrolled in both arms should be reported to the Sponsor for safety analysis following the reporting procedure and timelines for SAEs. During the treatment phase, all AESI need to be reported to the Sponsor irrespective of causality. During the follow-up phase, AESI need to be reported only if considered related to the study treatment except for all secondary hematological malignancies which need to be reported as AESI irrespective of causality.

The following AESI categories representing main risks of Lutathera and amino acid treatment are listed in Table 10-1.

Table 10-1. Adverse events of special interest

**AESI categories**
Hematotoxicity
Secondary hematological malignancies
Nephrotoxicityg
Cardiovascular and electrolyte disorders

Hematotoxicity: The main critical organ of Lutathera treatment is the bone marrow. Significant hematotoxicity, defined as Grade 2 or higher thrombocytopenia, or Grade 3 or 4 of anaemia, leuko-/neutropenia are considered dose-modifying toxicities in the study and must be reported as AESI when not strictly fulfilling the criteria of serious adverse events. Haematological toxicities regardless of severity must be reported as AESI if accompanied by clinical consequences, i.e., infections in the presence of leuko-/neutro-/lymphopenia, hemorrhages / purpuric lesions under thrombocytopenia that is not explained by another coagulation disorder, dyspnea / fatigue in the presence of anaemia not otherwise explained by the underlying carcinoid syndrome or other co-morbidity.

Secondary haematological malignancies: any secondary hematological malignancies, irrespective of causality, including MDS and acute myeloid leukemia, should be reported in every case, as AESI/SAE.

Nephrotoxicity: Since Lutathera is cleared through the kidneys and reabsorbed by the kidneys, the kidneys have always been considered the "critical organs". An infusion of sterile amino acid is used for kidneys protection by inhibition of tubular reabsorption of Lutathera. Pursuant to these risk minimization efforts and in addition to the criteria of dose-modifying toxicities and criteria of inclusion (at baseline and before subsequent treatments) pertinent to renal function measurements, renal and urinary tract toxicities are considered AESI. Investigators must report as AESI renal toxicities, including renal failure (ranging from significantly reduced measured or estimated creatinine clearance to clinically overt renal failure other than that of obvious non-

IMP-induced origin), suspected radiation nephropathy of any type, such as radiation-induced thrombotic microangiopathy (manifested with, e.g., proteinuria, hypertension, edema, anaemia, decrease serum haptoglobin), or general symptoms and signs of acute radiation toxicity (e.g., increased frequency and urgency of urination, nocturia, dysuria, bladder spasm, bladder obstruction, genitourinary ulceration or necrosis).

Cardiovascular and electrolyte disorders: All clinically significant changes in blood pressure, heart rate, and electrocardiogram parameters must be reported as AESI if they occur within reasonable propinquity of Lutathera and/or amino acid administration in the judgment of the Investigator. Likewise, clinically manifest and/or consequences of hypo-/hypertension, arrhythmias, cardiac conduction disturbances, and other cardiac pathologies evidenced by objective findings / changes on electrocardiogram or echocardiography should also be considered for AESI reporting. In addition, any clinically significant event of hyperkalemia or hypokalemia must be reported as AESI.

10.1.6 SAE reporting

To ensure subject safety, every SAE, regardless of causality, occurring after the subject has provided informed consent and until 30 days after the patient has stopped study treatment must be reported to AAA pharmacovigilance immediately, without undue delay and under no circumstances later than 24 hours following knowledge of its occurrence. Detailed instructions regarding the submission process and requirements are to be found in the investigator folder provided to each site.

All follow-up information for the SAE including information on complications, progression of the initial SAE and recurrent episodes must be reported as follow-up to the original episode immediately, without undue delay and under no circumstances later than within 24 hours of the investigator receiving the follow-up information. An SAE occurring at a different time interval or otherwise considered completely unrelated to a previously reported one must be reported separately as a new event.

Information about all AE/SAEs is collected and recorded on the SAE Report Form; all applicable sections of the form must be completed in order to provide a clinically thorough report. The investigator must assess and record the relationship of each AE/SAE to each specific study treatment (if there is more than one study treatment), complete the SAE Report Form in English, and submit the completed form under no circumstances later than 24 hours following knowledge to AAA pharmacovigilance.

Follow-up information is submitted in the same way as the original AE/SAE Report. Each reoccurrence, complication, or progression of the original event should be reported as a follow-up to that event regardless of when it occurs. The follow-up information should describe whether the event has resolved or continues, if and how it was treated, and whether the patient continued or withdrew from study participation.

If the SAE is not previously documented in the Investigator's Brochure or Package Insert (new occurrence) and is thought to be related to the study treatment, AAA pharmacovigilance associate may urgently require further information from the investigator for health authority

reporting. AAA may need to issue an Investigator Notification (IN) to inform all investigators involved in any study with the same study treatment that this SAE has been reported.

Suspected Unexpected Serious Adverse Reactions (SUSARs) will be collected and reported to the competent authorities and relevant ethics committees in accordance with EU Guidance 2011/C 172/01 or as per national regulatory requirements in participating countries.

Any SAEs experienced after the 30 day period should only be reported to AAA Safety if the investigator suspects a causal relationship to study treatment, except for secondary hematological malignancies which need to be reported irrespective of causality.

10.1.7 Pregnancy reporting

To ensure subject safety, each pregnancy occurring after signing the informed consent form must be reported to Advanced Accelerator Applications within 24 hours of learning of its occurrence. The pregnancy should be followed up to determine outcome, including spontaneous or voluntary termination, details of the birth, and the presence or absence of any birth defects, congenital abnormalities, or maternal and/or newborn complications.

Pregnancy should be recorded and reported by the investigator to the AAA Pharmacovigilance. Pregnancy follow-up should be recorded on the same form and should include an assessment of the possible relationship to the study treatment and any pregnancy outcome. Any SAE experienced during pregnancy must be reported.

10.1.8 Reporting of study treatment errors including misuse/abuse

Not applicable.

10.1.9 Annual Safety Report

For more information on AE and SAE definition and reporting requirements, please see the respective sections.

Once per year, the sponsor or PI will supply a report on the safety of trial patients with all available relevant information concerning patient safety during the reference period to the Competent Authorities of all Countries where the trial is being conducted. This report will also be supplied to the responsible ethics committee.

The annual safety report will be compiled according to the corresponding ICH guideline E2F

“Development Safety Update Report – DSUR”.

The data lock point for the patient data to be included and analyzed should be the last day of the one-year reporting period calculated from *The Development International Birth Date* (DIBD).

The sponsor will submit the report within 60 calendar days after the reference date (data-lock point).

10.2 Additional Safety Monitoring

Lutathera safety profile is well documented during Erasmus and NETTER-1 clinical trials as well as during post marketing surveillance, therefore additional data monitoring will not be used for this study. Safety data will be reviewed on regular basis by both clinical and pharmacovigilance departments.

10.2.1 Steering Committee

The steering committee will be established comprising investigators participating in the trial and Advanced Accelerator Applications representatives from the Clinical Trial Team.

The SC will ensure transparent management of the study according to the protocol through recommending and approving modifications as circumstances require. The SC will review protocol amendments as appropriate. Together with the clinical trial team, the SC will also develop recommendations for publications of study results including authorship rules. The details of the membership and role of the Steering Committee will be defined in a Steering Committee charter.

11 Data Collection and Database management

11.1 Data collection

The designated investigator staff will enter the data required by the protocol into the Electronic Case Report Forms (eCRF). The eCRFs have been built using fully validated secure web enabled software that conforms to 21 CFR Part 11 requirements. Investigator site staff will not be given access to the EDC system until they have been trained. Automatic validation programs check for data discrepancies in the eCRFs, allow modification and/or verification of the entered data by the investigator staff.

The Principal investigator is responsible for assuring that the data (recorded on eCRFs) is complete, accurate and that entry and updates are performed in a timely manner.

The Investigator must certify that the data entered are complete and accurate. After final database lock, the investigator will receive copies of the subject data for archiving at the investigational site.

All data should be recorded, handled and stored in a way that allows its accurate reporting, interpretation and verification.

11.2 Database management and quality control

Advanced Accelerator Applications personnel (or designated CRO) will review the data entered by investigational staff for completeness and accuracy. Electronic data queries stating the nature of the problem and requesting clarification will be created for discrepancies and missing values and sent to the investigational site via the EDC system. Designated investigator site staff is required to respond promptly to queries and to make any necessary changes to the data.

Concomitant treatments and prior medications entered into the database will be coded using the WHO Drug Reference List, which employs the Anatomical Therapeutic Chemical classification system. Medical history/current medical conditions and adverse events will be coded using the Medical dictionary for regulatory activities (MedDRA) terminology.

Data about all study treatment (s) dispensed to the subject and all dosage changes will be tracked on the study drug accountability logs. An electronic system for the Drug Supply Management might be implemented for this trial. The local monitor will provide training material to order the study drugs during the initiation visit. Once all the necessary actions have been completed and the database has been declared to be complete and accurate, it will be locked. Any changes to the database after that time can only be made after written agreement by Advanced Accelerator Applications development management.

After database lock, the investigator will receive a CD-ROM or paper copies of the subject data for archiving at the investigational site.

11.3 Site monitoring

Before study initiation, at a site initiation visit or at an investigator's meeting, an Advanced Accelerator Applications representative will review the protocol and data capture requirements (eCRFs) with the investigators and their staff. During the study, Advanced Accelerator

Applications employs several methods of ensuring protocol and GCP compliance and the quality/integrity of the sites' data. The field monitor will visit the site to check the completeness of subject records, the accuracy of data capture / data entry, the adherence to the protocol and to Good Clinical Practice, the progress of enrollment, and to ensure that study treatment is being stored, dispensed, and accounted for according to specifications. Key study personnel must be available to assist the field monitor during these visits. Continuous remote monitoring of each site's data may be performed by Advanced Accelerator Applications. Additionally, a central analytics organization may analyze data & identify risks & trends for site operational parameters, and provide reports to Advanced Accelerator Applications clinical teams to assist with trial oversight.

The investigator must maintain source documents for each subject in the study, consisting of case and visit notes (hospital or clinic medical records) containing demographic and medical information, laboratory data, electrocardiograms, and the results of any other tests or assessments. All information on eCRFs must be traceable to these source documents in the Subject's file. The investigator must also keep the original informed consent form signed by the subject (a signed copy is given to the subject).

The investigator must give the monitor access to all relevant source documents to confirm their consistency with the data capture and/or data entry. Advanced Accelerator Applications monitoring standards require full verification for the presence of informed consent, adherence to the inclusion/exclusion criteria, documentation of SAEs, and of data that will be used for all primary variables. Additional checks of the consistency of the source data with the eCRFs are performed according to the study-specific monitoring plan. No information in source documents about the identity of the subjects will be disclosed.

12 Data analysis and statistical methods

The primary efficacy and safety analyses will be performed after observing approximately 99 PFS events per central assessment. The primary CSR will be produced after the primary PFS analysis. The key secondary endpoints will be tested hierarchically at the time of the primary PFS analysis. Any additional data for participants continuing to receive study treatment past this time and for participants continuing for efficacy follow-up (PFS, OS) and quality of life follow-up, as allowed by the protocol, will be further summarized at the time of the final analysis. A final report to support the final analysis will be planned after End of Study (EOS).

Advanced Accelerator Applications and/or a designated contract research organization (CRO) will perform all analysis.

Any data analysis carried out independently by the investigator should be submitted to Advanced Accelerator Applications before publication or presentation.

12.1 Analysis sets

The Full Analysis Set (FAS) comprises all subjects to whom study treatment has been assigned by randomization. According to the intent to treat principle, subjects will be analyzed according to the treatment and strata they have been assigned to during the randomization procedure.

The Safety Set includes all subjects who received at least one dose of study treatment. Patients will be analyzed according to the study treatment received, where treatment received is defined as the randomized treatment if the subject took at least one dose of that treatment or the first treatment received if the randomized/assigned treatment was never received.

The pharmacokinetic analysis set (PAS) consists of all randomized patients who received at least one dose of study drug and had at least one post dosing evaluable PK assessment.

The cross-over set is comprised of all patients randomized to the Sandostatin LAR Depot (octreotide long-acting) arm who received at least one dose of Lutathera after cross-over following confirmed disease progression per central, blinded, real-time images reading in the randomized period.

The re-treatment set is comprised of all patients randomized to the Lutathera arm who received at least one dose of Lutathera during re-treatment period after confirmed disease progression per central, blinded, real-time images reading in the randomized period.

12.2 Subject demographics and other baseline characteristics

Demographic and other baseline data including disease characteristics will be listed and summarized descriptively by treatment group for the FAS and Safety set.

Categorical data will be presented as frequencies and percentages. For continuous data, mean, standard deviation, median, minimum, and maximum will be presented. For selected parameters, 25th and 75th percentiles will also be presented.

Relevant medical history and current medical conditions at baseline will be summarized separately by system organ class and preferred term, by treatment group.

12.3 Treatments

The Safety set will be used for the analyses below. Categorical data will be summarized as frequencies and percentages. For continuous data, mean, standard deviation, median, 25th and 75th percentiles, minimum, and maximum will be presented.

The duration of exposure in months to Lutathera plus Standard Dose octreotide long-acting (30 mg), and, High Dose octreotide long-acting (60 mg) as well as the dose intensity (computed as the ratio of actual cumulative dose received and actual duration of exposure) and the relative dose intensity (computed as the ratio of dose intensity and planned dose intensity) will be summarized by means of descriptive statistics using the safety set.

Concomitant medications and significant non-drug therapies prior to and after the start of the study treatment will be listed and summarized according to the Anatomical Therapeutic Chemical (ATC) classification system, by treatment group.

The number of subjects with dose adjustments (reductions, interruption, or permanent discontinuation) and the reasons will be summarized by treatment group and all dosing data will be listed.

12.4 Analysis of the primary endpoint(s)

The primary aim of the study is to demonstrate a prolongation of Progression Free Survival (PFS) time (centrally assessed according to RECIST 1.1).

12.4.1 Definition of primary endpoint(s)

The primary endpoint of the study is progression-free survival (PFS), defined as the time from the date of randomization to the date of the first documented progression or death due to any cause. PFS will be assessed via central review according to RECIST 1.1. Censoring conventions are provided in Section 12.4.3.

12.4.2 Statistical model, hypothesis, and method of analysis

Assuming proportional hazards model for PFS, the null hypothesis will be tested at one-sided 2.5% level of significance:

H_{01} (null hypotheses): $\Theta_1 \geq 0$ vs. H_{a1} (alternative hypotheses): $\Theta_1 < 0$

Where Θ_1 is the log hazard ratio of PFS in the Lutathera plus Standard Dose octreotide long-acting (30 mg) (investigational) arm vs. High Dose octreotide long-acting (60 mg) (control) arm.

The primary efficacy analysis to test this hypothesis and compare the two treatment groups will consist of a stratified log-rank test at an overall one-sided 2.5% level of significance in favor of the Lutathera plus Standard Dose octreotide long-acting (30 mg) arm. The stratification will be based on following randomization stratification factors (grade: G2 vs. G3; and tumor origin: pNET vs other origin).

Analyses will be based on the FAS population according to the randomized treatment group and strata assigned at randomization. The PFS distribution will be estimated using the Kaplan-Meier method, and Kaplan-Meier curves, median and associated 95% confidence intervals will be

presented for each treatment group. The hazard ratio for PFS will be calculated, along with its 95% confidence interval, from a stratified Cox model using the same stratification factors as for the log-rank test.

In case of few events in some stratum, the primary analysis will instead be unstratified (the precise handling of strata will be described in the SAP). Both stratified and unstratified analyses will be presented.

12.4.3 Handling of missing values/censoring/discontinuations

In cases of incomplete data to determine the exact time of tumor progression or death, the principle for imputation is to be conservative. The following rules can be used to determine the event/censoring date as well as the status of event or censoring at the time of the analysis of the primary end-point PFS depending on data pattern:

In the primary analysis, PFS will be censored at the date of the last adequate tumor assessment if no PFS event is observed prior to the analysis cut-off date. The censoring rules will be detailed in the SAP.

For sensitivity analyses, see next Section 12.4.4.

12.4.4 Sensitivity and Supportive analyses

Sensitivity analyses

A sensitivity analysis will assess PFS events if a patient has disease progression or death regardless of whether the patient had missing scheduled visits, treatment discontinuation for toxicity, or new anticancer treatment started without progression.

As sensitivity analyses performed in the FAS, the hazard ratio and 95% confidence interval for PFS per blinded independent central review will be obtained from an unstratified and covariate unadjusted Cox model, except when the primary analysis is unstratified, see Section 12.4.2.

Further sensitivity analyses will be specified in the SAP.

Supportive analyses

As a supportive analysis, PFS as per local assessment will be analyzed using a stratified Cox model, with the same analysis conventions as the primary efficacy analysis, and the treatment effect will be summarized by the hazard ratio with its 95% confidence interval. Kaplan-Meier curves, medians and 95% confidence intervals of the medians will be presented for each treatment group.

As supportive analyses performed in the FAS, the hazard ratio and 95% confidence interval for PFS per blinded independent central review will be obtained from:

- A stratified and covariate adjusted Cox model including as covariates the following: ALP, Time since Diagnosis, Time since Metastasis. The final list of covariates to be included in the model will be provided in the SAP.

- Further supportive analyses will be specified in the SAP

These analyses will include Kaplan-Meier medians with their 95% confidence intervals, and hazard ratios and their 95% confidence intervals from stratified Cox models.

If the primary analysis is statistically significant, subgroup analyses to assess the homogeneity of the treatment effect across demographic and baseline disease characteristics will be performed. The subgroups will include the stratification factors (Grade and Site), Age, Gender, Race, Karnofsky score, Tumor burden at baseline, BMI. The subgroups will be further detailed in the SAP.

The number of subjects censored and reasons for censoring will be summarized by treatment group using descriptive statistics, presented separately for local review and blinded independent central review.

12.5 Analysis of secondary endpoints

The key secondary objectives in this study are to compare the two treatment groups with respect to:

- ORR: rate of patients with best overall response of partial response (PR) or complete response (CR) (centrally assessed according to RECIST 1.1).
- Time to deterioration defined as the first deterioration of 10 points (TTD) defined for the EORTC QLQ-C30 and EORTC QLQ-G.I.NET21 global health scale, diarrhea, fatigue, and pain.

Other secondary objectives of the study are to compare the two treatment groups with respect to:

- DOR: Duration of Response defined as time from complete or partial response to progression or death due to underlying cancer only.
- Rate of adverse events and laboratory toxicities (scored according to CTCAE grade).
- OS: time from the randomization date until the day of death due to any cause.

12.5.1 Key secondary objectives

If the primary endpoint is significant, the key secondary endpoints of overall response rate (ORR) and QoL will be tested in a hierarchical fashion to protect the type I error rate. The order of the hypothesis testing shall be ORR followed by QoL Global Health Scale (TTD, see below), followed by QoL Diarrhea (TTD), (TTD)-fatigue, and, (TTD)-pain (TTD). The key secondary objectives will be tested at the time of the primary analysis. At the final analysis, only a descriptive analysis will be performed.

Overall response rate (ORR) is defined as the proportion of subjects with best overall response (BOR) of complete response (CR) or partial response (PR), as per central review and according to RECIST 1.1.

ORR will be calculated based on the FAS and according to the ITT principle. ORR and its 95% confidence interval will be presented by treatment group. The Cochran-Mantel-Haenszel chi-square test, stratified by the randomization stratification factors, will be used to compare ORR

between the two treatment groups, at the 1-sided 2.5% level of significance. As a supportive analysis, ORR as per local review will be presented by treatment group, along with 95% confidence intervals.

Time to deterioration (TTD) is defined as time from randomization to the first deterioration of 10 points in domain score compared to the baseline score for the EORTC QLQ-C30 global health scale, diarrhea, fatigue, and pain assessments. The TTD distribution will be estimated using the Kaplan-Meier method, and the Kaplan-Meier curves, medians and 95% confidence intervals of the medians will be presented for each treatment group. Treatment effect will be tested by the stratified log-rank test. The hazard ratio for TTD will be calculated, along with its 95% confidence interval, using a stratified Cox model using stratified the randomization stratification factors. Patients with no baseline and/or no follow-up are censored at randomization. Diarrhea, fatigue and pain are considered clinically relevant (Strosberg, et al., 2018), identified as top symptoms in a patient survey (Singh, et al., 2018) and TTD associated with these symptoms domains was significant in a post-hoc analysis of NETTER-1.

Further details regarding the planned sensitivity and supplementary analyses for the key secondary endpoints will be provided in the SAP.

12.5.2 Other efficacy endpoints

Disease Control Rate (DCR) is defined as rate of patients with best overall response of partial response (PR), complete response (CR), or stable disease (SD) (centrally assessed according to RECIST 1.1). More precisely, it counts the presence of at least one confirmed CR or confirmed PR or SD. The DCR will be calculated based on FAS according to the ITT principle. DCR and its 95% confidence interval will be presented by treatment group. The Cochrane-Mantel-Haenszel chi-square test, stratified by the randomization stratification factors, will be used to compare DCR between the two treatment groups, at the one-sided 2.5% level of significance. The p-value to be generated using the Cochrane-Mantel-Haenszel chi-square test is considered nominal, and not to be considered confirmatory as it is not part of the testing strategy. The SAP will advise on how to proceed in case of few events in any stratum.

Duration of response (DOR) only applies to subjects whose best overall response is complete response (CR) or partial response (PR) according to RECIST 1.1 based on tumor response data per local review. The start date is the date of first documented response of CR or PR (i.e., the start date of response, not the date when response was confirmed), and the end date is defined as the date of the first documented progression per local review or death due to underlying cancer. Subjects continuing without progression or death due to underlying cancer will be censored at the date of their last adequate tumor assessment. DOR will be listed and summarized by treatment group for all subjects in the FAS with confirmed BOR of CR or PR.

OS is defined as the time from date of randomization to date of death due to any cause. If a subject is not known to have died, then OS will be censored at the latest date the subject was known to be alive (on or before the cut-off date).

OS will be analyzed in the FAS population according to the randomized treatment group and strata assigned at randomization. At the time of the PFS primary analysis, the OS distribution will be estimated using the Kaplan-Meier method, and the Kaplan-Meier curves, medians and

95% confidence intervals of the medians will be presented for each treatment group. The hazard ratio for OS will be calculated, along with its 95% confidence interval, using a stratified Cox model.

At the final analysis time, a supportive analysis will adjust for cross-over from Control to Lutathera. This will be achieved by using a ***rank-preserving structural failure time method (RPSFT)*** to correct for confounding introduced by the change of treatment. The use of the RPSFT method allows survival time estimates gained by anyone receiving Lutathera (i.e. either as randomized to Lutathera or after cross-over from Control to Lutathera). The RPSFT model is based on an accelerated failure time model) and uses a structural assumption of time-proportionality instead of a proportional hazards assumption as used in the Cox model. The widely used Cox model measures drug effect on the hazard ratio scale, whereas the accelerated failure time model measures drug effect on the survival time ratio scale.

Additionally, a supportive analysis of OS based on the ***Inverse Probability Censoring Weight*** method will be performed at the final analysis time. Further details regarding theses analyses will be given in the SAP.

12.5.3 Safety endpoints

For all safety analyses, the safety set will be used. All listings and tables will be presented by treatment group.

The frequency of adverse events and laboratory toxicities (scored according to CTCAE 5.0 grade or current version) will be presented.

All adverse events (AEs), whether or not spontaneously reported by the patient, will be recorded starting from the signing of the ICF until the end of the Treatment Phase.

Safety summaries (tables, figures) include only data from the on-treatment period with the exception of baseline data which will also be summarized where appropriate (e.g. change from baseline summaries). In addition, a separate summary for death including on treatment and post treatment deaths will be provided. In particular, summary tables for adverse events (AEs) will summarize only on-treatment events, with a start date during the on-treatment period (*treatment-emergent* AEs).

The on-treatment period lasts from the date of first administration of study treatment to 30 days after the date of the last actual administration of any study treatment.

The overall observation period will be divided into three mutually exclusive segments:

- pre-treatment period: from day of subject's informed consent to the day before first dose of study medication
- on-treatment period: from day of first dose of study medication to 30 days after last dose of study medication
- post-treatment period: starting at day 30+1 after last dose of study medication.
- There will be a dedicated presentation of safety for patients who cross-over.

Adverse events

All information obtained on adverse events will be displayed by treatment group and subject.

The number (and percentage) of subjects with treatment emergent adverse events (events started after the first dose of study medication or events present prior to start of double-blind treatment but increased in severity based on preferred term) will be summarized in the following ways:

1. by treatment, primary system organ class and preferred term.
2. by treatment, primary system organ class, preferred term and maximum severity.
3. by treatment, Standardized MedDRA Query (SMQ) and preferred term

Separate summaries will be provided for study medication related adverse events, death, serious adverse events, other significant adverse events leading to discontinuation and adverse events leading to dose adjustment.

The number (and proportion) of subjects with AESI/related to identified and potential risks (see Section 10.1) will be summarized by treatment.

A subject with multiple adverse events within a primary system organ class is only counted once towards the total of the primary system organ class.

Vital signs

All vital signs data will be listed by treatment group, subject, and visit/time and if ranges are available, abnormalities (and relevant orthostatic changes) will be flagged. Summary statistics will be provided by treatment and visit/time.

12-lead ECG

PR, QRS, QT, QTcF, and RR intervals will be obtained from 12-lead ECGs for each subject during the study. ECG data will be read and interpreted (centrally/locally).

All ECG data will be listed by treatment group, subject and visit/time, abnormalities will be flagged. Summary statistics will be provided by treatment and visit/time.

Clinical laboratory evaluations

All laboratory data will be listed by treatment group, subject, and visit/time and if normal ranges are available abnormalities will be flagged. Summary statistics will be provided by treatment and visit/time. Shift tables using the low/normal/high/ (low and high) classification will be used to compare baseline to the worst on-treatment value.

Grading of laboratory values will be assigned programmatically as per NCI Common Terminology Criteria for Adverse Events (CTCAE) version 5.0 or current version. The calculation of CTCAE grades will be based on the observed laboratory values only, clinical assessments will not be taken into account.

CTCAE Grade 0 will be assigned for all non-missing values not graded as 1 or higher. Grade 5 will not be used.

For laboratory tests where grades are not defined by CTCAE, results will be categorized as low/normal/high based on laboratory normal ranges.

The following summaries will be generated separately for hematology, and biochemistry tests:

1. Listing of all laboratory data with values flagged to show the corresponding CTCAE grades if applicable and the classifications relative to the laboratory normal ranges
2. For laboratory tests where grades are defined by CTCAE,
3. Worst post-baseline CTCAE grade (regardless of the baseline status). Each subject will be counted only once for the worst grade observed post-baseline.
4. Shift tables using CTCAE grades to compare baseline to the worst on-treatment value
5. For laboratory tests where grades are not defined by CTCAE,
6. Shift tables using the low/normal/high/ (low and high) classification to compare baseline to the worst on-treatment value.

CgA assessment is discontinued in Q3 2022 in South Korea due to lab kits shortage (assessments performed before discontinuation will be included in the analyses).

12.6 Analysis of exploratory endpoints

12.6.1 PFS2

PFS2 is defined as time from date of randomization to the first documented local progression on next-line therapy or death from any cause, whichever occurs first. The first documented progression on next-line treatment is based on investigator assessment of PD (i.e. as captured on the anti-neoplastic therapy after treatment discontinuation eCRF page); it is not necessary to continue to collect tumor assessments data for subsequent anti-neoplastic therapies for the purpose of PFS2.

- Next-line therapy is defined as the first new (systemic) anti-neoplastic therapy initiated after discontinuation of study treatment regardless of EoT reason. Drugs given as part of the same regimen should be considered as first line (i.e. part of the next-line therapy).
- New anti-neoplastic therapies after EoT will be collected in the anti-neoplastic therapy after treatment discontinuation eCRF page including start/end date, reason for discontinuation, date and type of progression («clinical» vs «radiologic»).
- PFS2 will be censored if no PFS2 event (progression or death) is observed during next-line therapy before the analysis cut-off date; the censoring date will be the last contact date.
- However, in case a second new anti-neoplastic therapy is introduced without prior PFS2 event, then PFS2 will be censored at the end date of the first new anti-neoplastic therapy (i.e. next line therapy).
- Any death prior to initiation of next-line therapy will be considered as an event for PFS2. Any death occurring following end of next line therapy will be considered as an event if no second new anti-neoplastic therapy has been introduced.

- PFS and PFS2 may be identical if a subject did not experience an event (i.e. progression) prior to initiation of next-line therapy, and adequate tumor assessments continue until RECIST 1.1-documented disease progression after initiation of next-line therapy.

PFS2 will be analyzed in the FAS population according to the randomized treatment group and strata assigned at randomization. The PFS2 distribution will be estimated using the Kaplan-Meier method, and the Kaplan-Meier curves, medians and 95% confidence intervals of the medians will be presented for each treatment group. The hazard ratio for PFS2 will be calculated, along with its 95% confidence interval, using a stratified Cox model.

Time to Second Progression (PFS2) in the two treatment arms will be evaluated as a secondary endpoint. This endpoint is defined as the time in months from the first progression to the second progression or death. The date of both the first and second progression will be derived programmatically through the RECIST criteria.

It is recognized that due to cross-over, the lower scan frequency (every six months) after PEP, and, the relatively short follow-up, the possibility to draw firm conclusions is limited.

12.6.2 Patient reported outcomes not associated with key secondary endpoint

TTD for items/scales derived from EORTC QLQ-G.I.NET21 and EORTC QLQ-30 not included among the key secondary endpoints will be analyzed as exploratory endpoints. The TTD distribution will be estimated using the Kaplan-Meier method, and the Kaplan-Meier curves, medians and 95% confidence intervals of the medians will be presented for each treatment group. The hazard ratio for TTD will be calculated, along with its 95% confidence interval, using a stratified Cox model.

Quality of Life will also be assessed in terms of change from baseline in EORTC QLQ-G.I.NET21, EORTC QLQ-C30 and EQ-5D-5L through Mixed Model Repeated Measures (MMRM), which deals with missing values in a model based way, and implicitly imputes missing data under a missing at random assumption. The model will include the explanatory variables Treatment, Baseline, Week, and, the interaction between Treatment and Week. A test of treatment effect is performed at each week, and, overall.

12.6.3 Pharmacokinetics

Patients in the control arm (all countries except for China) will yield PK data from blood sampling performed at pre-dose with respect to the octreotide long-acting i.m. injection for the 2nd, 4th, 5th and 7th treatment cycle (that is respectively at week 4, 12, 16 and 24) for the determination of plasma trough levels. Data will be processed using a population based model, for exploratory purposes. More details on the presentation of results will be given in the SAP.

Additionally, descriptive statistics and graphical display of octreotide plasma concentrations during the course of treatment will presented by dose group. Exploratory analysis will be performed to assess the dose-exposure relationship. More details on the presentation of results will be given in the SAP.

12.6.4 Re-treatment with Lutathera

Safety will be presented in a similar manner to that in Section 12.5.3 for those participants who receive re-treatment with Lutathera during the re-treatment period. The re-treatment period lasts from the date of first administration of re-treatment with Lutathera to progression or death during the re-treatment period.

All information obtained on adverse events will be displayed by subject.

All vital signs data will be listed subject, and visit/time and if ranges are available, abnormalities (and relevant orthostatic changes) will be flagged. Summary statistics will be provided by visit/time.

All ECG data (as per local assessment) will be listed by subject and visit/time, abnormalities will be flagged. Summary statistics will be provided by visit/time.

All laboratory data will be listed by subject, and visit/time and if normal ranges are available abnormalities will be flagged. Summary statistics will be provided by visit/time. Shift tables using the low/normal/high/ (low and high) classification will be used to compare the latest assessment before re-treatment to the worst re-treatment value.

All efficacy analyses for ORR, DoR, PFS, PFS2, and OS will be descriptive. Definitions for ORR, DoR, and PFS (as per local assessment) are the same definitions in Section 12.5.2 except that the baseline will be based on the tumor assessment which demonstrated progression thus allowing re-treatment with Lutathera. Definitions for OS and PFS2 are the same to that in Section 12.5.2 and Section 12.6.1, respectively.

12.7 Interim analyses

An interim analysis with respect to OS will be performed at the time of the PFS final analysis. At this time point, only estimation of treatment effect will be performed (hazard ratio and its 95% confidence interval).

12.8 Sample size calculation

12.8.1 Primary endpoint(s)

The sample size calculation is based on the primary variable PFS. Assuming a median PFS in the control arm (High Dose octreotide long-acting (60 mg)) of approximately 15 months, it is hypothesized that treatment with Lutathera added to standard dose octreotide long-acting will result in a 50% reduction in the hazard rate (corresponding to an increase in median PFS from 15 months to 30 months).

To ensure 90% power to test the null hypothesis: PFS hazard ratio = 1, versus the specific alternative hypothesis: PFS hazard ratio = 0.50, it is calculated that a total of 99 PFS events need to be observed. This calculation assumes analysis by a one-sided log-rank test at the overall 2.5% level of significance, patients randomized to the two treatment groups in a 2:1 ratio. Assuming that enrolment will continue for approximately 22.2 months at a rate of 10 patients per month and a 15% dropout rate by the time of the final PFS analysis, a total of 222 patients (148 for Lutathera arm and 74 for the control arm) will need to be randomized to observe the targeted 99

PFS events at about 12.8 months after the randomization date of the last patient, i.e., 35 months after the randomization date of the first patient. If the final analysis is performed when the targeted 99 PFS events are observed, the observed hazard ratio will have to be < 0.658 which corresponds to a difference in median PFS of 7.8 months to declare statistical significance.

These calculations were made using the software package East 6.4.

Randomization will be stratified by Grade (G2 vs G3) and tumor origin (pNET vs other origin).

13 Ethical considerations and administrative procedures

13.1 Regulatory and ethical compliance

This clinical study was designed and shall be implemented, executed and reported in accordance with the ICH Harmonized Tripartite Guidelines for Good Clinical Practice, with applicable local regulations (including European Directive 2001/20/EC, US CFR 21), and with the ethical principles laid down in the Declaration of Helsinki.

13.2 Responsibilities of the investigator and IRB/IEC

Before initiating a trial, the investigator/institution must obtain approval/favorable opinion from the Institutional Review Board/Independent Ethics Committee (IRB/IEC) for the trial protocol, written informed consent form, consent form updates, subject recruitment procedures (e.g., advertisements) and any other written information to be provided to subjects. Prior to study start, the investigator is required to sign a protocol signature page confirming his/her agreement to conduct the study in accordance with these documents and all of the instructions and procedures found in this protocol and to give access to all relevant data and records to Advanced Accelerator Applications monitors, auditors, Advanced Accelerator Applications Quality Assurance representatives, designated agents of Advanced Accelerator Applications, IRBs/IECs, and regulatory authorities as required. If an inspection of the clinical site is requested by a regulatory authority, the investigator must inform Advanced Accelerator Applications immediately that this request has been made.

13.3 Publication of study protocol and results

The protocol will be registered in a publicly accessible database such as clinicaltrials.gov and as required in EudraCT. In addition, after study completion (defined as last patient last visit) and finalization of the study report the results of this trial will be submitted for publication and posted in a publicly accessible database of clinical trial results, such as the Advanced Accelerator Applications clinical trial results website and all required Health Authority websites (e.g. Clinicaltrials.gov, EudraCT etc.).

Advanced Accelerator Applications (AAA) follows the ICMJE authorship guidelines (www.icmje.org). Authors (including Sponsor associates who may qualify for authorship), must therefore satisfy all of the following ICMJE authorship criteria:

1. Substantial contributions to conception or design of the work; or the acquisition, analysis, or interpretation of data for the work; AND
2. Drafting the work or revising it critically for important intellectual content; AND
3. Final approval of the version to be published; AND
4. Agreement to be accountable for all aspects of the work in ensuring that questions related to the accuracy or integrity of any part of the work are appropriately investigated and resolved.

The key principles that will be followed for AAA-sponsored, research-related publications are:

- AAA supports the publication of study results for its innovative medicines in a timely manner, whatever their outcome. AAA policy is not to withhold, veto or suppress data. However, due consideration must be given to the rights of AAA to protect confidential and/or patentable information, and to the protection of personal information, in particular patient privacy.
- Review by AAA of draft publications by clinical investigators in advance of submission/presentation of publication is designed to:
  - Confirm the accuracy of the data
  - Verify that proprietary information is not being inadvertently disclosed
  - Secure intellectual property rights, as needed
  - Provide any relevant supplementary information
- Publication of partial data (unless planned in the protocol) is discouraged. As a matter of scientific rigor and fairness to all investigators involved in a clinical study, and in accordance with the Joint Position on the Publication of Clinical Trial Results in the Scientific Literature, issued by the International Federation of Pharmaceutical Manufacturers and Associations (IFPMA), European Federation of Pharmaceutical Industries and Associations (EFPIA), Japan Pharmaceutical Manufacturers Association (JPMA), and Pharmaceutical Research and Manufacturers of America (PhRMA), it is AAA policy for multicenter clinical studies that:
- The first publication in a journal, or a presentation at a congress, be based on consolidated data from all centers, analyzed as stipulated by the protocol and agreed upon by investigators before trial initiation.
- Multicenter trials are designed to take full account of data accumulated from all centers (sample sized, powered with appropriate error rates), and AAA discourages presenting or publishing data gathered from a single, or small group of centers, unless agreed to by study investigators (e.g., Study Steering Committee) and AAA. Center specific analyses have greater variability and lead to exaggerated observed-treatment effects that are inherently less reliable. Valid conclusions regarding the primary endpoint of a clinical trial can only be based on the analyses predefined by the protocol.
- Study results should be published according to the contracted protocol agreements.

13.4 Quality Control and Quality Assurance

Monitoring details describing strategy, including definition of study critical data items and processes (e.g. risk-based initiatives in operations and quality such as risk management and mitigation strategies and analytical risk-based monitoring), methods, responsibilities, and requirements, including handling of noncompliance issues and monitoring techniques (central, remote, or on-site monitoring) are provided in the monitoring plan.

Advanced Accelerator Applications maintains a robust Quality Management System (QMS) that includes all activities involved in quality assurance and quality control, to ensure compliance

with written Standard Operating Procedures as well as applicable global/local GCP regulations and ICH Guidelines.

Audits of investigator sites, vendors, and Advanced Accelerator Applications systems are performed by auditors, independent from those involved in conducting, monitoring or performing quality control of the clinical trial. The clinical audit process uses a knowledge/risk based approach.

Audits are conducted to assess GCP compliance with global and local regulatory requirements, protocols and internal SOPs, and are performed according to written Advanced Accelerator Applications processes.

Records and documents, including signed ICFs, pertaining to the conduct of this study must be retained by the investigator for at least 15 years after study completion unless local regulations or institutional policies require a longer retention period. No records may be destroyed during the retention period without the written approval of Sponsor. No records may be transferred to another location or party without written notification to Sponsor.

14 Protocol adherence

This protocol defines the study objectives, the study procedures and the data to be collected on study participants. Additional assessments required to ensure safety of subjects should be administered as deemed necessary on a case-by-case basis. Under no circumstances including incidental collection is an investigator allowed to collect additional data or conduct any additional procedures for any purpose involving any investigational drugs under the protocol, other than the purpose of the study. If despite this interdiction prohibition, data, information, observation would be incidentally collected, the investigator shall immediately disclose it to Advanced Accelerator Applications and not use it for any purpose other than the study, except for the appropriate monitoring on study participants.

Investigators ascertain they will apply due diligence to avoid protocol deviations. If an investigator feels a protocol deviation would improve the conduct of the study this must be considered a protocol amendment, and unless such an amendment is agreed upon by Advanced Accelerator Applications and approved by the IRB/IEC and health authorities, where required, it cannot be implemented.

Due to the ongoing COVID-19 pandemic during the conduction of this study, in addition to capturing protocol deviations, there is also a need to record details on the relationship of COVID-19 to protocol deviations occurring during the COVID-19 time period in a manner that will allow analysis and reporting. In order to capture this information, sites will be queried to provide the relationship based on the below categories. When answering these queries, sites will be required to select the most appropriate relationship from the options below only:

Predefined relationship options	When to use
COVID-19 health status related	i.e. patients' infection did lead to this PD
COVID-19 situation: Site issue*	e.g. site closed, personnel not available
COVID-19 situation: Lockdown / Quarantine of patient	e.g. site is active but patient not allowed to come
COVID-19 situation: Patient concern	e.g. site is active, patient could come but refused to come / do assessment
COVID-19 situation: Drug supply issue	e.g. drug was delivered to home
COVID-19 situation: Other	e.g. situation not already covered by the information above
PD without COVID relationship	e.g. situation was not in any way associated with COVID-19

*As only one option is possible, if "Site issue" is one of the relationships to COVID-19 for a PD, this should be reported as the primary relationship to COVID-19 for the PD.

Deviations related to COVID-19 will be documented as applicable and an explanation will be provided in the clinical study report (CSR) that this was attributed to the coronavirus outbreak.

14.1 Protocol Amendments

Any change or addition to the protocol can only be made in a written protocol amendment that must be approved by Advanced Accelerator Applications, health authorities where required, and the IRB/IEC prior to implementation.

Only amendments that are required for subject safety may be implemented immediately provided the health authorities are subsequently notified by protocol amendment and the reviewing IRB/IEC is notified.

Notwithstanding the need for approval of formal protocol amendments, the investigator is expected to take any immediate action required for the safety of any subject included in this study, even if this action represents a deviation from the protocol. In such cases, Advanced Accelerator Applications should be notified of this action and the IRB/IEC at the study site should be informed according to local regulations.

15 References

Anthony, L., & Vinik, A. I. (2011, 10). Evaluating the characteristics and the management of patients with neuroendocrine tumors receiving octreotide LAR during a 6-year period. *Pancreas, 40*(7), 987-994. doi:10.1097/MPA.0b013e31821f66b4

Anthony, S. S. (2004, 7). Octreotide doses used in clinical practice: Results from an internet survey and a clinical Practice. *Journal of Clinical Oncology, 22*, 4274.

Astruc, B., Marbach, P., Bouterfa, H., Denot, C., Safari, M., Vitaliti, A., & Sheppard, M. (2005, 7). Long-acting octreotide and prolonged-release lanreotide formulations have different pharmacokinetic profiles. *Journal of clinical pharmacology, 45*(7), 836-844. doi:10.1177/0091270005277936

Barani, I. J., & Larson, D. A. (2015). Radiation therapy of glioblastoma. *Cancer treatment and research, 163*, 49-73. doi:10.1007/978-3-319-12048-5_4

Bergsma, H., Konijnenberg, M. W., Kam, B. L., Teunissen, J. J., Kooij, P. P., Herder, W. W., . . . Kwekkeboom, D. J. (2016a, 3). Subacute haematotoxicity after PRRT with (177)Lu-DOTA-octreotate: prognostic factors, incidence and course. *European journal of nuclear medicine and molecular imaging, 43*(3), 453-463. doi:10.1007/s00259-015-3193-4

Bergsma, H., Konijnenberg, M. W., Zwan, W. A., Kam, B. L., Teunissen, J. J., Kooij, P. P., . . . Kwekkeboom, D. J. (2016b, 9). Nephrotoxicity after PRRT with (177)Lu-DOTA-octreotate. *European journal of nuclear medicine and molecular imaging, 43*(10), 1802-1811. doi:10.1007/s00259-016-3382-9

Brabander, T., Teunissen, J. J., Van Eijck, C. H., Franssen, G. J., Feelders, R. A., Herder, W. W., & Kwekkeboom, D. J. (2016, 1). Peptide receptor radionuclide therapy of neuroendocrine tumours. *Best practice & research. Clinical endocrinology & metabolism, 30*(1), 103-114. doi:10.1016/j.beem.2015.10.005

Brabander, T., Zwan, W. A., Teunissen, J. J., Kam, B. L., Feelders, R. A., Herder, W. W., . . . Kwekkeboom, D. J. (2017a, 8). Long-Term Efficacy, Survival, and Safety of [177Lu-DOTA0,Tyr3]octreotate in Patients with Gastroenteropancreatic and Bronchial Neuroendocrine Tumors. *Clinical cancer research : an official journal of the American Association for Cancer Research, 23*(16), 4617-4624. doi:10.1158/1078-0432.CCR-16-2743

Brabander, T., Zwan, W. A., Teunissen, J. J., Kam, B. L., Herder, W. W., Feelders, R. A., . . . Kwekkeboom, D. J. (2017b, 5). Pitfalls in the response evaluation after peptide receptor radionuclide therapy with [177Lu-DOTA0,Tyr3]octreotate. *Endocrine-related cancer, 24*(5), 243-251. doi:10.1530/ERC-16-0524

Broder, M. S., Beenhouwer, D., Strosberg, J. R., Neary, M. P., & Cherepanov, D. (2015, 2). Gastrointestinal neuroendocrine tumors treated with high dose octreotide-LAR: a systematic literature review. *World journal of gastroenterology, 21*(6), 1945-1955. doi:10.3748/wjg.v21.i6.1945

Caplin, M. E., Pavel, M., Ćwikła, J. B., Phan, A. T., Raderer, M., Sedláčková, E., . . . Investigators, C. L. (2014, 7). Lanreotide in metastatic enteropancreatic neuroendocrine

tumors. *The New England journal of medicine, 371*(3), 224-233. doi:10.1056/NEJMoa1316158

Catena, L., Bichisao, E., Milione, M., Valente, M., Platania, M., Pusceddu, S., . . . Bajetta, E. (2011). Neuroendocrine tumors of unknown primary site: gold dust or misdiagnosed neoplasms? *Tumori, 97*(5), 564-567. doi:10.1700/989.10712

Chadha, M. K., Lombardo, J., Mashtare, T., Wilding, G. E., Litwin, A., Raczyk, C., . . . Iyer, R. V. (2009, 10). High-dose octreotide acetate for management of gastroenteropancreatic neuroendocrine tumors. *Anticancer research, 29*(10), 4127-4130.

Chen, T., Miller, T. F., Prasad, P., Lee, J., Krauss, J., Miscik, K., . . . McLeod, J. F. (2000, 5). Pharmacokinetics, pharmacodynamics, and safety of microencapsulated octreotide acetate in healthy subjects. *Journal of clinical pharmacology, 40*(5), 475-481.

Clinical Trial Facilitation Group (CTFG) 2020: Recommendations related to contraception and pregnancy testing in clinical trials. Version 1.1, Sep 2020.

Coriat, R., Walter, T., Terris, B., Couvelard, A., & Ruszniewski, P. (2016, 10). Gastroenteropancreatic Well-Differentiated Grade 3 Neuroendocrine Tumors: Review and Position Statement. *The oncologist, 21*(10), 1191-1199. doi:10.1634/theoncologist.2015-0476

Dasari, A., Shen, C., Halperin, D., Zhao, B., Zhou, S., Xu, Y., ... Yao, J. C. (2017, 10). Trends in the Incidence, Prevalence, and Survival Outcomes in Patients With Neuroendocrine Tumors in the United States. *JAMA oncology, 3*(10), 1335-1342. doi:10.1001/jamaoncol.2017.0589

Dhall, D., Mertens, R., Bresee, C., Parakh, R., Wang, H. L., Li, M.,.......Wolin, E. (2012, 4). Ki-67 proliferative index predicts progression-free survival of patients with well-differentiated ileal neuroendocrine tumors. *Human pathology, 43*(4), 489-495. doi:10.1016/j.humpath.2011.06.011

Eisenhauer, E. A., Therasse, P., Bogaerts, J., Schwartz, L. H., Sargent, D., Ford, R., Verweij, J. (2009, 1). New response evaluation criteria in solid tumours: revised RECIST guideline (version 1.1). *European journal of cancer (Oxford, England : 1990), 45*(2), 228-247. doi:10.1016/j.ejca.2008.10.026

Eriksson, B., Renstrup, J., Imam, H., & Oberg, K. (1997, 10). High-dose treatment with lanreotide of patients with advanced neuroendocrine gastrointestinal tumors: clinical and biological effects. *Annals of oncology : official journal of the European Society for Medical Oncology, 8*(10), 1041-1044.

Faiss, S., Pape, U.-F., Böhmig, M., Dörffel, Y., Mansmann, U., Golder, W., . . . Group, I. A. (2003, 7). Prospective, randomized, multicenter trial on the antiproliferative effect of lanreotide, interferon alfa, and their combination for therapy of metastatic neuroendocrine gastroenteropancreatic tumors--the International Lanreotide and Interferon Alfa Study Group. *Journal of clinical oncology : official journal of the American Society of Clinical Oncology, 21*(14), 2689-2696. doi:10.1200/JCO.2003.12.142

Faiss, S., Räth, U., Mansmann, U., Caird, D., Clemens, N., Riecken, E. O., & Wiedenmann, B. (1999). Ultra-high-dose lanreotide treatment in patients with metastatic neuroendocrine gastroenteropancreatic tumors. *Digestion, 60*(5), 469-476. doi:10.1159/000007693

Ferolla, P., Faggiano, A., Grimaldi, F., Ferone, D., Scarpelli, G., Ramundo, V., . . . Colao, A. (2012, 3). Shortened interval of long-acting octreotide administration is effective in patients with well-differentiated neuroendocrine carcinomas in progression on standard doses. *Journal of endocrinological investigation, 35*(3), 326-331. doi:10.3275/7869

Forrer, F., Uusijärvi, H., Storch, D., Maecke, H. R., & Mueller-Brand, J. (2005, 8). Treatment with 177Lu-DOTATOC of patients with relapse of neuroendocrine tumors after treatment with 90Y-DOTATOC. *Journal of nuclear medicine : official publication, Society of Nuclear Medicine, 46*(8), 1310-1316.

Halperin, D. M., Shen, C., Dasari, A., Xu, Y., Chu, Y., Zhou, S., . . . Yao, J. C. (2017, 4). Frequency of carcinoid syndrome at neuroendocrine tumour diagnosis: a population-based study. *The Lancet. Oncology, 18*(4), 525-534. doi:10.1016/S1470-2045(17)30110-9

Hammond, P. J., Wade, A. F., Gwilliam, M. E., Peters, A. M., Myers, M. J., Gilbey, S. G., . . . Calam, J. (1993, 6). Amino acid infusion blocks renal tubular uptake of an indium-labelled somatostatin analogue. *British journal of cancer, 67*(6), 1437-1439.

Heetfeld, M., Chougnet, C. N., Olsen, I. H., Rinke, A., Borbath, I., Crespo, G., . . . Knowledge Network members. (2015, 8). Characteristics and treatment of patients with G3 gastroenteropancreatic neuroendocrine neoplasms. *Endocrine-related cancer, 22*(4), 657-664. doi:10.1530/ERC-15-0119

Herder, W. W., Kwekkeboom, D. J., Feelders, R. A., Aken, M. O., Lamberts, S. W., Lely, A.-J., & Krenning, E. P. (2006). Somatostatin receptor imaging for neuroendocrine tumors. *Pituitary, 9*(3), 243-248. doi:10.1007/s11102-006-0270-5

Hope, T. A., Bergsland, E. K., Bozkurt, M. F., Graham, M., Heaney, A. P., Herrmann, K., . . . Strosberg, J. R. (2018, 1). Appropriate Use Criteria for Somatostatin Receptor PET Imaging in Neuroendocrine Tumors. *Journal of nuclear medicine : official publication, Society of Nuclear Medicine, 59*(1), 66-74. doi:10.2967/jnumed.117.202275

Howell, D. L., & O'Dorisio, M. S. (2012, 5). Management of neuroendocrine tumors in children, adolescents, and young adults. *Journal of pediatric hematology/oncology, 34 Suppl 2*, S64--S68. doi:10.1097/MPH.0b013e31824e3885

Imam, H., Eriksson, B., Lukinius, A., Janson, E. T., Lindgren, P. G., Wilander, E., & Oberg, K. (1997). Induction of apoptosis in neuroendocrine tumors of the digestive system during treatment with somatostatin analogs. *Acta oncologica (Stockholm, Sweden), 36*(6), 607-614.

Inzani, F., Petrone, G., & Rindi, G. (2018, 9). The New World Health Organization Classification for Pancreatic Neuroendocrine Neoplasia. *Endocrinology and metabolism clinics of North America, 47*(3), 463-470. doi:10.1016/j.ecl.2018.04.008

Inzani, F., Petrone, G., Fadda, G., & Rindi, G. (2017, 12). Cyto-histology in NET: what is necessary today and what is the future? *Reviews in endocrine & metabolic disorders, 18*(4), 381-391. doi:10.1007/s11154-017-9428-x

Jann, H., Roll, S., Couvelard, A., Hentic, O., Pavel, M., Müller-Nordhorn, J., . . . Pape, U.-F. (2011, 8). Neuroendocrine tumors of midgut and hindgut origin: tumor-node-metastasis classification determines clinical outcome. *Cancer, 117*(15), 3332-3341. doi:10.1002/cncr.25855

Joseph, S., Li, G., Lindholm, E., Zhou, Y., Go, V. L., Vinik, A. I., . . . Woltering, E. A. (2010, 10). A prospective trial on the effect of body mass index and sex on plasma octreotide levels in patients undergoing long-term octreotide LAR therapy. *Pancreas, 39*(7), 964-966. doi:10.1097/MPA.0b013e3181db01a8

Keck, K. J., Choi, A., Maxwell, J. E., Li, G., O'Dorisio, T. M., Breheny, P., . . . Howe, J. R. (2017, 8). Increased Grade in Neuroendocrine Tumor Metastases Negatively Impacts Survival. *Annals of surgical oncology, 24*(8), 2206-2212. doi:10.1245/s10434-017-5899-y

Kirshbom, P. M., Kherani, A. R., Onaitis, M. W., Feldman, J. M., & Tyler, D. S. (1998, 12). Carcinoids of unknown origin: comparative analysis with foregut, midgut, and hindgut carcinoids. *Surgery, 124*(6), 1063-1070.

Klöppel, G. (2011, 10). Classification and pathology of gastroenteropancreatic neuroendocrine neoplasms. *Endocrine-related cancer, 18 Suppl 1*, S1--16. doi:10.1530/ERC-11-0013

Kneifel, S., Cordier, D., Good, S., Ionescu, M. C., Ghaffari, A., Hofer, S., . . . Merlo, A. (2006, 6). Local targeting of malignant gliomas by the diffusible peptidic vector 1,4,7,10-tetraazacyclododecane-1-glutaric acid-4,7,10-triacetic acid-substance p. *Clinical cancer research : an official journal of the American Association for Cancer Research, 12*(12), 3843-3850. doi:10.1158/1078-0432.CCR-05-2820

Kulke, M. H., Shah, M. H., Benson, A. B., Bergsland, E., Berlin, J. D., Blaszkowsky, L. S., . . . National. (2015, 1). Neuroendocrine tumors, version 1.2015. *Journal of the National Comprehensive Cancer Network : JNCCN, 13*(1), 78-108.

Kunz, P. L., Reidy-Lagunes, D., Anthony, L. B., Bertino, E. M., Brendtro, K., Chan, J. A., . . . Society, N. A. (2013, 5). Consensus guidelines for the management and treatment of neuroendocrine tumors. *Pancreas, 42*(4), 557-577. doi:10.1097/MPA.0b013e31828e34a4

Kwekkeboom, D. J., & Krenning, E. P. (2016, 2). Peptide Receptor Radionuclide Therapy in the Treatment of Neuroendocrine Tumors. *Hematology/oncology clinics of North America, 30*(1), 179-191. doi:10.1016/j.hoc.2015.09.009

Kwekkeboom, D. J., Bakker, W. H., Kam, B. L., Teunissen, J. J., Kooij, P. P., Herder, W. W., . . . Krenning, E. P. (2003, 3). Treatment of patients with gastro-entero-pancreatic (GEP) tumours with the novel radiolabelled somatostatin analogue [177Lu-DOTA(0),Tyr3]octreotate. *European journal of nuclear medicine and molecular imaging, 30*(3), 417-422. doi:10.1007/s00259-002-1050-8

Kwekkeboom, D. J., Bakker, W. H., Kooij, P. P., Konijnenberg, M. W., Srinivasan, A., Erion, J. L., . . . Krenning, E. P. (2001, 9). [177Lu-DOTAOTyr3]octreotate: comparison with [111In-DTPAo]octreotide in patients. *European journal of nuclear medicine, 28*(9), 1319-1325.

Kwekkeboom, D. J., Herder, W. W., Kam, B. L., Eijck, C. H., Essen, M., Kooij, P. P., . . . Krenning, E. P. (2008, 5). Treatment with the radiolabeled somatostatin analog [177 Lu-DOTA 0,Tyr3]octreotate: toxicity, efficacy, and survival. *Journal of clinical oncology : official journal of the American Society of Clinical Oncology, 26*(13), 2124-2130. doi:10.1200/JCO.2007.15.2553

Kwekkeboom, D. J., Teunissen, J. J., Bakker, W. H., Kooij, P. P., Herder, W. W., Feelders, R. A., . . . Krenning, E. P. (2005, 4). Radiolabeled somatostatin analog [177Lu-DOTA0,Tyr3]octreotate in patients with endocrine gastroenteropancreatic tumors. *Journal of clinical oncology : official journal of the American Society of Clinical Oncology, 23*(12), 2754-2762. doi:10.1200/JCO.2005.08.066

Lau, D., Rutledge, C., & Aghi, M. K. (2015, 2). Cushing's disease: current medical therapies and molecular insights guiding future therapies. *Neurosurgical focus, 38*(2), E11. doi:10.3171/2014.10.FOCUS14700

Lau, S. C., Abdel-Rahman, O., & Cheung, W. Y. (2018, 8). Improved survival with higher doses of octreotide long-acting release in gastroenteropancreatic neuroendocrine tumors. *Medical oncology (Northwood, London, England), 35*(9), 123. doi:10.1007/s12032-018-1189-1

Ludlam, W. H., & Anthony, L. (2011, 10). Safety review: dose optimization of somatostatin analogs in patients with acromegaly and neuroendocrine tumors. *Advances in therapy, 28*(10), 825-841. doi:10.1007/s12325-011-0062-9

Massironi, S., Conte, D., & Rossi, R. E. (2016). Somatostatin analogues in functioning gastroenteropancreatic neuroendocrine tumours: literature review, clinical recommendations and schedules. *Scandinavian journal of gastroenterology, 51*(5), 513-523. doi:10.3109/00365521.2015.1115117

Meachem, S. J., Nieschlag, E., & Simoni, M. (2001, 11). Inhibin B in male reproduction: pathophysiology and clinical relevance. *European journal of endocrinology, 145*(5), 561-571.

Modlin, I. M., Lye, K. D., & Kidd, M. (2003, 2). A 5-decade analysis of 13,715 carcinoid tumors. *Cancer, 97*(4), 934-959. doi:10.1002/cncr.11105

NICE guideline on Type 2 diabetes in adults: management, 2015. Published: 2 December 2015. Last updated: 16 December 2020. www.nice.org.uk/guidance/ng28

Oberg, K. (2012b, 7). Neuroendocrine tumors of the digestive tract: impact of new classifications and new agents on therapeutic approaches. *Current opinion in oncology, 24*(4), 433-440. doi:10.1097/CCO.0b013e328353d7ba

Oberg, K., Knigge, U., Kwekkeboom, D., Perren, A., & Group, E. S. (2012, 10). Neuroendocrine gastro-entero-pancreatic tumors: ESMO Clinical Practice Guidelines for diagnosis,

treatment and follow-up. *Annals of oncology : official journal of the European Society for Medical Oncology, 23 Suppl 7*, vii124--vii130. doi:10.1093/annonc/mds295

Pape, U.-F., Perren, A., Niederle, B., Gross, D., Gress, T., Costa, F., . . . B. C. (2012). ENETS Consensus Guidelines for the management of patients with neuroendocrine neoplasms from the jejuno-ileum and the appendix including goblet cell carcinomas. *Neuroendocrinology, 95*(2), 135-156. doi:10.1159/000335629

Pavel, M. E., Hainsworth, J. D., Baudin, E., Peeters, M., Hörsch, D., Winkler, R. E., . . . Group, R. A.-2. (2011, 12). Everolimus plus octreotide long-acting repeatable for the treatment of advanced neuroendocrine tumours associated with carcinoid syndrome (RADIANT-2): a randomised, placebo-controlled, phase 3 study. *Lancet (London, England), 378*(9808), 2005-2012. doi:10.1016/S0140-6736(11)61742-X

Pavel, M., Baudin, E., Couvelard, A., Krenning, E., Öberg, K., Steinmüller, T., . . . B. C. (2012). ENETS Consensus Guidelines for the management of patients with liver and other distant metastases from neuroendocrine neoplasms of foregut, midgut, hindgut, and unknown primary. *Neuroendocrinology, 95*(2), 157-176. doi:10.1159/000335597

Pelosi, G., Rindi, G., Travis, W. D., & Papotti, M. (2014, 3). Ki-67 antigen in lung neuroendocrine tumors: unraveling a role in clinical practice. *Journal of thoracic oncology : official publication of the International Association for the Study of Lung Cancer, 9*(3), 273-284. doi:10.1097/JTO.0000000000000092

Perren, A., Couvelard, A., Scoazec, J.-Y., Costa, F., Borbath, I., Delle Fave, G., . . . A. C. (2017). ENETS Consensus Guidelines for the Standards of Care in Neuroendocrine Tumors: Pathology: Diagnosis and Prognostic Stratification. *Neuroendocrinology, 105*(3), 196-200. doi:10.1159/000457956

Pierik, F. H., Vreeburg, J. T., Stijnen, T., De Jong, F. H., & Weber, R. F. (1998, 9). Serum inhibin B as a marker of spermatogenesis. *The Journal of clinical endocrinology and metabolism, 83*(9), 3110-3114. doi:10.1210/jcem.83.9.5121

Reubi, J. C. (2003, 8). Peptide receptors as molecular targets for cancer diagnosis and therapy. *Endocrine reviews, 24*(4), 389-427. doi:10.1210/er.2002-0007

Reubi, J. C., Schär, J. C., Waser, B., Wenger, S., Heppeler, A., Schmitt, J. S., & Mäcke, H. R. (2000, 3). Affinity profiles for human somatostatin receptor subtypes SST1-SST5 of somatostatin radiotracers selected for scintigraphic and radiotherapeutic use. *European journal of nuclear medicine, 27*(3), 273-282.

Reubi, J. C., Waser, B., Schaer, J. C., & Laissue, J. A. (2001, 7). Somatostatin receptor sst1-sst5 expression in normal and neoplastic human tissues using receptor autoradiography with subtype-selective ligands. *European journal of nuclear medicine, 28*(7), 836-846.

Rindi, G. (2010). The ENETS guidelines: the new TNM classification system. *Tumori, 96*(5), 806-809.

Rindi, G., Klersy, C., Albarello, L., Baudin, E., Bianchi, A., Buchler, M. W., . . . Falconi, M. (2018). Competitive Testing of the WHO 2010 versus the WHO 2017 Grading of

Pancreatic Neuroendocrine Neoplasms: Data from a Large International Cohort Study. *Neuroendocrinology, 107*(4), 375-386. doi:10.1159/000494355

Rindi, G., Klimstra, D. S., Abedi-Ardekani, B., Asa, S. L., Bosman, F. T., Brambilla, E., . . . Cree, I. A. (2018, 12). A common classification framework for neuroendocrine neoplasms: an International Agency for Research on Cancer (IARC) and World Health Organization (WHO) expert consensus proposal. *Modern pathology : an official journal of the United States and Canadian Academy of Pathology, Inc, 31*(12), 1770-1786. doi:10.1038/s41379-018-0110-y

Rindi, G., Petrone, G., & Inzani, F. (2014, 6). The 2010 WHO classification of digestive neuroendocrine neoplasms: a critical appraisal four years after its introduction. *Endocrine pathology, 25*(2), 186-192. doi:10.1007/s12022-014-9313-z

Rinke, A., Müller, H.-H., Schade-Brittinger, C., Klose, K.-J., Barth, P., Wied, M., . . . Group, P. R. (2009, 10). Placebo-controlled, double-blind, prospective, randomized study on the effect of octreotide LAR in the control of tumor growth in patients with metastatic neuroendocrine midgut tumors: a report from the PROMID Study Group. *Journal of clinical oncology : official journal of the American Society of Clinical Oncology, 27*(28), 4656-4663. doi:10.1200/JCO.2009.22.8510

Rolleman, E. J., Valkema, R., Jong, M., Kooij, P. P., & Krenning, E. P. (2003, 1). Safe and effective inhibition of renal uptake of radiolabelled octreotide by a combination of lysine and arginine. *European journal of nuclear medicine and molecular imaging, 30*(1), 9-15. doi:10.1007/s00259-002-0982-3

Sackstein, P. E., O'Neil, D. S., Neugut, A. I., Chabot, J., & Fojo, T. (2018, 8). Epidemiologic trends in neuroendocrine tumors: An examination of incidence rates and survival of specific patient subgroups over the past 20 years. *Seminars in oncology, 45*(4), 249-258. doi:10.1053/j.seminoncol.2018.07.001

Sierra, M. L., Agazzi, A., Bodei, L., Pacifici, M., Aricò, D., De Cicco, C., . . . Paganelli, G. (2009, 12). Lymphocytic toxicity in patients after peptide-receptor radionuclide therapy (PRRT) with 177Lu-DOTATATE and 90Y-DOTATOC. *Cancer biotherapy & radiopharmaceuticals, 24*(6), 659-665. doi:10.1089/cbr.2009.0641

Singh, S., Carnaghi, C., Buzzoni, R., Pommier, R. F., Raderer, M., Tomasek, J., . . . RAD001 in Advanced Neuroendocrine Tumors, F. T.-4. (2018). Everolimus in Neuroendocrine Tumors of the Gastrointestinal Tract and Unknown Primary. *Neuroendocrinology, 106*(3), 211-220. doi:10.1159/000477585

Sorbye, H., Baudin, E., Borbath, I., Caplin, M., Chen, J., Cwikla, J. B., . . . ENETS 2016 Munich Advisory Board Participants. (2019). Unmet Needs in High-Grade Gastroenteropancreatic Neuroendocrine Neoplasms (WHO G3). *Neuroendocrinology, 108*(1), 54-62. doi:10.1159/000493318

Strosberg, J., El-Haddad, G., Wolin, E., Hendifar, A., Yao, J., Chasen, B., . . . Investigators, N. E.-1. (2017, 1). Phase 3 Trial of , javax.xml.bind.JAXBElement@11e0f286, Lu-Dotatate for Midgut Neuroendocrine Tumors. *The New England journal of medicine, 376*(2), 125-135. doi:10.1056/NEJMoa1607427

Strosberg, J., Wolin, E., Chasen, B., Kulke, M., Bushnell, D., Caplin, M., . . . Group, N. E.-1. (2018, 9). Health-Related Quality of Life in Patients With Progressive Midgut Neuroendocrine Tumors Treated With , javax.xml.bind.JAXBElement@52bac87, Lu-Dotatate in the Phase III NETTER-1 Trial. *Journal of clinical oncology : official journal of the American Society of Clinical Oncology, 36*(25), 2578-2584. doi:10.1200/JCO.2018.78.5865

Strosberg J, Leeuwenkamp O, Siddiqui MK (2021) Peptide receptor radiotherapy re-treatment in patients with progressive neuroendocrine tumors: A systematic review and meta-analysis. Cancer Treat Rev; 93:102141.

Teunissen, J. J., Krenning, E. P., Jong, F. H., Rijke, Y. B., Feelders, R. A., Aken, M. O., . . . Kwekkeboom, D. J. (2009, 11). Effects of therapy with [177Lu-DOTA 0,Tyr 3]octreotate on endocrine function. *European journal of nuclear medicine and molecular imaging, 36*(11), 1758-1766. doi:10.1007/s00259-009-1151-8

Uri, I., & Grozinsky-Glasberg, S. (2018). Current treatment strategies for patients with advanced gastroenteropancreatic neuroendocrine tumors (GEP-NETs). *Clinical diabetes and endocrinology, 4*, 16. doi:10.1186/s40842-018-0066-3

van der Zwan, W. A., Brabander, T., Kam, B. L., Teunissen, J. J., Feelders, R. A., Hofland, J., . . . Herder, W. W. (2019, 3). Salvage peptide receptor radionuclide therapy with [177Lu-DOTA,Tyr3]octreotate in patients with bronchial and gastroenteropancreatic neuroendocrine tumours. *European journal of nuclear medicine and molecular imaging, 46*(3), 704-717. doi:10.1007/s00259-018-4158-1

Vélayoudom-Céphise, F.-L., Duvillard, P., Foucan, L., Hadoux, J., Chougnet, C. N., Leboulleux, S., . . . Baudin, E. (2013, 10). Are G3 ENETS neuroendocrine neoplasms heterogeneous? *Endocrine-related cancer, 20*(5), 649-657. doi:10.1530/ERC-13-0027

Welin, S. V., Janson, E. T., Sundin, A., Stridsberg, M., Lavenius, E., Granberg, D., . . . Eriksson, B. K. (2004, 7). High-dose treatment with a long-acting somatostatin analogue in patients with advanced midgut carcinoid tumours. *European journal of endocrinology, 151*(1), 107-112.

Wolchok, J. D., Hoos, A., O'Day, S., Weber, J. S., Hamid, O., Lebbé, C., . . . Hodi, F. S. (2009, 12). Guidelines for the evaluation of immune therapy activity in solid tumors: immune-related response criteria. *Clinical cancer research : an official journal of the American Association for Cancer Research, 15*(23), 7412-7420. doi:10.1158/1078-0432.CCR-09-1624

Wolin, E. M. (2012, 9). The expanding role of somatostatin analogs in the management of neuroendocrine tumors. *Gastrointestinal cancer research : GCR, 5*(5), 161-168.

Yao, J. C., Fazio, N., Singh, S., Buzzoni, R., Carnaghi, C., Wolin, E., . . . RAD001 in Advanced Neuroendocrine Tumours, F. T.-4. (2016, 3). Everolimus for the treatment of advanced, non-functional neuroendocrine tumours of the lung or gastrointestinal tract (RADIANT-4): a randomised, placebo-controlled, phase 3 study. *Lancet (London, England), 387*(10022), 968-977. doi:10.1016/S0140-6736(15)00817-X

Yao, J. C., Hassan, M., Phan, A., Dagohoy, C., Leary, C., Mares, J. E., . . . Evans, D. B. (2008, 6). One hundred years after "carcinoid": epidemiology of and prognostic factors for neuroendocrine tumors in 35,825 cases in the United States. *Journal of clinical oncology : official journal of the American Society of Clinical Oncology, 26*(18), 3063-3072. doi:10.1200/JCO.2007.15.4377

Yao, J. C., Lombard-Bohas, C., Baudin, E., Kvols, L. K., Rougier, P., Ruszniewski, P., . . . Wiedenmann, B. (2010, 1). Daily oral everolimus activity in patients with metastatic pancreatic neuroendocrine tumors after failure of cytotoxic chemotherapy: a phase II trial. *Journal of clinical oncology : official journal of the American Society of Clinical Oncology, 28*(1), 69-76. doi:10.1200/JCO.2009.24.2669

Yao, J. C., Shah, M. H., Ito, T., Bohas, C. L., Wolin, E. M., Van Cutsem, E., . . . RAD001 in Advanced Neuroendocrine Tumors, T. T.-3. (2011, 2). Everolimus for advanced pancreatic neuroendocrine tumors. *The New England journal of medicine, 364*(6), 514-523. doi:10.1056/NEJMoa1009290

Zatelli, M. C., Guadagno, E., Messina, E., Lo Calzo, F., Faggiano, A., Colao, A., & Group, N. I. (2018, 6). Open issues on G3 neuroendocrine neoplasms: back to the future. *Endocrine-related cancer, 25*(6), R375--R384. doi:10.1530/ERC-17-0507

16 Appendices

Appendix 1 – Precautions for Pregnancy

Women who receive Lutathera, including those in cross-over arm and those who are re-treated with Lutathera, should not procreate until seven months after the date of their last treatment with ^{177}Lu-DOTA^{0}-Tyr^{3}-Octreotate, or until the end of treatment period of the study (due to the CT scans foreseen during the study (every 12±1 weeks)), whichever is longer. Women in the control arm should not procreate until the end of treatment period.

It is noteworthy that β-HCG may be secreted by a small percentage of NETs, such that, in addition to being a pregnancy marker it also is a tumor marker. Consequently, NET female patients with positive β-HCG at baseline can be eligible to enter the study and receive treatment if pregnancy can be excluded by lack of expected doubling of β-HCG. Normally in pregnant subjects β-HCG doubles every 2 days during the first 4 weeks of pregnancy and every 3 ½ days by weeks 6 to 7.

Women of childbearing potential include any female who has experienced menarche and who has not undergone successful surgical sterilisation (hysterectomy, bilateral tubal ligation or bilateral ovariectomy) or is not postmenopausal (defined as amenorrhoea >12 consecutive months, and for women on hormone replacement therapy, only with a documented plasma follicle-stimulating hormone level >35 mIU/mL). Even women who are using oral, implanted or injected contraceptive hormones, an IUD, or barrier methods (diaphragm, condoms, spermicidal) to prevent pregnancy, are practicing true abstinence (see definition below) or where the partner is sterile (e.g. vasectomy, see below) should be considered to be of childbearing potential. Postmenopausal women who have fertilised eggs implanted are also considered to be of childbearing potential.

Highly effective methods of contraception must be used throughout the study and for 6 months after study drug discontinuation. Highly effective contraception methods include:

- True abstinence must be in line with the preferred and usual lifestyle of the patient. Periodic abstinence (e.g., calendar, ovulation, symptothermal, post-ovulation methods), declaration of abstinence for the duration of exposure to IMP, and withdrawal are not acceptable methods of contraception.
- Male or female sterilization (vasectomized partner is a highly effective birth control method provided that partner is the sole sexual partner of the trial participant and that the vasectomized partner has received medical assessment of the surgical success).
- Combination of any two of the following (a+b or a+c or b+c):

a. Use of oral, injected, or implanted hormonal methods of contraception. In case of use of oral contraception, women should be stable on the same pill for a minimum of 3 months before taking study treatment.

b. Placement of an intrauterine device (IUD) or intrauterine system (IUS)

c. Barrier methods of contraception: condom or occlusive cap (diaphragm or cervical/vault caps) with spermicidal foam/gel/film/cream/vaginal suppository. Post-menopausal women are allowed to participate in this study. Women are considered post-menopausal and not of child bearing potential if they have had 12 months of natural (spontaneous) amenorrhea with an appropriate clinical profile (e.g. age appropriate, history of vasomotor symptoms) confirmed by a high follicle stimulating hormone (FSH) level, or have had surgical bilateral oophorectomy (with or without hysterectomy) or tubal ligation at least six weeks prior to screening. In the case of oophorectomy alone, only when the reproductive status of the woman has been confirmed by follow up hormone level assessment is she considered not of child bearing potential.

Sexually active males in the Lutathera arm must:

- remain abstinent (refrain from heterosexual intercourse) or be willing to use condoms and highly effective methods of contraception with female partners of childbearing potential or pregnant female partners (see above) during the treatment period and for at least 4 months after the last dose of Lutathera (including cross-over and re-treatment, if applicable). Men should not father a child in this period. A condom is required to be used also by vasectomized men in order to prevent delivery of the drug via seminal fluid.
- refrain from donating sperm during this same period.

Appendix 2 – Lutathera Administration and Sterile Amino Acid Co-Infusion Scheme

The gravity method is a recommended infusion method of Lutathera. Please refer to the pharmacy manual for detailed guidance on Lutathera administration and sterile amino acid co-infusion scheme.

Appendix 3 – Karnofsky Performance Scale

Able to carry on normal activity and to work; no special care needed.	100	Normal no complaints; no evidence of disease.
	90	Able to carry on normal activity; minor signs or symptoms of disease.
	80	Normal activity with effort; some signs or symptoms of disease.
Unable to work; able to live at home and care for most personal needs; varying amount of assistance needed.	70	Cares for self; unable to carry on normal activity or to do active work.
	60	Requires occasional assistance, but is able to care for most of his personal needs.
	50	Requires considerable assistance and frequent medical care.
Unable to care for self; requires equivalent of institutional or hospital care; disease may be progressing rapidly.	40	Disabled; requires special care and assistance.
	30	Severely disabled; hospital admission is indicated although death not imminent.
	20	Very sick; hospital admission necessary; active supportive treatment necessary.
	10	Moribund; fatal processes progressing rapidly.
	0	Dead

Appendix 4 – Radioprotection Precautions for Treated with Lutathera ^{177}Lu-DOTA^{0}-Tyr^{3}-Octreotate

- **Technical Data**

The principle characteristics of lutetium-177 (^{177}Lu) are specified in Table 1 (from *Les principales caractéristiques du 177 lutetium (*^{177}*Lu*) and *Guide Pratique Radionucléides & Radioprotection*, D. Delacroix, J.P. Guerre, P. Leblanc, EDP Sciences, 2004).

Table 1: Emissions of ^{177}Lu

$^{177}_{71}$Lu Période : 6,71 jours Activité massique : 4,07.10^{15} Bq.g^{-1} Groupe de risque : 4

Lutétium - 177

Principales émissions

	Gamma / X		Beta (Emax)		Electrons		Alpha	
	E (keV)	%	E (keV)	%	E (keV)	%	E (keV)	%
E1	55	5	208	11	6	9		
E2	113	6	384	9	48	5		
E3	208	11	497	79	110	9		
% omis		5		< 1		4		

Seuils d'exemption

Quantité en Bq	1.10^{7}
Concentration en Bq.g^{-1}	1.10^{3}

Transport (Bq)

A1	3.10^{13}
A2	7.10^{11}

^{177}Lu is generally produced using enriched ^{176}Lu placed in a reactor, following a reaction of the type [^{176}Lu n,γ ^{177}Lu]. This reaction results the production of a small amount of ^{177}Lu meta-stable (^{177m}Lu) which varies according to sources between 10^{-5} and 4×10^{-4} per MBq of ^{177}Lu produced. The characteristics of this radionuclide are given in Table 2.

Table 2: Emissions of ^{177m}Lu

Période : 160,4 jours Activité massique : 1,69.10^{14} Bq.g^{-1} Groupe de risque : 3*
Descendant : Embr. = 0,215 ; T = 6,71 jours
$^{177m}_{71}$Lu/$^{177}_{71}$Lu

Lutétium - 177m / Lutétium - 177

Principales émissions

	Gamma / X		Beta (Emax)		Electrons		Alpha	
	E (keV)	%	E (keV)	%	E (keV)	%	E (keV)	%
E1	328	18	152	79	5	60		
E2	379	28	384	2	45	51		
E3	419	20	497	17	95	41		
% omis		450		12		60		

Seuils d'exemption

Quantité en Bq	-
Concentration en Bq.g^{-1}	-

Transport (Bq)

A1	-
A2	-

- **Recommendations**

Based on radiation exposure calculations employing whole body clearance data (Wehrmann C et al, 2007) and exposure rates at one meter at 24 h (7.5 ± 3.6 µSv/h, Dpt. of Nucl. Med. Erasmus MC Rotterdam), patients may be treated on an outpatient basis for an administration of 7.4 GBq of ^{177}Lu-DOTA^{0}-Tyr^{3}-Octreotate. As a precaution, it is recommended that patients be kept in radiation isolation for a period of 4 – 5 hours following administration, and be allowed to urinate during that time, and before release. This precaution is deemed prudent because this is the route of Lutathera elimination (approximately 50% of Lutathera eliminated by urination within the first 6 hours; Kwekkeboom et al, 2001). At the time of release, the patient is given written instructions which summarize the precautions to take, in order to keep the exposure to others below regulatory limits.

Appendix 5 – Recommendations for Patients Treated with Lutathera

In accordance with the medical staff, you have agreed to receive a treatment using a radioactive medicine. We think that the potential activity of this medicine in treating your tumor is due to the radioactivity of this medicine; it is also for this reason that it is necessary to follow certain precautions in order to limit the exposure of the people around you and to avoid contaminating them with radioactivity.
Because of knowledge and experience in the field, it is estimated that the health risks to your family members and the general public are low because of the physical and radiopharmaceutical properties and the metabolism of the radiopharmaceutical. You must adhere to the following rules to maximize the safety of other persons. They are the result of many years of experience in the use of radioactivity in medicine, and they include recommendations by international organizations.

1- General rule
You should limit close contact with people who live with you by keeping a distance of at least one meter for 7-days after you receive Lutathera.

2- Use of toilets
Toilets must be used in a seated position, even for men. It is absolutely necessary to use toilet paper each time. It is equally important to wash your hands to avoid contaminating the door handles. It is strongly recommended to have bowel movements every day and to use a laxative if you need help. Furthermore, empty your bladder (urinate) every hour or so on the day you received treatment and for day after. Follow your doctor's advice on how much fluid to drink. Furthermore, after you receive treatment, empty your bladder (urinate) every hour on the day of treatment and for two more days after treatment. After expelling fluids, drink a glass of water. You can substitute juice or a sports drink as a means to replace expelled fluids. Follow any additional advice that your doctor provides on how much to drink.

3- Contact with children (less than 10 years old)
After your receive Lutathera, it is strongly recommended that you limit close contact (less than 1 meter) with children to less than 15 minutes per day for 7 days.

4- Contact with spouse and people in the family circle
You should sleep in a separate bedroom from other people for 7 days after you receive Lutathera. You should sleep in separate bedrooms from children and/or pregnant women for 15 days after you receive Lutathera.

5- Seniors
Older people are less sensitive to radioactivity (between 3 and 10 times less than a middle-aged person). Therefore, the previous recommendations can be followed with a little more flexibility in the presence of the elderly.

6- Contact with pregnant women
Contact with pregnant women should follow the same restrictions recommended for children less than 10 years old.

7- Breastfeeding

Breastfeeding should be stopped because it is not compatible with a treatment using a radioactive product. If treatment with Lutathera during breast feeding is necessary, the child must be weaned.

8- Pregnancy

Pregnancy must be excluded before the start of treatment. Any woman who has missed a period must be assumed to be pregnant until proven otherwise and alternative therapies which do not involve ionizing radiation must be then considered.

There is a potential risk that ionizing radiation by Lutathera could cause toxic effects on female and male gonads. Due to the nature of the compound, women who receive Lutathera, including those in cross-over arm and those who are re-treated with Lutathera, should not procreate until seven months after the last Lutathera dose, or until the end of treatment period of the study (due to the CT scans foreseen during the study), whichever is longer. Sexually active males in the Lutathera arm must use condoms and highly effective methods of contraception with female partners of childbearing potential or pregnant female partners during the treatment period and for at least 4 months after the last dose of Lutathera (including cross-over and re-treatment, if applicable). Females of reproductive potential who are partners of male patients should be advised to use highly effective contraception during treatment and for 4 months after the last dose of Lutathera.

9- People who need extra assistance

People who are confined to the bed or have reduced mobility will preferably receive assistance by a care provider. It is recommended that when providing assistance in the bathroom, the care provider wear disposable gloves for 7 days after administration. In the case of the use of special medical equipment such as catheters, colostomy bags bedpan, water nozzle, or anything that could be contaminated by your body fluids they must be emptied immediately in the toilet and then cleaned. If anyone helps you clean up vomit, blood, urine, or stool they should wear plastic gloves; the gloves should then be put in the specified trash plastic bag.

10- Dishes and bathroom accessories

For the first two days after your treatment wipes and/or toilet paper must be flushed down the toilet. Always wash your hands well after using the toilet.

It is strongly recommended to shower every day for at least the first 7 days after your treatment.

Try to flush any tissues or any other items that contain anything from your body, such as blood, urine and faeces down the toilet (at least for two days after the therapy). Items that cannot be flushed, such as menstrual pads and bandages, must be placed in specified plastic trash bags.

Wash your underwear, pyjamas, sheets and any clothes that contain sweat, blood or urine separately from the laundry of others in your household. Wash your items two or three times; use a standard washing machine; you do not need to use bleach and do not need extra rinses.

11- Trash recommendations

Keep the specified plastic trash bags separate from other trash; keep the bags away from children and animals.

A member of the Study Staff will tell you how and when to get rid of the specified plastic trash bag; you may be asked to bring the bag back to your treatment facility, or, after 70 days, the bag may be removed as other trash bags.

12- Professional activities

Lutathera could affect your ability to drive and to use machines, as dizziness has been reported as a common side effect.

If there is a risk of frequent contact and being in close vicinity to the public and/or with children, the activity must be temporarily suspended.

13- Use of public transportation

For short trips (less than 30 minutes), the precautions are minimum. If you ride with someone else, confirm she is not pregnant, and maintain a distance of >1 meter (use the back seat on opposite side of the driver). If you are able to do so, it is best to drive yourself.

14- Public activities

Avoid assisting in shows or public meetings which could expose third-parties for more than 30 minutes in the first week after your treatment.

Ask Your Doctor or a member of the Study Staff when:

- It will be safe to eat out, go shopping and attend events such as religious services, parties and movies;
- You will be able to return to work and to care for or teach others;
- It would be safe to donate blood;
- Special or longer distance travel is possible (Note: For up to 3 months or more following radioactive treatment you may set off radiation detectors at: national borders, airports, bus and train stations, tunnels, bridges, trash collection sites and even your place of employment); a member of your Study Staff will issue you a letter or card describing the therapy and the phone number of a person knowledgeable about your treatment (usually at the treating facility) in case local law enforcement agents need to check on this information; you should keep the letter or card containing the information with you whenever you are travelling for at least 3 months.

15- Hospitalization

In the case that an unplanned hospitalization occurs, it is important to notify your doctor.

There is a possibility that due to an excessive release of hormones following the administration of ^{177}Lu-DOTA^{0}-Tyr^{3}-Octreotate your doctor may request that you stay in hospital overnight for observation and treatment if necessary, normally consists of intravenous fluids, corticosteroids and the correction of any chemical imbalance in the blood.

16- Domesticated animals

The lifespan of domesticated animals is much less than that of humans. Therefore, the effect of the radioactivity is less. It is not necessary to take any particular precautions. But do not sleep with pets (ask your doctor for how long) since your secretions may be carried away by the pet.

17- Emergency Care

You will get an information card or letter at the time of your treatment that will show the date, type and amount of radioactivity that you were treated with; carry this card with you at all times for at least 3 months following your treatment.

If you are in a traffic accident or any other medical emergency and require medical assistance during the first week after your treatment, you should show this card to the medical providers to let them know about the date and dose of your radioactive treatment.

18- Important information for patients on risks of radiation

Radiation exposure to others should always be As Low As Reasonably Achievable, a goal often abbreviated as ALARA. If you follow the above advice, the radiation from you to others is likely to be less than what they receive from radiation in nature over a year's time.
Please phone us if:

- you have any questions, and particularly if
- any of the above instructions cannot be followed and/or if
- you see anything that may have accidentally or unavoidably increased exposure of others to radiation.

19- Recommended Precautions after Lutathera treatments

		mCi (MBq) administered			
		Cycle 1	Cycle 2	Cycle 3	Cycle 4
		200 (7400)	200 (7400)	200 (7400)	200 (7400)
		Precaution Days			
Night-time restrictions					
	Sleep in a separate bedroom from adults for days shown	7	7	7	7
	Sleep in a separate bedroom from pregnant partners, infants, or children for days shown	15	15	15	15
Day-time restrictions					
	You may return to work after days shown	7	7	7	7
	Limit close contact (less than 1 meter) with children and pregnant women to less than 15 minutes per day for days shown.	7	7	7	7
	Avoid extended time in public places for days shown	7	7	7	7

Appendix 6 – Instructions for Shipment, Storage and Handling of ^{177}Lu-DOTA^{0}-Tyr^{3}-Octreotate (Lutathera) Solution for Infusion

NAME OF THE MEDICINAL PRODUCT

Lutathera 7400 MBq, radiopharmaceutical solution for infusion

QUALITATIVE AND QUANTITATIVE COMPOSITION

Lutathera is supplied as a sterile ready for use radiopharmaceutical solution for infusion.

A vial contains 7400 MBq of ^{177}Lu-DOTA^{0}-Tyr^{3}-Octreotate with a specific activity greater than 53 GBq/µmol and with a volumetric activity of 370 MBq/mL at reference date and time.

PHARMACEUTICAL FORM

Solution for infusion.
Clear, colorless to slightly yellow solution, free from visible particles.

PHARMACEUTICAL COMPONENTS

List of excipients
Acetic Acid
Sodium Acetate
Gentisic Acid
Ascorbic Acid
Pentetic acid (DTPA)
Sodium chloride
Sodium hydroxide
Water for Injections

Shelf life

72 hours after end of production.

Special precautions for storage

Store below 25°C.
Store in the original package to protect from ionizing radiation (lead shielding).
Storage of radiopharmaceuticals should be in accordance with national regulation on radioactive materials.

Nature and contents of container

Clear colourless Type I glass vial (30 ml vial), closed with a bromobutyl rubber stopper and aluminium seal.

Each vial contains a volume varying from 20 to 25 mL of solution corresponding to an activity of 7,400 MBq at date and time of infusion.

The vial is enclosed within a lead container for protective shielding. The secondary package complies with the requirements of a Type A package as described by the International Atomic Energy Agency (IAEA), International Air Transport Association (IATA) and International Carriage of Dangerous Goods by Road (ADR) dangerous goods regulations.

See below some pictures of example of packaging.

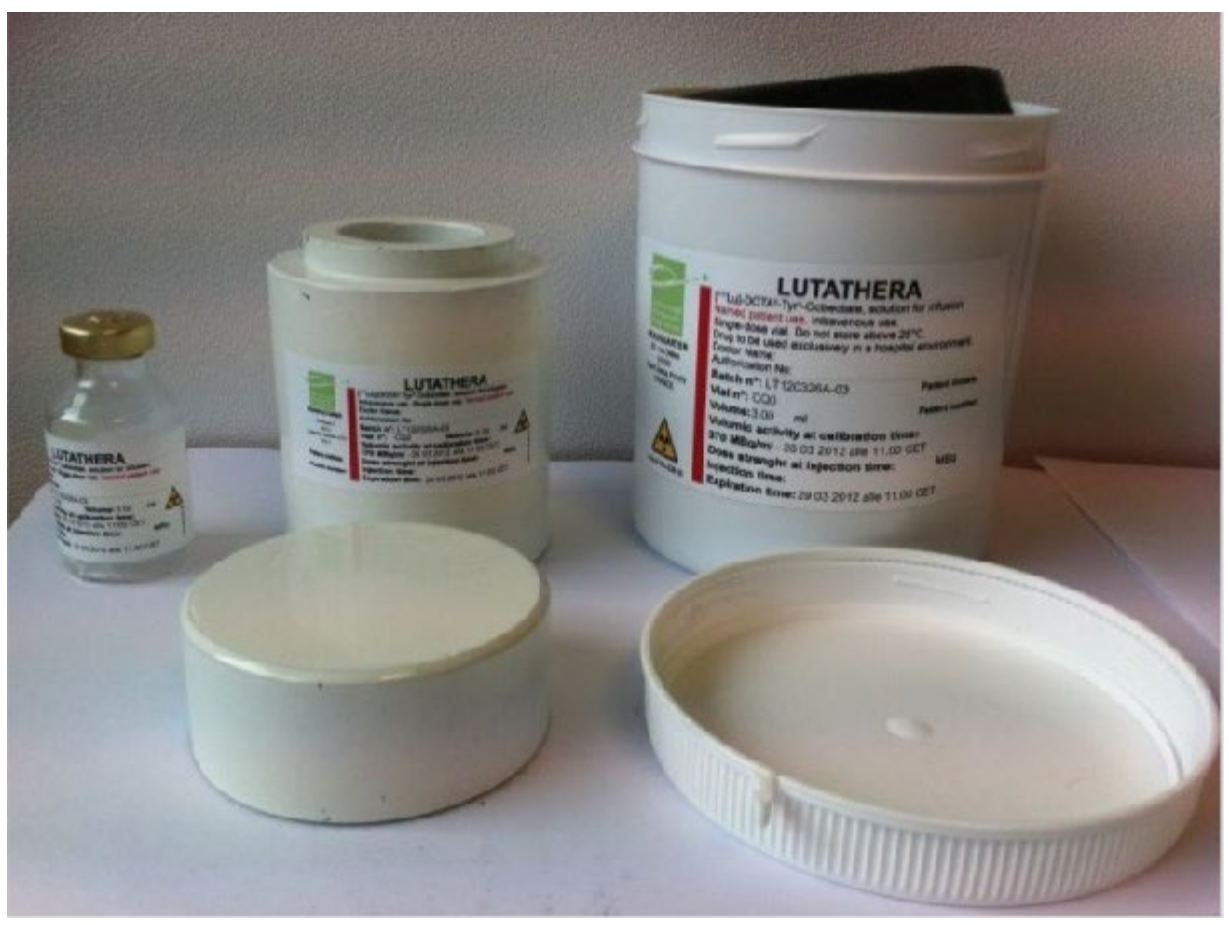

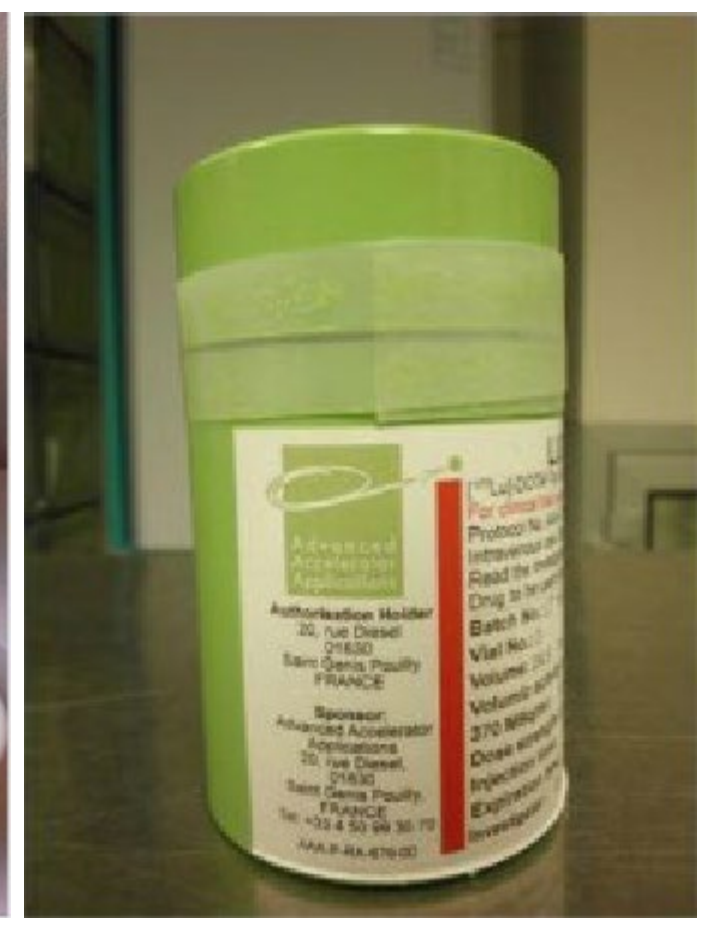

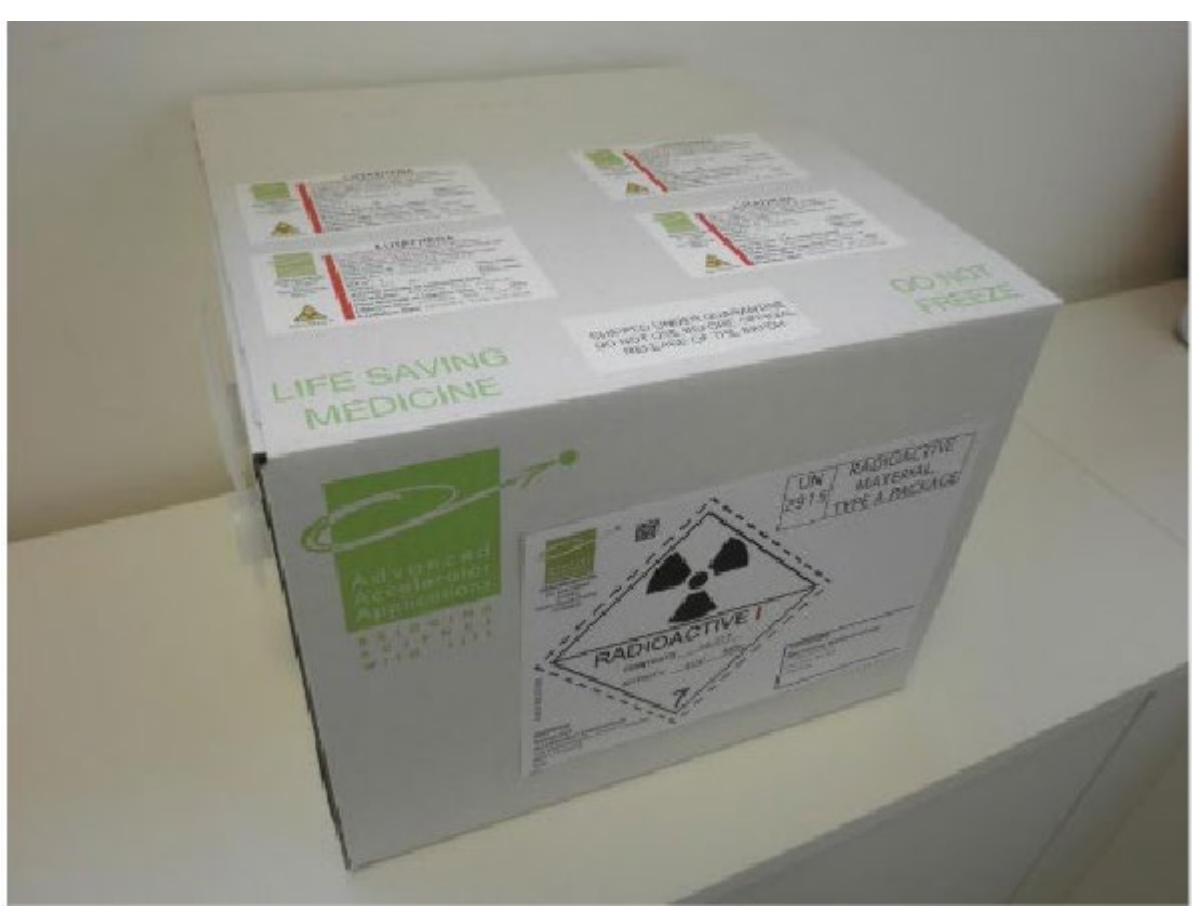

Primary and secondary packaging is labeled in compliance with annex 13 of Current GMP regulations. An example of label is shown below.

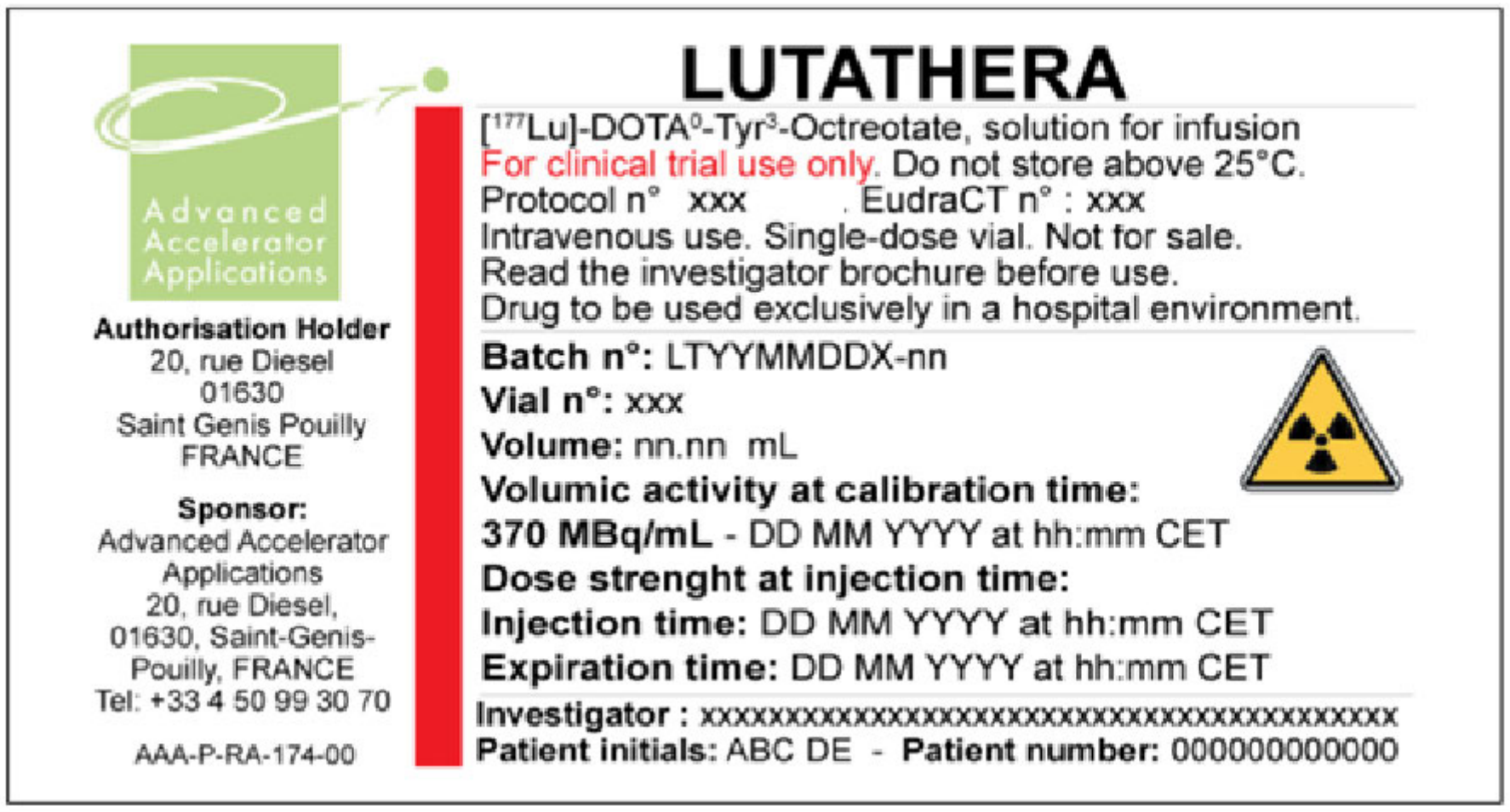

Special precautions for disposal and other handling

Radiopharmaceuticals should be received, used and administered only by authorized persons in designated clinical settings. Their receipt, storage, use, transfer and disposal are subject to the regulations and/or appropriate licenses of the competent official organization.

Radiopharmaceuticals should be prepared in a manner which satisfies both radiation safety and pharmaceutical quality requirements. Appropriate aseptic precautions should be taken.

If at any time in the preparation of this medicinal product the integrity of this container and vial is compromised it should not be used.

Administration procedures should be carried out in a way to minimize risk of contamination of the medicinal product and irradiation of the operators. Adequate shielding is mandatory.

It is necessary to wear waterproof gloves and suitable aseptic techniques when handling the medicinal product.

The administration of radiopharmaceuticals creates risks for other persons from external radiation or contamination from spill of urine, vomiting etc. Radiation protection precautions in accordance with national regulations must therefore be taken.

The surface dose rates and the accumulated dose depend on many factors. Measurements on the location and during work are critical and should be practiced for more precise and instructive determination of overall radiation dose to the staff. Healthcare personnel are advised to limit the time of close contact with patients injected with Lutathera. The use of television monitor systems to monitor the patients is recommended. Given the half life of 177Lu it is specially recommended to avoid internal contamination. It is necessary to use protective high quality (latex/nitrile) gloves to avoid direct contact with the radiopharmaceutical (vial/syringe). For minimizing radiation exposure, always use the principles of time, distance and shielding (reducing the manipulation of the vial and using the material already supplied par the manufacturer).

This preparation is likely to result in a relatively high radiation dose to most patients. The administration of 7,400 MBq may result in significant environmental hazard.

This may be of concern to the immediate family of those individuals undergoing treatment or the general public depending on the level of activity administered, hence radioprotection rules should be followed. Suitable precautions in accordance with national regulations should be taken concerning the activity eliminated by the patients in order to avoid any contaminations.

Any unused medicinal product or waste material should be disposed according to local requirements.

INSTRUCTIONS FOR THE USE OF Lutathera (7400 MBq)

Lutathera is for single use only.

The solution should be visually inspected for damage and contamination before use, and only clear solutions free of visible particles should be used. The visual inspection of the solution should be performed under a shielded screen for radioprotection purposes. The vial must not be opened.

If at any time in the preparation of this medicinal product the integrity of this vial is compromised, it should not be used.

The amount of radioactivity in the vial must be measured prior to infusion using a suitable radioactivity calibration system in order to confirm that the actual amount of radioactivity to be administered is equal to the planned amount at the infusion time.

Any unused medicinal product or waste material should be disposed of in accordance with local requirements. The activimeter or dose calibrator must be periodically calibrated by means of a standard source of Lu-177. If this procedure is not in place at the clinical site, Advanced Accelerator Applications will provide a calibration protocol and will ship a source of Lu-177 to perform the calibration if needed.

Lutathera does not need to be manipulated before administration because it is delivered as a ready to use monodose vial.

Lutathera must not be used after the expiry date which is stated on the label of the outer package.

Product receipt, tracking and administration data will be recorded using a informatics system provided by Advanced Accelerator Applications. In the event that the product is not administered for any reason, AAA will be notified, and a disposal verification record will be provided.

Quality Control of Lutathera

Minimum Quality Control is needed at the site, before the administration of Lutathera. The site will confirm that the product received has the correct release certificate. The results will be recorded. Additionally, the site’s measurement of total radioactivity and product administration data (start and stop time, and residual radioactivity not administered) will also be recorded.

Lutathera storage requirements

Lutathera must be stored at a temperature below 25°C (77° F), in its original package for radioprotection purposes, according to national regulations concerning radioactive products.

Shelf life: 72 hours after end of production.

Further information

The clinical site must request the required Lutathera dose for each patient at least 10 days before the scheduled treatment. A product order must be placed with AAA through the informatics system provided to each physician. The administration date of the product for each patient should not be confirmed until the clinical site has received both an order confirmation and a production dose confirmation from AAA. The scheduled date of treatment must be accepted by AAA so that the Manufacturer's Production Planning matches patient treatment schedules.

Appendix 7 – National Cancer Institute Common Terminology Criteria for Adverse Events

This is an extract of the whole document. For the complete CTCAE guide, version 5.0, please refer to the following website:
https://ctep.cancer.gov/protocoldevelopment/electronic_applications/docs/ctcae_v5_quick_reference_8.5x11.pdf

Blood and lymphatic system disorders					
	Grade				
**Adverse Event**	**1**	**2**	**3**	**4**	**5**
Anemia	Hemoglobin (Hgb) <LLN - 10.0 g/dL; <LLN - 6.2 mmol/L; <LLN - 100 g/L	Hgb <10.0 - 8.0 g/dL; <6.2 - 4.9 mmol/L; <100 - 80g/L	Hgb <8.0 g/dL; <4.9 mmol/L; <80 g/L; transfusion indicated	Life-threatening consequences; urgent intervention indicated	Death
Definition: A disorder characterized by an reduction in the amount of hemoglobin in 100 ml of blood. Signs and symptoms of anemia may include pallor of the skin and mucous membranes, shortness of breath, palpitations of the heart, soft systolic murmurs, lethargy, and fatigability.					
Bone marrow hypocellular	Mildly hypocellular or <=25% reduction from normal cellularity for age	Moderately hypocellular or >25 - <50% reduction from normal cellularity for age	Severely hypocellular or >50 - <=75% reduction cellularity from normal for age	Aplastic persistent for longer than 2 weeks	Death
Definition: A disorder characterized by the inability of the bone marrow to produce hematopoietic elements.					
Disseminated intravascular coagulation	-	Laboratory findings with no bleeding	Laboratory findings and bleeding	Life-threatening consequences; urgent intervention indicated	Death
Definition: A disorder characterized by systemic pathological activation of blood clotting mechanisms which results in clot formation throughout the body. There is an increase in the risk of hemorrhage as the body is depleted of platelets and coagulation factors.					
Febrile neutropenia	-	-	ANC <1000/mm3 with a single temperature of >38.3 degrees C (101 degrees F) or a sustained temperature of >=38 degrees C (100.4 degrees F) for more than one hour.	Life-threatening consequences; urgent intervention indicated	Death
Definition: A disorder characterized by an ANC <1000/mm3 and a single temperature of >38.3 degrees C (101 degrees F) or a sustained temperature of >=38 degrees C (100.4 degrees F) for more than one hour.					
Hemolysis	Laboratory evidence of hemolysis only (e.g., direct antiglobulin test; DAT; Coombs'; schistocytes; decreased haptoglobin)	Evidence of hemolysis and >=2 gm decrease in hemoglobin.	Transfusion or medical intervention indicated (e.g., steroids)	Life-threatening consequences; urgent intervention indicated	Death
Definition: A disorder characterized by laboratory test results that indicate widespread erythrocyte cell membrane destruction.					

Hemolytic uremic syndrome	Evidence of RBC destruction (schistocytosis) without clinical consequences	-	Laboratory findings with clinical consequences (e.g., renal insufficiency, petechiae)	Life-threatening consequences, (e.g., CNS hemorrhage or thrombosis/embolism or renal failure)	Death
Definition: A disorder characterized by a form of thrombotic microangiopathy with renal failure, hemolytic anemia, and severe thrombocytopenia.					
Leukocytosis	-	-	>100,000/mm3	Clinical manifestations of leucostasis; urgent intervention indicated	Death
Definition: A disorder characterized by laboratory test results that indicate an increased number of white blood cells in the blood.					
Lymph node pain	Mild pain	Moderate pain; limiting instrumental ADL	Severe pain; limiting self care ADL	-	-
Definition: A disorder characterized by a sensation of marked discomfort in a lymph node.					
Spleen disorder	Incidental findings (e.g., Howell-Jolly bodies); mild degree of thrombocytosis and leukocytosis	Prophylactic ant biotics indicated	-	Life-threatening consequences; urgent intervention indicated	Death
Definition: A disorder of the spleen.					
Thrombotic thrombocytopenic purpura	Evidence of RBC destruction (schistocytosis) without clinical consequences	-	Laboratory findings with clinical consequences (e.g., renal insufficiency, petechiae)	Life-threatening consequences, (e.g., CNS hemorrhage or thrombosis/embolism or renal failure)	Death
Definition: A disorder characterized by the presence of microangiopathic hemolytic anemia, thrombocytopenic purpura, fever, renal abnormalities and neurological abnormalities such as seizures, hemiplegia, and visual disturbances. It is an acute or subacute condition.					
Blood and lymphatic system disorders - Other, specify	Asymptomatic or mild symptoms; clinical or diagnostic observations only; intervention not indicated	Moderate; minimal, local or noninvasive intervention indicated; limiting age-appropriate instrumental ADL	Severe or medically significant but not immediately life-threatening; hospitalization or prolongation of existing hospitalization indicated; disabling; limiting self care ADL	Life-threatening consequences; urgent intervention indicated	Death

**Gastrointestinal disorders**					
	**Grade**				
**Adverse Event**	**1**	**2**	**3**	**4**	**5**
Diarrhea	Increase of <4 stools per day over baseline; mild increase in ostomy output compared to baseline	Increase of 4 - 6 stools per day over baseline; moderate increase in ostomy output compared to baseline	Increase of >=7 stools per day over baseline; incontinence; hospitalization indicated; severe increase in ostomy output compared to baseline; limiting self care ADL	Life-threatening consequences; urgent intervention indicated	Death
Definition: A disorder characterized by frequent and watery bowel movements.					

Dysphagia	Symptomatic, able to eat regular diet	Symptomatic and altered eating/swallowing	Severely altered eating/swallowing ; tube feeding or TPN or hospitalization indicated	Life-threatening consequences; urgent intervention indicated	Death
Definition: A disorder characterized by difficulty in swallowing.					
Esophagitis	Asymptomatic; clinical or diagnostic observations only; intervention not indicated	Symptomatic; altered eating/swallowing ; oral supplements indicated	Severely altered eating/swallowing ; tube feeding, TPN or hospitalization indicated	Life-threatening consequences; urgent operative intervention indicated	Death
Definition: A disorder characterized by inflammation of the esophageal wall.					
Gastric fistula	Asymptomatic; clinical or diagnostic observations only; intervention not indicated	Symptomatic; altered GI function	Severely altered GI function; bowel rest; tube feeding, TPN or hospitalization indicated; elective operative intervention indicated	Life-threatening consequences; urgent operative intervention indicated	Death
Definition: A disorder characterized by an abnormal communication between the stomach and another organ or anatomic site.					
Gastric ulcer	Asymptomatic; clinical or diagnostic observations only; intervention not indicated	Symptomatic; altered GI function; medical intervention indicated; limiting instrumental ADL	Severely altered GI function; TPN indicated; elective operative or endoscopic intervention indicated; limiting self care ADL; disabling	Life-threatening consequences; urgent operative intervention indicated	Death
Definition: A disorder characterized by a circumscr bed, inflammatory and necrotic erosive lesion on the mucosal surface of the stomach.					
Gastritis	Asymptomatic; clinical or diagnostic observations only; intervention not indicated	Symptomatic; altered GI function; medical intervention indicated	Severely altered eating or gastric function; TPN or hospitalization indicated	Life-threatening consequences; urgent operative intervention indicated	Death
Definition: A disorder characterized by inflammation of the stomach.					
Mucositis oral	Asymptomatic or mild symptoms; intervention not indicated	Moderate pain; not interfering with oral intake; modified diet indicated	Severe pain; interfering with oral intake	Life-threatening consequences; urgent intervention indicated	Death
Definition: A disorder characterized by inflammation of the oral mucosal.					
Nausea	Loss of appetite without alteration in eating habits	Oral intake decreased without significant weight loss, dehydration or malnutrition	Inadequate oral caloric or fluid intake; tube feeding, TPN, or hospitalization indicated	-	-
Definition: A disorder characterized by a queasy sensation and/or the urge to vomit.					
Small intestinal obstruction	Asymptomatic; clinical or diagnostic observations only; intervention not indicated	Symptomatic; altered GI function; limiting instrumental ADL	Hospitalization indicated; elective operative intervention indicated; limiting self care ADL; disabling	Life-threatening consequences; urgent operative intervention indicated	Death
Definition: A disorder characterized by blockage of the normal flow of the intestinal contents.					

Upper gastrointestinal hemorrhage	Mild; intervention not indicated	Moderate symptoms; medical intervention or minor cauterization indicated	Transfusion, radiologic, endoscopic, or elective operative intervention indicated	Life-threatening consequences; urgent intervention indicated	Death
Definition: A disorder characterized by bleeding from the upper gastrointestinal tract (oral cavity, pharynx, esophagus, and stomach).					
Vomiting	1 - 2 episodes (separated by 5 minutes) in 24 hrs	3 - 5 episodes (separated by 5 minutes) in 24 hrs	>=6 episodes (separated by 5 minutes) in 24 hrs; tube feeding, TPN or hospitalization indicated	Life-threatening consequences; urgent intervention indicated	Death
Definition: A disorder characterized by the reflexive act of ejecting the contents of the stomach through the mouth.					
Gastrointestinal disorders - Other, specify	Asymptomatic or mild symptoms; clinical or diagnostic observations only; intervention not indicated	Moderate; minimal, local or noninvasive intervention indicated; limiting age-appropriate instrumental ADL	Severe or medically significant but not immediately life-threatening; hospitalization or prolongation of existing hospitalization indicated; disabling; limiting self care ADL	Life-threatening consequences; urgent intervention indicated	Death
**Infections and infestations**					
	**Grade**				
**Adverse Event**	**1**	**2**	**3**	**4**	**5**
Infections and infestations - Other, specify	Asymptomatic or mild symptoms; clinical or diagnostic observations only; intervention not indicated	Moderate; minimal, local or noninvasive intervention indicated; limiting age-appropriate instrumental ADL	Severe or medically significant but not immediately life-threatening; hospitalization or prolongation of existing hospitalization indicated; disabling; limiting self care ADL	Life-threatening consequences; urgent intervention indicated	Death
**Investigations**					
	**Grade**				
**Adverse Event**	**1**	**2**	**3**	**4**	**5**
Alanine aminotransferase increased	>ULN - 3.0 x ULN	>3.0 - 5.0 x ULN	>5.0 - 20.0 x ULN	>20.0 x ULN	-
Definition: A finding based on laboratory test results that indicate an increase in the level of alanine aminotransferase (ALT or SGPT) in the blood specimen.					
Alkaline phosphatase increased	>ULN - 2.5 x ULN	>2.5 - 5.0 x ULN	>5.0 - 20.0 x ULN	>20.0 x ULN	-
Definition: A finding based on laboratory test results that indicate an increase in the level of alkaline phosphatase in a blood specimen.					
Aspartate aminotransferase increased	>ULN - 3.0 x ULN	>3.0 - 5.0 x ULN	>5.0 - 20.0 x ULN	>20.0 x ULN	-
Definition: A finding based on laboratory test results that indicate an increase in the level of aspartate aminotransferase (AST or SGOT) in a blood specimen.					
Blood bilirubin increased	>ULN - 1.5 x ULN	>1.5 - 3.0 x ULN	>3.0 - 10.0 x ULN	>10.0 x ULN	-
Definition: A finding based on laboratory test results that indicate an abnormally high level of bilirubin in the blood. Excess bilirubin is associated with jaundice.					
Lipase increased	>ULN- 1.5 x ULN	>1.5 - 2.0 x ULN	>2.0 - 5.0 x ULN	>5.0 x ULN	-
Definition: A finding based on laboratory test results that indicate an increase in the level of lipase in a biological specimen.					

Lymphocyte count decreased	<LLN - 800/mm3; <LLN - 0.8 x 10e9 /L	<800 - 500/mm3; <800 - 0.5 x 10e9 /L	<500 - 200/mm3; <0.5 - 0.2 x 10e9 /L	<200/mm3; <0.2 x 10e9 /L	-
Definition: A finding based on laboratory test results that indicate a decrease in number of lymphocytes in a blood specimen.					
Lymphocyte count increase	-	>4000/mm3 – 20,000/mm3 10e9 /L	>20,000/mm3	-	-
Definition: A finding based on laboratory test results that indicate an abnormal increase in the number of lymphocytes in the blood,					
Neutrophil count decreased	<LLN - 1500/mm3; <LLN - 1.5 x 10e9 /L	<1500 - 1000/mm3; <1.5 - 1.0 x 10e9 /L	<1000 - 500/mm3; <1.0 - 0.5 x 10e9 /L	<500/mm3; <0.5 x 10e9 /L	-
Definition: A finding based on laboratory test results that indicate a decrease in number of neutrophils in a blood specimen.					
Pancreatic enzymes decreased	<LLN and asymptomatic	Increase in stool frequency, Bulk, or odor, steatorrhea	Sequelae of absorption deficiency	-	-
Definition: A finding based on laboratory test results that indicate an decrease in levels of pancreatic enzymes in a biological specimen.					
Platelet count decreased	<LLN - 75,000/mm3; <LLN - 75.0 x 10e9 /L	<75,000 - 50,000/mm3; <75.0 - 50.0 x 10e9 /L	<50,000 - 25,000/mm3; <50.0 - 25.0 x 10e9 /L	<25,000/mm3; <25.0 x 10e9/L	-
Definition: A finding based on laboratory test results that indicate a decrease in number of platelets in a blood specimen.					
Serum amylase increased	>ULN - 1.5 x ULN	>1.5 - 2.0 x ULN	>2.0 - 5.0 x ULN	>5.0 x ULN	-
Definition: A finding based on laboratory test results that indicate an increase in the levels of amylase in a serum specimen.					
Urine output decreased	-	-	Oliguria (<80 ml in 8 hr)	Anuria (<240 ml in 24 hr)	-
Definition: A finding based on test results that indicate urine production is less relative to previous output.					
Vital capacity abnormal	90 - 75% of predicted value	<75 - 50% of predicted value; limiting instrumental ADL	<50% of predicted value; Limiting self care ADL	-	-
Definition: A finding based on pulmonary function test results that indicate an abnormal vital capacity (amount of exhaled after a maximum inhalation) when compared to the predicted value.					
Weight gain	5 - < 10% from baseline	10 - <20% from baseline	<=20% from baseline	-	-
Definition: A finding characterized by an increase in overall body weight; for pediatrics, greater than the baseline growth curve.					
Weight loss	5 to < 10% from baseline; Intervention not indicated	10 - <20% from baseline; nutritional support indicated	>=20% from baseline; tube Feeding or TPN indicated	-	-
Definition: A finding characterized by a decrease in overall body weight; for pediatrics, less than the baseline growth curve.					
White blood cell decreased	<LLN - 3000/mm3; <LLN - 3.0 x 10e9 /L	<3000 - 2000/mm3; <3.0 - 2.0 x 10e9 /L	<2000 - 1000/mm3; <2.0 - 1.0 x 10e9 /L	<1000/mm3; <1.0 x 10e9 /L	-
Definition: A finding based on laboratory test results that indicate an decrease in number of white blood cells in a blood specimen.					
Investigations - Other, specify	Asymptomatic or mild symptoms; clinical or diagnostic observations only; intervention not indicated	Moderate; minimal, local or noninvasive intervention indicated; limiting age-appropriate instrumental ADL	Severe or medically significant but not immediately life-threatening; hospitalization or prolongation of existing hospitalization indicated; disabling; limiting self care ADL	Life-threatening consequences; urgent intervention indicated	Death
**Metabolism and nutrition disorders**					
	**Grade**				
**Adverse Event**	**1**	**2**	**3**	**4**	**5**

Anorexia	Loss of appetite without alteration in eating habits	Oral intake altered without significant weight loss or malnutrition; oral nutritional supplements indicated	Associated with significant weight loss or malnutrition (e.g., inadequate oral caloric and/or fluid intake); tube feeding or TPN indicated	Life-threatening consequences; urgent intervention indicated	Death
Definition: A disorder characterized by a loss of appetite.					
Tumor lysis syndrome	-	-	Present	Life-threatening consequences; urgent intervention indicated	Death
Definition: A disorder characterized by metabolic abnormalities that result from a spontaneous or therapy-related cytolysis of tumor cells.					
Metabolism and nutrition disorders - Other, specify	Asymptomatic or mild symptoms; clinical or diagnostic observations only; intervention not indicated	Moderate; minimal, local or noninvasive intervention indicated; limiting age-appropriate instrumental ADL	Severe or medically significant but not immediately life-threatening; hospitalization or prolongation of existing hospitalization indicated; disabling; limiting self care ADL	Life-threatening consequences; urgent intervention indicated	Death

Common Terminology Criteria for Adverse Events (CTCAE) v5.0

Publish Date: November 27, 2017

Introduction

The NCI Common Terminology Criteria for Adverse Events is a descriptive terminology which can be utilized for Adverse Event (AE) reporting. A grading (severity) scale is provided for each AE term.

SOC

System Organ Class (SOC), the highest level of the MedDRA[1] hierarchy, is identified by anatomical or physiological system, etiology, or purpose (e.g., SOC Investigations for laboratory test results). CTCAE terms are grouped by MedDRA Primary SOCs. Within each SOC, AEs are listed and accompanied by descriptions of severity (Grade).

CTCAE Terms

An Adverse Event (AE) is any unfavorable and unintended sign (including an abnormal laboratory finding), symptom, or disease temporally associated with the use of a medical treatment or procedure that may or may not be considered related to the medical treatment or procedure. An AE is a term that is a unique representation of a specific event used for medical documentation and scientific analyses. Each CTCAE v4.0 term is a MedDRA LLT (Lowest Level Term).

Grades

Grade refers to the severity of the AE. The CTCAE displays Grades 1 through 5 with unique clinical descriptions of severity for each AE based on this general guideline:

Grade 1 Mild; asymptomatic or mild symptoms; clinical or diagnostic observations only; intervention not indicated.
Grade 2 Moderate; minimal, local or noninvasive intervention indicated; limiting age-appropriate instrumental ADL*.
Grade 3 Severe or medically significant but not immediately life-threatening; hospitalization or prolongation of hospitalization indicated; disabling; limiting self care ADL**.
Grade 4 Life-threatening consequences; urgent intervention indicated.
Grade 5 Death related to AE.

A Semi-colon indicates 'or' within the description of the grade.

A single dash (-) indicates a Grade is not available. Not all Grades are appropriate for all AEs. Therefore, some AEs are listed with fewer than five options for Grade selection.

Grade 5

Grade 5 (Death) is not appropriate for some AEs and therefore is not an option.

Definitions

A brief Definition is provided to clarify the meaning of each AE term. A single dash (-) indicates a Definition is not available.

Navigational Notes

A Navigational Note is used to assist the reporter in choosing a correct AE. It may list other AEs that should be considered in addition to or in place of the AE in question. A single dash (-) indicates a Navigational Note has not been defined for the AE term.

Activities of Daily Living (ADL)

*Instrumental ADL refer to preparing meals, shopping for groceries or clothes, using the telephone, managing money, etc.
**Self care ADL refer to bathing, dressing and undressing, feeding self, using the toilet, taking medications, and not bedridden.

Appendix 8 – Determination of LUTATHERA Administered Radioactivity

The total amount of radioactivity administered to the patient is determined by measuring the radioactivity present in the LUTATHERA (^{177}Lu-DOTA^{0}-Tyr^{3}-Octreotate, Infusion Solution) Vial before and after administration. The procedure is as follows:

A. Measurement of LUTATHERA Vial radioactivity *before* LUTATHERA **administration:**

1) remove the LUTATHERA Vial from the original package;
2) place the LUTATHERA Vial in the dose calibrator (activimeter) chamber;
3) select the Lu-177 setting on the dose calibrator;
4) press the "measure" button (or equivalent function button);
5) record the measured radioactivity, and the time of measurement in the appropriate electronic Case Report Form (eCRF);
6) remove the LUTATHERA Vial from the dose calibrator chamber;
7) return the LUTATHERA Vial to its original package or prepare the Vial for patient administration.

B. Measurement of LUTATHERA vial radioactivity *after* LUTATHERA **administration:**

1) remove needles and tubing attached to the LUTATHERA Vial (NB: The investigator must confirm that there is no significant residual radioactivity remaining in delivery needles and tubing after administration (< 0.1% of original Total Radioactivity in LUTATHERA Vial);
2) if the dose calibrator is in a different location, place the LUTATHERA Vial in the original package so that the material can be moved safely – otherwise go to step 4;
3) remove the LUTATHERA Vial from the original package;
4) place the LUTATHERA Vial in the dose calibrator chamber;
5) select the Lu-177 setting on the dose calibrator;
6) press the "measure" button (or equivalent function button);
7) record the measured radioactivity, and the time of measurement in the appropriate eCRF;
8) remove the LUTATHERA Vial from the dose calibrator chamber and dispose according to national and local regulations for disposal of radioactive waste;

C. Calculation of Total Administered and Residual Radioactivity:

1) Total Administered Radioactivity

   = (amount of radioactivity in LUTATHERA Vial before administration minus amount of radioactivity in LUTATHERA Vial after administration) times vial geometry correction factor

   $= (A.5 - B.7)\ x\ Correction\ Factor;$

2) Residual Radioactivity = amount of radioactivity remaining in LUTATHERA Vial after administration times vial geometry correction factor

   $= B.7\ x\ Correction\ Factor;$

3) record Total Administered Radioactivity and Residual Radioactivity in the appropriate sections of the patient's eCRF.
