# Supplementary material for: [177Lu]Lu-DOTA-TATE plus long-acting octreotide in patients with newly diagnosed, advanced, grade 2–3, gastroenteropancreatic neuroendocrine tumours: preplanned and post-hoc efficacy analyses from the randomised, phase 3 NETTER-2 trial
Source: eClinicalMedicine. 2026 Aug 7;98:104109. doi: 10.1016/j.eclinm.2026.104109 (PMC13476574; doi:10.1016/j.eclinm.2026.104109)
Supplement: Supplementary file 2 [file mmc2.pdf]

SUPPLEMENTARY APPENDIX

[^{177}Lu]Lu-DOTA-TATE plus long-acting octreotide in patients with newly diagnosed, advanced, grade 2–3, gastroenteropancreatic neuroendocrine tumours: preplanned and post-hoc efficacy analyses from the randomised, phase 3 NETTER-2 trial

Diego Ferone, Marianne Pavel, Ken Herrmann, Pamela L Kunz, Sten Myrehaug, Daniel Halperin, Beth Chasen, Jaume Capdevila, Amparo García-Burillo, Salvatore Tafuto, Secondo Lastoria, Do-Youn Oh, Changhoon Yoo, Stephen Falk, Thorvardur R Halfdanarson, Maddalena Sansovini, Laura Gerard, Emmanuel Deshayes, Angelina Filice, Ilya Folitar, Yufen Zhang, Wouter W de Herder, Simron Singh

Correspondence to: Dr Simron Singh, University of Toronto, Sunnybrook Odette Cancer Center, 2075 Bayview Avenue, Toronto, ON M4N 3M5, Canada
simron.singh@sunnybrook.ca

CONTENTS

***Figure S1*: Patient flow chart** 2
***Table S1:* Ethics approvals** 3
***Table S2:* Progression-free survival (post-hoc subgroup analysis)** 4
***Table S3:* Objective response rate (post-hoc subgroup analysis)** 5

Figure S1: **Patient flow chart**

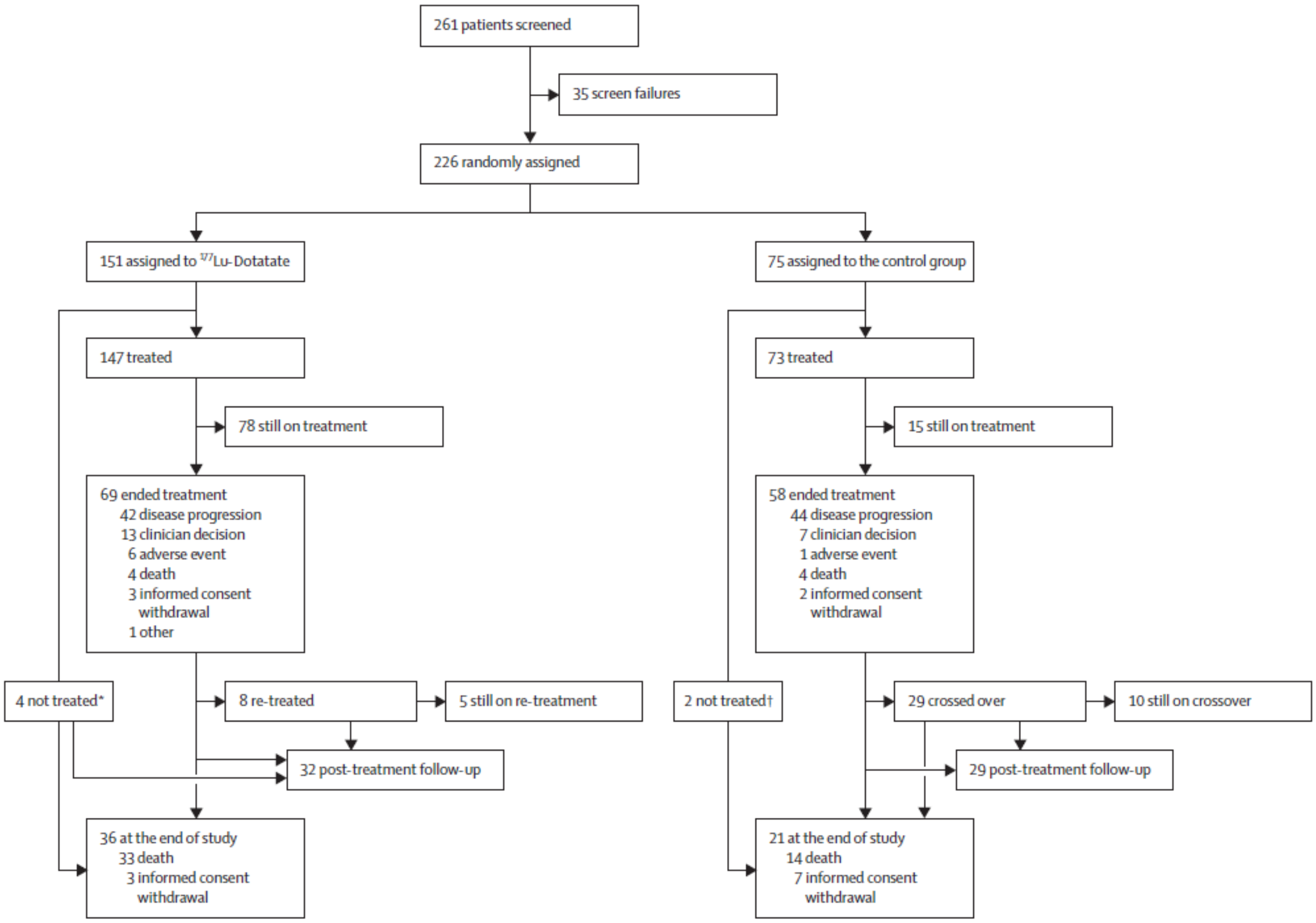

The figure patient flow for NETTER-2. ^{177}Lu-Dotatate=[^{177}Lu]Lu-DOTA-TATE. *Reasons for not being treated were surgery before first treatment (n=1), randomisation mistake (n=1), informed consent withdrawal (n=1), and adverse event (n=1). †Reasons for not being treated were surgery before first treatment (n=1) and informed consent withdrawal (n=1).

Originally published in: Singh S, Halperin D, Myrehaug S, et al. [^{177}Lu]Lu-DOTA-TATE plus long-acting octreotide versus high-dose long-acting octreotide for the treatment of newly diagnosed, advanced grade 2–3, well-differentiated, gastroenteropancreatic neuroendocrine tumours (NETTER-2): an open-label, randomised, phase 3 study. Lancet 2024; 403: 2807–17.

Table S1: **Ethics approvals**

Country	Name of EC/IRB	Reference number
Canada	OCREB IRB	IRB 00003960
	Comité d'éthique la Recherche Chu de Québec Université LavL	FWA00000329 and IRB00001242
	UBC BC Cancer Research Ethics Board	IRB 00003797
France	Comité de Protection des Personnes (IEC/IRB) Sud-Méditerranée I	EU-CT: 2023-507443-10-00
Germany	Ethikkommission der Medizinischen Fakultät der Friedrich-Alexander-Universität Erlangen-Nürnberg	EU-CT: 2023-507443-10-00
Italy	Comitato Etico Campania 1	EU-CT: 2023-507443-10-00
S. Korea	Institutional Review Board of Severance Hospital, Yonsei University Health System	4-2019-1103
	Asan Medical Center IRB	2019-1710
	Seoul National University Hospital – IRB	H-1911-148-1083
	The Institutional Review Board of Seoul National University Bundang Hospital	B-2002-592-406
Netherlands	Netherlands METC Erasmus MC	EU-CT: 2023-507443-10-00
Spain	CEIm Hospital General Universitario Gregorio Marañón	EU-CT: 2023-507443-10-00
UK	London – City and East Research Ethics Committee	REC reference: 19/LO/1551<br>IRAS project ID: 269540
US	Advarra	IRB#: 00000971
	Mayo Clinic	IRB#: 20-001356
	The University of Texas MD Anderson Cancer Center	IRB#: 00000121
	The University of Iowa	IRB#: 202004133
	WCG IRB	IRB#: 20213282

The table shows the name of the EC/IRB(s) that approved the trial protocol (and the corresponding reference number[s]) for participating countries. EC=Ethics Committee. IRB=Institutional Review Board.

Table S2: **Progression-free survival (post-hoc subgroup analysis)**

	Event/N (%)	Median progression-free survival (95% CI), months	HR (95% CI)
**Grade 2 pancreatic NET subgroup**			
^{177}Lu-DOTATATE	20/50 (40·0)	22·8 (16·6–NE)	0·364
Control	14/26 (53·8)	13·8 (7·7–19·4)	(0·178–0·742)
**Grade 2 gastrointestinal NET subgroup**			
^{177}Lu-DOTATATE	9/49 (18·4)	NE (22·6–NE)	0·217
Control	11/22 (50·0)	13·8 (8·0–NE)	(0·087–0·543)
**Grade 3 pancreatic NET subgroup**			
^{177}Lu-DOTATATE	19/32 (59·4)	14·7 (10·8–24·9)	0·278
Control	13/15 (86·7)	5·6 (2·1–9·1)	(0·130–0·592)
**Grade 3 gastrointestinal NET subgroup**			
^{177}Lu-DOTATATE	7/20 (35·0)	NE (8·4–NE)	0·232
Control	8/12 (66·7)	5·6 (2·8–NE)	(0·082–0·662)

The table shows progression-free survival for patients in the full analysis set (based on central review using Response Evaluation Criteria in Solid Tumours, version 1.1 criteria) by subgroups of interest. The median progression-free survival and corresponding 95% CIs were estimated using the Kaplan-Meier method. HRs and corresponding 95% CIs are for the ^{177}Lu-DOTATATE arm versus the control arm and were based on an unstratified Cox model. CI=confidence interval. HR=hazard ratio. NET=neuroendocrine tumour. NE=not estimable.

Table S3: **Objective response rate (post-hoc subgroup analysis)**

	Complete response, n (%)	Partial response, n (%)	Total responders/N	Objective response rate (95% CI)	OR (95% CI)
**Grade 2 pancreatic NET subgroup**					
^{177}Lu-DOTATATE	4 (8·0)	21 (42·0)	25/50	50·0 (35·5–64·5)	5·50
Control	0	4 (15·4)	4/26	15·4 (4·4–34·9)	(1·65–18·28)
**Grade 2 gastrointestinal NET subgroup**					
^{177}Lu-DOTATATE	2 (4·1)	13 (26·5)	15/49	30·6 (18·3–45·4)	9·26
Control	0	1 (4·5)	1/22	4.5 (0·1–22·8)	(1·14–75·36)
**Grade 3 pancreatic NET subgroup**					
^{177}Lu-DOTATATE	1 (3·1)	16 (50·0)	17/32	53·1 (34·7–70·9)	15·87
Control	0	1 (6·7)	1/15	6·7 (0·2–31·9)	(1·86–135·44)
**Grade 3 gastrointestinal NET subgroup**					
^{177}Lu-DOTATATE	1 (5·0)	7 (35·0)	8/20	40·0 (19·1–63·9)	7·33
Control	0	1 (8·3)	1/12	8·3 (0·2–38·5)	(0·79–68·48)

The table shows best overall response for patients in the full analysis set (based on central review using Response Evaluation Criteria in Solid Tumours, version 1.1 criteria) by subgroups of interest. For objective response rate, two-sided exact 95% CIs are presented. ORs are for the ^{177}Lu-DOTATATE arm versus the control arm and were based on unstratified Cochran-Mantel-Haenszel method. CI=confidence interval. NET=neuroendocrine tumour. OR=odds ratio.
