# Supplementary material for: [177Lu]Lu-DOTA-TATE plus long-acting octreotide in patients with newly diagnosed, advanced, grade 2–3, gastroenteropancreatic neuroendocrine tumours: preplanned and post-hoc efficacy analyses from the randomised, phase 3 NETTER-2 trial
Source: eClinicalMedicine. 2026 Aug 7;98:104109. doi: 10.1016/j.eclinm.2026.104109 (PMC13476574; doi:10.1016/j.eclinm.2026.104109)
Supplement: Supplementary file 3 [file mmc3.pdf]

Clinical Development

LUTATHERA®

Study number CAAA601A22301 (NETTER-2)

A phase III multi-center, randomized, open-label study to evaluate the efficacy and safety of Lutathera in patients with Grade 2 and Grade 3 advanced GEP-NET

Statistical Analysis Plan (SAP) – Amendment 2

Author:	Trial Statistician [redacted]
Document type:	SAP Documentation
Document status:	Version 2.0
Release date:	22-Aug-2023
Number of pages:	82

Property of Advanced Accelerator Applications, a Novartis company
Confidential
May not be used, divulged, published or otherwise disclosed
without the consent of Advanced Accelerator Applications,a Novartis company

Document History – Changes compared to previous final version of SAP

Date	Time point	Reason for update	Outcome for update	Section and title impacted (Current)
30-Jun-2020	Prior to DB lock	Creation of final version	N/A – First version	NA
18-Nov-2022	Prior to DB lock	Incorporating amendments 1 and 2 of protocol	Update roles and participants for signatures	Signature Page
			Addition of reference to Amendments 1 and 2 of protocol	1. Introduction
			Addition of reference to optional re-treatment phase	1.1 Study Design
			Update study design figure	
			Minor editorial updates for the endpoints	1.2 Study objectives and endpoints
			Addition of re-treatment exploratory endpoints	
			Addition/update of general definitions to account for the addition of re-treatment and to clarify some definitions	2.1.1 General definitions updates
			Update of Table 2.2.1	2.2 Analysis Sets
			Addition of appropriate summaries and listings for patients who crossover or receive re-treatment therapy separately	2.3 Patient disposition, demographics and other baseline characteristics
			Addition of appropriate summaries and listings for the extension and re-treatment phases	2.4.1 Study treatment / compliance
			Addition of appropriate summaries and listings for the extension and re-treatment phases	2.4.2 Prior, concomitant and post therapies
			Addition of more details to improve clarity on estimation of PFS at the primary analysis	2.5.1 Primary endpoint
			Addition of more details on censoring and progression rules for PFS	2.5.3 Handling of missing values/censoring/discontinuation
			Updated Table 2.5-1	
			Addition of supportive analyses to support estimand analysis	2.5.6 Supportive analyses
			Highlighting that COVID-19 sensitivity analysis will be discussed in SAP	
			Addition of the 24 week requirement for SD	2.7.1 Other secondary and exploratory endpoints
			Addition of exploratory PFS, ORR, DCR, DoR, PFS2 and OS analysis in re-treatment Lutathera patients sub-section	

Date	Time point	Reason for update	Outcome for update	Section and title impacted (Current)
			Moved Table 2.7-1 to Section 2.7.3	
			Removed censoring language for DOR since that is mentioned in 2.7.1	2.7.2 Statistical hypothesis, model, and method of analysis
			Highlighted that all efficacy analysis for re-treatment phase is descriptive	
			Added PFS2 censoring rules<br>Added statement on OS censoring	2.7.3 Handling of missing values/censoring/discontinuation
			Highlighted throughout section that safety analyses will be conducted for the on-treatment period, crossover period, and re-treatment period separately	2.8 Safety analyses
			Highlighted that for AE summaries, SOCs will be collected alphabetically.	2.8.1 Adverse events (AEs)
			Stated that all AE groupings for a clinical program are stored in CRS	2.8.1.2 Adverse events of special interest/ grouping of AEs
			Added details about potential Hy's law events	2.8.3 Laboratory data
			Highlighted that if an ECG is conducted, but data is not available in triplicate, the average of the ECG parameters at that assessment should be used in the analysis.	2.8.4.1 ECG and cardiac imaging data
			Added analyses to support dosimetry collection in Germany	2.10 Dosimetry analysis
			Moved duration of follow-up analysis to this section	2.12 Duration of follow-up
			Separated follow-up time for PFS and OS	
			For PFS follow-up, highlighted that this is for 1st central progression	
			Replaced dose with treatment throughout section	5.1.1 Study treatment
			Event end date only applicable for AE and CM datasets in Table 5.1-1	5.1.2 AE, ConMeds and safety assessment date imputation
			Clarified Table 5.1-2 to capture end date of final treatment on trial	
			Imputation used to determine TEAEs	
			Removed project specific ranges language	5.3 Laboratory parameters derivations
			Added information on missing scan capturing	5.5 Determination of missing adequate assessments
22-Aug-2023	Prior to DB lock	To align on more details of analysis rules before DBL	• Added 'unconfirmed' for CR and PR.<br>• Removed PD, disposition by center summary<br>• Clarified baseline for cross-over/re-treatment.<br>• Updated terms for study phase	1.3.2 Secondary estimand #1<br>2.1 Data analysis general information<br>2.1.1.8 Baseline<br>2.1.1.9 Definition of study phases

Date	Time point	Reason for update	Outcome for update	Section and title impacted (Current)
			• Updated section title from 'On-treatment assessment/event' to 'Definition of treatment periods used in analyses'; updated definitions for different treatment periods; added definition for between treatment period; clarified scope of cross-over/re-treatment analyses at the time of primary PFS analysis.	2.1.1.10 Definition of treatment periods used in analyses
			• Added analysis window table for PRO data; removed wordings that are not applicable for this study; updated source for last contact date.	2.1.1.11 Windows for multiple assessments.
			• Updated subgroups based on clinical input.	2.2.3 Subgroup of interest
			• Added Table 2-3 for grouping of primary sites of cancer; removed baseline characteristics summary for cross-over/re-treatment at the time of primary PFS analysis; removed major protocol deviation leading to exclusion from analysis, which is not applicable for this study.	2.3 Patient disposition, demographics and other baseline characteristics
			• Adjusted exposure summaries for study treatment based on actual data in this study; added clarification for PDI of Octreotide LAR	2.4 Treatment (study treatment, rescue medication, concomitant therapies, compliance)
			• Adjusted summaries for prior anti-cancer therapy; also removed redundant wordings that are not applicable to this study or for the primary PFS analysis.	2.4.2 Prior, concomitant and post therapies.
			• Removed censoring rule for supplementary analysis from Table 2.5-1, which is already covered later.	2.5.4 Handling of missing values/censoring/discontinuations
			• Removed repeated paragraph; shifted PFS analysis based on local assessment to 2.5.6 supplementary analysis; adjusted variables to be considered in stratified multivariate Cox model; removed unstratified multivariate Cox model from the sensitivity analyses.	2.5.5 Sensitivity analyses
			• Clarified detailed PFS analyses for subgroups; fixed derivation logic for PFS censoring reasons; added rules for waterfall plot.	2.5.6 Supplementary analyses

Date	Time point	Reason for update	Outcome for update	Section and title impacted (Current)
			• Added ‘unconfirmed’ before PR and CR; shifted EORTC QLQ-G.I.NET21 from this section to 2.7.1	2.6.1 Key secondary endpoints
			• Updated handling of discontinuation of study treatment for QoL relevant endpoints from Treatment policy strategy to While-on-treatment strategy, with the fact that measurements of QoL are planned for the randomized treatment period only per protocol.	2.6.3 Handing of intercurrent events of secondary estimands
			• Clarified censoring rule for 2 or more missing assessments in the key secondary endpoints of QoL.	2.6.4 Handling of missing values/censoring/discontinuations
			• Removed 2 supplementary analyses for ORR; added concordance summary for BOR based on central vs. local; clarified reasons for BOR as unknown; Clarified detailed ORR/TTD analyses for subgroups; removed forest plot for ORR.	2.6.6 Supplementary analyses
			• Clarified PD window as 25 weeks (i.e., first 2 efficacy assessments +1wk) in DCR and ORR calculation; Clarified analysis for DOR; added censoring reasons for OS; updated PFS2 rules and clarified analysis plan; shifted EORTC QLQ-GI.NET 21 to this section instead of the original section for key secondary endpoints; removed OS sensitivity and exploratory analysis.	2.7.1 Other secondary and exploratory endpoints
			• Added ADAE definition and clarified the main AE summaries in this study; Removed paragraphs about AE occurrence summary, which is not planned for CSR.	2.8.1 Adverse events (AEs)
			• Removed exposure adjusted AE analysis	2.8.1.1 (old) AE adjusted for subject exposure time
			• Updated AESI grouping per latest team discussion and alignment. Added definition of time to first occurrence of AESI in the randomized treatment period vs. during the study.	2.8.1.1 Adverse events of special interest / grouping of AEs
			• Updated death summary per collected data	2.8.2 Deaths
			• Removed time course plot for hepatic injury and eDISH plot.	2.8.3 Laboratory data

Date	Time point	Reason for update	Outcome for update	Section and title impacted (Current)
			• Clarified analysis for ECG data; removed change from baseline analysis.	2.8.4.1 ECG data
			• Removed change from baseline analysis for vital signs.	2.8.4.2 Vital signs
			• Removed summary for TTD follow-up time	2.12 Duration of follow-up
			• Updated imputation rules for start/end dates for AE, ConMeds and other safety assessments.	5.1.2 AE, ConMeds and safety assessment date imputation
			• Added Cockroft-Gault formula for calculating creatine clearance based on serum creatine; Added tables for Novartis internal criteria for CTCAE grading of lab parameters.	5.3 Laboratory parameters derivations
			• Moved technical notes of special statistical methods for OS into a separate section 5.4.3 instead of 5.4.1.	5.4 Statistical models

Table of contents

Table of contents 7
List of tables 9
List of figures 9
1 Introduction 12
1.1 Study design 12
1.2 Study objectives and endpoints 13
1.3 Study estimands 16
1.3.1 Primary estimand 16
1.3.2 Secondary estimand #1 17
1.3.3 Secondary estimand #2 17
2 Statistical methods 18
2.1 Data analysis general information 18
2.1.1 General definitions 19
2.2 Analysis sets 25
2.2.1 Participant classification 25
2.2.2 Withdrawal of Informed Consent 26
2.2.3 Subgroup of interest 26
2.3 Patient disposition, demographics and other baseline characteristics 27
2.3.1 Patient disposition 28
2.4 Treatments (study treatment, rescue medication, concomitant therapies, compliance) 29
2.4.1 Study treatment / compliance 29
2.4.2 Prior, concomitant and post therapies 32
2.5 Analysis of the primary objective 33
2.5.1 Primary endpoint 33
2.5.2 Statistical hypothesis, model, and method of analysis 34
2.5.3 Handing of intercurrent events of primary estimand 34
2.5.4 Handling of missing values/censoring/discontinuations 35
2.5.5 Sensitivity analyses 37
2.5.6 Supplementary analyses 37
2.6 Analysis of the key secondary objectives 42
2.6.1 Key secondary endpoints 42
2.6.2 Statistical hypothesis, model, and method of analysis 44
2.6.3 Handing of intercurrent events of secondary estimands 44

2.6.4 Handling of missing values/censoring/discontinuations 45
2.6.5 Sensitivity analyses 46
2.6.6 Supplementary analyses on Key secondary endpoints 46
2.7 Analysis of other secondary and exploratory objective(s) 48
2.7.1 Other Secondary and Exploratory endpoints 49
2.7.2 Statistical hypothesis, model, and method of analysis 53
2.7.3 Handling of missing values/censoring/discontinuations 53
2.8 Safety analyses 53
2.8.1 Adverse events (AEs) 53
2.8.2 Deaths 56
2.8.3 Laboratory data 56
2.8.4 Other safety data 58
2.9 Pharmacokinetic analysis 60
2.10 Dosimetry analysis 61
2.11 Patient-reported outcomes 61
2.12 Duration of follow-up 61
2.13 Interim analysis 62
3 Sample size calculation 62
3.1 Power for analysis of key secondary variables 63
4 Change to protocol specified analyses 63
5 Appendix 63
5.1 Imputation rules 63
5.1.1 Study treatment 63
5.1.2 AE, ConMeds and safety assessment date imputation 64
5.2 AEs coding/grading 67
5.3 Laboratory parameters derivations 67
5.4 Statistical models 69
5.4.1 Primary analysis 69
5.4.2 Key secondary analysis 71
5.4.3 Special statistical methods for OS analysis 72
5.5 Determination of missing adequate assessments 78
6 References 80

List of tables

Table 1.2-1 Study objectives and endpoints 14
Table 2.1-1 Time Window for PROs 23
Table 2.1-2 Last contact date data sources 24
Table 2.2-1 Subject classification based on protocol deviations and non-PD criteria 25
Table 2.5-1 Derivation of primary PFS 36
Table 2.5-2 Inclusion/exclusion of assessments used in waterfall graph 40
Table 2.5-3 Comparison of PFS using RECIST 1.1 between central review and investigator 40
Table 2.5-4 Comparison of PFS event times using RECIST 1.1 between central review and investigator assessments 41
Table 2.6-1 Overall lesion response at each assessment: patients with non-target disease only 42
Table 2.6-2 Derivation of TTD 45
Table 2.8-1 Adverse events of special interest 54
Table 2.8-2 Clinically notable ECG values 59
Table 2.8-3 Clinically notable changes in vital signs 59
Table 5.1-1 Imputation of start dates (AE, CM) and assessments (LB, EG, VS) 64
Table 5.1-2 Imputation of end dates (AE, CM) 65
Table 5.5-1 Schedule for tumor assessment and time windows 79

List of figures

Figure 1.1-1 Study design 13

List of abbreviations

AE	Adverse event
AESI	Adverse Events of Special Interest
ALP	Alkaline Phosphatase
ALT/ALAT	Alanine Aminotransferase
AST/ASAT	Aspartate Aminotransferase
ATC	Anatomical Therapeutic Classification
AUC	Area Under the Curve
BIRC	Blinded Independent Central Review
Bid	bis in diem/twice a day
BOR	Best Overall Response
CRS	Case Retrieval Strategy
CMH	Cochran-Mantel-Haenszel
CSR	Clinical Study report
CTC	Common Toxicity Criteria
CTCAE	Common Terminology Criteria for Adverse Events
DI	Dose Intensity
DMC	Data Monitoring Committee
FAS	Full Analysis Set
ECG	Electrocardiogram
eCRF	Electronic Case Report Form
EOS	End of Study
FAS	Full Analysis Set
GEP-NET	Gastro-Entero-Pancreatic Neuroendocrine Tumors
ITT	Intent to Treat
IVR	Interactive Voice Response
IWR	Interactive Web Response
MedDRA	Medical Dictionary for Regulatory Activities
LAR	Long-Acting Repeatable
NCI	National Cancer Institute
o.d.	Once Daily
ORR	Objective Response Rate
OS	Overall Survival
PDI	Planned dose intensity
PFS	Progression-Free Survival
PK	Pharmacokinetics
pNET	Pancreatic NET
PPS	Per-Protocol Set
PRO	Patient-reported Outcomes
Qd	Qua'que di'e / once a day
QoL	Quality of Life

RAP	Report and Analysis Process
RDI	Relative Dose Intensity
RECIST	Response Evaluation Criteria in Solid Tumors
SAE	Serious Adverse Event
SAP	Statistical Analysis Plan
SOC	System Organ Class
SSAs	Somatostatin Analogs
TEAE	Treatment Emergent Adverse Event
TFLs	Tables, Figures, Listings
TTD	Time to Deterioration
ULN	Upper Limit of Normal
WBC	White blood cells
WHO	World Health Organization

1 Introduction

CAAA601A22301 (NETTER-2) is a phase III multi-center, randomized, open-label study to evaluate the efficacy and safety of Lutathera in patients with Grade 2 and Grade 3 advanced Gastro-Entero-Pancreatic Neuroendocrine tumor (GEP-NET).

The content of this SAP is based on protocol CAAA601A22301 (NETTER-2) version 2.0 released on 05-OCT-2022. All decisions regarding the analysis of the primary and key secondary endpoints, as defined in this document, have been made prior to the database lock and unblinding of the study data.

1.1 Study design

This is a multicenter, stratified, randomized, open-label comparator-controlled, Phase III study in patients ≥15 years with somatostatin receptor positive, well-differentiated G2 and G3, advanced GEP NETs, diagnosed within 6 months prior to screening.

The study consists of a Screening Phase, a Treatment Phase, an optional Treatment Extension Phase (cross-over to Lutathera) for patients assigned to high-dose octreotide long-acting, an optional Re-Treatment Phase (re-treatment with Lutathera) for patients assigned to Lutathera plus octreotide long-acting, and a Follow-up Phase as shown in the below figure.

Overall, approximately 222 patients will be randomized (2:1 randomization ratio) to receive treatment with Lutathera (7.4 GBq/200 mCi x 4 administrations every 8 ± 1 weeks; cumulative dose: 29.6 GBq/800 mCi) plus octreotide long-acting (30 mg every 8 weeks during Lutathera treatment and every 4 weeks after last Lutathera treatment) or high dose octreotide long-acting (60 mg every 4 weeks). Randomization will be stratified by tumor grade (G2 vs G3) and tumor origin (pancreatic NET [pNET] vs other origin).

The primary endpoint of the study is radiological Progression-Free Survival (PFS) centrally assessed. The primary efficacy and safety analysis will be performed after 99 PFS events (99 evaluable and centrally confirmed disease progressions or death events) have occurred. This is expected to occur about 35 months after the first patient was randomized.

At the time of the PFS primary analysis, the key secondary endpoints of Objective Response Rate (ORR), and the Time to Deterioration (TTD) in the following scales from the EORTC QLQ-C30 questionnaire: global health status, diarrhoea, fatigue, and pain, will also be assessed.

The End of Study (EOS) is after four years have elapsed from the randomization of the last patient or six months after the last cross-over or re-treatment dose in the study, whichever occurs last. The time window to start cross-over or re-treatment with Lutathera in this study is within four years after the last patient has been randomized. No formal interim efficacy analysis for inferential purposes is planned for the primary and key secondary endpoints in this study.

Figure 1.1-1 Study design

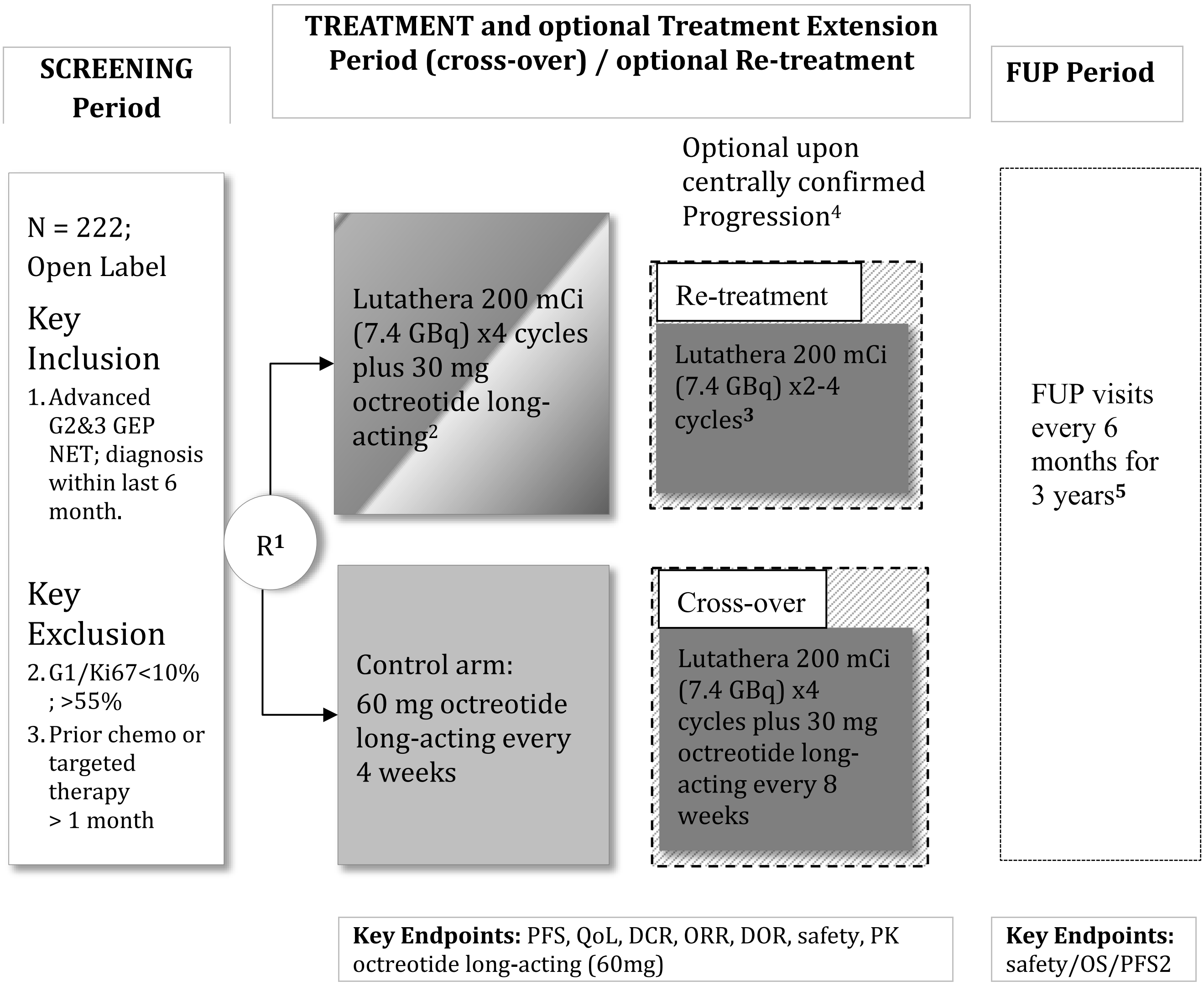

[1]Randomization ratio: 2:1. Stratification Factors: Grade (G2 vs G3) and Tumour Origin (pNET vs other origin).
[2]Octreotide long-acting 30 mg every 8 weeks during Lutathera treatment and every 4 weeks after last Lutathera treatment.
[3]Somatostatin analogues in re-treatment phase are at discretion of investigator
[4]If RECIST progression occurs after Week 72 post the primary end point analysis, the decision to enroll the patient in re-treatment will be based on local assessment.
[5]Patients included in the optional crossover or re-treatment phase will be followed up to 3 years or EoS, whichever occurs first.
For additional details on the efficacy and safety assessment schedules by visit, see Table 8-3 and Table 8-4 of the protocol.

1.2 Study objectives and endpoints

The study's primary, secondary, and explorative objectives and endpoints are described in Table 1.2-1.

Table 1.2-1 Study objectives and endpoints

**Primary Objective**	**Endpoint for primary objective**
To demonstrate that Lutathera is superior to the active comparator in delaying the time-to-first occurrence of progression or death (PFS) as first line treatment	PFS: Time from randomization to the first line progression (centrally assessed according to RECIST 1.1) or death due to any cause
**Key Secondary Objectives**	**Endpoints for key secondary objectives**
-To demonstrate the superiority of Lutathera, compared to active comparator, in terms of objective response	-ORR: Rate of patients with best overall response of partial response (PR) or complete response (CR) or stable disease (SD) (centrally assessed according to RECIST 1.1)
-To demonstrate the superiority of Lutathera, compared to active comparator, in terms of time to deterioration in selected QoL items/scales	-Time to deterioration (TTD) by 10 points from baseline in the following scores measured by the EORTC QLQ-G.I.NET21 questionnaire andEORTC QLQ-C30 questionnaire: global health status, diarrhea, fatigue and pain.
**Other Secondary Objectives**	**Endpoints for other secondary objectives**
-To evaluate the efficacy of Lutathera, compared to active comparator, in keeping the disease under control	-DCR: Rate of patients with best overall response of partial response (PR) or complete response (CR) or stable disease (SD) (centrally assessed according to RECIST 1.1)
-To evaluate the efficacy of Lutathera, compared to active comparator, in terms of duration of response	-DOR: The Duration of Response (DOR) is defined as the time from initially meeting the criteria for response (CR or PR) until the time of progression according to RECIST 1.1 or death due to underlying disease only.
-To evaluate the safety and tolerability of Lutathera	-Rate of adverse events and laboratory toxicities (scored according to CTCAE grade).
-To evaluate the effect of Lutathera on survival	-Time from the randomization date until the day of death due to any cause.
**Exploratory Objectives**	**Endpoints for exploratory objectives**

-To explore the effect of Lutathera on Time to Second Progression (PFS2)	-Time from randomization to objective tumor progression on next line treatment or death due to any cause (PFS2), assessed locally according to RECIST 1.1.
-To explore Health related quality of life (QoL) as measured by the EORTC QLQ-G.I.NET21, EORTC QLQ-C30, EQ-5D-5L questionnaires	-TTD for Items/Scales not included among key secondary endpoints. Change from baseline in the total score for health status from the EQ-5D questionnaire.
-To explore PK of octreotide long-acting at the high dose of 60 mg.	-Steady-state trough plasma concentration of octreotide.
-To explore the safety and efficacy (ORR, PFS, PFS2, DoR, OS) of re-treatment with Lutathera in progressive patients in Lutathera arm	- Safety (Re-treatment): Rate of adverse events and laboratory toxicities during re-treatment with Lutathera.<br>- ORR (Re-treatment): Rate of patients with best overall response of partial response (PR) or complete response (CR) after receiving re-treatment with Lutathera (locally assessed according to RECIST 1.1).<br>- PFS (Re-treatment): Time from the 1st dose of re-treatment with Lutathera to objective tumor progression (locally assessed according to RECIST 1.1) or to death due to any cause.<br>- PFS2 (Re-treatment): Time from randomization to the objective tumor progression (locally assessed according to RECIST 1.1) or to death due to any cause after receiving re-treatment with Lutathera.<br>- DOR (Re-treatment): Time from initially meeting the criteria for response (CR or PR) after receiving re-treatment with Lutathera until the time of progression according to RECIST 1.1 (locally assessed) or death due to underlying disease only.<br>- OS (Re-treatment): Time from the randomization date until the day of death due to any cause in patients who have received re-treatment with Lutathera.

1.3 Study estimands

1.3.1 Primary estimand

The scientific objective guiding the primary analysis is to demonstrate the superiority of Lutathera plus octreotide long-acting compared to high dose octreotide long-acting in delaying the time to first occurrence of radiological progression or death, for the target population, based on central assessment, had no participant initiated new antineoplastic therapy, crossed over to Lutathera or being re-treated with Lutathera.

The primary efficacy endpoint, PFS, is defined as the time from randomization to the first line progression (centrally assessed according to RECIST v1.1) or death due to any cause, before initiation of any new antineoplastic therapy, cross-over to Lutathera or re-treatment with Lutathera (hereafter referred to as new therapies) and regardless of treatment discontinuation. If a participant has not progressed or died before initiation of a new therapy, then PFS will be censored at the date of the last adequate tumor assessment prior to start of the new therapy.

The primary estimand is comprehensively described by the following attributes:

1. The **target population** comprises all participants randomized with somatostatin receptor positive, well differentiated G2 (Ki67 index ≥10% to ≤ 20%) or G3 (Ki67 > 20% and ≤ 55%) advanced GEP-NETs, previously untreated with any systemic therapies other than Somatostain analogues (SSAs) for inoperable metastatic disease. Patients with documented RECIST progression to SSAs for the current GEP-NET at any time prior to randomization are not eligible to participate in this study.
2. The **primary variable** is PFS, defined as the time from the date of randomization to the date of the first documented radiological progression or death due to any cause, based on central assessment and using RECIST v1.1 criteria.
3. **Intercurrent events** of interest in this study are:
    a. Discontinuation of study treatment for any reason before central PFS event: this intercurrent event is ignored (treatment policy strategy).
    b. New anti-neoplastic therapy, including cross-over to Lutathera, before central PFS event: PFS is censored at the date of the last adequate tumor assessment prior to initiation of the new anti-neoplastic therapy (hypothetical strategy).
    c. Re-treatment before central PFS event: PFS is censored at the date of the last adequate tumor assessment prior to re-treatment (hypothetical strategy).
4. The **summary measure** is hazard ratio for PFS between the 2 treatments. It will be estimated using the Cox proportional hazards model stratified by randomization stratification factors. The primary comparison will be performed using log-rank test stratified by randomization stratification factors.

1.3.2 Secondary estimand #1

The first key secondary objective is to demonstrate the superiority of Lutathera plus octreotide long-acting compared to high dose octreotide long-acting, in terms of objective response rate, for the target population, based on central assessment regardless of discontinuation of study treatment.

The first key secondary endpoint, ORR, is defined as the proportion of participants with best overall response (BOR) of unconfirmed complete response (CR) or unconfirmed partial response (PR), as per central review and according to RECIST v1.1, reported before initiation of any new therapy.

The secondary estimand #1 is comprehensively described by the following four attributes:

1. The **target population** comprises all participants randomized with somatostatin receptor positive, well differentiated G2 (Ki67 index ≥10% to ≤ 20%) or G3 (Ki67 > 20% and ≤ 55%) advanced GEP-NETs, previously untreated with any systemic therapies other than Somatostain analogues (SSAs) for in-operable metastatic disease. Patients with documented RECIST progression to SSAs for the current GEP-NET at any time prior to randomization are not eligible to participate in this study.
2. The **variable** is best overall response (BOR) with unconfirmed complete response (CR) or unconfirmed partial response (PR), as per central review and according to RECIST 1.1.
3. **Intercurrent events** of interest in this study are:
    a. Discontinuation of study treatment for any reason before response.
    b. New anti-neoplastic therapy, including cross-over to Lutathera, before response.
    c. Re-treatment before response.

   Details on how to handle intercurrent events are provided in Section 2.6.3.
4. The **summary measure** is the odds ratio for ORR between the 2 treatments. It will be estimated using the Cochran-Mantel-Haenszel (CMH) with stratification by randomization stratification factors.

1.3.3 Secondary estimand #2

The second key secondary objective is to demonstrate the superiority of Lutathera plus octreotide long-acting compared to high dose octreotide long-acting, in terms of time to deterioration in selected QoL items/scales, had no participant initiated new antineoplastic therapy, crossed over to Lutathera or being re-treated with Lutathera.

The second key secondary endpoint, time to deterioration (TTD) for EORTC QLQ-C30 questionnaire in the following scores: global health status, diarrhoea, fatigue, and pain, is defined as time from randomization to the first deterioration of at least 10 points in score compared to the baseline score or death while on-treatment (Strosberg *et al.* 2018), before initiation of any new antineoplastic therapy, cross-over to Lutathera or re-treatment with

Lutathera (hereafter referred to as new therapies) and regardless of use of symptomatic medications while on treatment. Each selected item/scale is analyzed separately as outlined.

The secondary estimand #2 is comprehensively described by the following four attributes:

1. The **target population** comprises all participants randomized with somatostatin receptor positive, well differentiated G2 (Ki67 index ≥10% to ≤ 20%) or G3 (Ki67 > 20% and ≤ 55%) advanced GEP-NETs, previously untreated with any systemic therapies other than Somatostain analogues (SSAs) for in-operable metastatic disease. Patients with documented RECIST progression to SSAs for the current GEP-NET at any time prior to randomization are not eligible to participate in this study.
2. The **variable** is TTD, defined as the time from the date of randomization to the date of the first deterioration of 10 points (TTD) of the considered item/scale or death while on-treatment.
3. **Intercurrent events** of interest in this study are:
    a. Discontinuation of study treatment for any reason before deterioration.
    b. New anti-neoplastic therapy, including cross-over to Lutathera, before deterioration.
    c. Re-treatment before deterioration.
    d. Use of symptomatic medications associated with the considered scale/item before deterioration.

   Details on how to handle intercurrent events are provided in Section 2.6.3.
4. The **summary measure** is hazard ratio for TDD between the 2 treatments. It will be estimated using the Cox proportional hazards model stratified by randomization stratification factors. The primary comparison will be performed using log-rank test stratified by randomization stratification factors.

2 Statistical methods

2.1 Data analysis general information

All planned analyses will be performed by the Sponsor. SAS version 9.4 or later will be used to perform all data analyses and to generate tables, figures and listings.

Data included in the analysis

The primary analysis is planned for the efficacy (including primary efficacy endpoint) and safety after 99 PFS events have been confirmed. A unique cut-off date will be established after the targeted number of events for the planned analysis has been documented. Similarly, at End of Study a unique cut-off date will be established. For each of the analysis time points, all statistical analyses will be performed using all data collected in the database up to the data cut-off date. All data with an assessment date or event start date (e.g., vital sign assessment

date or start date of an adverse event) prior to or on the cut-off date will be included in the analysis. Any data collected beyond the cut-off date will not be included in the analysis and will not be used for any derivations.

All events with start date before or on the cut-off date and end date after the cut-off date will be reported as ‘ongoing’. The same rule will be applied to events starting before or on the cut-off date and not having documented end date.

The analysis cutoff date for the final analysis of study data will be established at the end of the study after four years have elapsed from the randomization of the last patient or six months after the last cross-over or re-treatment dose in the study, whichever occurs last.

General analysis conventions

Pooling of centers: Unless specified otherwise, data from all study centers will be pooled for the analysis. Due to expected small number of patients enrolled at centers, no center effect will be assessed. Protocol deviations, number of patients in analysis populations and discontinuations from study treatment will be summarized by center.
Qualitative data (e.g., gender, race, etc.) will be summarized by means of contingency tables by treatment group; a missing category will be included as applicable. Percentages will be calculated using the number of patients in the relevant population or subgroup as the denominator.

Quantitative data (e.g., age, body weight, etc.) will be summarized by appropriate descriptive statistics (i.e., mean, standard deviation, median, Q1, Q3, minimum, and maximum) by treatment group.

2.1.1 General definitions

2.1.1.1 Investigational drug and study treatment

Investigational drug, will refer to Lutathera® ([^{177}Lu]Lu-DOTA-TATE) only.

Whereas, ***study treatment*** will refer to the combination of drugs in the experimental and control treatment arms: Lutathera® plus Sandostatin® LAR Depot (octreotide long-acting), and, Sandostatin® LAR Depot (octreotide long-acting), respectively.

The term ***investigational arm*** will be used to refer to patients who were randomized to receive investigational drug, and, the term ***control arm*** will refer to those patients that were randomized to receive high dose octreotide long-acting (60 mg) in the treatment phase.

2.1.1.2 Date of first administration of study treatment component

The date of first administration of certain component of study treatment in a specific study treatment period is defined as the first date when a non-zero dose of that component of study treatment is administered in that period. The date of first administration of component of study treatment will also be referred to as the start of component of study treatment.

2.1.1.3 Date of last administration of study treatment component

The date of last administration of certain component of study treatment in a specific study treatment period is defined as the last date when a non-zero dose of that component of study treatment is administered in that period. The date of last administration of component of study treatment will also be referred to as the end of component of study treatment.

2.1.1.4 Date of first administration of study treatment

The date of first administration of study treatment in a specific study treatment period is defined as the first date when a non-zero dose of any component of study treatment is administered in that period. The date of first administration of study treatment will also be referred to as the start of study treatment. (Example: if 1st dose of Lutathera® is administered on 03-April-2020, and 1st dose of Sandostatin® LAR Depot (octreotide long-acting) is administered on 04-April-2020, then the date of first administration of study treatment is on 03-April-2020.)

2.1.1.5 Date of last administration of study treatment

The date of last administration of study treatment in a specific study treatment period is defined as the last date when a non-zero dose of any component of study treatment is administered in that period. The date of last administration of study treatment will also be referred to as the end of study treatment. In this particular study, Lutathera® is given every 8 weeks up to four doses, and Sandostatin® LAR Depot (octreotide long-acting).

(Example: if last dose of Lutathera® is administered on 03-April-2021, and last dose of Sandostatin® LAR Depot (octreotide long-acting) is administered on 04-April-2022, then the date of last administration of study treatment is on 04-April-2022).

2.1.1.6 Study day

The study day, describes the day of the event or assessment date, relative to the reference start date.

The reference start date is designated as Study Day 1. Study Day -1 is the day that preceded Day 1. Study Day 0 is not defined. Study day is not to be used in numerical computations, for example in calculating exposure. The reference start date for patient's age is the date of patient's Informed Consent.

The study day is defined as:

- The date of the event (visit date, onset date of an event, assessment date etc.) − reference start date + 1 if event is on or after the reference start date;
- The date of the event (visit date, onset date of an event, assessment date etc.) − reference start date if event precedes the reference start date.

The reference start date for **safety assessments (**e.g., adverse event onset, laboratory abnormality occurrence, vital sign measurement, Karnofsky performance status, dose interruption, etc..) is the start of study treatment. The reference start date for **all other, non-**

safety assessments (i.e., tumor assessment, death, disease progression, tumor response, and patient reported outcomes (PRO)) is the date of randomization.

The study day will be displayed in the data listings. If an event starts before the reference start date, the study day displayed on the listing will be negative.

For efficacy analysis related to the cross-over or re-treatment period, study day is defined as above, with the reference date being the date of first administration of Lutathera® during these periods.

Key note: For certain project level long-term safety examinations of interest with Lutathera® (such as myelodysplastic syndrome and acute myeloid leukemia), the start date of these analyses will be the 1st dose of Lutathera® treatment, irrespective of patient entering either cross-over or re-treatment phase.

2.1.1.7 Time unit

A year length is defined as 365.25 days. A month length is 30.4375 days (365.25/12). Duration reported in months will be obtained by dividing duration in days by 30.4375. Duration reported in years will be obtained by dividing duration in days by 365.25.

2.1.1.8 Baseline

For efficacy evaluations, the last non-missing assessment, including unscheduled assessments on or before the date of randomization is taken as “baseline” value or “baseline” assessment. In the context of baseline definition, the efficacy evaluations also include PRO.

For safety evaluations (e.g., laboratory assessments, ECGs, and vital signs), the last available assessment, including unscheduled assessments, on or before the start of study treatment as described in Section 2.1.1.4 will be used as the “baseline” assessment. Assessments specified to be collected post-dose on the 1st date of treatment are not considered as baseline values.

If participants have no value as defined above, the baseline results will be considered missing.

Since study treatment in the randomized treatment phase can still potentially contribute to the safety profile in the cross-over and re-treatment phase, therefore, for cross-over and re-treatment analyses, baseline for safety analysis is the same as defined above, i.e., last available assessment on or before the start of study treatment.

For efficacy evaluations of the cross-over and re-treatment period, the last available assessment on or before first cross over treatment/re-treatment with Lutathera® is regarded as “baseline” for the assessments.

2.1.1.9 Definition of study phases

Randomized Treatment Phase

The randomized treatment phase starts on the randomization date and ends at the end of treatment date filled on the end of treatment CRF page (For EOT during treatment phase).

Extension Treatment Phase

For the control arm patients going into the cross-over period and receiving Lutathera treatment, the cross-over phase starts at the date of 1st treatment with Lutathera and ends at the end of treatment date filled on the end of treatment cross-over CRF page.

For the investigational arm patients going into the re-treatment period, the re-treatment period starts at the date of 1st re-treatment with Lutathera and ends at the end of treatment date filled on the end of treatment re-treatment page.

Follow-up Phase

The Follow-up phase starts one day after the end of treatment phase/extension phase (whichever was the last phase that the patient completed) and ends at the end of study date filled on the end of study CRF Page.

2.1.1.10 Definition of treatment periods used in analyses

Pre-treatment period:

From day of patient's informed consent to the day before first administration of study treatment in the treatment phase.

Randomized treatment period:

An on-treatment assessment/event during randomized treatment period is defined as any assessment/event in the following time intervals:

- [date of first administration of study treatment, minimum (date of last administration of study treatment in randomized treatment phase + 30 days, 1st injection of Lutathera in extension phase – 1 day)]

Between treatments period:

This period starts from the end of randomized treatment period as defined above to the day before the 1st injection of Lutathera in the extension (cross-over/re-treatment) treatment period. It is not applicable for patients without cross-over/re-treatment and for those who start the cross-over/re-treatment period less than 30 days from the last administration of study treatment in the randomized treatment period.

Cross-over or re-treatment period:

An assessment/event during the cross-over or re-treatment period is defined as any assessment/event in the following time interval:

- [date of first administration (or re-treatment) of Lutathera, date of last administration of study treatment + 30 days]

Note: the calculation of study treatment duration will use different rules as specified in Section 2.4.1.1.

Due to the limited and immature data in cross-over/re-treatment period, efficacy analyses for this period or the corresponding subset will not be generated at the time of primary PFS analysis. Instead, they will be summarized in the future for study update and publication use. Nevertheless, selected key safety output will be generated to include this period for the completeness of safety review.

Note: The date of first administration of study treatment and the date of last administration of study treatment are defined in Section 2.1.1.4 and Section 2.1.1.5, respectively.

Post-treatment follow up period:

The post treatment period is defined as the following:

- For patients who do not receive either cross-over or re-treatment: Post-treatment period starts at the 31st day after the last study treatment administration in the randomized Treatment Phase (i.e,. the day after end of on-treatment period) to the End of Study.
- For patients who cross over to Lutathera: Post-treatment period starts at the 31st day after the last study treatment administration in the Extension Treatment Phase (cross-over) to the End of Study.
- For patients who receive re-treatment with Lutathera: Post-treatment period starts at the 31st day after the last study treatment administration in the Extension Treatment Phase (re-treatment) to the End of Study.

2.1.1.11 Windows for multiple assessments

For the definition of time windows for visits please refer to the study protocol, [Tables 8-3] and [Table 8-4]. Variations of ± 1 week in the visits schedule are allowed.

Time windows will be defined for descriptive summary of PRO data by visit and longitudinal data analysis. If more than one assessment is available in the same time window, the assessment closest to the planned date will be considered. If two assessments are obtained equidistant compared to the scheduled visit day, the assessment obtained prior to visit will be considered.

Table 2.1-1 Time Window for PROs

Time Window	Planned Visit Timing	Time Window Definition
**On treatment**		
Baseline	On or before Study Day 1*	≤ Study Day 1*
Week 12	Study Day 78	Study Days 2 – 120
Week 24	Study Day 162	Study Days 121 – 204
Week 36	Study Day 246	Study Days 205 – 288
Every 12 weeks thereafter		
Week 12*k (k = 4, 5, …)	d = 7*(12*k-1)+1	Study Days d-41 to d+42<br>Note: window for last visit before EOT: d-41 to EOT date-1

End of treatment during randomized treatment period		
End of treatment	N.A.	Data collected under EOT visit
* Study Day 1 = randomization date		

Data from all assessments (scheduled and unscheduled), including multiple assessments, will be listed.

Last contact date

The last contact date will be derived for patients not known to have died at the analysis cut-off using the last complete date among the following:

Table 2.1-2 Last contact date data sources

Source data	Conditions
Date of Randomization	No Condition
Last contact date/last date patient was known to be alive from Visit Form 3	Patient status is reported to be alive
Start/End* dates from drug administration record	Non-missing dose. Doses of 0 are allowed.
End of treatment date from end of treatment page	No condition.
Date of PRO assessment	At least one non-missing answer to questionnaire
Tumor (RECIST) assessment date	For non-target lesion: non-missing lesion status;<br>For target lesion: non-missing lesion diameter;<br>For new lesion: “Is there a new lesion?” = yes
Laboratory/PK collection date	Sample collection marked as ‘done’.
Vital signs and ECG date	At least one non-missing parameter value
Performance Status date	Non-missing performance status
Start/End dates of AE	Non-missing verbatim term
Concomitant medication/Non-drug therapy date	Non-missing medication
Physical exams	At least one non-missing value

The last contact date is defined as the latest complete date from the above list. The cut-off date will not be used for last contact date, unless the patient was seen or contacted on that date. No date post cut-off date will be used. Completely imputed dates (e.g., the analysis cut-off date programmatically imputed to replace the missing end date of a dose administration record) will not be used to derive the last contact date. Partial date imputation is allowed to derive the last contact date to estimate death or censoring coming from the death form or end of study eCRF, respectively, and these rules are summarized in Section 5.1.2.1.

The last contact date will be used for censoring of patients in the analysis of overall survival.

2.2 Analysis sets

Full Analysis Set

The Full Analysis Set (FAS) comprises all patients to whom study treatment has been assigned by randomization. According to the intent to treat (ITT) principle, patients will be analyzed according to the treatment and strata, they have been assigned to during the randomization procedure. The FAS will be the primary population for all efficacy analyses.

Safety set

The Safety set (SAF) includes all subjects who received at least one dose of study treatment. Patients will be analyzed according to the study treatment received, where treatment received is defined as the randomized treatment if the subject took at least one dose of that treatment or the first treatment received if the randomized/assigned treatment was never received. So, if a patient randomized to the Lutathera arm receives only Octreotide, that patient will be counted as an Octreotide arm patient as far as safety is concerned. Likewise, if a patient randomized to the Octreotide arm receives Lutathera instead, that patient counts as a Lutathera patient.

Cross-over set

The cross-over set is comprised of all patients randomized to the Sandostatin® LAR Depot (octreotide long-acting) arm who received at least one cross-over dose of Lutathera® (^{177}Lu-DOTA^{0}-Tyr^{3}-Octreotate) following confirmed disease progression per BIRC in the randomized period.

Re-treatment set

The re-treatment set is comprised of all patients randomized to the Lutathera® (^{177}Lu-DOTA^{0}-Tyr^{3}-Octreotate) arm who received at least one re-treatment dose of Lutathera® (^{177}Lu-DOTA^{0}-Tyr^{3}-Octreotate) after confirmed disease progression per BIRC in the randomized period.

Pharmacokinetic analysis set (PAS)

The Pharmacokinetic analysis set (PAS) includes all subjects who provide at least one evaluable PK concentration at baseline and post-dose.

2.2.1 Participant classification

Patients may be excluded from the analysis populations defined above based on the protocol deviations entered in the database and/or on specific subject classification rules defined in Table 2.2-1.

Table 2.2-1 Subject classification based on protocol deviations and non-PD criteria

Analysis set	Protocol deviations leading to exclusion	Non protocol deviation leading to exclusion

FAS	No written informed consent	Not applicable
Safety set	No written informed consent	No dose of study medication
Cross-over Set	Cross-over specific ICF not completed	Not applicable
Re-treatment Set	Re-treatment specific ICF not completed	Not applicable
PK Analysis Set	No written informed consent	No evaluable PK concentration at baseline or post-dose.

2.2.2 Withdrawal of Informed Consent

Any data collected in the clinical database after a subject withdraws informed consent from all further participation in the trial, will not be included in the analysis. The date on which a patient withdraws full consent is recorded in the eCRF.

Death events may be used in the analysis if captured from public records (registers), local law and subject informed consent permitting.

Additional data for which there is a separate informed consent, e.g., PK, biomarker etc., collected in the clinical database without having obtained that consent will not be included in the analysis. These data will be excluded by the presence of the appropriate protocol deviation criterion.

2.2.3 Subgroup of interest

Subgroup analyses will be performed for efficacy and safety as outlined below.

Efficacy

The primary efficacy and key secondary endpoints will be summarized by the following subgroups to examine the homogeneity of treatment effect provided that the primary efficacy analysis, and, key secondary analysis, based on the FAS are statistically significant:

- Tumor grade: G2 vs. G3 per eCRF
- Tumor origin: pNET vs. other origin per eCRF
- Small intestine as primary site of cancer (a subset of other origin per eCRF)
- Age category (<65 years vs. ≥ 65 years)
- Sex (Female vs. Male)
- Race (White vs. Asian)
- Tumor burden at baseline (Limited vs. Moderate vs. Extensive)

- Chromogranin A (CgA) prior to randomization (≤2xULN vs. > 2xULN)
- SSTR highest tumor uptake score as per central assessment (Grade 3 vs. Grade 4)

So, if the primary efficacy endpoint is significant, the above applies to that endpoint. If a key secondary endpoint is significant, the above applies to that endpoint.

No formal statistical test of hypotheses will be performed for the subgroups, only point estimate of the treatment effect and 95%-confidence intervals will be provided (see Section 2.5.6 for further analysis details). The objective of the efficacy subgroup analysis is to demonstrate homogeneity of treatment effect in the above subgroups.

Safety

Safety subgroup analyses will use the same method as for the analysis in the overall analysis set. Key safety analyses (deaths and SAEs) will be repeated on safety set in the following subgroups:

- Age category (<65 years vs. ≥ 65 years)
- Sex (Female vs. Male)
- Race (White vs. Asian)
- Creatine clearance at baseline (<60 mL/min vs. ≥ 60 mL/min)

The objective for carrying out these subgroup analyses is to identify potential safety issues that may be limited to a subgroup of patients, or safety issues that are more commonly observed in a subgroup of patients.

Summary tables will only be performed if at least 10% of patients or 10 patients are present in each subgroup. Some grouping of classes will be considered if necessary to obtain a sufficient number of observations.

2.3 Patient disposition, demographics and other baseline characteristics

The Full Analysis Set (FAS) will be used for all baseline and demographic summaries and listings unless otherwise specified. Summaries will be reported by treatment arm and for all patients and listings will be reported by treatment arm to assess baseline comparability. No inferential statistics will be provided. Demographic and baseline characteristics will also be summarized for patients who cross over to Lutathera or are re-treated with Lutathera, separately, unless otherwise specified.

Basic demographic and background data

All demographic and baseline disease characteristics data will be summarized and listed by treatment arm. Categorical data (e.g., gender, race, ethnicity, Karnofsky performance score as categorical) will be summarized by frequency counts and percentages; the number and percentage of patients with missing data will be provided. Those with Asian race will be further

broken down by Chinese, Indian, Japanese, Korean, or Vietnamese. Those with multiple races will be included in Other category. Continuous data (e.g., age, weight, height, body mass index) will be summarized by descriptive statistics (N, mean, median, standard deviation, Q1, Q3, minimum and maximum). Separate summaries will be prepared for patients who cross over to Lutathera or are re-treated with Lutathera.

Baseline stratification factors

The number (%) of patients in each randomization stratum based on data obtained from the IRT system will be summarized overall and by treatment arm for the FAS.

Diagnosis and extent of cancer

Summary statistics will be tabulated for diagnosis and extent of cancer. This analysis will include the following: primary site of cancer, presence of metastases, site of metastases, histopathology grade at diagnosis, time since initial diagnosis, presence/absence of target and non-target lesions, number and type of metastatic sites involved, ki67, highest SSTR tumor uptake score (based on cancer diagnosis page), extent of overall tumor burden, and, TNM criteria at diagnosis. Note: Presence/absence of target and non-target lesions will be based on the central laboratory report on RECIST target/non-target lesion assessment. Metastatic sites will be based on GEP-NET diagnosis page. Separate summaries will be prepared for patients who cross over to Lutathera or are re-treated with Lutathera.

To further characterize primary site of cancer, primary site locations will be grouped into three categories: pNET, small intestine and other by clinical review. The details of grouping will be covered in the programming specifications.

Medical history

Medical history and ongoing conditions, including cancer-related conditions and symptoms entered on (e) CRF will be summarized and listed by treatment arm. This will include Prior Antineoplastic Therapy (GEP-NET tumor procedures (including surgery and radiotherapy), and, GEP-NET tumor medication). Separate summaries will be presented for ongoing and historical medical conditions. The summaries will be presented by primary system organ class (SOC), preferred term (PT) and treatment arm. Medical history and current medical conditions will be coded using the Medical Dictionary for Regulatory Activities (MedDRA) terminology. The MedDRA version used for reporting will be specified in the CSR and as a footnote in the applicable tables/listings.

2.3.1 Patient disposition

Enrollment by country and center will be summarized for all screened patients and also by treatment arm using the FAS. The number (%) of randomized patients included in the FAS will be presented overall and by treatment group. The number (%) of screened and not-randomized patients and the reasons for screening failure will also be displayed. The number (%) of patients in the FAS who are still on treatment, who discontinued the study phases and the reason for discontinuation will be presented overall and by treatment group. Death due to COVID-19 will

be included in the display. Patient disposition will also be summarized for patients who cross over to Lutathera or are re-treated with Lutathera, separately, unless otherwise specified.

Protocol deviations

In this study, the term "major" protocol deviations corresponds to the term "important" protocol deviations.

The number (%) of patients in the FAS with any major protocol deviation will be tabulated by deviation category (as specified in the study Data Management Plan) overall and by treatment group for the FAS. Major protocol deviations related to Covid-19 will be tabulated separately overall and by treatment group. All protocol deviations will be listed.

Analysis sets

The number (%) of patients in each analysis set (defined in Section 2.2) will be summarized by treatment group and stratum.

2.4 Treatments (study treatment, rescue medication, concomitant therapies, compliance)

For details concerning rescue medication see Section 6.1 of the Protocol.

2.4.1 Study treatment / compliance

Duration of study treatment exposure, cumulative dose, number of doses, average duration of treatment cycles (for Lutathera only), dose intensity (DI) and relative dose intensity (RDI) will be summarized by treatment group. The number of participants with dose reductions/interruptions/modification and the reasons, will be summarized and listed. Details of the derivations and summaries are provided in the following sections. Participants with no exposure to the study treatment component will be excluded from the corresponding tabular summaries.

Categorical data will be summarized as frequencies and percentages. For continuous data, mean, standard deviation, median, 25th and 75th percentiles, minimum, and maximum will be presented.

The safety set will be used for all summaries and listings of study treatment.

2.4.1.1 Duration of exposure to study treatment

The duration of exposure to study treatment will be calculated as:

Duration of exposure to study treatment (days) = (**last date of exposure** to any treatment component) – (date of first administration of study treatment component) + 1

The duration of exposure to any single component of study treatment will be calculated as:

Duration of exposure (days) = (last day of exposure to study treatment component) – (date of first administration of study treatment component) + 1

The **last date of exposure** is defined as follows for the study treatment component:

- For Lutathera, the last date of exposure is defined as the last date of administration + 55 days
- For Octreotide LAR, the rules are the following:
  - For patients randomized in the investigational arm, if the last date of administration occurs after an administration of Lutathera, the last date of exposure is defined as the last date of administration for Octreotide + 55 days
  - For patients randomized in the investigational arm, if the last date of administration occurs after the 6-week washout period of the last dose of Lutathera (i.e., Octreotide starts the every 4 weeks schedule after discontinuation of Lutathera), then the last date of exposure is defined as the last date of administration + 27 days
  - For patients randomized in the control arm, the last date of exposure is defined as the last date of administration + 27 days

This duration of exposure includes the periods of temporary interruption (of any component of the study treatment for any reason). The duration of study treatment exposure and exposure to each treatment component will be summarized by treatment group and by treatment period. In addition, number of cyles and average duration of cycle (for Lutathera only) will also be summarized for single component of study treatment by treatment period.

For analyses related to the cross-over/re-treatment period, duration of exposure to Lutathera will be calculated as above during either the cross-over or re-treatment period as appropriate. Since administration of Octreotide LAR is optional in the re-treatment period (expected very few cases), the duration of exposure to Octreotide LAR will not be calculated for the re-treatment period.

2.4.1.2 Cumulative dose

Cumulative dose of a study treatment is defined as the total dose given during the study treatment exposure and will be summarized for each of the study treatment components, separately for randomized period, cross-over period, and re-treatment period, except that the cumulative dose for Octreotide in the re-treatment period will not be summarized due to very limited cases (Octreotide is optional in the re-treament period).

The **planned cumulative dose** for a study treatment component refers to the total planned dose as per the protocol up to the last date of that study treatment component administration.

The planned cumulative dose is not summarized/listed. It is used for relative dose intensity calculations.

The **actual cumulative dose** refers to the total actual dose administered, over the duration for which the subject is on the study treatment as documented in the Dose Administration eCRF.

For patients who did not take any drug the cumulative dose is by definition equal to zero.

2.4.1.3 Dose intensity and relative dose intensity

Dose intensity (DI) for participants with non-zero duration of exposure to each study treatment component is defined as follows:

- DI (dosing unit / unit of time) = Cumulative dose (dosing unit) / Duration of exposure (unit of time).

For participants who did not receive any study drug, the DI is equal to zero. Planned dose intensity (PDI) is defined as the assigned dose by unit of time planned to be given to participants as per protocol in the same dose unit and unit of time as that of the Dose Intensity. DI, PDI and Relative dose intensity (RDI) is defined as:

For Lutathera:

- DI (GBq/week) = Cumulative dose (GBq) / duration of exposure (weeks)
- PDI is 7.4 GBq/8 weeks i.e., 0.925 GBq/week
- RDI (%) = DI (GBq/week) / PDI (Gbq/week) *100

For Octreotide LAR:

- DI (mg/week) = Cumulative dose (mg) / duration of exposure (week)
- PDI is the following:
  - 30 mg every 8 weeks for those randomized in the investigational arm after Lutathera infusion i.e., 3.75 mg/week
  - 30 mg every 4 weeks for those randomized in the investigational arm after Lutathera treatment is complete (or if Lutathera has been suspended) i.e., 7.5 mg/week
  - 60 mg every 4 weeks for those randomized in the control arm i.e, 15 mg/week

Note if Octreotide is administrated > 32 weeks (i.e., first 4 cycles) in the randomized period, then PDI = (7.5*duration of exposure (week)-120)/duration of exposure (week).

- RDI (%) = DI (mg/week) / PDI (mg/week) *100

For each study drug (Lutathera or Octreotide), RDI will be summarized by treatment period. Since DI for Octreotide have different schedules between 2 arms, DI will be only presented for Lutathera to avoid any mis-interpretation and mis-comparison of Octreotide dose intensity between 2 arms.

Both categorical and continuous summaries (i.e., mean, standard deviation, etc.) of RDI for Lutathera and Octreotide LAR will be presented by treatment period.

Similarly, since administration of Octreotide LAR is optional in the re-treatment period (expected very few cases), DI and RDI for Octreotide LAR will not be calculated for the re-treatment period.

2.4.1.4 Dose delays/interruptions/modifications

The number of subjects who have dose delays/interruptions (for Lutathera only)/modifications, and the reasons, will be summarized separately for each of the study treatment components.

For Lutathera, 'Was there any delay?', 'If administered dose is not full dose', and 'Has Lutathera infusion been interrupted?'' fields from the Lutathera treatment eCRF will be used to determine the dose delays/modifications/interruptions, dose reductions, infusion interruptions, respectively. In addition, the reason for dose delays/modifications/interruptions will be presented for the Lutathera arm. The number of cycles delayed, number of infusion interruptions for Lutathera will be summarized descriptively.

For Octreotide LAR, 'Is there any delay?', and 'Different dose, specify' fields from the Octreotide Long-Acting treatment eCRF will be used to determine the dose delays, and dose modifications, respectively. For both arms, delay/modifications of Octreotide dose will be summarized with reason presented. In addition, the number of cycles delayed for Octreotide will be summarized descriptively.

2.4.2 Prior, concomitant and post therapies

Prior anti-cancer therapy

The number and percentage of patients who received any prior anti-neoplastic medications, prior anti-neoplastic radiotherapy or prior anti-neoplastic surgery will be summarized by treatment arm. Prior anti-neoplastic medications will be summarized by therapy type (e.g., chemotherapy, immunotherapy, hormone therapy, and other). The medication therapy type of any combination therapy will be classified based on the following order: chemotherapy, immunotherapy, hormone therapy, other. In addition, prior anti-neoplastic medications will be further summarized by lowest ATC class, preferred term and treatment. The above analyses will be performed using the FAS.

Post treatment anti-cancer therapy

Anti-neoplastic therapies since discontinuation of study treatment will be listed and summarized by ATC class, preferred term, overall and by treatment group by means of frequency counts and percentages using FAS. Anti-neoplastic cancer surgeries/procedures will also be listed and summarized by overall and treatment group using the FAS.

Post treatment anti-neoplastic therapy will include:

- Medications:
    - Medications which are in the ATC2 class of Antineoplastic agents or
    - Medications which are in the ATC4 class of Various therapeutic radiopharmaceuticals
    - Medications which are in the ATC4 class of Somatostatin and Analogues
- Procedures:

- Any radiotherapies
- Procedures (including surgery) with indication of Underlying disease in Concomitant non-drug therapies and interventions eCRF page

Concomitant medications

All medications taken at the start of screening until the end of the treatment phase /optional treatment extension Phase (Cross-over period)/optional re-treatment phase (re-treatment period), or early termination, are to be recorded. This includes prescription and over-the-counter medications taken during this time frame.

During the Follow-up Phase, concomitant medications must be collected only if administered for related SAEs/AESI and/or for secondary hematological malignancies. In addition, further anti-tumor treatments for GEP-NET administered after progression must be reported until the end of the Follow-up phase. Concomitant therapy include medications (other than study drugs) starting on or after the start date of study treatment or medications starting prior to the start date of study treatment and continuing after the start date of study treatment.

Concomitant medications will be coded using the World Health Organization (WHO) Drug Reference Listing (DRL) dictionary that employs the WHO Anatomical Therapeutic Chemical (ATC) classification system and summarized by lowest ATC class and preferred term using frequency counts and percentages. Surgical and medical procedures will be coded using MedDRA and summarized by SOC and preferred term (PT).

All concomitant therapies will be listed. Any concomitant therapies starting and ending prior to the start of study treatment or starting more than 30 days after the last date of study treatment will be flagged in the listing. The safety set will be used for concomitant medication tables and listings for the randomized period.

2.5 Analysis of the primary objective

The primary objective of the study is to determine whether Lutathera is superior to active comparator in delaying the time-to-first occurrence of progression or death (PFS) as first line treatment.

2.5.1 Primary endpoint

The primary endpoint of the study is progression-free survival (PFS), defined as the time from the date of randomization to the date of the first documented progression or death due to any cause. PFS will be assessed via central review according to RECIST 1.1. The Full Analysis Set will be the primary analysis population and will include all data observed up to the cut-off date.

If a patient has not progressed or died by the analysis cut-off date, PFS will be censored at the date of the last adequate tumor evaluation date before the cut-off date.

PFS events documented after the initiation of new anti-neoplastic therapy (i.e., RECIST 1.1 documented disease progression per central review or death) will be censored for the primary analysis at the last adequate tumor assessment prior to start of the new therapy. A separate

sensitivity analysis will be conducted that considers such events as PFS events for the primary analysis provided central review of tumor assessments continues after initiation of new cancer therapy. Additional details regarding censoring rules and determination of date of last adequate tumor assessment are provided in Section 2.5.3.

Discontinuation due to disease progression (collected on the 'End of Treatment' disposition page) without supporting objective evidence satisfying progression criteria per RECIST 1.1 will not be considered disease progression for PFS derivation. Clinical deterioration will not be considered as a qualifying event for progression in the primary analysis.

2.5.2 Statistical hypothesis, model, and method of analysis

Assuming proportional hazards model for PFS, the null hypothesis will be tested at one-sided 2.5% level of significance:

H_{01} (null hypotheses): $\Theta_1 \geq 0$ vs. H_{a1} (alternative hypotheses): $\Theta_1 < 0$, where Θ_1 is the log hazard ratio of PFS in the Lutathera plus Standard Dose octreotide long-acting (30 mg) (investigational) arm vs. High Dose octreotide long-acting (60 mg) (control) arm.

H_0 (null hypothesis): $S_L(t) \leq S_C(t)$ will be tested against the following

H_a (alternative hypothesis): $S_L(t) > S_C(t)$,

where SL(t) is the survival function of PFS in the Lutathera plus Standard Dose octreotide long-acting (30 mg) (investigational) arm, and SC(t) is the survival function of PFS in the High Dose octreotide long-acting (60 mg) (control) arm.

The primary efficacy analysis to test this hypothesis and compare the two treatment groups will consist of a stratified log-rank test at an overall one-sided 2.5% level of significance in favor of the Lutathera plus Standard Dose octreotide long-acting (30 mg) arm. The stratification will be based on following randomization stratification factors (histological grade: G2 vs. G3; and tumor origin: pNET vs other origin).

Analyses will be based on the FAS population according to the randomized treatment group and strata assigned at randomization. The PFS distribution will be estimated using the Kaplan-Meier method, and Kaplan-Meier curves, median and associated 95% confidence intervals will be presented for each treatment group. The hazard ratio for PFS will be calculated, along with its 95% confidence interval, from a stratified Cox model using the same stratification factors as for the log-rank test.

In case of few events in some stratum, which may seriously impact the power, the primary analysis will instead be unstratified. More specifically, if any of the four strata counts less than eight PFS events, the primary analysis will be unstratified (Vittinghof and McCullugh, 2007). Both stratified and unstratified analyses will be presented.

More technical details concerning the analysis of PFS follow in Section 5.4.1.

2.5.3 Handing of intercurrent events of primary estimand

The analysis will account for different intercurrent events as explained in the following:

a. Discontinuation of study treatment for any reason before central PFS event: this intercurrent event is ignored (treatment policy strategy).

b. New anti-neoplastic therapy, including cross-over to Lutathera, before central PFS event: PFS is censored at the date of the last adequate tumor assessment prior to initiation of the new anti-neoplastic therapy (hypothetical strategy).

c. Re-treatment before central PFS event: PFS is censored at the date of the last adequate tumor assessment prior to re-treatment (hypothetical strategy).

2.5.4 Handling of missing values/censoring/discontinuations

This is an event-driven trial and the primary analysis for PFS will be performed after 99 PFS events have been documented based on central review of tumor assessments.

In the primary analysis, PFS will be censored at the date of the last adequate tumor assessment if no PFS event is observed prior to the analysis cut-off date.

Disease progressions (i.e., central review RECIST 1.1 documented disease progression) or deaths documented after the initiation of new anti-cancer therapy will not be counted as PFS events for the primary analysis.

The date of last adequate tumor assessment is the date of the last tumor assessment with overall response of CR, PR, or SD before an event or a censoring reason occurred. In this case, the last tumor evaluation date at that assessment will be used. If no post-baseline assessments are available (before an event or a censoring reason occurred) then the date of randomization will be used.

In particular, PFS will be censored at the last adequate tumor assessment if one of the following occurs: absence of event; the event occurred after two or more missing tumor assessments.

The term "missing adequate tumor assessment" is defined as a tumor assessment not performed or tumor assessment with overall response of "UNK". The rule to determine number of missing tumor assessments is based on the time interval between the date of last adequate tumor assessment and the date of an event. If the interval is greater than twice the protocol-specified interval between the tumor assessments and 2 times the protocol-allowed time window around assessments, then the number of missing assessments will be 2 or more.

Note that according to the protocol the first tumor assessment should occur at 16 weeks, then the second tumor assessment should occur 8 weeks later, and then the frequency should be every 12 weeks thereafter. For patients who discontinue from treatment without PD due to central review, tumor assessments should be taken every 12 weeks starting from the last tumor assessment until documented disease progression for central review per RECIST 1.1. Exact time windows to take into account missing assessments for PFS event determination is further discussed in Section 5.5.

Refer to Table 2.5-1 for censoring and event date options and outcomes for primary PFS.

Table 2.5-1 Derivation of primary PFS

Situation	Options	Outcome
A. No baseline assessment, or,<br>No post baseline assessment	Date of randomization	Censored
B. Progression or death at or before next scheduled assessment	Date of progression (or death)	Progressed
C. Progression or death after exactly one missing assessment	Date of progression (or death)	Progressed
D. Progression or death after two or more missing assessments	Date of last adequate assessment prior to missed assessment	Censored
E. No progression (or death)	Date of last adequate assessment	Censored
F. Treatment discontinuation due to 'Disease progression' without documented progression, i.e., clinical progression based on investigator claim	Ignore clinical progression and follow situations above	As per above situations
G. Start of new anticancer therapy, cross-over to or re-treatment with Lutathera prior to protocol defined progression per central review	Primary analysis: Censor patient at last adequate tumor assessment prior to new anti-cancer therapy	Censored
H. Death before first PD assessment	Date of death	Event

In particular, for censoring reason G in Table 2.5-1, new anticancer therapy is defined as one of the following:

- For new anti-cancer medications post-randomization:
  - Medications which are in the ATC2 class of Antineoplastic agents or
  - Medications which are in the ATC4 class of Various therapeutic radiopharmaceuticals

  - Class of medications of Somatostatin and Analogues excluding Octreotide and Octreotide Acetate
- For new anti-cancer procedures post randomization:
  - Any radiotherapies
  - Procedures (including surgery) with indication of Underlying disease in Concomitant non-drug therapies and interventions eCRF page

2.5.5 Sensitivity analyses

If there is a high rate of discrepancy (≥10%) between the strata classifications constructed using the electronic Case Report Form (eCRF) data and those obtained from the Interactive Response Technology (IRT), a sensitivity analysis will be performed for PFS per central assessment in which a stratified Cox regression model will be used to estimate the treatment hazard ratio and the associated 95% confidence interval based on the eCRF-derived strata. No other inferential statistics will be provided.

In addition, the hazard ratio and 95% confidence interval for PFS as per central assessment will also be obtained from an unstratified Cox model.

Finally, as sensitivity analyses performed in the FAS, the hazard ratio and 95% confidence interval for PFS per blinded independent central review will be obtained from:

- A stratified (using the randomization stratification factors) and covariate adjusted Cox model including covariates at baseline: Age (<65 years vs. ≥ 65 years), Gender, Race, Tumor burden at baseline, CgA, and SSTR tumor uptake score per central review. See Section 2.2.3 for more detailed definition of involved covariates. Time since diagnosis or metastasis will be taken as continuous variables in the model. All covariates will be included in the model regardless of their observed significance (p-value for given covariate).

2.5.6 Supplementary analyses

As supplementary analyses, PFS as per local review will be analyzed using a stratified Cox model, with the same analysis conventions as the primary efficacy analysis, and the treatment effect will be summarized by the hazard ratio with its 95% confidence interval. Kaplan-Meier curves, medians and 95% confidence intervals of the medians will be presented for each treatment group.

In addition, the primary efficacy endpoint will be summarized based on the data obtained below to assess different censoring mechanisms. Kaplan-Meier estimate of median PFS along with 95% confidence intervals, and hazard ratio and corresponding 95% confidence interval obtained using the Cox proportional hazards model will be provided. No other inferential statistics will be provided.

- Using the primary analysis source (i.e., central review using RECIST 1.1 criteria) on the FAS and including events whenever they occur, even after two or missing tumor assessments. The rule to determine number of missing tumor assessments is based on

the time interval between the date of last adequate tumor assessment and the date of event (see Section 5.5). If the interval is greater than twice the protocol-specified interval between the tumor assessment and two times the protocol-allowed time window around assessments, then the number of missing assessments will be two or more. In the summary tables, this approach is referred to as 'actual event PFS supplementary analysis'.

- Using the primary analysis source (i.e., central review using RECIST 1.1 criteria) on the FAS and backdating events that occurred after missing one or more tumor assessments (i.e., if progression/death is observed after one or more missing tumor assessments, it is considered as an event at the date of the next scheduled assessment following the last adequate tumor assessment. In the summary tables, this approach is referred to as 'backdating PFS supplementary analysis'.
- Using the primary analysis source (i.e., central review using RECIST 1.1 criteria) on the FAS and including events that occur after the start of new antineoplastic therapy if no PFS event is observed prior to the start of new antineoplastic therapy (not counting biopsies as a new antineoplastic surgery). In the summary tables, this approach is referred to as 'new anticancer PFS supplementary analysis'.

For these supplementary analyses, all analyses will be conducted using stratified Cox proportional hazards model. If there are not enough events in all strata, the analysis will be conducted using an unstratified Cox proportional hazards model.

Additionally, the following analyses will be performed:

- Timing of all tumor assessments according to RECIST 1.1 will be depicted graphically, separately for central radiology and investigator/local radiology and displaced by treatment group.
- Comparison of PFS event type/censor between local radiology review and central radiology assessment
- Number of participants with a PFS event and number of participants censored for the PFS analysis will be summarized. In addition, a summary of reasons for PFS censoring will be provided by treatment group. These summaries on censoring reasons will be produced for PFS by investigator radiology and central radiology.

Subgroup analyses for the primary endpoint

If the primary analysis is statistically significant, subgroup analyses to assess the homogeneity of the treatment effect across demographic, and baseline disease characteristics will be performed. Please refer to Section 2.2.3 for detailed subgroup definitions.

For each of the subgroups, the following analyses will be performed:

- Median Kaplan-Meier estimates of the survival distribution of PFS
- Hazard ratio with 95% CI using unstratified Cox proportional hazards model.

Efficacy analyses in subgroups are intended to explore the consistency (homogeneity) of treatment effect. Forest plot (including sample size/number of events and HR with 95% CI) will be produced to graphically depict the treatment effect estimates in different subgroups. No inferential statistics (p-values) will be produced for the subgroups.

Censoring pattern of PFS

The number of patients censored for the PFS analysis will be summarized. In addition, a summary for PFS censoring will be provided by treatment group based on the following reasons:

1. Ongoing without event
2. New cancer therapy added
3. Lost to follow-up
4. Withdrew consent
5. Adequate assessment no longer available
6. Event after ≥ 2 missing tumor assessments

The PFS censoring are defined in the following way:

If the time interval between the last adequate tumor assessment date and the earliest of the following dates is smaller or equal to interval of 2 missing tumor assessments:

1. Analysis cut-off date
2. Start date of further anti-neoplastic therapy
3. Date of consent withdrawal
4. Visit date of study treatment discontinuation or end of post-treatment follow-up discontinuation due to lost to follow-up

Then the PFS censoring reason will be:

1. 'Ongoing'
2. 'New cancer therapy added'
3. 'Withdrew consent'
4. 'Lost to follow-up'

If the time interval is larger than the interval of 2 missing tumor assessments with no event observed, then the PFS censoring reason will always default to 'Adequate assessment no longer available'. If the time interval between the last adequate tumor assessment and the PFS event date is larger than 2 missing tumor assessments, then the patient will be censored and the censored reason will be 'Event documented after two or more missing tumor assessments'.

These summaries on censoring reasons will be produced for PFS by central review and investigator using RECIST 1.1.

Waterfall plot to depict anti-tumor activity

Waterfall graphs will be used to depict the anti-tumor activity. These plots will display the best percentage change from baseline in the sum of diameters of all target lesions for each patient. Only patients with measurable disease at baseline will be included in the waterfall graphs. Special consideration is needed for assessments where the target lesion response is CR, PR or SD, but the appearance of a new lesion or a worsening of non-target lesions results in an overall lesion response of PD. As a conservative approach, such assessments will not be considered for display as bars in the graph, since the percentage change in the sum of diameters of target lesions reflects the non-PD target lesion response, but the overall lesion response is PD. A patient with only such assessments will be represented by a special symbol (e.g. ✶) in the waterfall graph. Assessments with "unknown" target lesion response and assessments with unknown overall response will be excluded from the waterfall plots. Patients without any valid assessments will be completely excluded from the graphs.

The total number of patients displayed in the graph will be shown and this number will be used as the denominator for calculating the percentages of patients with tumor shrinkage and tumor growth. Footnote will explain the reason for excluding some patients (due to absence of any valid assessment).

All possible assessment scenarios are described in Table 2.5-2.

Table 2.5-2 Inclusion/exclusion of assessments used in waterfall graph

	Criteria for inclusion/exclusion			Possible sources of contradictions	
Case	Target response	Overall lesion response	Include in waterfall?	Non-target response	New lesion?
1	CR/PR/SD	PD	Yes but as ✶ only	PD	any
2	CR/PR/SD	PD	Yes but as ✶ only	any	Yes
3	UNK	UNK or PD	No	any	any
4	CR/PR/SD	UNK	No	UNK	No
5	CR/PR/SD	CR/PR/SD	Yes as a bar	SD/IR	No
6	PD	PD	Yes as a bar	any	any

Concordance analysis of PFS using RECIST 1.1

Cross-tabulation of 'PFS by central review' vs 'PFS by investigator' by PFS event type (i.e., 'death', 'PD', 'censor' for each of the two sources resulting in a 3 by 3 table) and by treatment will be constructed to investigate discordance between the two sources on a patient-by-patient basis (see Table 2.5-3). Discordance rate between central radiology and investigator will be calculated and presented as % as follows: $100*(n_{13} + n_{23} + n_{31} + n_{32})/N$ by treatment group.

Table 2.5-3 Comparison of PFS using RECIST 1.1 between central review and investigator

	Central review PFS result

Investigator PFS	Death	PD	Censor
Death	n_{11}	n_{12}	n_{13}
PD	n_{21}	n_{22}	n_{23}
Censor	n_{31}	n_{32}	n_{33}

A cross-tabulation will be produced displaying the PFS timing for the local investigators' assessment compared to the central review assessment (see Table 2.5-4). For progression assessments, the frequency and percent of subjects with complete agreement, progression later, progression earlier, and cases where progression was called by one method and censored by the other will be displayed. Similarly, if censoring was recorded, the frequency and percent of subjects with complete agreement, censoring called later, censoring called earlier, and cases where censoring was called by one method and progression was called by the other method will be displayed.

Table 2.5-4 Comparison of PFS event times using RECIST 1.1 between central review and investigator assessments

Investigator	Central review	Treatment group (N=xxx)			
		Same time n (%)	Central review after investigator n (%)	Central review before investigator n (%)	Total
PD	PD	xx (xx.x)	xx (xx.x)	xx (xx.x)	xx (xx.x)
Death	Death	xx (xx.x)	0	0	xx (xx.x)
Censor	Censor	xx (xx.x)	xx (xx.x)	xx (xx.x)	xx (xx.x)
PD	Censor	xx (xx.x)	xx (xx.x)	xx (xx.x)	xx (xx.x)
PD	Death	0	xx (xx.x)	0	xx (xx.x)
Death	PD	0	0	xx (xx.x)	xx (xx.x)
Death	Censor	0	0	xx (xx.x)	xx (xx.x)
Censor	PD	xx (xx.x)	xx (xx.x)	xx (xx.x)	xx (xx.x)
Censor	Death	0	xx (xx.x)	0	xx (xx.x)
Total		xx (xx.x)	xx (xx.x)	xx (xx.x)	xx (xx.x)

Cross-over

PFS based on investigator assessment may be performed on the crossover set, if the number of PFS events will allow to perform such analysis. PFS will be defined as the time from the day of the first dose of crossover Lutathera therapy to the date of the first documented disease progression according to RECIST 1.1 as per investigator assessment, or death due to any

cause. Kaplan-Meier curve, median and its corresponding 95% CI of the survival distribution of PFS will be presented.

2.6 Analysis of the key secondary objectives

The key secondary objectives in this study are to:

1. demonstrate the superiority of Lutathera, compared to active comparator, in terms of objective response rate (complete + partial response)
2. demonstrate the superiority of Lutathera, compared to active comparator, in terms of time to deterioration in selected QoL items/scales

2.6.1 Key secondary endpoints

If the primary endpoint is significant, the key secondary endpoints of overall response rate (ORR) and QoL will be tested in a hierarchical fashion to protect the type I error rate. The order of the hypothesis testing shall be ORR followed by QoL Global Health Scale (TTD, see below), followed by QoL Diarrhea (TTD), QoL fatigue (TTD), and, QoL pain (TTD).

Overall response rate (ORR) is defined as the rate of patients with best overall response of either unconfirmed partial response (PR) or unconfirmed complete response (CR), as per central review and according to RECIST 1.1. ORR will be calculated based on the FAS according to the ITT principle. Only tumor assessments performed before the start of any further anti-neoplastic therapies (i.e., any additional anti-neoplastic therapy or surgery) will be considered in the assessment of best overall response. Also patients with non-measurable lesions per central review at baseline will contribute to the ITT analysis, see Table 2.6-1 below.

Table 2.6-1 Overall lesion response at each assessment: patients with non-target disease only

Non-target lesions	New lesions	Overall lesion response
CR	No	CR
Non-CR/Non-PD	No	Non-CR/Non-PD
UNK	No	UNK
PD	Yes or No	PD
Any	Yes	PD

EORTC QLQ-C30 includes 30 items. For more information regarding this instrument, see https://www.eortc.org/app/uploads/sites/2/2018/02/SCmanual.pdf . The following 9 scales and 6 single items are defined (where FS = Functional scale and SS = Symptom scale):

- Global health status / QoL (items 29 and 30)
- Physical functioning (FS) (items 1, 2, 3, 4 and 5)

- Role functioning (FS) (items 6 and 7)
- Emotional functioning (FS) (items 21, 22, 23 and 24)
- Cognitive functioning (FS) (items 20 and 25)
- Social functioning (FS) (items 26 and 27)
- Fatigue (SS) (items 10, 12 and 18)
- Nausea and vomiting (SS) (items 14 and 15)
- Pain (SS) (items 9 and 19)
- Dyspnoea (item 8)
- Insomnia (item 11)
- Appetite loss (item 13)
- Constipation (item 16)
- Diarrhoea (item 17)
- Financial difficulties (item 28)

For all scales the raw score (RS) is defined as the mean of the respective items:

$$RS = (item_{i_1} + item_{i_2} \ldots + item_{i_n})/n$$

For a single item, RS is identical to the score of the item itself.

For the functional scales (FS), the score is calculated by the formula:

Score = {1 – (RS – 1)/range} x 100.

For the symptom scales (SS), the single items and Global health status /QoL the score is calculated by the formula:

Score = {(RS – 1) / range} x 100,

For a single item, RS is identical to the score of the item itself.

The “range” value is the difference between the maximum possible value of RS and the minimum possible value for RS. The range = 3 for all scales and single items except for “Global health status /QoL”. For “Global health status /QoL” the range is 6.

The formulas for the scores are linear transformations of 0-100.

Time to deterioration (TTD) is the main analysis and is defined as time from randomization to the first deterioration of at least 10 points in domain score compared to the baseline score for the same domain or death while on treatment (Strosberg *et al.* 2018). Patients with no deterioration are censored at the last QoL assessment date. Patients with no baseline and/or no follow-up are censored at randomization.

2.6.2 Statistical hypothesis, model, and method of analysis

ORR and its 95% confidence interval will be presented by treatment group.

The null hypothesis of the ORR in the investigational arm is less than or equal to the ORR in the control arm will be tested against the one-sided alternative. The statistical hypotheses are:

$$H_{02}: ORRR \leq ORRC \text{ versus } H_{A2}: ORRR > ORRC, \text{ for a one-sided test}$$

where ORRR is the probability of response in investigational arm and ORRC is the probability of response in control arm.

The Cochran-Mantel-Haenszel chi-square test, stratified by the randomization stratification factors, will be used to compare ORR between the two treatment groups, at the 1-sided 2.5% level of significance. More details concerning the calculation are provided in Section 5.4.2.

The analysis of QoL Time to Deterioration (TTD) will be the same as for PFS, employing log-rank test and Cox regression, see above, and, Appendix for further details.

Assuming proportional hazards model for TTD, the null hypothesis will be tested at one-sided 2.5% level of significance:

$$H_{01} \text{ (null hypotheses): } \Theta_1 \geq 0 \text{ vs. } H_{a1} \text{ (alternative hypotheses): } \Theta_1 < 0,$$

where Θ_1 is the log hazard ratio of TTD in the Lutathera plus Standard Dose octreotide long-acting (30 mg) (investigational) arm vs. High Dose octreotide long-acting (60 mg) (control) arm.

For each domain, a Kaplan-Meier plot will be produced showing time to event by treatment arm. Kaplan-Meier methods will be used to generate a point estimate of the median time to event with corresponding 95% confidence interval (CI).

The stratified log-rank test will be used to compare the time to event in the two groups at the one-sided 2.5% level. The hazard ratios (HR) and corresponding 95% CIs were estimated from a stratified Cox proportional hazards model including randomized treatment as a factor. If too few events occur in any stratum (less than eight), then the main analysis will be unstratified. Both stratified and unstratified analyses will be presented.

2.6.3 Handing of intercurrent events of secondary estimands

Secondary estimand #1 (ORR)

The analysis will account for different intercurrent events as explained in the following:

a. Discontinuation of study treatment for any reason before response: this event is ignored (treatment policy strategy).
b. New anti-neoplastic therapy, including cross-over to Lutathera, before response: the participant is considered as non-responder (composite strategy).

c. Re-treatment before response: the participant is considered as non-responder (composite strategy).

Secondary estimand #2 (TTD)

The analysis will account for different intercurrent events as explained in the following:

a. Discontinuation of study treatment for any reason before deterioration: QoL assessments collected on the randomized study treatment will be used in the analysis. Assessments collected after end of study treatment will be excluded from the analysis (While on treatment strategy).

b. New anti-neoplastic therapy, including cross-over to Lutathera, before deterioration: TDD is censored at the date of the last assessment prior to initiation of the new anti-neoplastic therapy (hypothetical strategy).

c. Re-treatment before deterioration: TDD is censored at the date of the last assessment prior to re-treatment (hypothetical strategy).

d. Use of symptomatic medications associated with the considered scale/item before deterioration: this intercurrent event is ignored (treatment policy strategy).

2.6.4 Handling of missing values/censoring/discontinuations

Secondary estimand #1 (ORR)

Patients with unknown or missing best overall response (BOR) will be counted as failures, i.e., non-responders. Patients who were non-responders before initiation of subsequent anti-cancer therapy will still be non-responders. If there is no baseline tumor assessment, all post-baseline overall lesion responses are expected to be 'Unknown'. If no valid post-baseline tumor assessments are available, the best overall response must be "Unknown". For the computation of ORR, these patients will be included in the FAS and will be counted as 'failures'.

Secondary estimand #2 (TTD)

Missing items in scores will be imputed by the following method:

For QoL scores, if at least half of the items from the scale have been answered then use all the items that were completed and apply the standard equation for calculating the Raw Score (RS). No imputation of missing booklets will be performed.

The assignment of event/censoring will follow the scheme in Table 2.6-2 below.

Table 2.6-2 Derivation of TTD

Situation	Options	Outcome
A. No baseline or post-baseline assessment	Date of randomization/start of treatment	Censored

B. Deterioration at or before next scheduled assessment	Date of assessment	Event
C. Deterioration after exactly one missing assessment	Date of assessment	Event
D. Deterioration after two or more missing assessments	Date of last adequate (with at least half of items from appropriate scale completed) assessment	Censored
E. No deterioration	Date of last adequate (with at least half of items from appropriate scale completed) assessment	Censored

2.6.5 Sensitivity analyses

Secondary estimand #1 (ORR)

If there is a high rate of discrepancy (≥10%) between the strata classifications constructed using the electronic Case Report Form (eCRF) data and those obtained from the Interactive Response Technology (IRT), a sensitivity analysis will be performed for ORR, in which Odds ratio of ORR between treatment groups along with 95% CIs will be provided using stratified CMH method based on the eCRF-derived strata. No other inferential statistics will be provided.

Secondary estimand #2 (TTD)

If there is a high rate of discrepancy (≥10%) between the strata classifications constructed using the electronic Case Report Form (eCRF) data and those obtained from the Interactive Response Technology (IRT), a sensitivity analysis will be performed for TTD in which a stratified Cox regression model will be used to estimate the treatment hazard ratio and the associated 95% confidence interval based on the eCRF-derived strata. No other inferential statistics will be provided.

In addition, the hazard ratio and 95% confidence interval for TTD will also be obtained from an unstratified Cox model.

2.6.6 Supplementary analyses on Key secondary endpoints

Secondary estimand #1 (ORR)

As a supplementary analysis, ORR with unconfirmed response based on the FAS will be summarized using descriptive statistics (N, %) by treatment group along with two-sided exact binomial 95% CIs for the following calculations:

- ORR with unconfirmed response per local review based on the FAS

A concordance summary table will be generated to compare the best overall response based on central vs. local review.

Patients with best overall response “unknown” will be summarized by reason for having unknown status. The following reasons will be used:

- No valid post-baseline assessment
- All post-baseline assessments have overall response UNK
- New anti-neoplastic therapy started before first post-baseline assessment
- SD too early (≤15 weeks after randomization)
- PD too late (>25 weeks after randomization and not qualifying for CR, PR, SD or non-CR/non-PD)

Special (and rare) cases where BOR is unknown due to both early SD and late PD will be classified as “SD too early”.

Subgroup analyses for ORR

If the primary analysis and ORR is statistically significant, subgroup analyses to assess the homogeneity of the treatment effect across demographic and baseline disease characteristics will be performed. Please refer to Section 2.2.3 for detailed subgroup definitions.

For each of the subgroups, the following analyses will be performed:

- Number and percentage of participants with unconfirmed CR, unconfirmed PR;
- ORR with two-sided exact binomial 95% CIs for each treatment group;
- Odds ratio of ORR between treatment groups along with 95% CIs using stratified CMH method, where IRT stratification factors will be used.

Efficacy analyses in subgroups are intended to explore the consistency (homogeneity) of treatment effect.

Secondary estimand #2 (TTD)

As supplementary analyses, the secondary estimand endpoint of QoL Global Health Scale (TTD, see below), followed by QoL Diarrhea (TTD), QoL fatigue (TTD), and, QoL pain (TTD) will be summarized based on the data obtained below to assess different censoring mechanisms. Kaplan-Meier estimands of median TTD of the specific QoL scales discussed above, along with 95% confidence intervals, and hazard ratio and corresponding 95% confidence interval obtained using the Cox proportional hazards model will be provided. No other inferential statistics will be provided.

- Using the primary analysis source on the FAS and including TTD events whenever they occur, even after two or missing QoL assessments. In the summary tables, this approach is referred to as ‘actual event TTD supplementary analyses’.

For this supplementary analyses, all analyses will be conducted using stratified Cox proportional hazards model. If there are not enough events in all strata, the analysis will be conducted using an unstratified Cox proportional hazards model.

Quality of life will also be assessed in terms of change from baseline through Mixed Model Repeated Measures (MMRM), which are outside the scope of the primary analysis, and will be conducted in the final analysis.

Subgroup analyses for TTD

If the primary analysis is statistically significant, as well as the key secondary ORR analysis and the TTD QoL analyses are statistically significant, to assess the homogeneity of the treatment effect across demographic and baseline disease characteristics, subgroup analysis for TTD will be performed. Please refer to Section 2.2.3 for detailed subgroup definitions.

For each of the subgroups, the following analyses will be performed:

- Median Kaplan-Meier estimates of the survival distribution of TTD
- Hazard ratio with 95% CI using unstratified Cox proportional hazards model.

Efficacy analyses in subgroups are intended to explore the consistency (homogeneity) of treatment effect. Forest plot (including sample size/number of TTD events and HR with 95% CI) will be produced to graphically depict the treatment effect estimates in different subgroups. No inferential statistics (p-values) will be produced for the subgroups.

2.7 Analysis of other secondary and exploratory objective(s)

Other secondary objectives of the study are to compare the two treatment groups with respect to:

1. DCR: Disease Control Rate, the proportion of patients with a best overall response of CR, PR, or SD per central review. Timing: at the primary analysis of PFS.
2. DOR: Duration of Response per central review, time from first complete or partial response to progression or death due to underlying cancer. Timing: at the primary analysis of PFS.
3. OS: time from the randomization date until the day of death due to any cause. Timing of final analysis: when all patients have either had an event, are lost to follow-up, or, four years have elapsed from the randomization of the last patient or six months after the last cross-over or re-treatment dose in the study, whichever occurs last.
4. Rate of adverse events and laboratory toxicities (scored according to CTCAE grade). See Section 2.8 for safety analyses.

Further, these exploratory endpoints are also covered in this section:

- Time to second progression (PFS2), the time from randomization to objective tumor progression on next line treatment or death due to any cause, assessed locally according to RECIST 1.1
- Health related quality of life (QoL) scales, TTD for items/scales not included among key secondary endpoints as well as change from baseline in the total score for health status from the EQ-5D questionnaire.

- PK of octreotide long-acting at the high dose of 60 mg by examining the steady-state trough plasma concentration of octreotide.

2.7.1 Other Secondary and Exploratory endpoints

Disease control rate (DCR)

Disease control rate (DCR) is the proportion of patients with a best overall response of CR, PR, or SD lasting 15 weeks or longer (corresponds to the schedule of the first tumor assessment [16±1 week]). A patient will be considered to have SD for 15 weeks or longer if a SD (or NCR/NPD) response is recorded at 15 weeks or later from randomization. A patient is consider to have PD if the PD occurred at or before week 25, and not qualifying for CR, PR, and SD. The objective of this endpoint is to summarize patients with signs of "activity" defined as either shrinkage of tumor (regardless of duration) or slowing down of tumor growth. DCR will be calculated using the FAS for both central review and investigator tumor assessments.

DCR will also be calculated during the re-treatment period for those that are randomized to the Lutathera arm, and then re-treated with Lutathera. In this case, DCR is the proportion of patients with a best overall response of CR or PR or SD lasting 15 weeks or longer after 1st re-treatment dose of Lutathera, and will be assessed using investigator tumor assessments.

Duration of response

The Duration of Response (DOR), for participants with BOR of unconfirmed CR or unconfirmed PR according to RECIST 1.1, is defined as the time from initially meeting the criteria for response (unconfirmed CR or unconfirmed PR) until the time of progression by RECIST 1.1 or death due to underlying cancer. DOR will be reported descriptively for each group without comparison between groups. The censoring rules in Table 2.5-1 apply. DOR will be calculated using the subset of responders in the FAS for both central review and investigator tumor assessments.

DOR will also be calculated during the re-treatment period for those that are randomized to the Lutathera arm, and then re-treated with Lutathera. In this case, DOR is the defined as the time from initially meeting the criteria for response after receiving re-treatment with Lutathera (CR or PR) until the time of progression by RECIST 1.1 (locally assessed) or death due to underlying cancer.

Overall Survival (OS)

The time from the randomization date until the day of death due to any cause will be analysed with three different approaches, that all employ Cox regression to obtain the hazard ratio and its 95% confidence interval.

1. ITT approach, which analyses data with treatment allocated according to randomization
2. Rank Preserving Structural Failure Time model (RPSFT), which uses an acceleration factor to adjust for any cross-over from Octreotide to Lutathera or for re-treatment of Lutathera for those in the investigational arm; this implies a modification of the

proportional hazards model, see Section 5.4.1. This approach will be analyzed in the final study analysis, and not in the primary PFS analysis.

3. IPCW, which uses Inverse Probability of Censoring Weighting to adjust for any cross-over from Octreotide to Lutathera or for re-treatment of Lutathera for those in the investigational arm, see Section 5.4.1. This approach will be analyzed in the final study analysis, and not in the primary PFS analysis.

ITT approach is primary. Approaches 2 and 3 are outside the scope of the primary analysis, and will be conducted in the final analysis. The analysis proceeds by Cox regression as in the primary analysis of PFS.
Reasons for censoring ('Alive' or 'Lost to follow-up') and death cause will be listed by treatment arm. Survival status, reason for censoring and death cause will be listed. Patients not known to have died will be censored for 'Loss to follow-up' if the time between their last contact date and the analysis cut-off date is longer than 12 months + 2 months window (i.e. the planned interval between two OS follow-up visits in follow up plus the 1-month window on either side).

Time to second progression (PFS2)

As additional exploratory end-point, the Time to Second Progression (PFS2) will be assessed in the two study arms. PFS2 is defined as the time from randomization to the first documented progression after the start of next-line anti-neoplastic therapy or death from any cause, whichever occurs first. The first documented progression on next-line treatment is based on investigator RECIST assessment of PD. It is recognized that the limitation due to potential cross-over or re-treatment and the lower scans frequency during follow-up will reduce the possibility to reach firm conclusions on PFS2.

- Next-line therapy is defined as the first new (systemic) anti-neoplastic therapy initiated after discontinuation of study treatment regardless of the discontinuation reason. Drugs given as part of the same regimen should be considered as one line (i.e., part of the next-line therapy).
- Re-treatment with Lutathera (additional cycles after completion of the 4 cycle treatment on study) will count as next-line anticancer therapy.
- Crossover therapy with Lutathera will also count as next-line anticancer therapy
- PFS2 will be censored if no PFS2 event (progression or death) is observed after the start of next line anti-neoplastic therapy and before the analysis cut-off date; the censoring date will be the date of last scan.
- Any death prior to initiation of next-line therapy will be considered as an event for PFS2. Any death occurring following end of next line therapy will be considered as an event for PFS2 if no second new anti-neoplastic therapy has been introduced. In case that death is more than one year since last scan it will be censored at the time of the last available scan.

As an exploratory long-term endpoint for efficacy, PFS2 analysis will not be performed at the time of the primary PFS analysis, but it will be summarized in the final analysis when data is more mature.

Exploratory PFS and ORR analysis in re-treatment Lutathera patients

Additionally, for those that receive re-treatment with Lutathera during the re-treatment phase after receiving Lutathera treatment in the treatment phase, ORR and PFS will be assessed during the re-treatment phase for the final study analysis. The last scan per investigator assessment before the beginning of re-treatment will act as the baseline assessment for assessing ORR and PFS in the re-treatment period.

- ORR (Re-treatment): Rate of unconfirmed complete and unconfirmed partial responses after receiving re-treatment with Lutathera (CR, PR) which is locally assessed according to RECIST 1.1
- PFS (Re-treatment): Time from the 1st dose of re-treatment with Lutathera to objective tumor progression (locally assessed according to RECIST 1.1) or to death due to any cause.

Karnofsky Performance Status

The Karnofsky Performance Status (KPS) scale is an assessment tool for functional impairment, ranging from 0 (death) to 100 (normal). Shift table by KPS scale categories (<50, 50-60, 70-80, 90-100) to compare baseline to the worst post-baseline value will be generated.

QoL Items/Scales

TTD for Items/Scales derived from those EORTC QLQ-C30 subscales not included among key secondary endpoints will be analyzed as exploratory endpoints. Additionally, change from baseline in the total score for health status using GI.NET21 and EQ-5D questionnaire will be explored.

EORTC QLQ-GI.NET21 includes 21 items. For more information regarding this instrument, see Yadegarfar et al (2013). The following 5 scales and 4 single items are defined:

- Endocrine scale (items 31, 32 and 33)
- G.I. scale (items 34, 35, 36, 37 and 38)
- Treatment scale (items 39, 40 and 46)
- Social function scale (items 42, 44 and 49)
- Disease related worries scale (items 41, 43 and 47)
- Single item 1: Muscle/bone pain symptom (item 48)
- Single item 2: Sexual function (item 51)
- Single item 3: Information/communication function (item 50)
- Single item 4: Body image (item 45)

Similar as EORTC QLQ-C30, for all scales the Raw Score (RS) is defined as the mean of the respective items:

$$RS = (item_{i_1} + item_{i_2} \ldots + item_{i_n})/n$$

For a single item, RS is identical to the score of the item itself.

For the scales and the single item, the score is calculated by the formula:

Score = {(RS – 1) / range} x 100

Where "range" is the difference between the maximum possible value of RS and the minimum possible value for RS. The range = 3 for all scales and single items.

The formulas for the scores are linear transformations to 0-100.

Missing items in scores will be imputed by the following method:

If at least half of the items from the scale have been answered then use all the items that were completed and apply the standard equation for calculating the Raw Score (RS). No imputation of missing booklets will be performed.

The EQ-5D-5L consists of the EQ-5D descriptive system and the EQ visual analogue scale (EQ-VAS). The EQ-5D-5L descriptive system comprises the following 5 dimensions: mobility, self-care, usual activities, pain/discomfort, and anxiety/depression. The EQ-VAS records the respondent's self-rated health on a vertical, visual analogue scale.

Each of the five dimension scales contain five levels, with level 1 indicating no problems, level 2 indicating slight problems, level 3 indicating moderate problems, level 4 indicating severe problems, and level 5 indicating unable to/extreme problems.

The EQ-VAS is scored by assigning an integer value, ranging from 0 (Worst imaginable health state) to 100 (Best imaginable health state), corresponding to the mark placed by the patient on the VAS. Ambiguous answers (e.g., two marks placed on the scale by a patient) should be treated as missing values.

A utility score will be obtained by using a weighted combination of the levels of the five dimension scales. The weights are based on value sets which are country-specific. Each patient's 5 digit health states code (response to question 1,2,3,4, and 5 concatenated (ex., 41325 results in a utility score of 0.193)) is converted to a utility score using the EQ-5D-5L value set, available in the cross-walk index value calculator which can be downloaded from the web site https://euroqol.org/eq-5d-instruments/eq-5d-5l-about/valuation-standard-value-sets/crosswalk-index-value-calculator/ . (Scroll to the bottom of the web page and download the Excel file. Use the sheet labelled 'EQ-5D-5L Value Sets.')

Since utility score depends on the combination of all items' responses, any missing response results in a missing utility score. If a patient dies, for analysis he will be assigned a score of 0 on the date of death.

Steady-state trough plasma concentration of octreotide

The analysis of this exploratory endpoint is further explained in Section 2.9.

2.7.2 Statistical hypothesis, model, and method of analysis

DCR: DCR per either central or local assessment will be summarized using descriptive statistics (N, %) by treatment group along with two-sided exact binomial 95% CIs.

DOR: DOR will be listed and summarized for patients with BOR of CR or PR (unconfirmed). The distribution of duration of response will be estimated using the Kaplan-Meier method and the median duration of response will be presented along with 95% confidence interval. In addition, a swimmer plot will show the time to onset and duration of response based on central review for all patients with unconfirmed CR or unconfirmed PR.

PFS2: PFS2 analyses will be based on the FAS population according to the randomized treatment group and strata assigned at randomization. The PFS2 distribution will be estimated using the Kaplan-Meier method, and Kaplan-Meier curves, median and associated 95% confidence intervals will be presented for each treatment group.

For the efficacy analysis in the re-treatment phase, all efficacy analysis (ORR, DOR, DCR, PFS, PFS2, OS) will be descriptive in nature only.

2.7.3 Handling of missing values/censoring/discontinuations

Time to second progression (PFS2)

The rules for censoring and event for PFS2 are summarized in Section 2.7.1.

Overall Survival (OS)

If a patient is not known to have died at the time of analysis cut-off, then OS will be censored at the date of last known patient was alive, i.e., last contact date (see Table 2.1-2).

2.8 Safety analyses

For the treatment phase, all safety analyses will be based on the safety set. Separate safety analyses will be performed for patients during the crossover and re-treatment phase as appropriate.

2.8.1 Adverse events (AEs)

All adverse events (AEs), whether or not spontaneously reported by the patient, will be recorded starting from the signing of the ICF until the end of the randomized period/cross-over period/re-treatment period. During the Follow up Phase, only related serious adverse events and adverse events of special interest will be recorded, except for secondary hematological malignancies which will be recorded irrespective of relationship to Lutathera, Octreotide, and amino acid. The data provides information regarding relatedness to Lutathera, Octreotide and amino acid.

AEs that started or worsened during the on-treatment period (including randomized treatment, cross-over and re-treatment periods as defined in Section 2.1.1.10) is defined as treatment-emergent AEs (TEAE). The primary AE summaries will focus on all treatment-emergent AEs in the randomized treatment period that started or worsened during the randomized treatment

period, defined as from date of first administration of study treatment to 30 days after the date of the last actual administration of any study treatment in the randomized period before cross-over or retreatment. However, selected AE summaries will also be generated for cross-over and re-treatment periods separately. All AEs collected in the eCRF page will be listed along with the information collected e.g., AE relationship to study drug, AE outcome etc. In addition, each AE record in the listings will be flagged for its study period based on the start date.

AEs will be summarized by number and percentage of subjects having at least one AE, having at least one AE in each primary system organ class (SOC) and for each preferred term (PT) using MedDRA coding. A subject with multiple occurrences of an AE will be counted only once in the respective AE category. A subject with multiple CTCAE grades for the same preferred term will be summarized under the maximum CTCAE grade recorded for the event. AE with missing CTCAE grade will be included in the 'All grades' column of the summary tables.

In AE summaries, the primary SOC will be presented alphabetically and the PT will be sorted within primary SOC in descending frequency. For randomized period AE summaries, the sort order for the PT will be based on their frequency in the investigational arm.

The following AE summaries will be produced by treatment arm; overview of AEs and deaths (number and % of subjects who died, with any AE, any SAE, any dose reductions/interruptions etc), AEs by SOC and PT, summarized by relationship (all AEs and AEs related to study treatment), seriousness (SAEs and non-SAEs), leading to treatment discontinuation, leading to dose reduction/interruption, requiring additional therapy and leading to death (both related to study treatment and irrespective of study treatment). In addition, a summary of SAEs with number of occurrences will be produced (an occurrence is defined as >1 day between start and prior end date of record of same preferred term).

2.8.1.1 Adverse events of special interest / grouping of AEs

An adverse event of special interest (AESI) is a grouping of adverse events that are of scientific and medical concern specific to Lutathera.

Certain AESIs in this study are flagged in the eCRF by the investigator within the adverse event form, and the categories for Lutathera are included in Table 2.8-1 below:

Table 2.8-1 Adverse events of special interest

AESI categories	Special consideration regarding grade
Immediate hematotoxicities	For Haematopoietic thrombocytopenia (SMQ), only include Grade >=2; for others only include Grade >=3.
Cardiovascular and Electrolyte Disorder	Only include Grade >=3.
Secondary hematological malignancies	
Nephrotoxicities	

To be conservative and align within the Lutathera NET program, these groupings are re-derived using MedDRA terms, SMQs (standardized MedDRA queries), HGLTs (high level group terms), HLT (high level terms) or PT. Customized SMQs (Novartis MedDRA queries, NMQ) may also be used. A NMQ is a customized group of search terms which defines a medical concept for which there is no official SMQ available or the available SMQ does not completely fit the need. It may include a combination of single terms and/or an existing SMQ, narrow or broad.

The grouping of AEs in AESI according to project standards will be specified in the electronic Case Retrieval Sheet (eCRS) and may be regularly updated. In addition, further rules regarding grade (see Table 2.8-1) will be implemented for the groupings of Hematotoxicities, Cardiovascular and electrolyte disorder.

Data analysis of AESIs

The re-derived AESI groupings using eCRS will be used in the analyses. Summaries of AESIs will be provided by treatment arm (specifying grade, seriousness, relationship, leading to treatment discontinuation, leading to dose reduction/interruption, death etc.). Separate summaries for AESIs will be provided for the randomized treatment period, and the overall study period. An analysis of time to first occurrence of each type of AESIs will be performed for the randomized treatment period. In addition, time to first occurrence will also be performed for long-term AESIs for patients who have been treated with Lutathera (see below for more details).

Time to first occurrence in the randomized treatment period

Median time to first occurrence and 95% CI will be provided based on the Kaplan-Meier method. The event free rate at selected timepoints will be also estimated with 95% CI. In addition, Kaplan-Meier plots will be generated.

Time to first occurrence of AESI is defined as the time from the start of treatment to the start date of the first occurrence of any AE under the specific AESI in the randomized treatment period, i.e. time in days is calculated as (start date of first occurrence in the randomized treatment period) – (date of first dose of study treatment) +1. A participant will be censored if:

- The participant did not report any post-baseline AESI on or before the analysis cut-off date in the randomized treatment period.
- The participant discontinued randomized treatment without reporting the specific AESI in the randomized treatment period.
- The participant died without reporting the specific AESI in the randomized treatment period.
- The participant received a new anticancer therapy before reporting the specific AESI in the randomized treatment period.

The censoring date will be the earliest of the following dates: end date in the randomized treatment period, analysis cut-off, new anti-cancer therapy start date, death date and last contact date.

In addition, the median time to first occurrence for the subset of participants who experienced the event of interest will be calculated. Simple descriptive statistics, median, min and max as well as 25th percentile and 75th percentile, will be presented.

Time to first occurrence during the study

For long-term AESIs that can potentially occur after study treatment during follow-up, similar analysis as above will be repeated to consider all study periods, including randomized treatment period and any subsequent periods during the study. Such AESI includes secondary hematological malignancies and nephrotoxicity. This analysis is only for patients who have been treated with Lutathera, i.e., patients who were treated in the Lutathera arm and patients who did cross-over from the control arm. For cross-over patients, the time to first occurrence of AESI is counted from the start of cross-over to Lutathera instead of the randomized treatment and it will be summarized separately from the Lutathera arm.

Events/censoring rules are same except that all assessments in the study will be used regardless of study periods. The censoring date will be the earliest of the following dates: analysis cut-off, death date and last contact date in the study.

2.8.2 Deaths

Overview of death will be summarized separately for randomized period, cross-over period, re-treatment period and post-treatment follow up period. All deaths in the randomized treatment period will be summarized by treatment arm, and by SOC and PT if the primary reason of death is AE(s). All deaths during the study will be listed and flagged by treatment period.

2.8.3 Laboratory data

The laboratory assessments require that blood samples for hematology and blood chemistry, and a urine sample for urinalysis are taken (see Table 8-2, Table 8-3, and Table 8-4 of the study protocol). Laboratory assessments will be performed at the investigational site.

The following laboratory parameters will be investigated and analyzed:

(1) Haematology: White blood cells (WBC) with differential (i.e., Lymphocytes, Monocytes, Neutrophils, Eosinophils, Basophils), Platelets, Haemoglobin (Hb), Haematocrit, MCV

(2) Blood chemistry: Blood urea nitrogen (BUN) or Urea, Serum creatinine, Uric acid, Albumin, Total bilirubin, ALP, AST/ASAT, ALT/ALAT, Gamma-GT, Sodium, Potassium, LDH, CgA (centralized assessment), Haemoglobin A1C (GlycoHb), fT4, Calcium, Magnesium, Fasting blood glucose, Thyrotropin, Creatinine clearance (calculated by the Cockroft-Gault formula)

(3) Pregnancy test (if applicable)

On analyzing laboratory, data from all sources (central and local laboratories) will be combined. The summaries will include all assessments available for the lab parameter collected no later than 30 days after the last study treatment administration date (see Section 2.1.1). Separate

summaries will be generated for the randomized period, cross-over period, and re-treatment period.

Laboratory values outside normal ranges will be listed and flagged. The following flags will be used:

'+': 'Higher than reference value'

'-': 'Lower than reference value'

The respective parameter and the flagged value will be presented patient-wise by visit together with the reference values

The following summaries will be produced for hematology and biochemistry laboratory data (by laboratory parameter and treatment):

- Worst post-baseline CTC grade (regardless of the baseline status). Each subject will be counted only for the worst grade observed post-baseline.
- Shift tables using CTC grades to compare baseline to the worst on-treatment value
- For laboratory tests where CTC grades are not defined, shift tables using the low/normal/high/(low and high) classification to compare baseline to the worst on-treatment value.
- Box plots of laboratory values by scheduled time point.

The following listings will be produced for the laboratory data:

- Listings of all laboratory data, with CTC grades and classification relative to the laboratory normal range. Lab data collected during the post-treatment period will be flagged.
- Listing of all CTC grade 3 or 4 laboratory toxicities

Liver toxicity parameters

Liver parameters of interest are Total Bilirubin (TBIL), Alanine Transaminase (ALT), Asparate Aminotransferase (AST) and Alkaline Phosphatase (ALP). The number (%) of patients with peak post-baseline values and combined elevations post-baseline will be summarized as follows:

- ALT > 3x ULN, 5x ULN, 20x ULN
- AST > 3x ULN, 5x ULN, 20x ULN
- ALT or AST > 3x ULN, 5x ULN, 20x ULN
- ALP ≥ 2x ULN, 3x ULN, 5x ULN, 8xULN, 10x ULN
- TBIL > 2xULN, 3x ULN, 5xULN, 10x ULN

For the following combined categories, the assessments need not be concurrent, i.e., participants are counted based on the most extreme value for each parameter.

If AST and ALT ≤ ULN at baseline:

- ALT or AST >3x ULN & TBL >2x ULN
- ALT or AST >3x ULN & TBL >2x ULN & ALP ≥2xULN
- ALT or AST >3x ULN & TBL >2x ULN & ALP <2xULN

If AST or ALT > ULN at baseline

- Elevated ALT or AST (>3x Baseline value or 8x ULN) and TBIL (>2x Baseline value and 2x ULN)
- Elevated ALT or AST (>3x Baseline value or 8x ULN) and TBIL (>2x Baseline value and 2x ULN) and ALP ≥ 2x ULN
- Elevated ALT or AST (>3x Baseline value or 8x ULN) and TBIL (>2x Baseline value and 2x ULN) and ALP < 2x ULN

Further medical review has to be conducted to assess potential confounding factors such as, liver metastases, liver function at baseline etc.

A figure displaying time course of hepatic injury tests (ALT, AST, TBL, ALP) in patients with Hy's law will be displayed in the Safety Set. Additionally, evaluation of Drug-Induced Serious Hepatotoxicity (eDISH) plots will be produced to display ALT and AST values by TBL values in units of ULN.

Depending on availability of data, plots of hepatic tumor volume against ALT, AST, or TBL values in units of ULN may be generated.

Separate displays will be generated for the randomized period, cross-over period, and re-treatment period.

2.8.4 Other safety data

2.8.4.1 ECG data

ECG data will be summarized by presenting summary statistics of the raw data and change from baseline by treatment group and time point. Notable ECG values will be summarized for the randomized treatment period. All ECG data will be included in listings, and those beyond the randomized treatment period will be flagged by study period.

Notable elevations of ECG summarize the number of participants meeting or exceeding predefined limits in terms of absolute QT/QTc interval data/PR/RR/QRS or changes from baseline as defined in Table 2.8-2, and notable elevations of ECG includes only newly occurring ECG abnormality. A newly occurring ECG abnormality is defined as an abnormal post-baseline ECG finding that is not present at baseline. The percentage of participants having notable ECG interval values is based on the number of participants at risk for the change with a value at baseline and post-baseline.

Table 2.8-2 Clinically notable ECG values

ECG parameter (unit)	Clinically notable criteria
QT, QTcF (msec)	New > 450 and ≤ 480<br>New > 480 and ≤ 500<br>New > 500<br>Increase from Baseline > 30 and ≤ 60<br>Increase from Baseline > 60
PR duration (msec)	Increase > 25% from baseline and to PR duration > 200<br>New > 200
QRS duration (msec)	Increase > 25% from baseline and to QRS duration > 120<br>New > 120
Heart rate (beats/min)	<50 and decrease from baseline of > 25%<br>> 100 and increase from baseline of > 25%

All ECG data will also be listed by treatment group, participant, and visit. Abnormalities will be flagged.

An ECG in triplicate (at least 5 minutes apart) is recorded with the patient supine, after 5-minute rest, and not immediately after a meal. The parameters will be measured as a mean value of minimally 3 beats; the mean of each parameter will be used for statistical analysis. If an ECG is conducted, but data is not available in triplicate, the average of the ECG parameters at that assessment by (un)scheduled visit should be used in the analysis.

Frequency tabulations for the ECG interpretation (Normal / Abnormal, not clinically significant / Abnormal, clinically significant) will be presented.

2.8.4.2 Vital signs

Vital signs assessments are performed in order to characterize basic body function. The parameters expected to be collected include: weight, pulse rate, and systolic and diastolic blood pressure. Vital sign data will be summarized for randomized, cross-over, and re-treatment periods, separately.

The criteria for clinically notable abnormalities are defined in Table 2.8-3 below:

Table 2.8-3 Clinically notable changes in vital signs

Vital sign (unit)	Clinically notable criteria	
	**Above normal value**	**Below normal value**
Systolic blood pressure (mmHg)	≥ 180 with increase from baseline of ≥ 20	≤ 90 with decrease from baseline of ≥ 20
Diastolic blood pressure (mmHg)	≥ 105 with increase from baseline of ≥ 15	≤ 50 with decrease from baseline of ≥ 15

Pulse rate (bpm)	≥ 100 with increase from baseline of ≥ 25%	≤ 50 with decrease from baseline of ≥ 25%
Weight (kg)	increase ≥ 10% from baseline	decrease ≥ 10% from baseline

The following summaries will be produced for each vital sign parameter by treatment group:

- Number of percentage of participants with at least one post-baseline vital sign abnormality (in both directions, i.e., both elevated and below normal values)

In addition, the following two listings will be produced by treatment group:

- Participants with clinically notable vital sign abnormalities
- All vital sign assessments will be listed by participant and vital sign parameter

In both listings, the clinically notable values will be flagged and also assessments collected later than 30 days after the last treatment date (for those who receive cross-over or re-treatment therapy it would be 30 days after that last treatment date) will be flagged.

2.8.4.3 Additional Analyses

Data from other tests will be listed, notable values will be flagged, and any other information will be listed as appropriate.

All assessments collected later than 30 days after the last treatment date will be flagged in the listings.

Subgroup analyses will be explored as described in Section 2.2.3.

2.9 Pharmacokinetic analysis

PK assessments:

Patients in the control arm will yield PK data from blood sampling performed at pre-dose with respect to the octreotide long-acting i.m. injection at week 4, 12, 16 and 24 for the determination of plasma trough levels. These trough levels will be presented descriptively.

Descriptive statistics regarding trough levels of octreotide (n, arithmetic mean, CV% mean, standard deviation (SD), median, minimum and maximum) will be presented for Pharmacokinetic analysis set. Since patients in the control arm can start on a lower dose than that specified for the control arm (60 mg), descriptive statistics will be presented for those who start below 60 mg, those that start at 60 mg, and overall.

In addition, the arithmetic/geometric mean (±SD) and median concentration-time profiles over time will be displayed graphically on the linear and semi-log view.

All individual through levels will be listed using the Full analysis set.

The above PK summary will be generated separately, but not be included in the primary CSR.

PK parameters

Not applicable. No PK parameters will be derived.

Handling of PK data below LLOQ or missing

All concentration values below the lower limit of quantitation (LLOQ) are set to zero by the Bioanalyst, and will be displayed in the listings as zero and flagged. LLOQ values will be treated as zero in any calculations of summary statistics. The number of non-zero concentrations will also be reported in the summary statistics.

Missing values for any PK data will not be imputed and will be treated as missing.

2.10 Dosimetry analysis

For patients randomized in Germany to the Lutathera investigational arm that receive Lutathera (including re-treatment administrations), or patients randomized in Germany to the control arm that receive Lutathera during cross-over, some high-level dosimetry results will be listed as captured in the eCRF per timepoint.

Below are the dosimetry data to be listed:

- Whole body planar imaging
  - Was whole body planar imaging done? If not done, reason not done
  - Date of Scan and start time of scan
- SPECT/CT imaging
  - Was SPECT/CT imaging done? If not done, reason not done
  - Anatomical location of imaging coverage (select all that apply)
  - Date and start time of imaging
- Dosimetry calculation
  - Was dosimetry calculation performed? If not done, reason not done
  - Absorbed radiation dose in kidneys in Grays
  - Absorbed radiation dose in bone marrow in Grays

Any further analyses of dosimetry will be captured in a separate report.

2.11 Patient-reported outcomes

These endpoints are covered in Section 2.6.1 and 2.7.1.

2.12 Duration of follow-up

Study follow-up will be summarized, including duration between randomization and cut-off date, and follow-up times for PFS (1st central progression) and OS separately, which are defined as follows:

- Duration between randomization and data cut-off date = (Cut-off date – Date of randomization + 1) / 30.4375 (months). This item will be summarized overall.
- Follow-up time for PFS = (Date of event or censoring – Date of randomization + 1) / 30.4375 (months) regardless of censoring. Date of censoring is defined as the last adequate tumor assessment date for PFS. This item will be summarized by treatment arm.
- Follow-up time for OS = (Date of event or censoring – Date of randomization + 1) / 30.4375 (months) regardless of censoring. Date of censoring is defined as the last contact date for OS. This item will be summarized by treatment arm.

All summaries will be reported in months. The calculations for PFS will be based on central assessment.

In addition, the time from PFS/OS censoring to the data cut-off date will be separately summarized by time intervals in months: <3, 3 to <6, 6 to <12, 12 to <18, 18 to <24 and by 12 month intervals thereafter if necessary. The gap time is calculated as ([analysis cut-off date] – [censoring date] +1)/30.4375.

2.13 Interim analysis

There will be no interim analysis with inferential intent. But, at the time of the PFS primary analysis, an estimate of overall survival will be calculated in terms of hazard ratio (point estimate) and 95% confidence interval. Furthermore, a presentation of safety will be prepared including data from the time period up the interim.

3 Sample size calculation

The sample size calculation is based on the primary variable PFS. Assuming a median PFS in the control arm (High Dose octreotide long-acting (60 mg)) of approximately 15 months, it is hypothesized that treatment with Lutathera added to standard dose octreotide long-acting will result in a 50% reduction in the hazard rate (corresponding to an increase in median PFS from 15 months to 30 months).

To ensure 90% power to test the null hypothesis: PFS hazard ratio = 1, versus the specific alternative hypothesis: PFS hazard ratio = 0.50, it is calculated that a total of 99 PFS events need to be observed. This calculation assumes analysis by a one-sided log-rank test at the overall 2.5% level of significance, patients randomized to the two treatment groups in a 2:1 ratio. Assuming that enrolment will continue for approximately 22.2 months at a rate of 10 patients per month and a 15% dropout rate by the time of the final PFS analysis, a total of 222 patients (148 for Lutathera arm and 74 for the control arm) will need to be randomized to observe the targeted 99 PFS events at about 12.8 months after the randomization date of the last patient, i.e., 35 months after the randomization date of the first patient. If the final analysis is performed when the targeted 99 PFS events are observed, the observed hazard ratio will have to be < 0.658 which corresponds to a difference in median PFS of 7.8 months to declare statistical significance.

These calculations were made using the software package East 6.4.

3.1 Power for analysis of key secondary variables

No power calculation has been performed for the key secondary variables.

4 Change to protocol specified analyses

Not applicable

5 Appendix

5.1 Imputation rules

5.1.1 Study treatment

The following rule should be used for the imputation of the treatment end date for a given study treatment component:

Scenario 1: If the treatment end date is completely missing and there is no EOT page and no death date, the patient is considered as on-going:

- The patient should be treated as on-going and the cut-off date should be used as the treatment end date.

Scenario 2: If the treatment end date is completely or partially missing and the EOT page is available:

- Case 1: The treatment end date is completely missing, and the EOT completion date is complete, then this latter date should be used.
- Case 2: Only Year(yyyy) of the treatment end date is available and yyyy < the year of EOT date: **Use Dec31yyyy**
- Case 3: Only Year(yyyy) of the treatment end date is available and yyyy = the year of EOT date: **Use EOT date**
- Case 4: Both Year(yyyy) and Month (mm) are available for treatment end date, and yyyy = the year of EOT date and mm < the month of EOT date: **Use last day of the Month (mm)**

All other cases should be considered as a data issue and data should be queried.

After imputation, compare the imputed date with start date of treatment, if the imputed date is < start date of treatment: **Use the treatment start date**

Patients with missing start dates are to be considered missing for all study treatment component related calculations and no imputation will be made. If the start date is missing, then end date should not be imputed.

5.1.2 AE, ConMeds and safety assessment date imputation

Table 5.1-1 Imputation of start dates (AE, CM) and assessments (LB, EG, VS)

Missing Element	Rule
day, month, and year	No imputation will be implemented.
day, month	• If available year < year of randomized treatment start date then 01JulYYYY<br>• If available year = year of randomized treatment start date then<br>o If stop date contains a full date and end date is earlier than randomized treatment start date then set start date = 01JanYYYY<br>o Else set start date = randomized study treatment start date.<br>• If available year > year of randomized treatment start date then<br>o For those that do not receive cross-over/re-treatment then 01JanYYYY<br>o For those that did receive cross-over/re-treatment therapy (during cross-over/re-treatment therapy)<br>▪ If available year < year of cross-over/re-treatment start date then 01JanYYYY<br>▪ If available year = year of cross-over/re-treatment start date, then<br>- If end date contains a full date and end date is earlier than cross-over/re-treatment start date then set start date = 01JanYYYY<br>- Else start date = cross-over/re-treatment start date.<br>▪ If available year > year of cross-over/re-treatment start date then 01JanYYYY

Missing Element	Rule
day	• If available month and year < month year of randomized treatment start date then 15MONYYYY<br>If available month and year = month and year of randomized treatment start date then<br>o If stop date contains a full date and end date is earlier than randomized treatment start date then set start date= 01MONYYYY.<br>o Else set start date = randomized treatment start date.<br>• If available month and year > month and year of randomized treatment start date<br>o For those that do not receive cross-over/re-treatment therapy then 01MONYYYY<br>o For those that did receive cross-over/re-treatment therapy (during cross-over/re-treatment therapy)<br>▪ If available month and year < month year of cross-over/re-treatment start date then 01MONYYYY<br>▪ If available month and year = month and year of cross-over/re-treatment start date then<br>- If end date contains a full date and end date is earlier than cross-over/re-treatment start date then set start date = 01MONYYYY<br>- Else start date = cross-over/re-treatment start date<br>▪ If available month and year > month and year of cross-over/re-treatment start date then 01MONYYYY

Table 5.1-2 Imputation of end dates (AE, CM)

Missing Element	Rule
day, month, and year	• No imputation will be implemented.
day, month	• If partial end date contains year only, set end date = earliest of 31DecYYYY, end date of the randomized/crossover/re-treatment/follow up period (whichever is most applicable on study), death date, cut-off date, withdrawal of consent date, end of study date.*

Missing Element	Rule
day	• If partial end date contains month and year, set end date = earliest of last day of the month, end date of the randomized/crossover/re-treatment/follow up period (whichever is most applicable on study), death date, cut-off date, withdrawal of consent date, end of study date.*
* If the imputed AE end date is earlier than the existing full AE start date, then use the AE start date as AE end date.	

Any AEs and ConMeds with partial/missing dates will be displayed as such in the data listings.

Any AEs and ConMeds which are continuing as per data cut-off will be shown as 'ongoing' rather than the end date provided.

The above imputations are used for analyses of time to and duration of AEs, determination of TEAEs, and concomitant medications.

5.1.2.1 Other imputations

Incomplete date of initial diagnosis of cancer and date of most recent recurrence

Missing day is defaulted to the 15th of the month and missing month and day is defaulted to 01-Jan.

Incomplete assessment dates for tumor assessment

All investigation dates (e.g., MRI scan, CT scan) must be completed with day, month and year. If one or more assessment dates are incomplete but other investigation dates are available, this/these incomplete date(s) are not considered for calculation of the assessment date and assessment date is calculated as the latest of all investigation dates (e.g., MRI scan, CT scan) if the overall response at that assessment is CR/PR/SD/UNK. Otherwise – if overall response is progression – the assessment date is calculated as the earliest date of all investigation dates at that evaluation number. If all measurement dates have no day recorded, the 1st of the month is used. If the month is not completed, for any of the investigations, the respective assessment will be considered to be at the date which is exactly between previous and following assessment. If a previous and following assessment is not available, this assessment will not be used for any calculation.

Applying the cut-off to tumor assessment

For tumor related assessments, if an evaluation has some assessments done prior to cut-off date and others after the cut-off date , then the evaluation is considered post-cut-off date and will be excluded from analysis.

Handling Missing month/day in date of death

For rare cases when either day is missing or both month and day are missing for the date of death the following imputation rules will be implemented:

- If only day is missing, then impute max [(1 mmm-yyyy), min(any valid date from data base used for deriving last contact date+1, cutoff date)]
- If both day and month are missing, then impute max [(1 Jan-yyyy, min(any valid date from data base used from deriving last contact date +1, cutoff date)].

5.2 AEs coding/grading

Adverse events are coded using the Medical dictionary for regulatory activities (MedDRA) terminology.

AEs will be assessed according to the Common Terminology Criteria for Adverse Events (CTCAE) version 5.0 or current version, or, if the term does not exist in CTCAE version 5.0, according to the "severity grade".

The CTCAE represents a comprehensive grading system for reporting the acute and late effects of cancer treatments. CTCAE grading is by definition a 5-point scale generally corresponding to mild, moderate, severe, life threatening, and death. This grading system inherently places a value on the importance of an event, although there is not necessarily proportionality among grades (a grade 2 is not necessarily twice as bad as a grade 1).

"Severity grade" is a 3-point scale corresponding to mild, moderate and severe categories.

5.3 Laboratory parameters derivations

Grade categorization of lab values will be assigned programmatically as per NCI Common Terminology Criteria for Adverse Events (CTCAE) version 5.0 or current. The calculation of CTCAE grades will be based on the observed laboratory values only, clinical assessments will not be taken into account. The criteria to assign CTCAE grades are given in Novartis internal criteria for CTCAE grading of laboratory parameters shown below.

For laboratory tests where grades are not defined by CTCAE v5.0, results will be graded by the low/normal/high based on laboratory normal ranges.

A severity grade of 0 will be assigned for all non-missing lab values not graded as 1 or higher. Grade 5 will not be used. For laboratory tests that are graded for both low and high values, summaries will be done separately and labelled by direction, e.g., sodium will be summarized as hyponatremia and hypernatremia.

CTC grades for laboratory values in Novartis Oncology (based on CTCAE v5 – Nov 2017)

Page 1

				CTC Grades(1)				
**Lab test (toxicity)**	**SI unit**	**Lab test (NCDS)**	**Normal ranges (Merck manual, July 2015) and *conversion factors***	**0**	**1**	**2**	**3**	**4**
**Hematology**								
WBC ↓<br>WBC (Leukocytosis)	10^9/L<br>10^9/L	WBC<br>WBC	**3.9 – 10.7 x 10^9/L**	≥ LLN	< LLN - 3.0 x 10^9/L<br>-	< 3.0 – 2.0 x 10^9/L<br>-	< 2.0 – 1.0 x 10^9/L<br>> 100 x 10^9/L	< 1.0 x 10^9/L<br>-
Hemoglobin (Anemia)<br>Hemoglobin ↑	g/L<br>g/L	HGB<br>HGB	**120 - 160 g/L** or 7.4 - 9.9 mmol/L (F)<br>**140 - 170 g/L** or 8.7 – 10.6 mmol/L (M)<br>*(16.113 x mmol/L = g/L)*	≥ LLN	< LLN - 100 g/L<br>< LLN - 6.2 mmol/L<br>Increase >0-20 g/L above ULN	< 100 - 80 g/L<br>< 6.2 - 4.9 mmol/L<br>Increase >20-40 g/L above ULN	< 80 g/L<br>< 4.9 mmol/L<br>Increase >40 g/L above ULN	-<br>-
Platelets ↓	10^9/L	PLAT	**150 - 350 x 10^9/L**	≥ LLN	< LLN - 75.0 x 10^9/L	< 75.0 - 50.0 x 10^9 L	< 50.0 - 25.0 x 10^9/L	< 25.0 x 10^9/L
Neutrophils ↓	10^9/L	NEUT		≥2x10^9/L	< 2.0 - 1.5 x 10^9/L	< 1.5 - 1.0 x 10^9/L	< 1.0 - 0.5 x 10^9/L	< 0.5 x 10^9/L
Lymphocytes↓<br>Lymphocytes ↑	10^9/L<br>10^9/L	LYM<br>LYM		≥1.5x10^9/L	< 1.5 - 0.8 x 10^9/L<br>-	< 0.8 - 0.5 x 10^9/L<br>> 4 - 20 x 10^9/L	< 0.5 - 0.2 x 10^9/L<br>> 20 x 10^9/L	< 0.2 x 10^9/L<br>-
**Biochemistry**								
AST ↑	U/L	AST	**0 - 35 U/L** or 0 – 0.58 ukat/L<br>*(60 x ukat/L = U/L)*	≤ ULN	> ULN – 3.0 x ULN	> 3.0 - 5.0 x ULN	> 5.0 - 20.0 x ULN	> 20.0 x ULN
ALT ↑	U/L	ALT	**0 - 35 U/L** or 0 – 0.58 ukat/L<br>*(60 x ukat/L = U/L)*	≤ ULN	> ULN – 3.0 x ULN	> 3.0 - 5.0 x ULN	> 5.0 - 20.0 x ULN	> 20.0 x ULN
Total bilirubin ↑	umol/L	BILI	**5.1 – 20.5 umol/L** or 0.3 – 1.2 mg/dL<br>*(17.1 x mg/dL = umol/L)*	≤ ULN	> ULN - 1.5 x ULN	> 1.5 - 3.0 x ULN	> 3.0 - 10.0 x ULN	> 10.0 x ULN
Alk. Phosphatase ↑	U/L	ALP	**36 - 92 U/L** or 0.5 - 1.5 ukat/L<br>*(60 x ukat/L = U/L)*	≤ ULN	> ULN - 2.5 x ULN	> 2.5 - 5.0 x ULN	> 5.0 - 20.0 x ULN	> 20.0 x ULN
Creatinine ↑	umol/L	CREAT	**61.9 - 115 umol/L** or 0.7 – 1.3 mg/dL<br>*(88.4 x mg/dL = umol/L)*	≤ ULN	> ULN - 1.5 x ULN	> 1.5 - 3.0 x ULN	> 3.0 - 6.0 x ULN	> 6.0 x ULN
Creatinine kinase↑	U/L	CK	**30 - 170 U/L** or 0.5 – 2.83 ukat/L<br>*(60 x ukat/L = U/L)*	≤ ULN	> ULN - 2.5 x ULN	> 2.5 - 5.0 x ULN	> 5.0 - 10.0 x ULN	> 10.0 x ULN
Albumin (Hypoalbuminemia)	g/L	ALB	**35 - 55 g/L** or 3.5 to 5.5 g/dL	≥ LLN	< LLN - 30 g/L	< 30 - 20 g/L	< 20 g/L	-
Total Cholesterol ↑	mmol/L	CHOL	**3.88 – 5.15 mmol/L** or 150 - 199 mg/dL<br>*(38.67 x mg/dL = mmol/L)*	≤ ULN	> ULN - 7.75 mmol/L<br>> ULN - 300 mg/dL	> 7.75 -10.34 mmol/L<br>> 300 – 400 mg/dL	>10.34-12.92 mmol/L<br>> 400 – 500 mg/dL	>12.92 mmol/L<br>> 500 mg/dL
Lipase ↑	U/L	LIPASE	**<95 U/L** or <1.58 ukat/L<br>*(60 x ukat/L = U/L)*	≤ ULN	> ULN - 1.5 x ULN	> 1.5 - 2.0 x ULN	> 2.0 - 5.0 x ULN	> 5.0 x ULN
Amylase ↑	U/L	AMYLASE	**0 - 130 U/L** or 0 – 2.17 ukat/L<br>*(60 x ukat/L = U/L)*	≤ ULN	> ULN - 1.5 x ULN	> 1.5 - 2.0 x ULN	> 2.0 - 5.0 x ULN	> 5.0 x ULN
Uric acid (Hyperuricemia)	umol/L	URATE	**150 - 470 umol/L** or 2.5 – 8 mg/dL<br>*(59.48 x mg/dL = umol/L)*	Defined by clinical criteria only in CTCAE V5				

ULN = Upper Limit of Normal range; LLN = Lower Limit of Normal range

LAB - CTC grades in Novartis Oncology (26jul18)

CTC grades for laboratory values in Novartis Oncology (based on CTCAE v5 – Nov 2017)

Page 2

				CTC Grades(1)				
**Lab test (toxicity)**	**SI unit**	**Lab test (NCDS)**	**Normal ranges (Merck manual, July 2015) and *conversion factors***	**0**	**1**	**2**	**3**	**4**
Phosphorus (Hypophosphatemia)	mmol/L	PHOS	**0.97 – 1.45 mmol/L** or 3.0 - 4.5 mg/dL<br>*(0.32 x mg/dL = mmol/L)*	Defined by clinical criteria only in CTCAE V5				
Calcium (corrected) (Hypercalcemia)<br>Calcium (corrected) (Hypocalcemia)	mmol/L<br>mmol/L	CACALC<br>CACALC	**2.2 - 2.6 mmol/L** or 9 - 10.5 mg/dL<br>*(0.2495 x mg/dL = mmol/L)*	≤ ULN<br>≥ LLN	> ULN - 11.5 mg/dL<br>> ULN - 2.9 mmol/L<br>< LLN - 8.0 mg/dL<br>< LLN - 2.0 mmol/L	> 11.5 - 12.5 mg/dL<br>> 2.9 - 3.1 mmol/L<br>< 8.0 - 7.0 mg/dL<br>< 2.0 - 1.75 mmol/L	> 12.5 - 13.5 mg/dL<br>> 3.1 - 3.4 mmol/L<br>< 7.0 - 6.0 mg/dL<br>< 1.75 - 1.5 mmol/L	> 13.5 mg/dL<br>> 3.4 mmol/L<br>< 6.0 mg/dL<br>< 1.5 mmol/L
Magnesium (Hypermagnesemia)<br>Magnesium (Hypomagnesemia)	mmol/L<br>mmol/L	MG<br>MG	**0.62 – 0.99 mmol/L** or 1.5 – 2.4 mg/dL<br>*(0.4114 x mg/dL = mmol/L)*	≤ ULN<br>≥ LLN	> ULN - 3.0 mg/dL<br>> ULN - 1.23 mmol/L<br>< LLN - 1.2 mg/dL<br>< LLN - 0.5 mmol/L	-<br>< 1.2 - 0.9 mg/dL<br>< 0.5 - 0.4 mmol/L	> 3.0 – 8.0 mg/dL<br>> 1.23 – 3.3 mmol/L<br>< 0.9 - 0.7 mg/dL<br>< 0.4 - 0.3 mmol/L	> 8.0 mg/dL<br>> 3.3 mmol/L<br>< 0.7 mg/dL<br>< 0.3 mmol/L
Glucose (non-fasting) (Hyperglycemia)<br>Glucose (fasting) (Hyperglycemia)	mmol/L<br>mmol/L	GLUCSN<br>GLUCSF	**<7.8 mmol/L** or <140 mg/dL<br>*(0.05551 x mg/dL = mmol/L)*<br>**3.9 – 5.8 mmol/L** or 70 - 105 mg/dL<br>*(0.05551 x mg/dL = mmol/L)*	Defined by clinical criteria only in CTCAE V5				
Glucose (Hypoglycemia)	mmol/L	GLUCSN/ GLUCSF		≥ LLN	< LLN - 55 mg/dL<br>< LLN - 3.0 mmol/L	< 55 - 40 mg/dL<br>< 3.0 - 2.2 mmol/L	< 40 - 30 mg/dL<br>< 2.2 - 1.7 mmol/L	< 30 mg/dL<br>< 1.7 mmol/L
Potassium (Hyperkalemia)<br>Potassium (Hypokalemia)	mmol/L<br>mmol/L	K<br>K	**3.5 - 5.0 mmol/L**<br>*(0.2558 x mg/dL = mEq/L = mmol/L)*	≤ ULN<br>≥ LLN	> ULN - 5.5 mmol/L<br>< LLN - 3.0 mmol/L	> 5.5 - 6.0 mmol/L<br>-	> 6.0 - 7.0 mmol/L<br>< 3.0 - 2.5 mmol/L	> 7.0 mmol/L<br>< 2.5 mmol/L
Sodium (Hypernatremia)<br>Sodium (Hyponatremia)	mmol/L<br>mmol/L	SODIUM<br>SODIUM	**136 - 145 mmol/L**<br>*(0.435 x mg/dL = mEq/L = mmol/L)*	≤ ULN<br>≥ LLN	> ULN - 150 mmol/L<br>< LLN - 130 mmol/L	> 150 - 155 mmol/L<br>< 129 - 125 mmol/L	> 155 - 160 mmol/L<br>< 124 - 120 mmol/L	> 160 mmol/L<br>< 120 mmol/L
Triglyceride ↑	mmol/L	TRIG	**< 2.82 mmol/L** or < 250 mg/dL<br>*(0.01129 x mg/dL = umol/L)*	< 150<br>< 1.71	≥ 150 - 300 mg/dL<br>≥ 1.71 – 3.42 mmol/L	> 300 - 500 mg/dL<br>> 3.42 – 5.7 mmol/L	> 500 - 1000 mg/dL<br>> 5.7 – 11.4 mmol/L	> 1000 mg/dL<br>> 11.4 mmol/L
**Coagulation**								
INR↑	1	INR	**0.8 – 1.2**	≤ 1.2	> 1.2- 1.5	> 1.5- 2.5	> 2.5	-
Activated partial thromboplastin time↑	sec	APTT	**25 - 35 sec**	≤ ULN	> ULN - 1.5 x ULN	> 1.5 - 2.5 x ULN	> 2.5 x ULN	-
Fibrinogen ↓	g/L	FIBRINO	**1.5 – 3.5 g/L** or 150 – 350 mg/dL<br>*(0.01 x mg/dL = g/L)*	≥ LLN	< LLN - 0.75 x LLN	< 0.75 - 0.5 x LLN	< 0.5 - 0.25 x LLN	< 0.25 x LLN

ULN = Upper Limit of Normal range; LLN = Lower Limit of Normal range

(1) **LAB CTC grades 1, 2, 3, 4 overrule the study specific (central or local) normal range criteria**, e.g. if ULN of Sodium is 151 mmol/L and the value is 151 mmol/L, CTC grade 2 is assigned although the value is ≥ ULN.
Clinical criteria such as 'asymptomatic' or 'Life-threatening consequences' are not considered for determination of LAB CTC grades. Concomitant usage of therapy is also not considered.
Values and LNRs for blood differentials can be given as %, absolute values should then be calculated using WBC. Generally, ≥ 1.5 x 109 /L (lymphocytes) and ≥ 2 x 109 /L (neutrophils) are considered as LAB CTC grade 0
The **comparison with baseline is not considered** for derivation of LAB CTC grades

Imputation Rules

CTC grading for blood differentials is based on absolute values. However, this data may not be reported as absolute counts but rather as percentage of WBC.

If laboratory values are provided as ‘<X’ (i.e., below limit of detection) or ‘>X’, prior to conversion of laboratory values to SI unit, these numeric values are set to X.

The following rules will be applied to derive the WBC differential counts when only percentages are available for a xxx differential

xxx count = (WBC count) * (xxx %value / 100)

Further derivation of laboratory parameters might be required for CTCAE grading. For instance, corrected calcium can be derived using the reported total calcium value and albumin at the same assessment using the following formula:

Corrected Calcium (mg/dL) = Calcium (mg/dL) – 0.8 [Albumin (g/dL)-4]

In order to apply the above formula, albumin values in g/L will be converted to g/dL by multiplying by 0.1, calcium values in mmol/L will be converted to mg/dL by dividing by 0.2495. For calculation of laboratory CTC grades 0 and 1, the normal range for derived corrected calcium is set to the same limits (in mg/dL) as for calcium.

CTC grades for the derived absolute WBC differential counts (neutrophils, lymphocytes) and corrected calcium will be assigned as described above for grading.

For creatine clearance (CrCl), the calculated CrCl from sites will be used. If calculated value is not available, then the below Cockroft-Gault formula will be used to do the imputation:

- Male GFR (mL/min) = [140 – age (years)] x weight (kg) x 1.23 / serum creatinine (µmol/L)
- Female GFR (mL/min) = [140 – age (years)] x weight (kg) x 1.04 / serum creatinine (µmol/L)
  OR
- Male GFR (mL/min) = [140 – age (years)] x weight (kg) / [72 x serum creatinine (mg/dL)]
- Female GFR (mL/min) = [140 – age (years)] x weight (kg) x 0.85 / [72 x serum creatinine (mg/dL)]

Age/weight at the visit of CrCl assessment will be used. If weight is not available at that specific visit, the latest weight collected before the CrCl assessment will be used in the calculation.

5.4 Statistical models

5.4.1 Primary analysis

<u>**Analysis of time to events Data**</u>

Hypothesis and test statistic

The below description is for PFS

The null hypothesis stating that PFS survival distributions of the two treatment groups are equivalent will be tested against one-sided alternative.

Assuming proportional hazards for PFS, the following statistical hypotheses will be tested:

$$H_{01}: \theta_1 \geq 1 \text{ vs. } H_{A1}: \theta_1 < 1$$

where θ_1 is the PFS hazard ratio (Lutathera arm vs Octreotide arm).

Stratified log-rank test adjusting for the strata used in the randomization will be implemented as follows: In each of the K strata separately, the LIFETEST procedure with STRATA statement including only the treatment group variable and with the TIME statement will be used to obtain the rank statistic S_k and variance var(S_k) where k=1, 2, …, K. The final test statistics will then be reconstructed as follows:

$$Z=[S_1+\ldots+S_K]/\sqrt{[var(S_1)+\ldots+var(S_k)]}.$$

As a sensitivity analysis, unstratified Log-rank test will be used to test the difference between the treatment groups. The LIFETEST procedure in SAS with the TIME statement including a variable with survival times and a (right) censoring variable, and with STRATA statement including a variable for identifying treatment groups will be used. As an output of the procedure, the rank statistic S and variance var(S) will be obtained. Under the null hypothesis, the test statistic Z=S/√[var(S)] is approximately normally distributed.

Kaplan-Meier estimates

An estimate of the survival function in each treatment group will be constructed using Kaplan-Meier (product-limit) method as implemented in PROC LIFETEST with METHOD=KM option. The PROC LIFETEST statement will use the option CONFTYPE=LOGLOG.

Median survival for each treatment group will be obtained along with 95% confidence intervals calculated from PROC LIFETEST output using the method of [Brookmeyer and Crowley 1982]. Kaplan-Meier estimates of the survival function with 95% confidence intervals at specific time points will be summarized. The standard error of the Kaplan-Meier estimate will be calculated using Greenwood's formula [Collett 1994].

Hazard ratio

Hazard ratio will be estimated by fitting the Cox proportional hazards model using SAS procedure PHREG (with TIES=EXACT option in the MODEL statement).

- For estimation without stratification the following applies. An unadjusted Cox model will be used, i.e., the MODEL statement will include the treatment group variable as the only covariate.
- For estimation with stratification the following applies. A stratified unadjusted Cox model will be, i.e., the MODEL statement will include the treatment group variable as the only covariate and the STRATA statement will include stratification variable(s).

Hazard ratio with two-sided 95% confidence interval will be based on Wald test.

Treatment of ties

The STRATA statement in LIFETEST procedure will be used to analyze time to event data with ties. The PHREG procedure in SAS with option TIES=EXACT will be used to fit the Cox proportional hazards model.

Checking proportionality of hazard assumption

Plots (SURVIVAL LOGSURV LOGLOGS) generated by LIFETEST procedure in SAS will be used to provide visual checks of the proportional hazard assumption.

- SURVIVAL plots estimated survivor functions. The shape of the curves should be basically the same if hazards are proportional.
- LOGSURV plots the cumulative hazard functions The larger cumulative hazard should be a multiple of smaller if hazards are proportional
- LOGLOGS plots log (cumulative hazard). The LOGLOG plot will show parallel curves if hazards are proportional.

As an exploratory measure, to test the proportional hazard assumption, the treatment group * time interaction will be added in the MODEL statement in PHREG procedure in SAS. Evidence that interaction is not zero is evidence against proportional hazards. Further, the correlation between time and Schoenfeld residuals will be calculated to provide further evidence concerning the degree of proportionality.

Multiplicity adjustment

No adjustment for multiplicity will be applied. To protect the type I error for the primary and key secondary endpoints, these will be tested in a hierarchical fashion in the following order: PFS (centrally assessed), ORR, QoL Global Health (TTD), QoL Diarrhea (TTD), fatigue (TTD), and pain (TTD).

5.4.2 Key secondary analysis

Analysis of Binary Data (ORR)

The null hypothesis of equality of response rate in the two treatment arm will be tested against one-sided alternative. The statistical hypotheses are:

$$H0: ORRR \leq ORRC \text{ versus } HA: ORRR > ORRC, \text{ for a one-sided test}$$

where ORRR is the probability of response in investigational arm and ORRC is the probability of response in control arm.

Both a stratified and an unstratified analysis will be presented.

The stratified analysis proceeds as outlined below.

The Cochran-Mantel-Haenszel chi-square test X^2_{CMH} (implemented again via SAS procedure FREQ with CMH option in the TABLES statement) will be used to test the difference in response rates between the treatment arms. The p-value corresponding to the CMH test for "general association" will be used which follows a Chi-square distribution with one degree of freedom.

If the sampling assumptions for chi-square test is not met, the exact Cochran-Mantel-Haenszel test will be used (implemented via SAS procedure MULTTEST). The test is performed by running a stratified version of the Cochran-Armitage permutation test [Armitage et al. 1969]. In studies with stratified randomization, the chi-square approximation is considered appropriate for the X^2_{CMH} statistics if the rule of Mantel and Fleiss [Mantel and Fleiss 1980] is satisfied.

The unstratified analysis proceeds outlined as below.

The Mantel-Haenszel chi-square test X^2_{MH} (implemented via SAS procedure FREQ with CMH option in the TABLES statement) will be used to test the difference in response rates between the treatment arms. The p-value corresponding to the CMH test for "general association" will be used which follows a Chi-square distribution with one degree of freedom.

If the sampling assumptions for chi-square test is not met, Fisher's exact test (implemented via SAS procedure FREQ with EXACT option in the TABLES statement) will be used to test the difference in response rates between the treatment arms. the rule for determining adequate sample size for $X^2$is that expected values should exceed 5 for all of the table cells.

Confidence interval for response rate

Responses will be summarized in terms of percentage rates with $100(1 - \alpha)\%$ confidence interval using exact binomial confidence interval (implemented using SAS procedure FREQ with EXACT statement for one-way table (Clopper and Pearson 1934).

5.4.3 Special statistical methods for OS analysis

Patients who progressed on Octreotide LAR are allowed to cross-over to open-label Lutathera. However, Octreotide LAR patients who switch to Lutathera after they progress are likely to benefit from the delayed administration of Lutathera and, therefore, the survival treatment estimate becomes confounded. Under such circumstances, the statistical test of the treatment effect is known to be biased towards the null hypothesis of no difference (Robins and Tsiatis 1991; Korhonen et al 1999; Greenland et al 2008).

However, with the inclusion of Lutathera re-treatment for some patients randomized in the Lutathera investigational arm, these patients may have increased OS compared to those who do not receive Lutathera re-treatment after central progression.

Therefore, the following modeling approaches will be used to obtain an estimate of survival treatment effect corrected for treatment cross-over and re-treatment: 'Rank-preserving

structural failure time method' and 'Cox model with Inverse Probability of Censoring Weighting'. More details are provided in sections below. Should a patient have switched prior to progression (locally or centrally confirmed), that patient will be analysed similarly to those that switched after progression.

Despite the cross-over and re-treatment design, a supportive analysis of OS will use the strict intention-to-treat (ITT) approach, i.e., 'analyze as randomized' and ignore the treatment switch or re-treatment with Lutathera (and ignore additional anticancer therapies administered after study treatment discontinuation).

5.4.3.1 RPSFT approach

The primary analysis of OS will be using ***rank-preserving structural failure time method (RPSFT)*** to correct for confounding introduced by the change of treatment (Robins and Tsiatis 1991; Korhonen et al 1999). The use of RPSFT method allows to estimate the survival time gained by anyone receiving Lutathera (i.e., either as randomized to Lutathera or after cross-over from Octreotide LAR to open-label Lutathera). The RPSFT model is based on an accelerated failure time model (Kalbfleisch and Prentice 2002) and uses a structural assumption of time-proportionality instead of a proportional hazards assumption as used in the Cox model. The widely used Cox model measures drug effect on the hazard ratio scale, whereas the accelerated failure time model measures drug effect on the survival time ratio scale.

Notation

We follow (Korhonen et al 1999) for the notation. The treatment assignment indicator is denoted by R_i and takes the value 0 if allocated to Octreotide LAR and 1 if allocated to active drug (Lutathera 10 mg). $Strata_i$ stands to baseline stratification factors. Each individual is followed from the time of randomization (DR_i) until death or the date of last contact (EOF_i) and the fixed closing date (CD_i) which ever comes first. The censoring time $C_i = min(EOF_i - DR_i, CD_i - DR_i)$, is measured in days and is assumed to be independent of the treatment assignment. (Note: in a sensitivity analysis, censoring time $C_i = EOF_i - DR_i$ will also be considered). Time to death (T_i) is the time from randomization to death which is also measured in days. We can only observe $X_i = \min(T_i, C_i)$ with $\delta_i = 1$ if $X_i = T_i$ and $\delta_i = 0$ otherwise. Some of the subjects randomized to the Octreotide LAR arm switch to the active drug at some point after randomization. There may be several different periods when active drug has been received instead of the randomized Octreotide LAR. The total time from randomization to switch is denoted as A_i. If no switch occurs in the Octreotide LAR arm then $A_i = 0$. Switching to the active drug is potentially related to the patient's prognosis and thus cannot be assumed independent of the death time.

Thus, the observed data for each subject i are $\{R_i, Strata_i, C_i, X_i, \delta_i, A_i\}$.

Model

For subject i, we denote U_i as the potential survival time without Lutathera. U_i is a concept that is defined for each subject at the time of the randomization and U_i may depend on the value of

the baseline risk factor. U_i will be observed only for those subjects who will never receive active drug during the study and die before $min(EOF_i, CD_i)$.

For a given time to switch A_i , the Lutathera-free survival time U_i is linked to the death time T_i through a structural model

$$U_i = \int_0^{T_i} e^{\psi A_i(s)} ds$$

where $A_i(t) = 1$ if subject i has not switched at time t and 0 otherwise. For a fixed value of the parameter ψ the above model can be written as

$$U_i(\psi) = \int_0^{A_i} e^{\psi \times 1} ds + \int_0^{T_i - A_i} e^{\psi \times 0} ds = A_i e^{\psi} + (T_i - A_i) \cdot$$

If $A_i(t) = 0$ for all t then $U_i = T_i$. If, on the other hand $A_i(t) = 1$ for all t then $U_i = T_i e^{\Psi}$. Thus the parameter ψ has the following interpretation: $e^{-\Psi}$ expresses how much the survival time is increased or decreased on relative scale if on Lutathera versus if not on Lutathera.

Therefore, we can see that the main assumption of this method is that the total duration of survival time for each patient is the sum of two distinct parts:

- potential survival time, had no Lutathera been received, and
- time added from multiplying the duration on Lutathera by an unknown factor.

For the estimation of the parameter ψ it needs to be assumed that:

1. the above model correctly captures the drug action
2. no other factor than the amount of Lutathera received, A_i, induces a difference in survival experience for an individual i under different treatment arm assignments
3. the Lutathera-free survival time U_i for an individual i is unaffected by the drug assignment or by the survival experience of other individuals
4. the censoring time C_i is independent of the randomization mechanism R_i

Estimation algorithm for parameter ψ

Randomization guarantees that any variable measured at baseline will on average be balanced with respect to the drug assignment. In particular, $U_i \amalg R_i$ where $\amalg$ denotes statistical independence. This holds also within each of the k strata of the baseline risk factor. It follows that \

$$\Pr(U_i(\psi) \leq x \mid R_i = 0, Strata_i = z) = \Pr(U_i(\psi) \leq x \mid R_i = 1, Strata_i = z), \quad z = 1,2,..,k.$$

In other words, the drug free survival time is independent of the randomization within the different strata of the baseline risk factor. Therefore, a procedure for estimating the parameter ψ can be based on computing $U_i(\psi)$ for various values of ψ and finding a value $\hat{\psi}$ where $U_i(\hat{\psi}) \amalg R_i \mid Strata_i$. We use the stratified log-rank test statistic for a device to estimate the

parameter ψ. The stratified log-rank test statistic is obtained from PROC PHREG procedure in SAS. The stratified log-rank test has an asymptotic chi-squared distribution with one degree of freedom under the hypothesis $\psi = \psi_0$ where ψ_0 denotes the true parameter value. From a grid of values for ψ we choose as $\hat{\psi}$ where the stratified log-rank test statistic has its minimum value. The approximate lower and upper 95% confidence limits are obtained as those values where the stratified log-rank test statistic is close to 3.84. In reality the stratified log-rank test statistic is a step function in ψ and therefore there might be several values where the minimum is obtained and also the upper and lower confidence limits need to be chosen as the point for ψ where the value of the stratified log-rank test statistic changes from less than 3.84 to greater than 3.84.

Complication due to censoring in the estimation algorithm

One complication arises from the fact that due to censoring $U_i(\psi)$ cannot always be computed from the observed data using the above model. One cannot simply replace T_i by X_i in the above model and calculate the respective value, say $U_i^x(\hat{\psi})$ from the model because then $U_i^x(\hat{\psi})$ would not be independent of R_i and the assumptions for the estimation would not hold. Instead the censoring is handled as follows.

i) If $\delta_i = 1$ then calculate

$$C_i(\psi) = \min(C_i, C_i e^{\psi})$$
$$X_i(\psi) = \min(U_i(\psi), C_i(\psi))$$
$$\delta_i(\psi) = I(X_i(\psi) = U_i(\psi))$$

ii) If $\delta_i = 0$ then calculate

$$C_i(\psi) = \min(C_i, C_i e^{\psi})$$
$$X_i(\psi) = C_i(\psi)$$
$$\delta_i(\psi) = 0$$

By these definitions, $C_i(\psi)$ is independent of $U_i(\psi)$ and R_i. The new censoring time $C_i(\psi)$ can be interpreted as the maximum $U_i(\psi)$ that can be calculated which is smaller than C_i no matter how long individual i is on the randomized active drug. Both $X_i(\psi)$ and $\delta_i(\psi)$ can be calculated based on observed data and furthermore $\{X_i(\psi),\ \delta_i(\psi)\} \coprod R_i \mid Strata_i$. Thus the estimation can be performed by calculating $X_i(\psi)$ and $\delta_i(\psi)$ for the various values of ψ within the different strata of the baseline risk factor and proceed as described in the estimation section.

This way of dealing with censoring was introduced by Robins and Tsiatis 1991. Note that some extra censoring will occur due to nature of handling the censoring times in this estimation procedure. This causes some loss of power.

Summary of the method and interpretation

Drug effect is obtained by estimating the multiplicative factor; which would be interpreted as either a relative increase or decrease in survival if one took active drug (Lutathera) compared to taking Octreotide LAR. The multiplicative factor is determined by repeatedly reconstructing the anticipated survival duration of all patients by varying degrees of the factor, until both survival curves (Lutathera arm and Octreotide LAR) can no longer be distinguished, i.e., as if all patients only received Octreotide LAR.

This method maintains the original randomized-group definitions and thus preserves the validity of between group comparison. It provides a randomization based estimate of drug effect corrected for the bias due to crossover (under the assumption that the effect is multiplicative in time). This estimate is valid even in presence of outcome dependent drug change. Of note, this method requires extra censoring (i.e., some events become censored) which impacts the precision of the drug effect estimate.

5.4.3.2 Marginal Structural Cox Proportional Hazards Model using the Inverse Probability of Censoring Weighting (IPCW)

The Marginal Structural Cox Proportional Hazards Model using the Inverse Probability of Censoring Weighting (IPCW) will also be considered (Hernan et al 2000) to account for the treatment cross-over.

We follow Hernan et al 2000 and Korhonen et al 1999 for the notation. The treatment assignment indicator is denoted by R_i and takes the value 0 if allocated to Octreotide LAR and 1 if allocated to active treatment (Lutathera). The baseline covariates to be included in the model are jointly denoted as matrix $V_{i,}$ containing the variables Ki67 and Karnosky score. We will consider two time-dependent covariates: Karnofsky score and investigators assessment of disease progression which are collectively denoted as matrix L_i.

Each individual is followed from the time of randomization (DR_i) until death or the last date of contact (EOF_i) and the fixed closing date (CD_i) which ever comes first. The censoring time, $C_i = min(EOF_i - DR_{i,} CD_i - DR_i)$, is measured in days and is assumed to be independent of the treatment assignment and also from the death time. Time to death (T_i) is the time from randomization to death which is also measured in days. We can only observe $X_i = \min(T_i, C_i)$ with $\delta_i = 1$ if $X_i = T_i$ and $\delta_i = 0$ otherwise. The follow-up time is divided in to intervals $k=0, 1, 2,\ldots, K$ where 0 denotes the baseline. The length of the intervals after baseline may vary but the same intervals are used for each individual. Some of the subjects randomized to the Octreotide LAR arm switch to the active treatment at some point after randomization. Switching to the active treatment is potentially related to the patient's prognosis and thus cannot be assumed independent of the death time. The time to switching to the active treatment is denoted by S_i and for subjects who do not switch to the active treatment arm we define $S_i = X_i$. We denote

$A_i(k)=0$, if the subject did not receive active treatment during the time interval k and $A_i(k)=1$, if the subject received active treatment during the time interval k. For subjects randomized to the active treatment arm $A_i(k)=1$ for all k until X_i. Similarly, for subjects randomized to the Octreotide LAR arm $A_i(k)=0$ for all time intervals k prior to S_i and $A_i(k)=1$ thereafter for all k until X_i. With these notations we call $A_i(X_i)=\{A_i(u);0\le u\le X_i\}$ subjects observed active treatment until the end of follow-up and $\overline{A}_i(k)=\{A_i(u);u=0,1,...,k-1\}$ a subject's active treatment history until the start of the kth time interval. The time-dependent covariate history is similarly denoted by $\overline{L}_i(k)=\{L_i(u);u=0,1,...,k-1\}$ and subjects observed time-dependent covariates until the end of follow-up as $L_i(X_i)=\{L_i(u);0\le u\le X_i\}$.

Thus the observed data for each subject i are $\{R_i, V_i, L_i(X_i), C_i, X_i, \delta_i, A_i(X_i)\}$.

The usual ITT approach for estimating the treatment effect concerning overall survival is likely to be biased towards zero due to considerable amount of switching to the active treatment in the Octreotide LAR arm. In order to trying to estimate the treatment effect one might think of estimating the time varying effect of treatment received on overall survival possibly using Cox's proportional hazards model to model the death risk as a function of the treatment history $\overline{A}_i(k)=\{A_i(u);u=0,1,...,k-1\}$. This approach is likely to be biased whenever there is a time-dependent covariate that is (i) both a risk factor for mortality and (ii) which also predicts subsequent treatment $A_i(u)$ and (iii) at the same time the treatment history $\overline{A}_i(k)$ predicts the subsequent level of the time-dependent covariate $L_i(u)$. In this study the Kanrofsky performance status after baseline, Ki67 and investigator's assessment of disease progression are such time-dependent covariates.

One approach trying to finding an unbiased estimate of the treatment effect is to use the marginal structural Cox proportional hazards model where the death risk is modeled using a conventional Cox's proportional hazards model using time-dependent case weights for those who did not switch to active treatment but continue to remain on their original randomized treatment.

The marginal structural Cox model is specified as follows:

$$\lambda(t\mid\overline{A}(t),V)=\lambda_0(t)\exp(\gamma_1 A_i(t)+\gamma_2 V_i);t\le S_i$$

where a subject stays at risk for death until (s)he switches to the active treatment. Thus, the whole follow-up experience from randomization to $X_i=\min(T_i, C_i)$ is used from subjects randomized to the active treatment arm. But, in the Octreotide LAR arm only the follow-up experience from randomization to $\min(S_i, X_i)$ is used in the marginal structural Cox model. This

means in practice that in the Octreotide LAR arm the data after switching to the open label active treatment is discarded. Each subject is given in this model the case weight indicating the cumulative probability of not switching to the active treatment at time *t*. Unfortunately, the PROC PHREG procedure does not allow to specify time-dependent weights. Therefore one needs to divide the follow-up time into discrete time intervals and fit the above model using the PROC GENMOD procedure which allows time-dependent weights.

The case weights are estimated using the IPCW scheme for the probability of switching in the Octreotide LAR arm. In the IPCW, the probability of not switching is modeled using two separate logistic regression models as follows:

$$sw_i(k) = \prod_{u=0}^{k} \frac{\Pr(A_i(u) = a_i(u) \mid \overline{A}_i(u) = (0,\ldots,0)^T, V_i)}{\Pr(A_i(u) = a_i(u) \mid \overline{A}_i(u) = (0,\ldots,0)^T, V_i, \overline{L}_i(u))}$$

$$a_i(u) = \begin{cases} 1 & \text{if subject crossed - over at time k} \\ 0 & \text{if subject did not crossed - over at time k} \end{cases}$$

The first logistic regression model (i.e., the nominator model) uses only the baseline covariate information to describe the probability of switching. The second logistic regression model (i.e., the denominator model) uses both baseline covariate information and time-dependent covariate information. The estimation of these stabilized weights can easily be done with the PROC LOGISTIC procedure in SAS.

Note that the estimation of the stabilized weights is only needed in the Octreotide LAR arm. In the active treatment arm we set the stabilized weights to 1 for all subjects and for all discrete time periods.

This type of weighting effectively creates, for a risk set at time t, a pseudo-population in which $L_i(u)$ no longer predicts the receipt of active treatment at time t (that is, $L_i(u)$ is not a confounder). If we can be comfortable assuming that after having modeled the dependence of the subsequent treatment $A_i(u)$ with the stabilized weights then under this assumption of no unmeasured confounders, the coefficient γ_1 of $A_i(u)$ in the marginal structural Cox model represents the causal association between the receipt of active treatment and mortality on the log-hazard ratio scale.

Note: For the estimation of the stabilized weights and the discrete version of the marginal structural Cox model one has to model the time-dependent intercepts using either a smooth function of time, such as a cubic spline or by combining time intervals. Otherwise the estimation may not be possible and also the asymptotic theory may break down.

5.5 Determination of missing adequate assessments

The term ‘missing adequate assessment’ refers to assessments that are not done or for which the overall lesion response is ‘NE’. ‘Missing adequate assessment’ will also be referred to as ‘missing assessment’.

The PFS censoring and event date options depend on the presence and the number of missing tumor assessments.

An exact rule to determine whether there are no, one or two missing TAs is therefore needed. This rule is based on the interval between the last adequate tumor assessment (LATA) date and the event date. The scheduled date of tumor assessments (in weeks from randomization), protocol specified window for tumor assessments, and the thresholds for LATA that belong to a visit can be found in Table 5.5-1 below.

Table 5.5-1 Schedule for tumor assessment and time windows

Assessment schedule	Schedule week – 1 week	Schedule week (weeks from randomization)	Schedule week + 1 week	Threshold (weeks)*
Baseline	N/A	N/A	N/A	N/A
Week 16	15	16	17	20
Week 24	23	24	23	28
Week 36	35	36	37	42
Week 48	47	48	49	54
Week 60	59	60	61	66
Week 72	71	72	73	78
Week 72+12x**	71+12x	72+12x	73+12x	78+12x

* The mid-point between current and next visit (except for baseline) and the upper limit for LATA to be matched to a certain scheduled assessment, e.g., if LATA is at Week 21, this is after the threshold for Week 16, and before that for Week 24, so the matching scheduled assessment is Week 24.

** For all scheduled assessments after week 72, such as Week 84 (x = 1), Week 96 (x = 2), etc...

To calculate the number of missing tumor assessments, the LATA before an event is matched with a scheduled tumor assessment using the time window in the table above (essentially whichever scheduled assessment it is closest to). Two thresholds, D1 and D2 are calculated for that scheduled assessment based on the protocol-specified scheduled and windows:

- An event after LATA+D1 will be considered as having ≥ 1 missing assessment
- An event after LATA+D2 will be considered as having ≥ 2 missing assessments

Since there is a change of schedule for tumor assessments at Week 16 and Week 24, D1 and D2 are defined differently depending on when LATA happens.

Rule 1: if LATA happens before Week 16 (if LATA is at baseline, then LATA starts at Day 1)

- D1 = 16 weeks + 14 days = 126 days
- D2 = 16 weeks + 8 weeks + 14 days = 182 days

Rule 2: if LATA occurs between Week 16 and Week 24

- D1 = 8 weeks + 14 days = 70 days
- D2 = 8 weeks + 12 weeks + 14 days = 154 days

Rule 3: if LATA occurs from Week 24 onward

- D1 = 12 weeks + 14 days = 98 days
- D2 = 12 weeks + 12 weeks + 14 days = 182 days

The date of next scheduled assessment is defined as the date of the late adequate tumor assessment plus the protocol specified time interval for assessments. The protocol specified time interval for tumor assessments is captured in the above table (± 1 week).

6 References

1. Amit O, Mannino F, Stone AM, Bushnell W, Denne J, Helterbrand J and Burger HU. Blinded independent central review of progression in cancer clinical trials: results from a meta-analysis. European Journal of Cancer 2011; 47: 1772-1778.
2. Armitage P, McPherson CK and Rowe BC (1969). Repeated significance tests on accumulating data. J .R. Statist. Soc. A, 132, 232-244.
3. Aithal GP, Watkins PB, Andrade RJ, et al. Case definition and phenotype standardization in drug-induced liver injury. Clin Pharmacol Ther 2011;89:806-15.
4. Brookmeyer R and Crowley J (1982). A Confidence Interval for the Median Survival Time. Biometrics, 38, 29 - 41.
5. Clopper CJ and Pearson ES (1934). The use of confidence or fiducial limits illustrated in the case of the binomial. Biometrical, 26, 404-413.
6. Collet D (1994). Modelling survival data in medical research. London, Chapman & Hall.
7. Dodd EL, Korn LE, Freidlin B, Gray R, Bhattacharya S. An audit strategy for progression-free survival. Biometrics, 2011, 67 (3): 1092-99.
8. FDA briefing Document Oncologic Drugs Advisory Committee Meeting, July 24, 2012, Evaluation of radiologic review of progression-free survival in Non-hematologic malignancies. http://www.fda.gov/downloads/advisorycommittees/committeesmeetingmaterials/drugs/oncologicdrugsadvisorycommittee/ucm311141.pdf
9. Fleming TR, Harrington DP and O'Brien PC (1984). Designs for group sequential tests. Controlled Clinical Trials, 5, 348-361.
10. Greenland S, Lanes S and Jara M (2008). Estimating effects from randomized trials with discontinuations: the need for intent-to-treat design and G-estimation. Clinical Trials, 5, 5–13.

11. Glimm E, Maurer W and Bretz F (2010). Hierarchical testing of multiple endpoints in group-sequential trials. Statistics in Medicine, 29, 219-228.
12. Hernan MA, Brumback b, Robins JM (2000). Marginal structural models to estimate the causal effect of zidovudine on the survival of HIV-positive men. Epidemiology 11:561-570.
13. Hwang IK, Shih WJ and De Cani JS (1990). Group sequential designs using a family of type 1 error probability spending functions. Statistics in medicine, 9(12), 1439-1445.
14. Kalbfleisch JD and Prentice RL. The Statistical Analysis of Failure Time Data (2002). Wiley Series in Probability and Statistics.
15. Korhonen P, Laird NM and Palmgren J. Correcting for non-compliance in randomized trials: an application to the ATBC study (1999). Statistics in Medicine, 18, 2879-2897.
16. Lan KKG, DeMets DL (1983). Discrete sequential boundaries for clinical trials. Biometrika, 70, 649-53.
17. Mantel, N. and Joseph L Fleiss (1980). Minimum expected cell size requirements for the Mantel-Haenszel one degree-of-freedom Chi-Square Test and a related rapid procedure. American Journal of Epidemiology, 112, 129-134.
18. Neuenschwander B, Branson M and Gsponer T (2008). Critical aspects of the Bayesian approach to Phase I cancer trials. Statistics in Medicine, 27, 2420-2439.
19. O'Brien PC and Fleming TR (1979). A multiple testing procedure for clinical trials. Biometrics, 35, 549-53.
20. Oncology Guideline for Safety Analyses for exposure adjustment methodology, Novartis
21. Peto R, Pike MC, Armitage P, Breslow NE, Cox DR, Howard SV, Mantel N, McPherson K, Peto J and Smith PG (1976). Design and analysis of randomized clinical trials requiring prolonged observation of each patient. British Journal of Cancer, 34, 585-607.
22. Robins JM and Finkelstein DM (2000). Correcting for noncompliance and dependent censoring in an AIDS Clinical Trial with inverse probability of censoring weighted (IPCW) log-rank tests. Biometrics, 56(3),779-88.
23. Robins JM and Tsiatis AA (1991). Correcting for non-compliance in randomized trials using rank preserving structural failure time models (RPSFTM). Commun. Statist.-Theory Meth., 20(8), 2609-31.
24. Smith B, Vandenhende FR, DeSante KA, Farid NA, Welch PA, Callaghan JT and Forgue ST (2000). Confidence Interval Criteria for Assessment of Dose Proportionality. Pharmaceutical Research, 17(10), 1278-83.
25. Spiegelhalter DJ, Abrams KR and Myles JP (2004). Bayesian Approaches to Clinical Trials and Health-care Evaluation. Chichester, Wiley.
26. Strosberg J, Wolin E, Chasen B, et al. Health-Related Quality of Life in Patients With Progressive Midgut Neuroendocrine Tumors Treated With 177Lu-Dotatate in the Phase III NETTER-1 Trial. J Clin Oncol. 2018;36(25):2578-2584. doi:10.1200/JCO.2018.78.5865

27. Vittinghoff E and McCulloch CE, Relaxing the Rule of Ten Events per Variable in Logistic and Cox Regression, American Journal of Epidemiology, Volume 165, Issue 6, 15 March 2007, Pages 710–718, https://doi.org/10.1093/aje/kwk052
28. Yadegarfar G, Friend L, Jones L, et al. Validation of the EORTC QLQ-GINET21 questionnaire for assessing quality of life of patients with gastrointestinal neuroendocrine tumours. Br J Cancer. 2013;108(2):301-310. doi:10.1038/bjc.2012.560
29. Zhang JJ, Chen H, He K, Tang S, Justice R, Keegan P, Pazdur R and Sridhara R. Evaluation of blinded independent central review of tumor progression in oncology clinical trials: a meta-analysis. Therapeutic innovation & regulatory science: 2012, 47(2): 167-174.
